# Combination therapy with budesonide and acetylcysteine alleviates LPS-induced acute lung injury via the miR-381/NLRP3 molecular axis

**DOI:** 10.1371/journal.pone.0289818

**Published:** 2023-08-09

**Authors:** Huimin Yu, Meifen Lv, Shiying Zhang, Kaiwen Zou, Yan Qian, Shaokun Lv

**Affiliations:** Department of Rehabilitation Medicine, Qujing No.1 Hospital, Qujing, Yunnan, China; University of the Pacific, UNITED STATES

## Abstract

**Background:**

Acute lung injury (ALI) usually has a high morbidity and mortality rate, but the current treatment is relatively scarce. Both budesonide (Bud) and N-acetylcysteine (NAC) exhibit protective effects in ALI, so we further investigated whether they have a synergistic effect on ALI when used together.

**Methods:**

Establishment of a rat model of ALI with Lipopolysaccharide (LPS). Bud and NAC were administered by nebulized inhalation alone or in combination. Subsequently, HE staining was performed to observe the pathological changes in lungs of rat. Evans blue staining was implemented to assess alveolar permeability, and the pulmonary edema was assessed by measuring the ratio of wet to dry weight of the lung. Moreover, a TUNEL kit was served to test apoptosis in lung tissues. Western blot and immunohistochemistry were analyzed for expression of scorch-related proteins and NLRP3 in lung tissue, respectively. ELISA was implemented to detect inflammatory factor levels in BALF. and RT–qPCR was utilized to assess the expression level of miR-381. After stable transfection of miR-381 inhibitor or OE-NLRP3 in BEAS-2B treated with LPS, Bud and NAC, miR-381 expression was assessed by RT–qPCR, scorch death-related protein expression was measured by western blot, cell proliferation/viability was assayed by CCK-8, apoptosis was measured by flow cytometry, and ELISA was implemented to assess inflammatory factor levels. Furthermore, the Dual-luciferase assay was used to verify the targeting relationship.

**Results:**

Bud and NAC treatment alone or in combination with nebulized inhalation attenuated the increased alveolar permeability, pulmonary edema, inflammatory response and scorching in LPS-induced ALI rats, and combined treatment with Bud and NAC was the most effective. In addition, combined treatment with Bud and NAC upregulated miR-381 expression and inhibited NLRP3 expression in cellular models and LPS-induced ALI rats. Transfection of the miR-381 inhibitor and OE-NLRP3 partially reversed the protective effects of Bud and NAC combination treatment on BEAS-2B cell proliferation inhibition, apoptosis, focal death and the inflammatory response.

**Conclusion:**

Combined Bud and NAC nebulization therapy alleviates LPS-induced ALI by modulating the miR-381/NLRP3 molecular axis.

## 1. Introduction

Acute respiratory failure in severe patients is usually caused by acute lung injury (ALI), in which patients lose most of the function of healthy lung organs, resulting in poor breathing and insufficient oxygen supply, resulting in a life-threatening condition. It is characterized by symptoms such as acute hypoxia, alveolar epithelial and endothelial damage, and neutrophil infiltration [1–3]. Despite intensive research on preclinical and clinical conditions and advances in treatment, therapeutic options remain extremely limited, and ALI morbidity and mortality remain high [4, 5]. Therefore, identification of new therapeutic approaches for ALI is urgent. Lipopolysaccharide (LPS) is an important inflammatory inducer that has been used in multiple studies to induce ALI models to investigate the mechanism of action and possible therapeutic approaches for ALI [6–8]. In the present study, we treated lung epithelial cell lines and SD rats with LPS to establish ALI cell and animal models for further studies.

Budesonide (Bud) and N-acetylcysteine (NAC) are both commonly used inhalers to treat lung diseases including chronic obstructive pulmonary disease, asthma, and bronchitis [9]. Both treatments inhibit inflammation in lung diseases [10, 11]. Inhalers been shown to inhibit inflammation in lung diseases [12]. Some studies have shown that Bud [13] and NAC [14] both reduce LPS-induced ALI by suppressing the inflammatory response. Because the incidence of lung diseases has always been at a high level, and there are many types of lung diseases, and the treatment methods also show certain limitations [15]. As a result, researchers are urgently seeking more treatments for various lung diseases. In the modern medical system, drug synergistic therapy can often enhance the therapeutic effect, and in many cases, it can achieve twice the result with half the effort and reduce the medical cost [16]. Considering that both Bud and NAC exhibit protective effects in ALI [17], we further investigated whether they could have a synergistic effect on ALI when used together.

Programmed inflammatory cell death usually contains multiple forms, and among them, scorch death has been reported more prominently, which is usually triggered by the activation of inflammatory vesicles, leading to the shear and multimerization of gasdermin family members, cell death by perforation, and proliferation of multiple inflammatory factors that promote the inflammatory response [18]. NLRP3 inflammatory vesicle activation is a hallmark event of cellular scorch death [19]. Meanwhile, the activation of inflammatory vesicles of NLRP3 has significant functional effects in the early pathophysiological process of ALI. Under its action, the multi-protein complexes in the cytoplasm are continuously processed and combined by a variety of inflammatory components [20]. Liu [19] et al. found that the AMPK-dependent pathway was an effective breakthrough to reduce the effects of NLRP3-mediated cell scorch, thereby inhibiting the buformin attenuates sepsis-induced ALI. Some scholars also suggested that NLRP3 activation-mediated cellular scorching has a great probability to be the target of precise treatment of ALI in the future [21]. Furthermore, NAC is capable of regulating the NLRP3 inflammatory vesicle activation for the treatment of diseases, including aortic aneurysms, sepsis to inhibit NLRP3 inflammatory vesicle activation [22]. However, whether Bud or NAC regulate NLRP3 inflammatory vesicle activation during the developmental process of ALI needs further exploration.

It is reported that protein-coding genes in organisms can be regulated by endogenous miRNAs, leading to messenger RNA degradation or disruption of the protein translation process [23]. Online prediction of miRNAs that are possible targets of NLRP3 mRNA using the "http://www.microrna.org/" website revealed that miR-381 has a target binding site with NLRP3. In the LPS-induced mouse model ALI, dexmedetomidine treatment reduced LPS-induced acute lung injury, reduced NLRP3 levels, reduced lung injury and inhibited the expression levels of inflammatory factors by miR-381-targeted NLRP3 [24].

Hence, the content of this study is mainly to describe miR-381/NLRP3 in ALI to reveal the role of Bud and NAC in the occurrence and development of ALI.

## 2. Materials and methods

### 2.1. Animal experiments and ethical statement

50 adult male Sprague-Dawley rats (6–8 weeks old, weighing 180–200 g) were purchased from Hunan Slaughter Jingda Laboratory Animal Co Ltd (Hunan, China) and housed in specific pathogen-free animal cages with constant temperature and humidity, a 12 h/ 12 h dark/ light cycle, adequate food and water, and after 7 d of acclimatisation, were randomly divided into NC group, ALI group, Bud group, NAC group, Bud+NAC group (n = 10 animals/group). Except for the NC group, the rats in all groups were used to establish the ALI model by LPS tracheal drip method [25], the rats were anaesthetized by intraperitoneal injection of 3% pentobarbital sodium (50 mg/kg), then fixed on the experimental table, the trachea was exposed along the midline of the neck and LPS was injected into the trachea by endotracheal intubation at a dose of 5 mg/kg, after which the rat was held upright and rotated vertically to ensure uniform distribution of the drug in the rat’s lungs. The rats were treated with Bud (0.36mg/kg, 2ml: 1mg*5 sticks, AstraZeneca Pty Ltd, Australia) and NAC (60mg/kg, 3ml: 0.3g*5 sticks, ZAMBON S.p.A, Italy) alone or in combination with a nebuliser 4h after surgery. The rats were observed every 2 hours so that they had a good physical condition and were free from extreme behaviour. At the end of the experiment 24 hours after administration, 5 rats in each group were randomly selected and executed by intraperitoneal injection of pentobarbital sodium (100 mg/kg) in an overdose and alveolar permeability was examined by Evans blue staining. The remaining rats were anaesthetized by intraperitoneal injection of sodium pentobarbital, the right lung was washed three times with PBS, bronchoalveolar lavage fluid (BALF) was collected, and the left lung tissue not washed with PBS was then removed by cervical dislocation for subsequent testing. All experimental rat protocols were in accordance with the ARRIVE guidelines and approved by the Animal Ethics Committee of Qujing Hospital, Kunming Medical University. All procedures were performed under pentobarbital sodium anaesthesia, care was taken to maintain the temperature of the rats during the procedures and every effort was made to minimise pain.

### 2.2. Histological analysis

Lung tissue was cut to 4 microns after fixation and embedding. During the experiment, try to ensure that the slices are not contaminated, and at the same time, ensure the storage time of the samples, so that they can be processed in time and follow-up experiments can be carried out in time. According to a previous study [26], the sections were dewaxed and stained with hematoxylin and eosin to observe lung pathological changes. The sections were stained according to the experimental steps indicated by TUNEL kit (Thermo Fisher Scientific, UA) to observe the death of apoptotic cells. In addition, the expression of NLRP3 in Sample tissues was assessed by staining for NLRP3 using immunohistochemical methods.

### 2.3. The assessment for ratio of wet to dry weight of the lung

The lung tissue was sucked dry with filter paper and weighed (wet weight, W). Subsequently, it was dried to constant weight and weighed (dry weight, D) to calculate W/D as an assessment of pulmonary edema.

### 2.4. Evans blue staining for alveolar permeability

First, 0.2 ml of 1% Evans blue dye solution was injected into rats through the tail vein, followed by heparinized saline 30 min later into the right ventricle to deplete the dye contained in the pulmonary vasculature. Then, lung tissue was isolated and placed in 2 ml of formamide solution for 48 h. Evans blue was extracted from the lung tissue, and the absorbance was read at 630 nm.

### 2.5. Cell and transfection

The human bronchial epithelial cell line BEAS-2B (American Type Culture Collection, USA) was selected for experiments. Treatment with 10 μg/ml LPS, 2 ng/ml Bud and 3 μg/ml NAC was applied, and subsequent experiments were performed after 24 h. The transfection kit used Lipofectamine 2000 kit (Invitrogen, UA); miR-381 inhibitor OE-NLRP3 and negative control were synthesized by Shanghai Ji’an Pharmaceutical Co., Ltd., China. The cells used for transfection were 293T cells, and the treated cell material was incubated at 37°C, usually for no more than two days.

### 2.6. RT–qPCR

TRIzol RNA extraction kit (Thermo Fisher Scientific, UA) is served by extracting the total RNA from the collected cells and tissues. The extraction steps were carried out in strict accordance with the reference experimental steps of the kit. Then, the first strand of cDNA was assembled using the total RNA of the sample as a template, and the cDNA obtained in is used as a template for qPCR amplification. The qPCR analyses were completed with the ABI 7300 system (Thermo Fisher Scientific, UA) and SYBR Premix Ex Taq (TaKaRa, Japan). Then, the expression levels of cells and tissues were calculated using the 2^–ΔΔCT^ method. The primer sequences of U6F/U6R and miR-381F/ miR-381R were displayed in the Table 1.

**Table 1:** Primer sequences.

Target	Sequence (F: Forward primer, R: Reversed primer)
U6	F: 5’-CTCGCTTCGGCAGCACA-3’
	R: 5’-AACGCTTCACGAATTTGCGT-3’
miR-381	F: 5’-TCAGACGACAACCGTCTGTG-3’
	R: 5’-AAAATTGAGCACCAACGGGC-3’

### 2.7. CCK-8

Lung epithelial cells (1×10^4^) were inoculated into 96-well plates andcultured for 24 h. After adding CCK-8 solution to the sample and modulating the enzyme label, the absorbance was measured at 450 nm wavelength. According to the absorbance values obtained in the experimental group and the control group, the Cell viability was calculated.

### 2. 8. Annexin V-FITC/PI double staining

The staining solution in Annexin V-FITC/PI kit (Thermo Fisher Scientific, USA) was incubated with cells, and then FITC and PI fluorescence were detected at 488 nm and 530 nm emission wavelength by flow cytometer, respectively.

### 2.9. ELISA

Levels of inflammatory factor including TNF-α, IL-1β and IL-6 in BALF and cell supernatant were assessed through the ELISA kit (both Abcam, UK).

### 2.10. Western blot

The total protein in each group of samples was extracted and subjected to mass determination. Then proceed to electrophoresis separation, mold transfer, primary antibody (1:2000. Abeam, UK) and secondary antibody (1:5000. Abeam, UK). After incubation, the membranes were finally assessed for expression semi-quantitatively by enhanced chemiluminescence (ECL) chromogenic and gel imaging.

### 2.11. Dual luciferase reporter gene

The binding site sequences of miR-381 together with NLRP3 were predicted through Bioinformatics tools http://www.microrna.org/. The selected luciferase reporter vector was connected with the NLRP 3 ′-UTR sequence containing the binding site of miR-381 and the binding site sequence to be mutated, respectively, and then the wild-type plasmid (WT) and mutant plasmid (MUT) were constructed. Plasmids were extracted, and the plasmids, miR-381 mimics and NCs were transfected two days later and 48 h later to detect the enzymes.

### 2.12. Statistical analysis

All experiments in this study were repeated at least three times and data were expressed as mean ± standard deviation. GraphPad Prism7.0 software was used for data analysis. t-test was used for comparison between two groups and one-way ANOVA was used for comparison between multiple groups, with p<0.05 indicating that the difference was statistically significant.

## 3. Results

### 3.1. Bud and NAC combined with nebulizer therapy provide better protection against ALI

**Fig 1:**
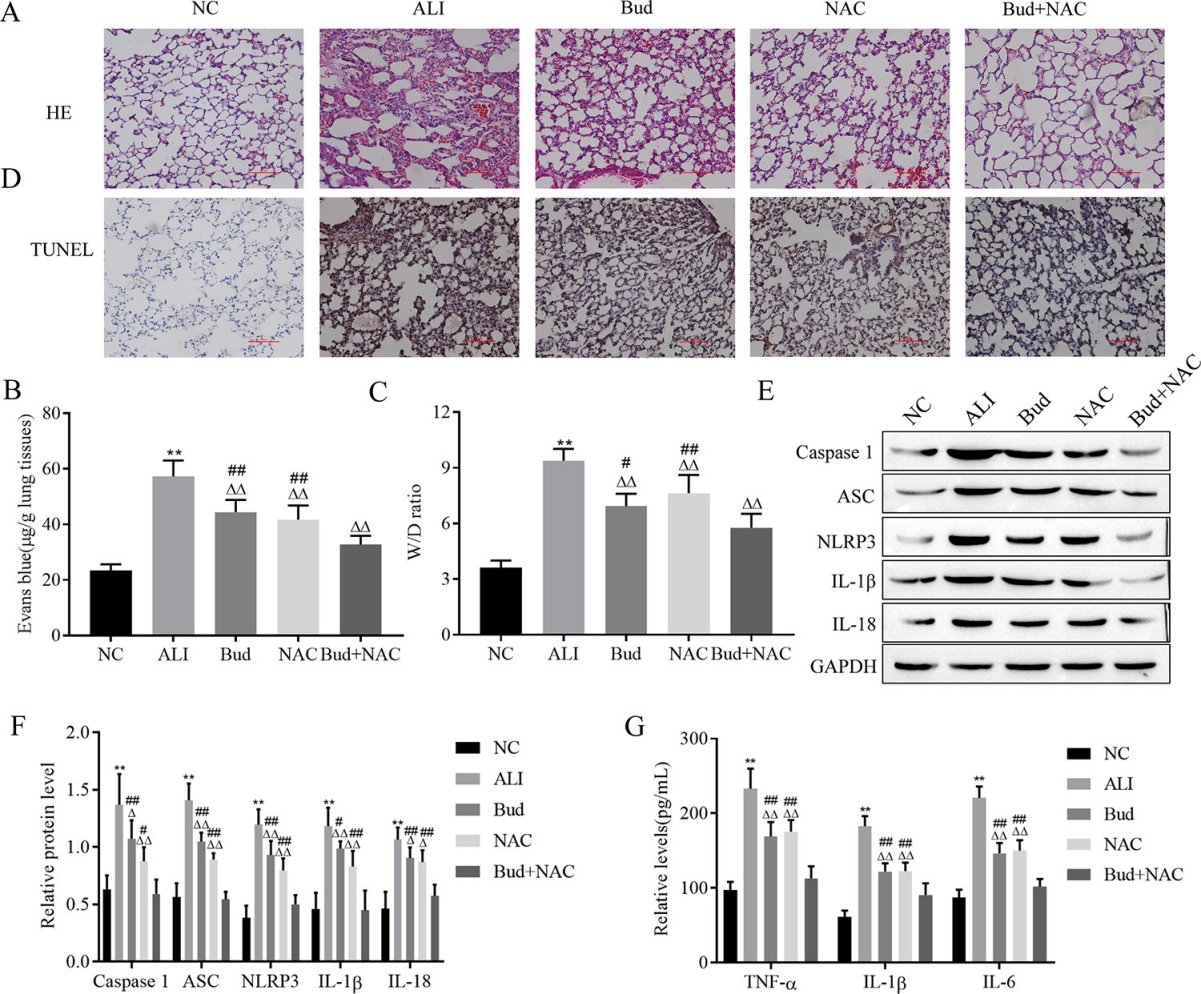
Combined treatment with Bud and NAC provides stronger protection against ALI. A. HE staining for lung pathological changes. B. Evans blue staining for alveolar permeability. C. Detection of ratio of wet to dry weight of the lung assessment for pulmonary edema. D. TUNEL kit for apoptosis. E-F. The expression of focal death-related proteins was detected by Western blot. G. ELISA for inflammatory factor levels. caspase -1: active-caspase -1; IL-1β: mature- IL-1β; IL-18: mature-IL-18. Vs NC, ** p < 0.01; Vs ALI, ^△^p < 0.05, ^△△^p < 0.01; Vs Bud+NAC ^#^p < 0.05, ^##^p < 0.01.

The results of HE staining showed that the alveolar structure and capillaries in the NC group were not distinguishing damaged. In contrast, the ALI group exhibited severe inflammation with disorganized and blurred alveolar structure, ruptured alveolar septa, inflammatory cell infiltration and abundant erythrocytes in the capillaries. Lung injury was reduced by either Bud or NAC treatment alone and their combined treatment, and the alveoli and interstitium displayed approximately normal alveolar structure. The combination of Bud and NAC produced the best results (Fig 1A). Since ALI usually has symptoms such as increased alveolar permeability and severe pulmonary edema, Evans blue staining and wet/dry weight ratio of lungs are used to judge alveolar permeability and pulmonary edema, respectively, and the results showed that the alveolar permeability of the ALI group increased, and the pulmonary edema was significantly aggravated. Nevertheless, the treatment group showed the opposite symptoms. Moreover, alveolar permeability and pulmonary edema were lowest in the Bud and NAC combination treatment group (Fig 1B and 1C). Apoptosis detection results showed that apoptotic cells were increased in the lung tissue of the ALI group, and this increase was attenuated in each treatment group, with the strongest effect observed in the Bud and NAC combination treatment group (Fig 1D). Since scorch death is an important factor in inducing ALI, the expression of the scorch death-related proteins Caspase-1, ASC, NLRP3, IL-1β, and IL-18 were detected by western blotting, and the results showed that expression of these proteins was significantly increased in the ALI group. Each treatment had a mitigating effect on LPS-induced cell scorch death, and the combined Bud and NAC treatment group showed the strongest effect (Fig 1E and 1F). Furthermore, ELISA detection is an efficient method to detect the levels of TNF-α, IL-1β and IL-6. The data showed that the above inflammatory factors were prominently increased in the ALI group and decreased in each treatment group, especially in the combined treatment group of bud and NAC (Fig 1G). These results demonstrate that combined treatment with Bud and NAC has a stronger defensive effect on LPS-induced ALI as well as inhibits scorch death more efficiently.

### 3.2. Combined treatment with Bud and NAC attenuates LPS-induced reduction in proliferative viability, apoptosis, scorching and inflammatory response in human bronchial epithelial cells

**Fig 2:**
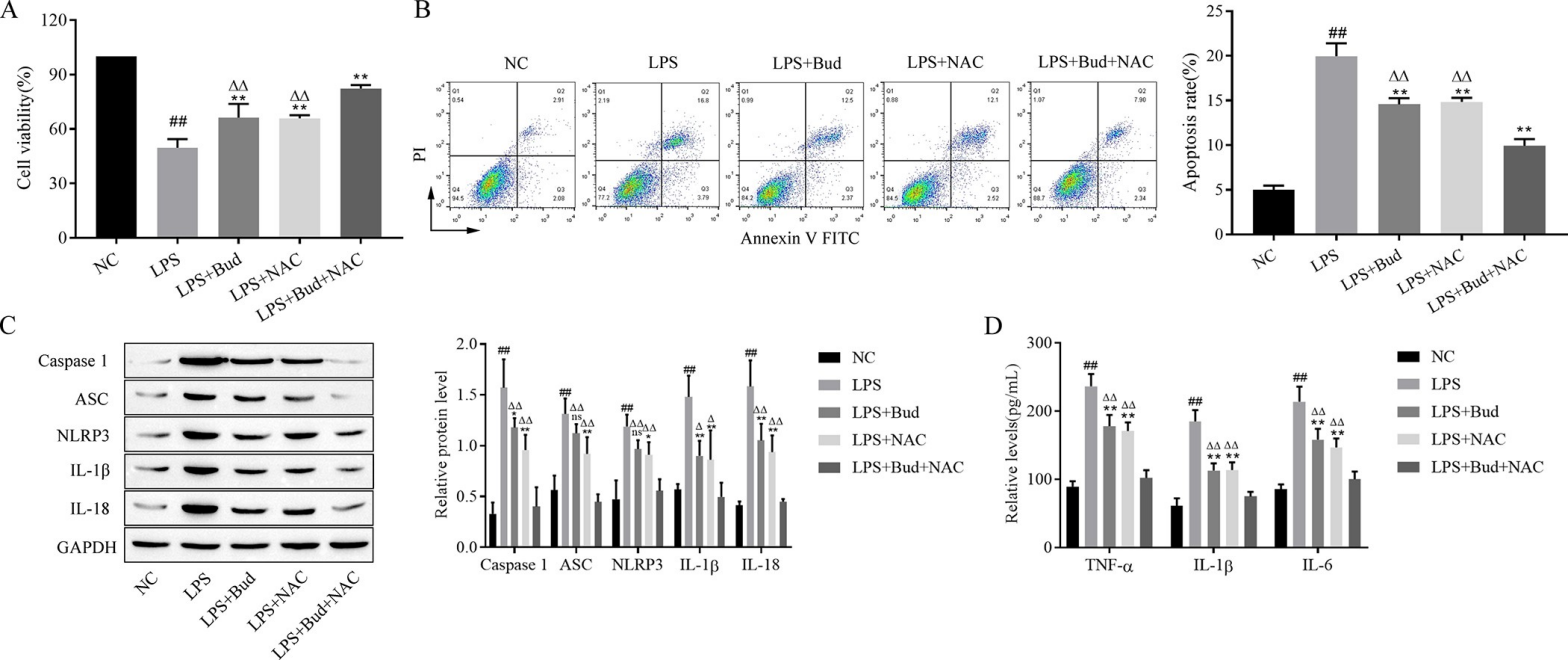
Combined treatment with Bud and NAC attenuates LPS-induced alterations in proliferative viability, apoptosis, scorching and inflammatory response in human bronchial epithelial cells. A. CCK-8 analysis of cell proliferation activity. B. Flow cytometry for apoptosis. C. Western blot for scorch death-related protein expression. D. ELISA for inflammatory factor levels. caspase -1: active-caspase -1; IL-1β: mature- IL-1β; IL-18: mature-IL-18. Vs NC, ^##^ p < 0.01; Vs LPS, ** p < 0.01; Vs LPS+Bud+NAC, ^△△^p < 0.01.

CCK-8 can measure cell proliferation activity, and analysis of experimental data shows that LPS remarkably reduces the proliferation activity of BEAS-2B. Bud and NAC treatment alone and together alleviated the LPS-induced reduction in proliferation viability, and the effect of Bud and NAC together was more significant (Fig 2A). Cell apoptosis was assessed by flow cytometry, from which it was found that LPS significantly induced apoptosis of BEAS-2B cells. Both Bud and NAC alone and cotreatment reversed LPS-induced apoptosis, and the effect of Bud and NAC cotreatment was more significant (Fig 2B). Moreover, the expression of the scorch death-related proteins was analyzed by western blotting, and the test data suggested that LPS treatment increased the expression of BEAS-2B Caspase-1, ASC, NLRP3, IL-1β, and IL-18 after LPS treatment. Bud and NAC treatment alone and together reversed the effect of LPS, and the combined treatment of Bud and NAC had a more significant effect (Fig 2C). Furthermore, ELISA detection as a method to detect the levels of inflammatory factors including TNF-α, IL-1β and IL-6 were prominently raised in the LPS-treated group. Both Bud and NAC alone and cotreatment reduced lipopolysaccharide-induced inflammatory response, while the inhibitory effect of Bud and NAC cotreatment was more significant (Fig 2D). Thus, cotreatment with Bud and NAC markedly attenuated LPS-induced alterations in proliferative viability, apoptosis, scorching and inflammatory response of human bronchial epithelial cells more strongly than either treatment alone.

### 3.3. Combined treatment with Bud and NAC exerts ALI-protective effects through upregulation of miR-381 expression

**Fig 3:**
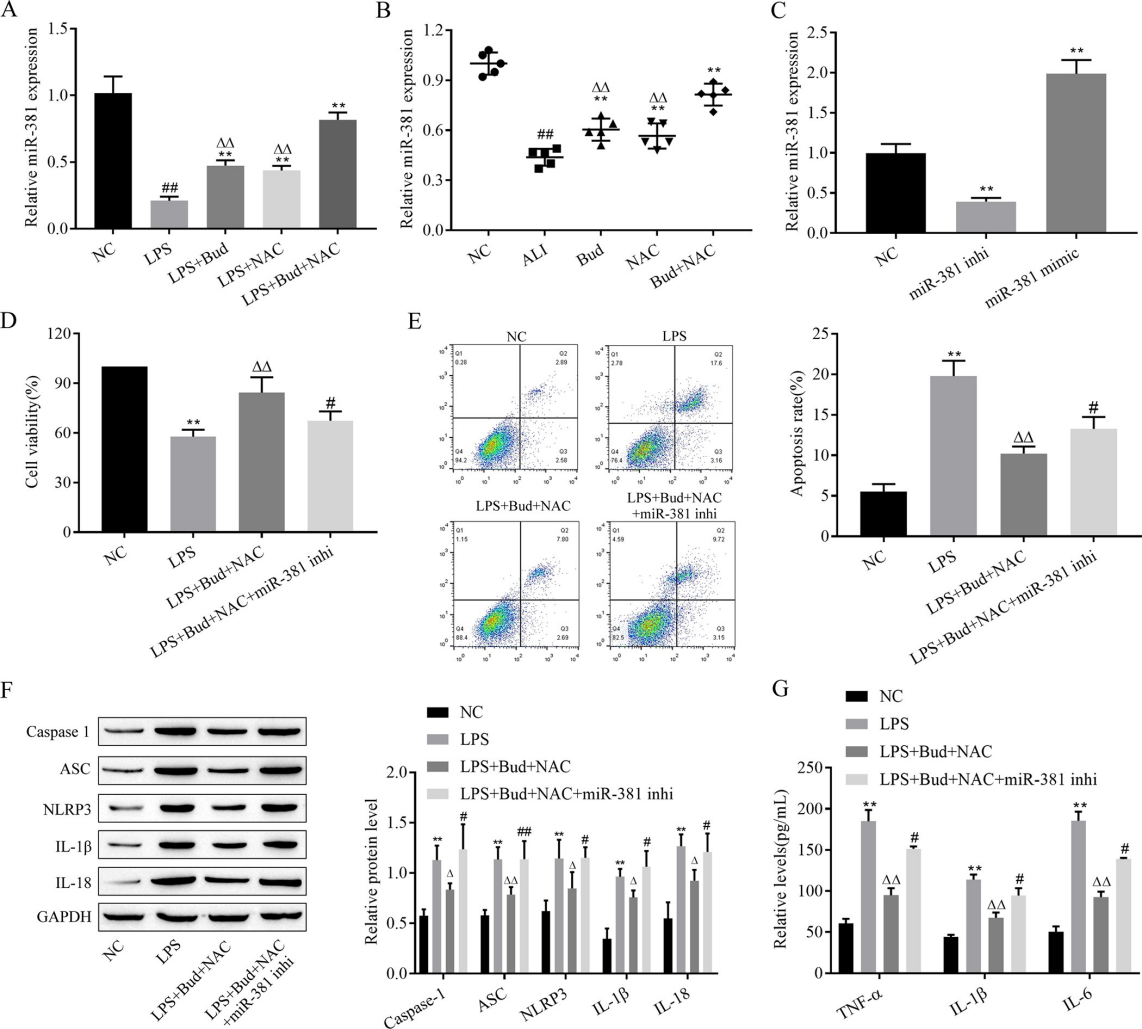
Combined treatment with Bud and NAC exerts ALI protective effects through upregulation of miR-381 expression. A-C. RT–qPCR to detect miR-381 expression in BEAS-2B cells. D. CCK-8 analysis of cell proliferation activity. E. Flow cytometry for apoptosis. F. Western blot for scorch death-related protein expression. G. ELISA for inflammatory factor levels. caspase -1: active-caspase -1; IL-1β: mature- IL-1β; IL-18: mature-IL-18. Vs NC, ^##^ p < 0.01; Vs LPS, ** p < 0.01;; Vs LPS+Bud+NAC, ^△△^p < 0.01.

RT–qPCR assays revealed that LPS decreased miR-381 expression in BEAS-2B cells. Bud and NAC treatment alone and together inhibited the effect of LPS on miR-381 expression, with Bud and NAC cotreatment exerting a more significant effect than either treatment alone (Fig 3A), similar results were obtained in animal experiments (Fig 3B). Since the mechanism of action of miR-381 in Bud and NAC on ALI is unclear, miR-381 inhibitors were transfected into BEAS-2B cells, which were then treated with Bud and NAC. First, the transfection efficiency was reflected by the RT-qPCR results, which showed that the expression of miR-381 was memorably up-regulated or down-regulated in cells after transfection with miR-381 inhibitor and miR-381 mimic, respectively. (Fig 3C). Furthermore, CCK-8, flow cytometry, western blotting as well as ELISA were applied to assess cell proliferation viability, apoptosis rate, scorch-related protein and inflammatory factor levels, respectively, and the experimental data showed that miR-381 inhibitor altered the influence of Bud and NAC on cell proliferation, apoptosis, charring and inflammatory reaction. (Fig 3D–3G). Therefore, combined Bud and NAC treatment clearly exerts ALI protective effects through upregulation of miR-381 expression.

### 3.4. miR-381 targets and regulates NLRP3 expression

**Fig 4:**
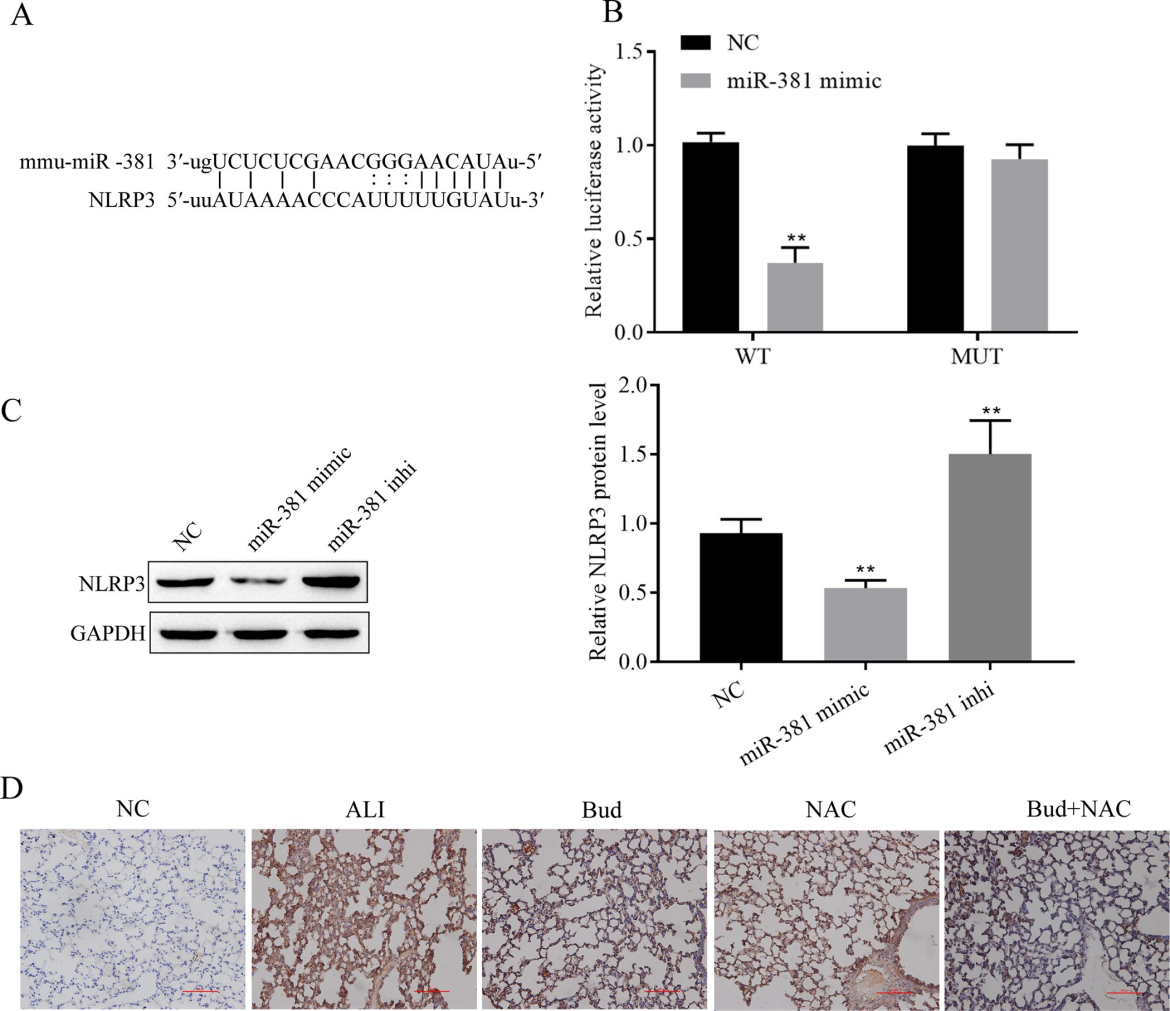
miR-381 targets and regulates NLRP3 expression. A. Targeted binding sites for miR-381 and NLRP3 are predicted by http://www.microrna.org/. B. Validation of the targeting relationship between miR-381 and NLRP3 with dual luciferase reporter genes. C. Western blot for NLRP3 expression. D. Immunohistochemical detection of NLRP3 expression in rat lung tissues. Vs NC, ** p < 0.01.

According to the bioinformatics tool http://www.microrna.org/, miR-381 was predicted to have targeted binding sites with NLRP3 (Fig 4A). The targeting relationship between miR-381 and NLRP3 was validated using a dual luciferase reporter gene, and the experimental results indicated that the miR-381 mimic reduced NLRP3 wild-type plasmid luciferase activity but had no significant effect on NLRP3 mutant plasmid luciferase activity (Fig 4B). The results of Western blot revealed that the miR-381 mimic dramatically reduced NLRP3 expression and miR-381 inhibitor significantly increased NLRP3 expression (Fig 4C). In addition, in Fig 2C we could find that LPS upregulated NLRP3 expression in BEAS-2B cells, while both Bud and NAC cotreatment and single treatment alleviated the LPS-induced increase in NLRP3 expression, and similar results were obtained in animal experiments (Fig 4D). Therefore, miR-381 clearly targets and downregulates NLRP3 expression.

### 3.5. Bud and NAC attenuate LPS-induced alterations in proliferative viability, apoptosis, focal death and the inflammatory response in human bronchial epithelial cells via miR-381/NLRP3

**Fig 5:**
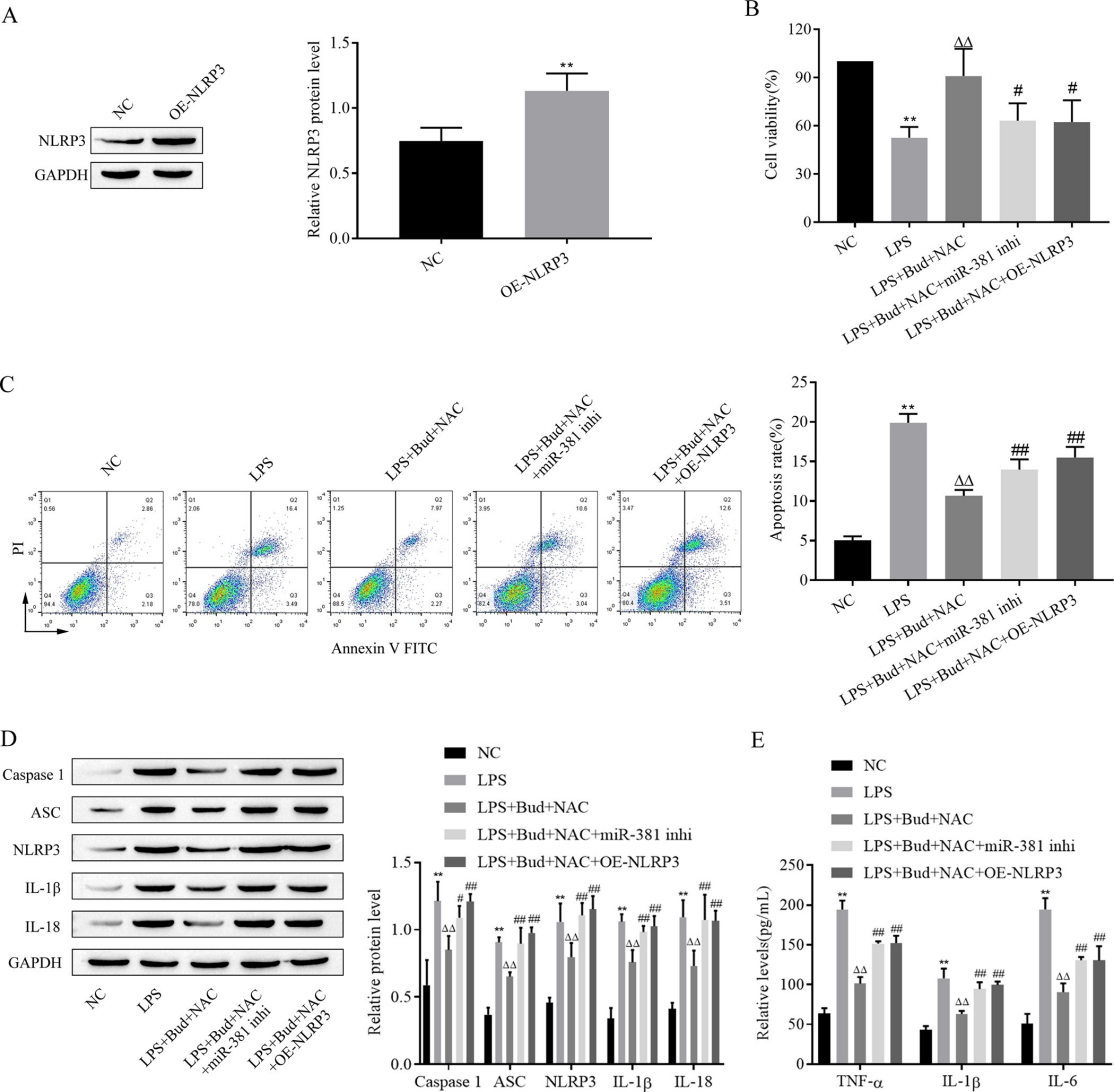
Bud and NAC attenuate LPS-induced alterations in the proliferative viability, apoptosis, focal death and inflammatory response of human bronchial epithelial cells via miR-381/NLRP3. A and D. western blot for the expression of the NLRP3 and pyrodeath-related protein; B. CCK-8 analysis of cell proliferation viability. C. Flow cytometry for apoptosis. E. ELISA for inflammatory factor levels. caspase -1: active-caspase -1; IL-1β: mature- IL-1β; IL-18: mature-IL-18. Vs NC, ** p < 0.01; Vs LPS, ^△△^p < 0.01; Vs LPS+Bud+NAC ^#^p < 0.05, ^##^p < 0.01.

The molecular mechanisms by which Bud and NAC attenuate LPS-induced reduction in proliferative viability, apoptosis, scorch death and inflammatory response in human bronchial epithelial cells through miR-381/NLRP3 were confirmed by transfection of miR-381 inhibitor and OE-NLRP3 into cells. Firstly, the expression of NLRP3 increased significantly after transfection of OE-NLRP3 from the results of Western blot (Fig 5A). After transfection, LPS, Bud and NAC treatments were administered followed by assessment of cell proliferation viability, apoptosis rate, scorch death-related protein and inflammatory factor levels by CCK-8, flow cytometry, western blot and ELISA, respectively. The miR-381 inhibitor and OE-NLRP3 both reversed the impacts of Bud and NAC on cell proliferation, viability, apoptosis, scorch death and the inflammatory response (Fig 5B–5E). Thus, Bud and NAC attenuate LPS-induced alterations in proliferation, viability, apoptosis, scorching and inflammatory responses in human bronchial epithelial cells via miR-381/NLRP3.

## 4. Discussion

Bud is an inhaled glucocorticoid with an extremely strong affinity for the glucocorticoid receptor (GR) and is highly lipophilic. Notably, there was an adequate lipid environment in the animal’s airways, which favored bud attachment and dissolution. Therefore, after entering the airway, the buds rapidly dissolve into cellular lipids in the airway and then rapidly esterify under the strong action of acidic substances, including oleic acid. Due to the low affinity of bud esters to GR, it is then slowly de-esterified to free buds to prolong lung exposure [27]. At present, the methods for the treatment of acute respiratory distress syndrome (ARDS) are not abundant, and the current treatment methods are not economical and efficient enough. It is worth noting that many researchers have carried out studies on the pharmacological effects of Bud in various lung tissue diseases. Among them, a clinical trial shows that early inhalation of Bud β agonist has a certain effect on the treatment and prevention of the disease [28], which also lays a foundation for the application research of Bud. Nevertheless, more research is still needed to further support the development and use of more Bud-related agents. Ansari [29]used NAC as a mucolytic agent with important functions in the clinical treatment of various pulmonary diseases. In addition, NAC plays an important role in clarifying the mechanism of free radicals, and it can regulate gene expression in cells and in vitro and inflammatory responses [30, 31]. Ansari [29] et al. found that NAC improved the mean partial pressure of oxygen, oxygen saturation, and clinical symptoms, including wheezing, dyspnea, in COPD patients. Moradi [32] et al. also found that NAC improved oxygen saturation and reduced mortality in patients with ALI. Our study demonstrated that both Bud and NAC attenuate pulmonary edema and alveolar permeability and reduced levels of scorching and inflammation in both LPS-induced ALI rat models and cellular models, while Bud and NAC cotreatment exerted stronger better protective effects.

Scorch pathways include classical scorch pathways and nonclassical scorch pathways. The classical scorch pathway involves the recruitment of pro-caspase-1 and apoptosis-associated spot-like proteins containing the cysteine recruitment structural domain (ASC) by inflammasome sensors (NLRP3, AIM2 or pyrin) to form NLRP3 inflammatory vesicles. NLRP3 inflammatory vesicles then process pro-caspase-1 into mature caspase-1, which activates gasdermin D to form pores in the cell membrane and release large amounts of inflammatory factors including IL-18 and IL-1β. The concentration of inflammatory factors is often proportional to the degree of inflammatory response, and the higher the concentration of inflammatory factors, the stronger the inflammatory response [33–35]. The nonclassical cell scorching pathway involves the direct cleavage of gasdermin D by caspase-4/5/11 to induce cell scorching [35–37]. Scorch death is an important mechanism leading to ALI, and inhibition of scorch death alleviates ALI [38]. For example, syringaresinol inhibited sepsis-induced ALI by inhibiting cellular scorching through the estrogen receptor-β signaling pathway [39]. In renal ischemia reperfusion induced ALI, propofol alleviates ALI by inhibiting pyroptosis via up regulating SIRT1 in the lung [40]. Our study showed that in LPS-induced rat models and lung epithelial cells, cell proliferation/viability was reduced, apoptosis was increased, the expression of the scorch-related proteins Caspase-1, ASC, NLRP3, IL-1β and IL-18 were increased, and the levels of the TNF-α, IL-1β and IL-6 were increased.

As previously described, NLRP3 is a key gene leading to cellular scorch and and also has a crucial function in ALI [13, 24]. In a rat model of spinal cord injury-induced ALI with NLRP3 inflammasome activation, administration of the dopamine D1 receptor agonist A-68930 significantly decreased NLRP3 inflammasome activation and reduced inflammatory cytokine levels and myeloperoxidase (MPO) activity, attenuating pulmonary edema and histopathology [41]. Xanthohumol effectively attenuated lung injury by activating the AMPK/GSK3β upregulation of the Nrf2 pathway, thereby inhibiting LPS-activated Txnip/NLRP3 inflammasomes and attenuating lung W/D ratio, neutrophil infiltration, MPO and malondialdehyde (MDA) formation, and oxidative dismutase (SOD) and GSH depletion [42]. The results showed that NLRP3 was increased at a higher level in LPS-induced ALI rat model and cell model. Furthermore, either Bud or NAC treatment alone decreased NLRP3 expression, but the inhibitory effect of combined Bud and NAC treatment on NLRP3 expression was more pronounced. Overexpression of NLRP3 partially restored the effects of combined Bud and NAC treatment on reduced proliferative viability, apoptosis, scorching of lung epithelial cells and inflammatory inhibition.

Non-coding small RNAs include a variety of RNA molecules including miRNAs and siRNAs. Among them, miRNAs are relatively small in molecular weight and are 18–23 nucleotides in length. They can silence or degrade mRNA, and then metabolize and degrade mRNA in biological processes. regulation of death [43]. Numerous studies have identified the important role of miRNAs in the development and progression of inflammatory lung diseases, including ALI [44]. For instance, miR-181a-5p directly inhibits Fas and human lung microvascular endothelial cell apoptosis while reducing inflammation and alleviating ALI [45]. Inflammation and apoptosis in an in vitro ALI model are be capable to increased by MiR-1246 through inducing NF-κB activation, and inhibiting Wnt/β-linked protein [46]. Moreover, miR-381 is also reported to play a therapeutic role in ALI [47]. We also concluded that miR-381 was detected at a low level in LPS-induced ALI rat models and lung epithelial cells. In addition, combined treatment with Bud and NAC alleviated ALI by upregulating miR-381 expression.

In summary, the combination of Bud and NAC therapy provided stronger remission of LPS-induced ALI than either Bud or NAC alone, and the mechanism occurred through miR-181/NLRP3. This study identifies the combination of Bud and NAC as a treatment for ALI and provides a new therapeutic theory scheme for ALI patients.

## Supporting information

S1 File (ZIP)

S2 File (ZIP)

S3 File (ZIP)

S4 File (ZIP)

S5 File (ZIP)

S1 Raw images (PDF)
