# Supplementary figures and images for: Combination therapy with budesonide and acetylcysteine alleviates LPS-induced acute lung injury via the miR-381/NLRP3 molecular axis (part 1 of 2)
Source: PLoS One. 2023 Aug 9;18(8):e0289818. doi: 10.1371/journal.pone.0289818 (PMC10411794; doi:10.1371/journal.pone.0289818)

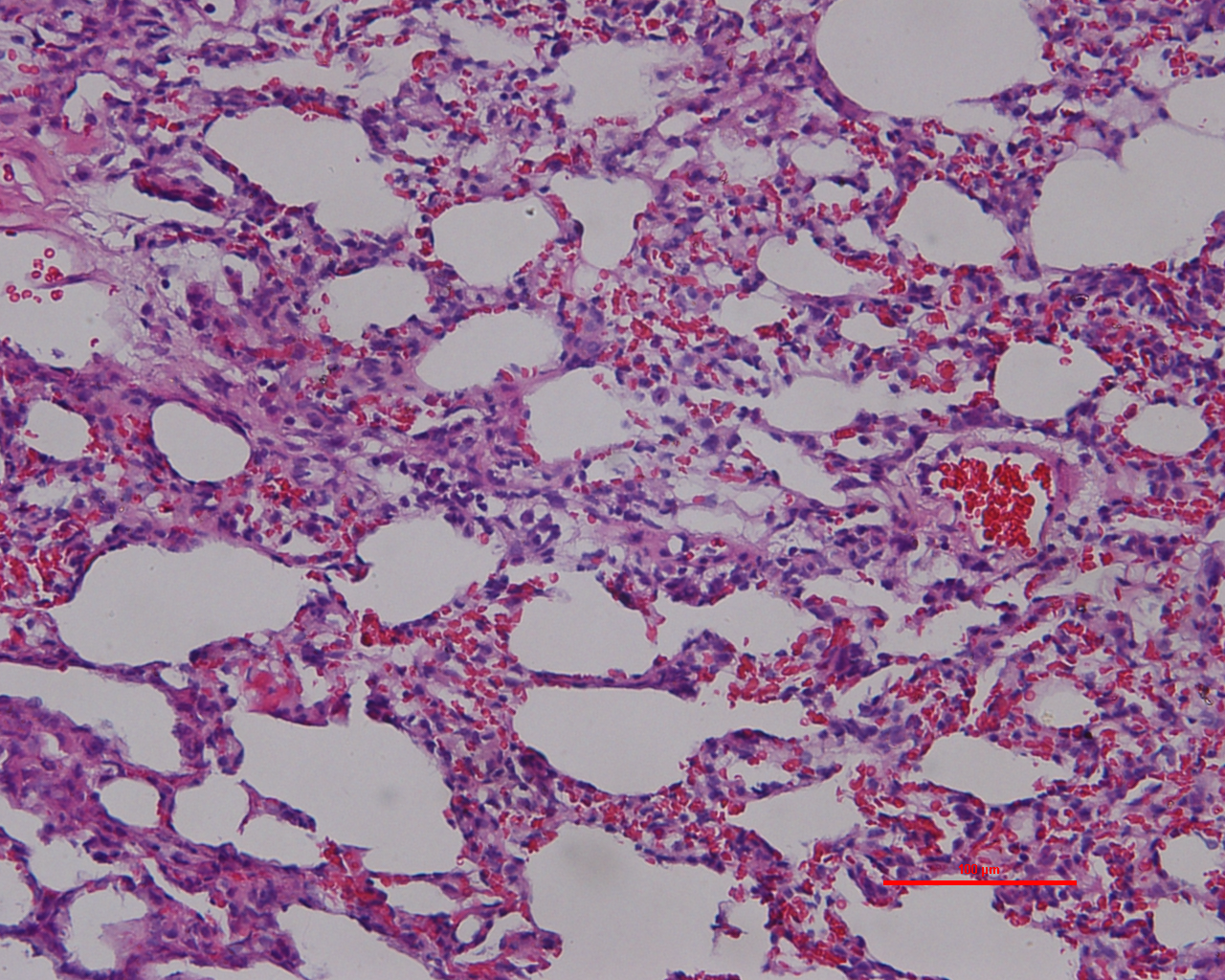

Supplement: S1 File — (ZIP) [file pone.0289818.s001.zip › S1 File. Fig1 Original data/image/1A/ALI.tif]

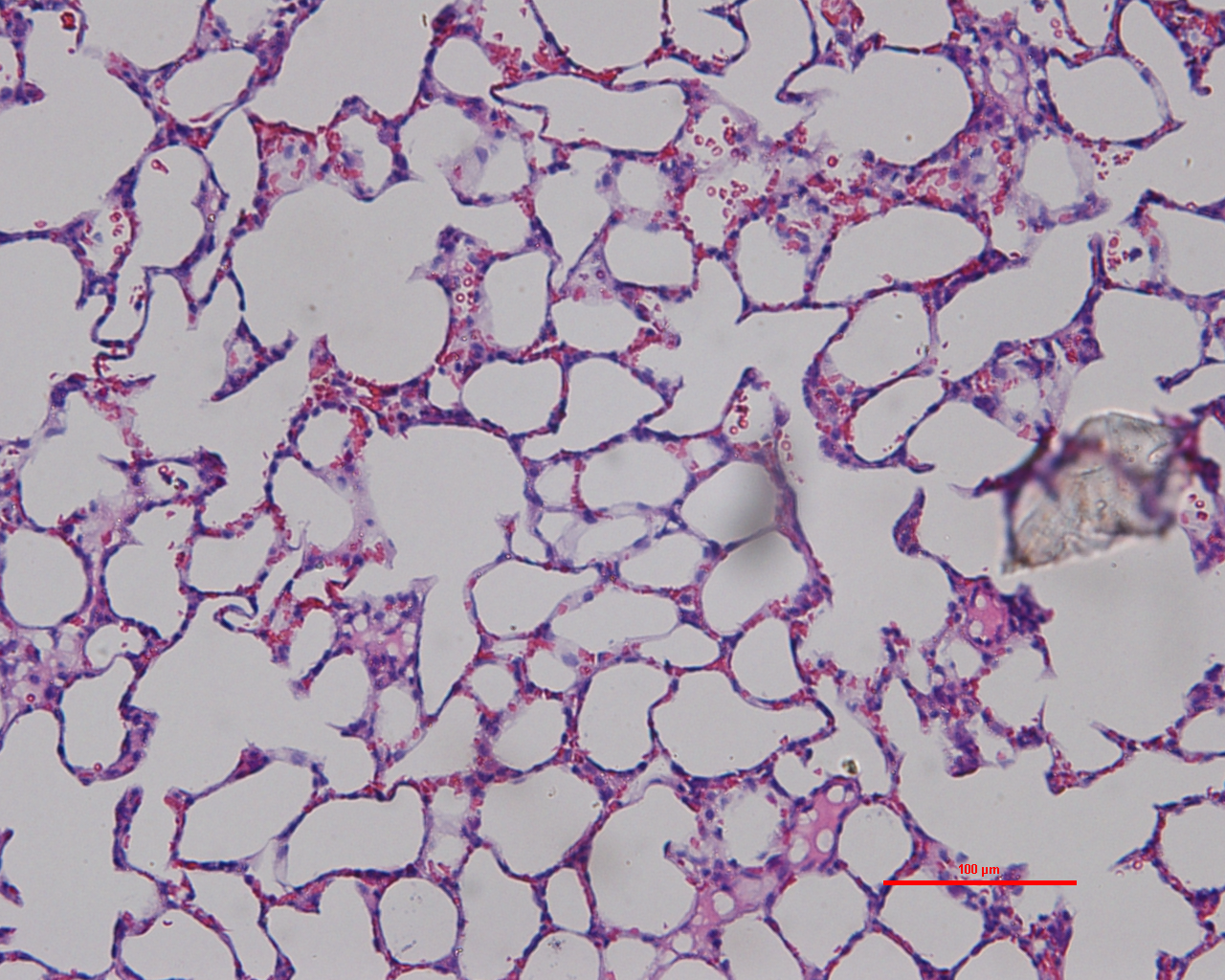

Supplement: S1 File — (ZIP) [file pone.0289818.s001.zip › S1 File. Fig1 Original data/image/1A/Bud+NAC.tif]

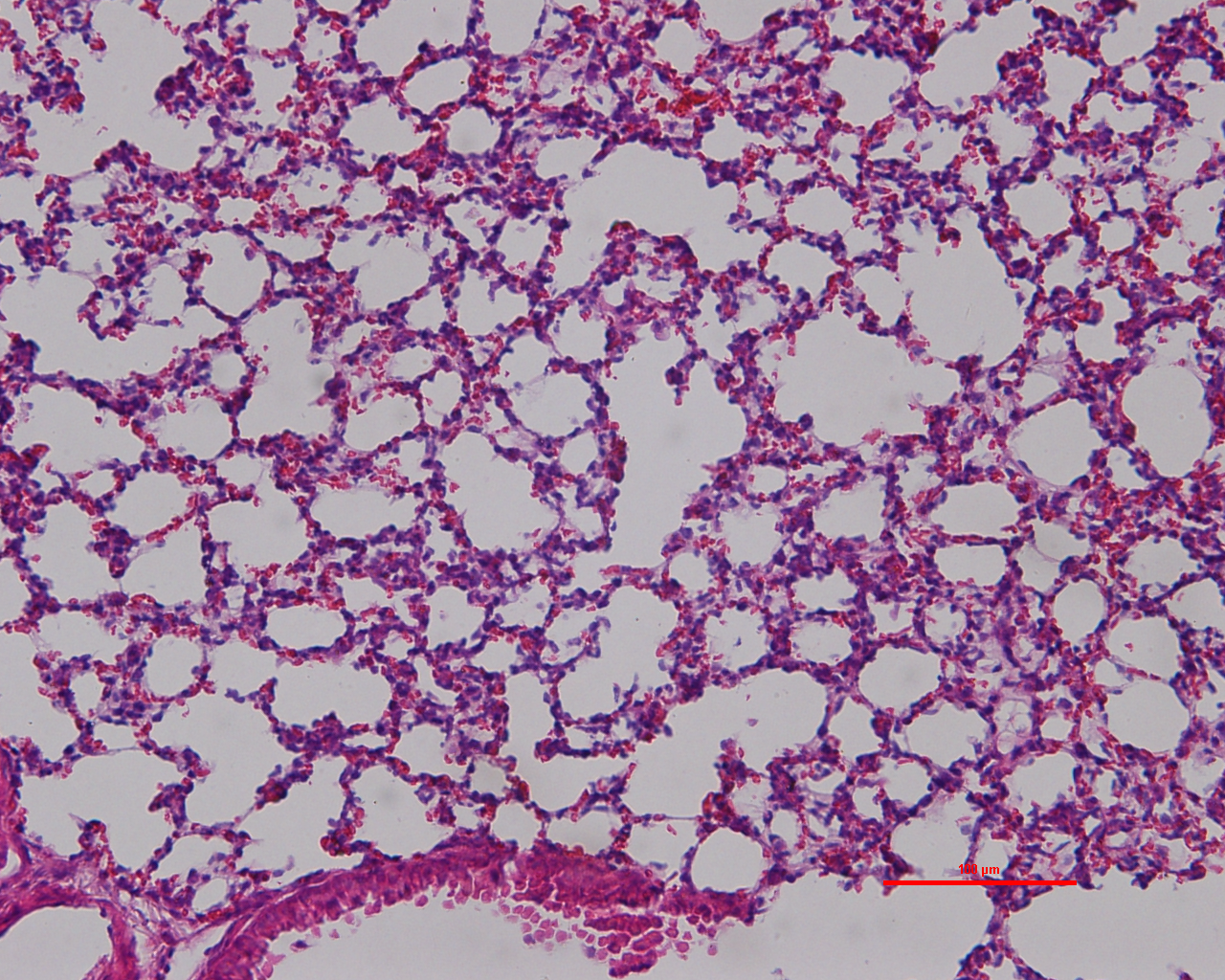

Supplement: S1 File — (ZIP) [file pone.0289818.s001.zip › S1 File. Fig1 Original data/image/1A/Bud.tif]

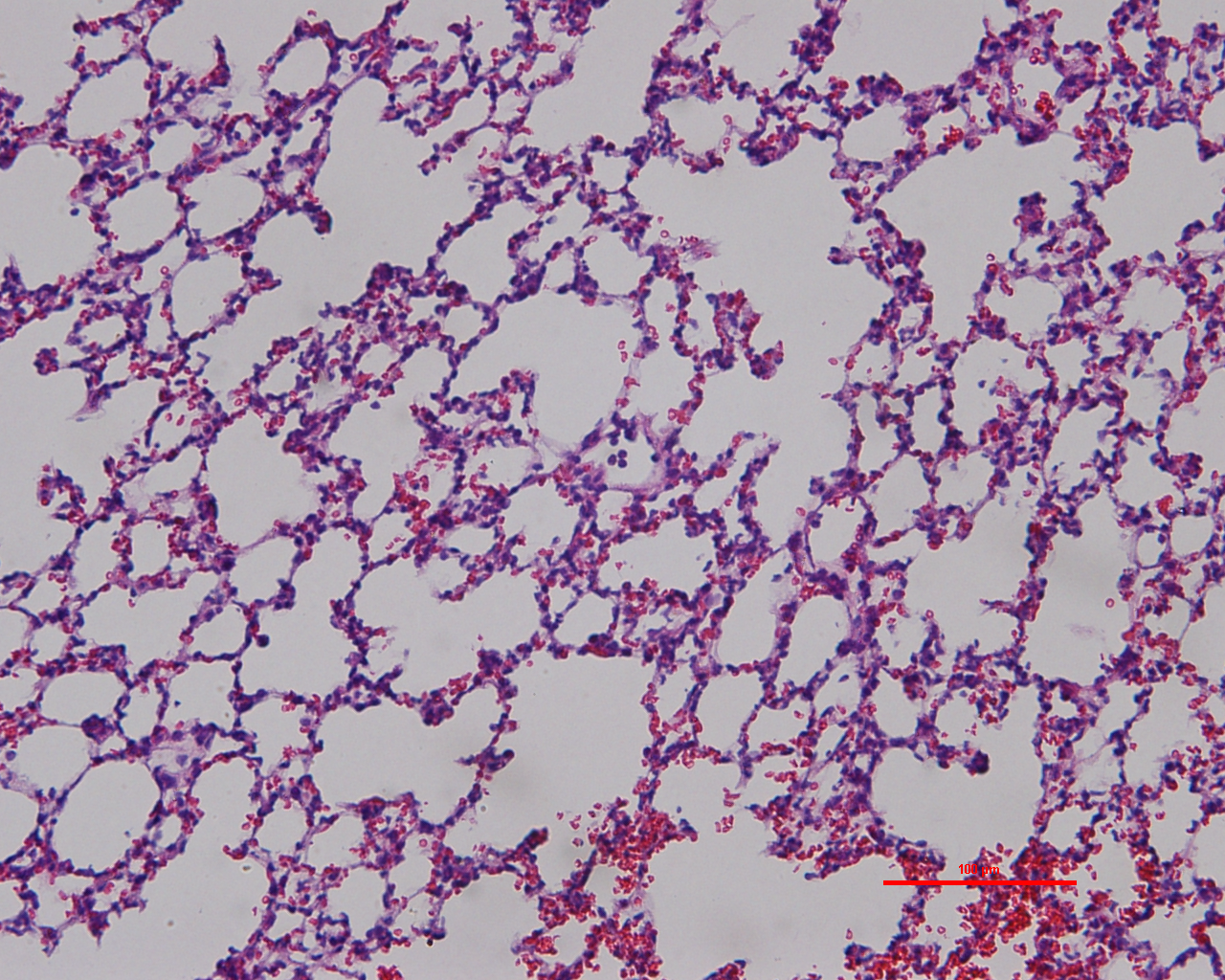

Supplement: S1 File — (ZIP) [file pone.0289818.s001.zip › S1 File. Fig1 Original data/image/1A/NAC.tif]

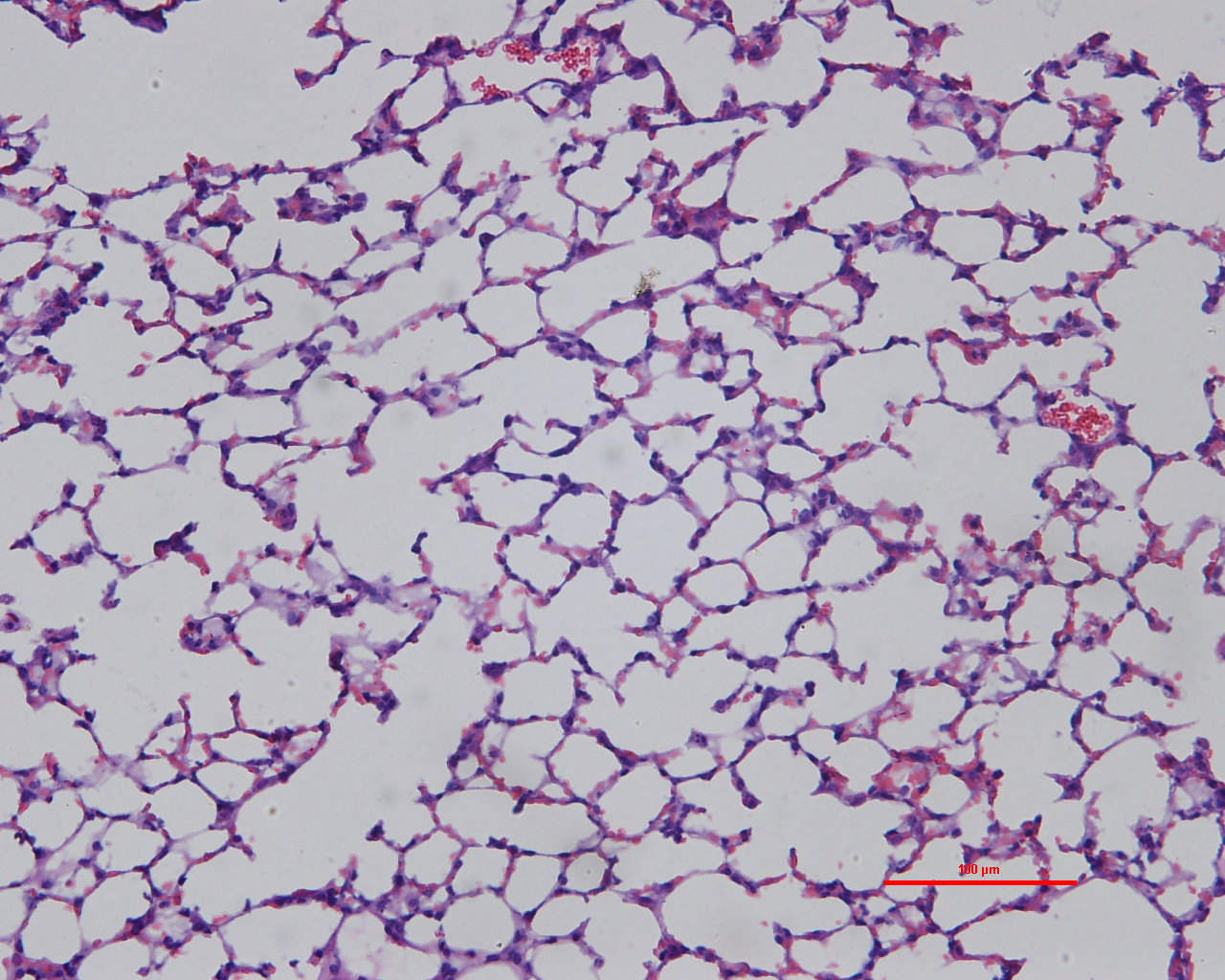

Supplement: S1 File — (ZIP) [file pone.0289818.s001.zip › S1 File. Fig1 Original data/image/1A/NC.tif]

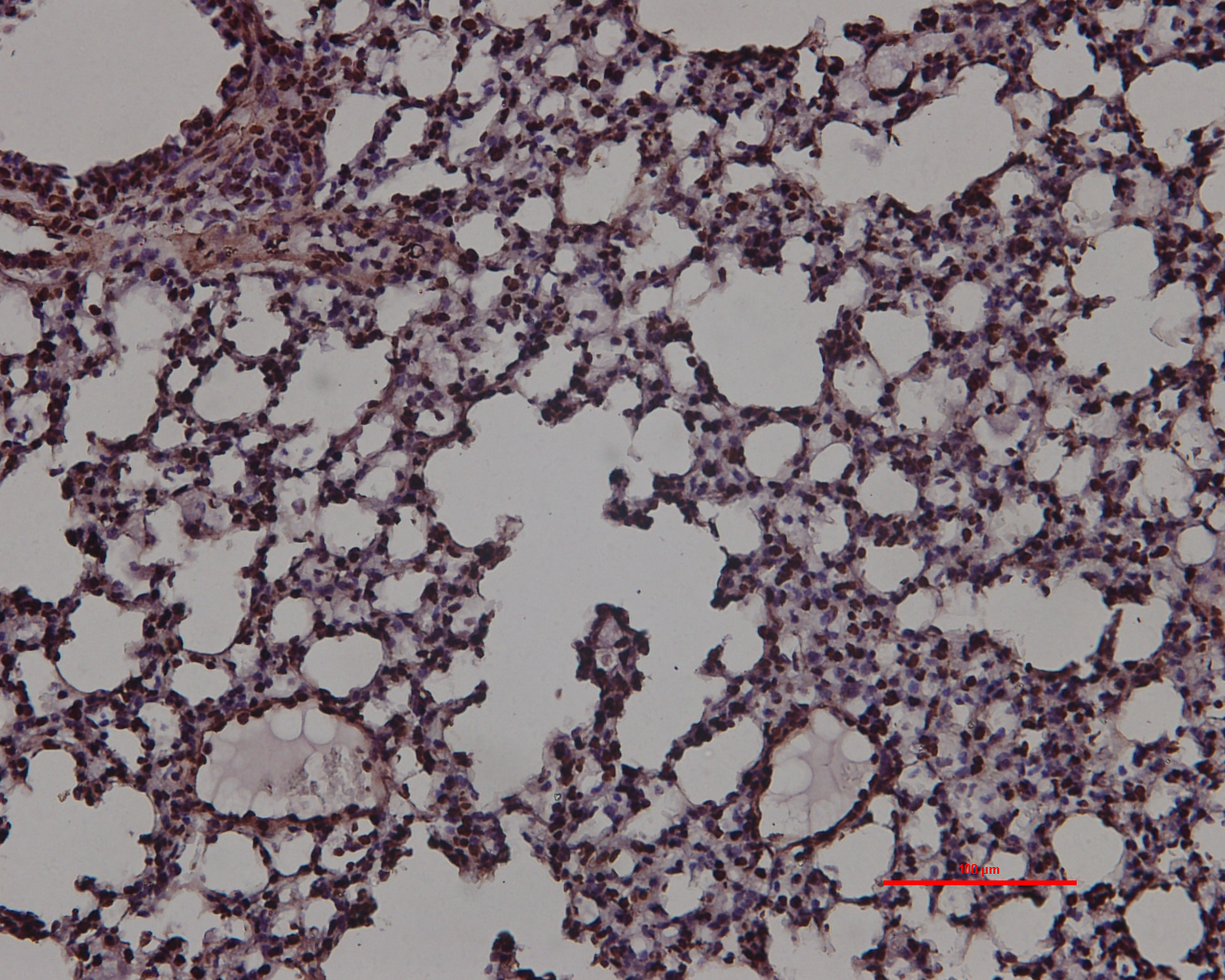

Supplement: S1 File — (ZIP) [file pone.0289818.s001.zip › S1 File. Fig1 Original data/image/1D/ALI.tif]

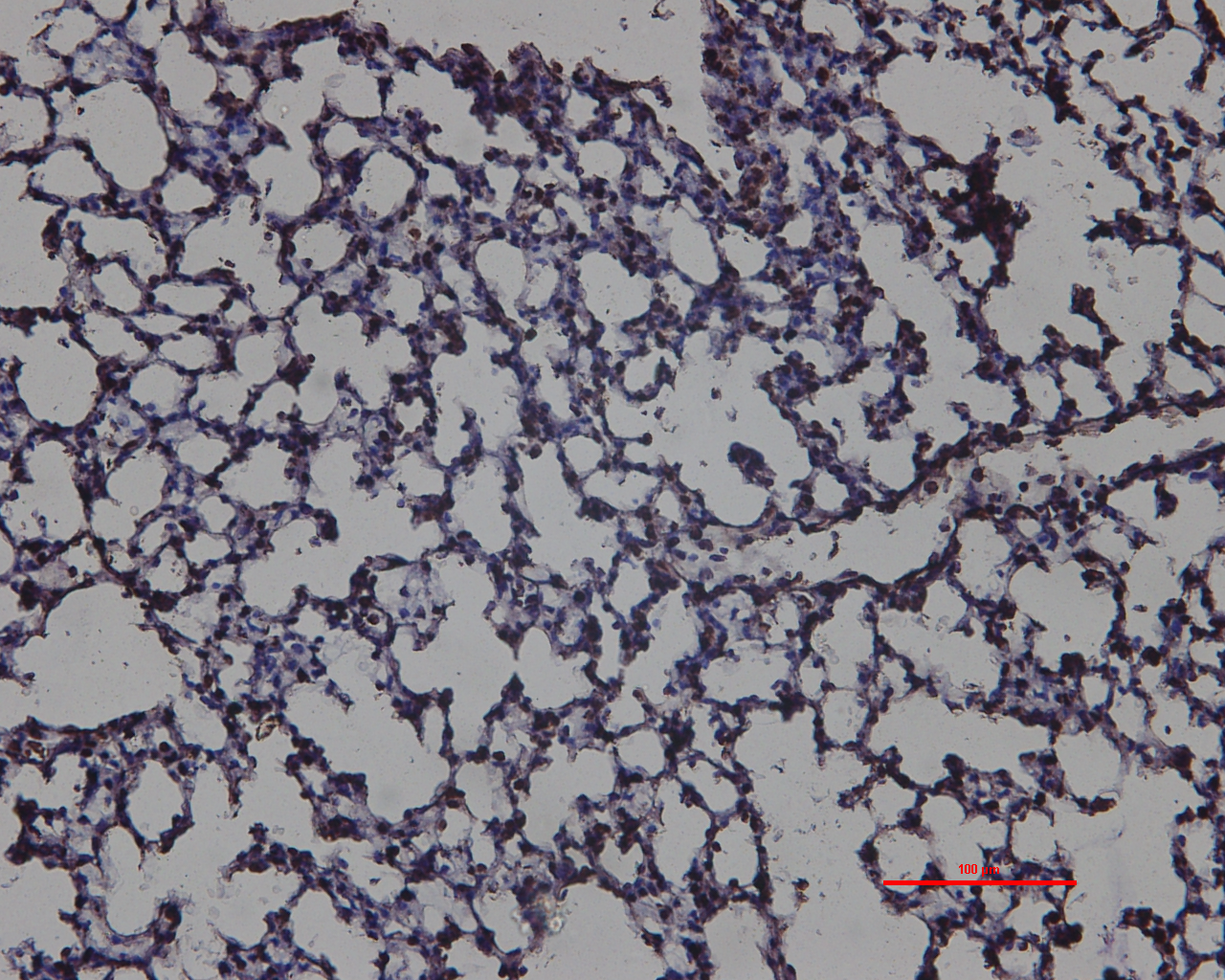

Supplement: S1 File — (ZIP) [file pone.0289818.s001.zip › S1 File. Fig1 Original data/image/1D/Bud+NAC.tif]

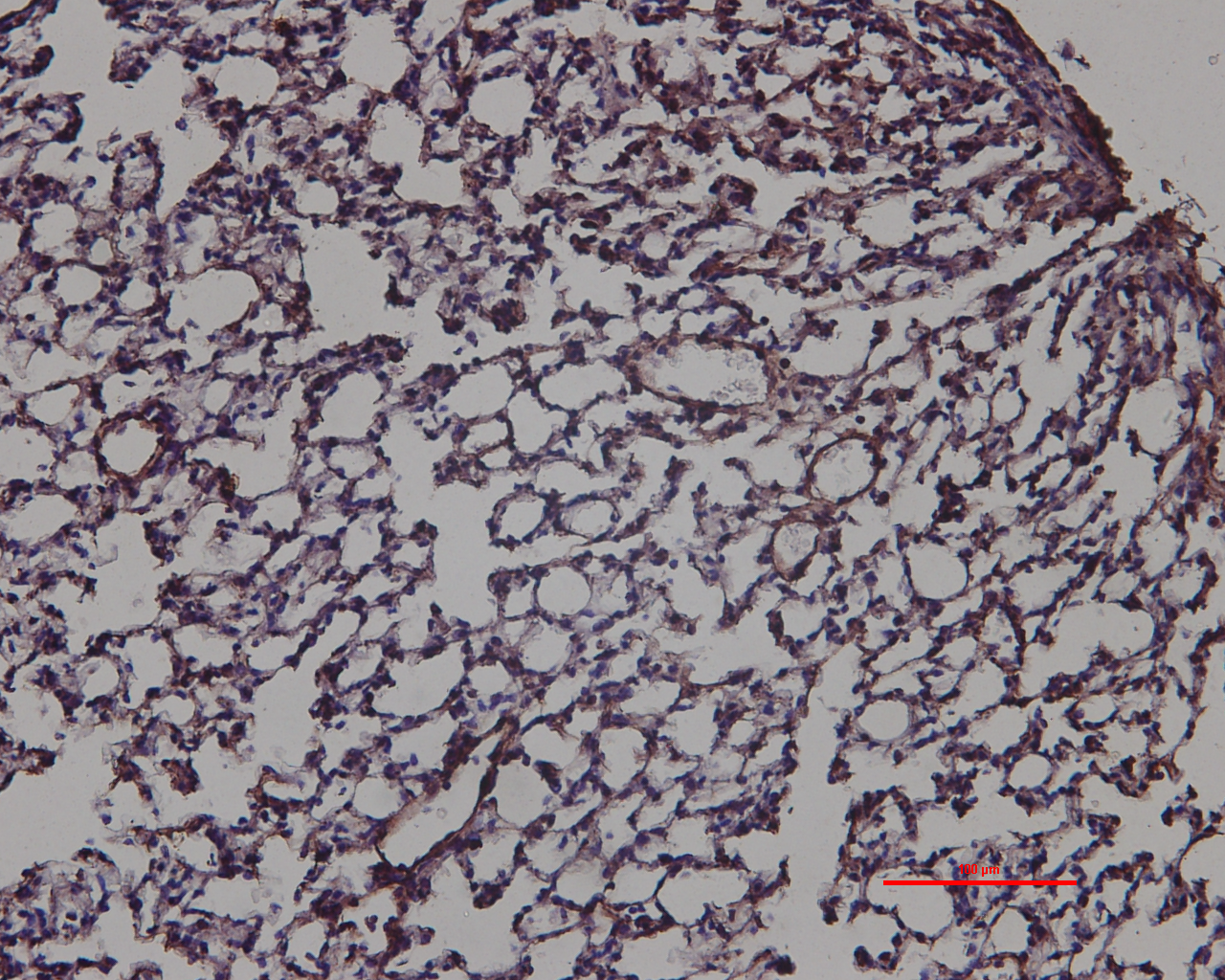

Supplement: S1 File — (ZIP) [file pone.0289818.s001.zip › S1 File. Fig1 Original data/image/1D/Bud.tif]

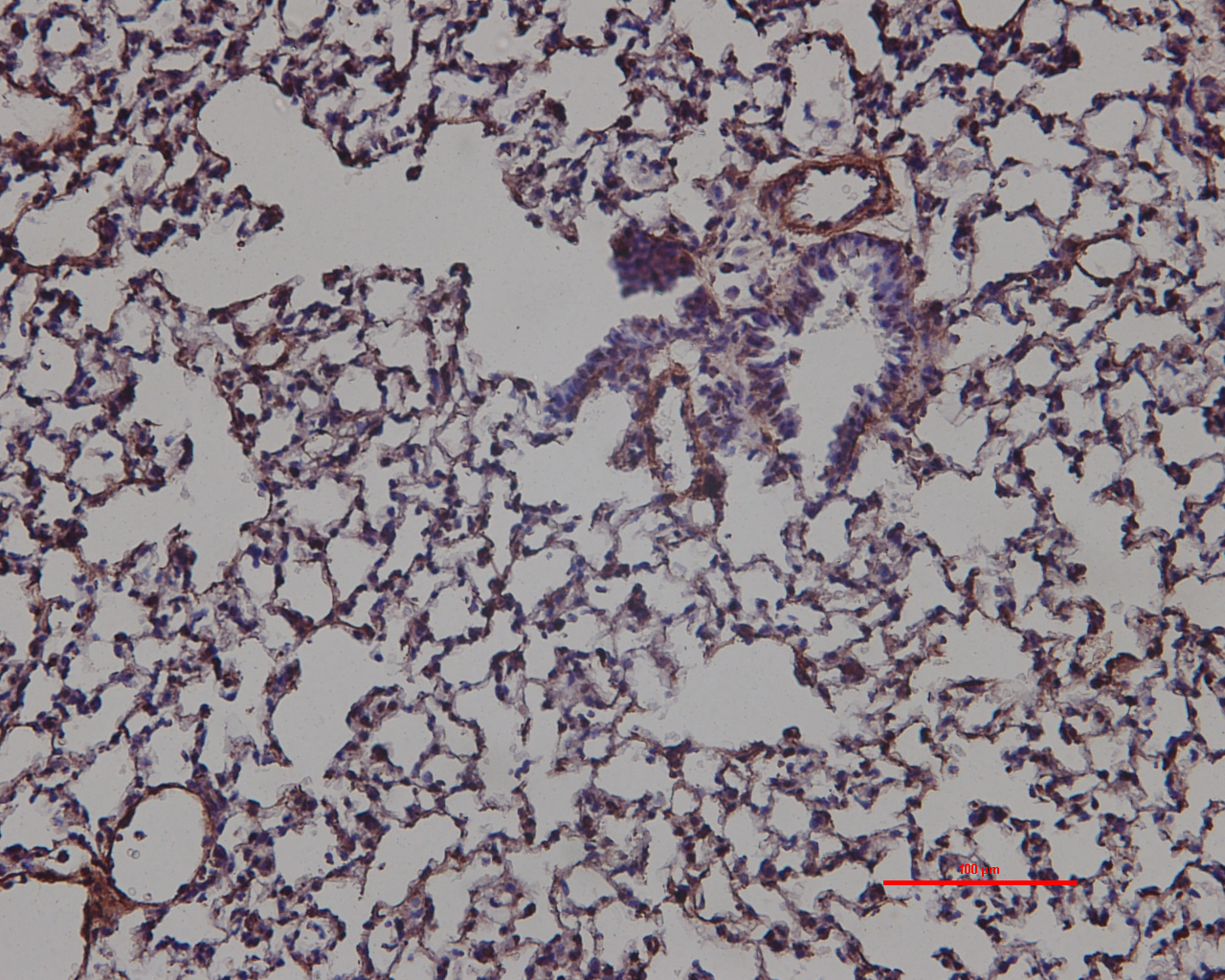

Supplement: S1 File — (ZIP) [file pone.0289818.s001.zip › S1 File. Fig1 Original data/image/1D/NAC.tif]

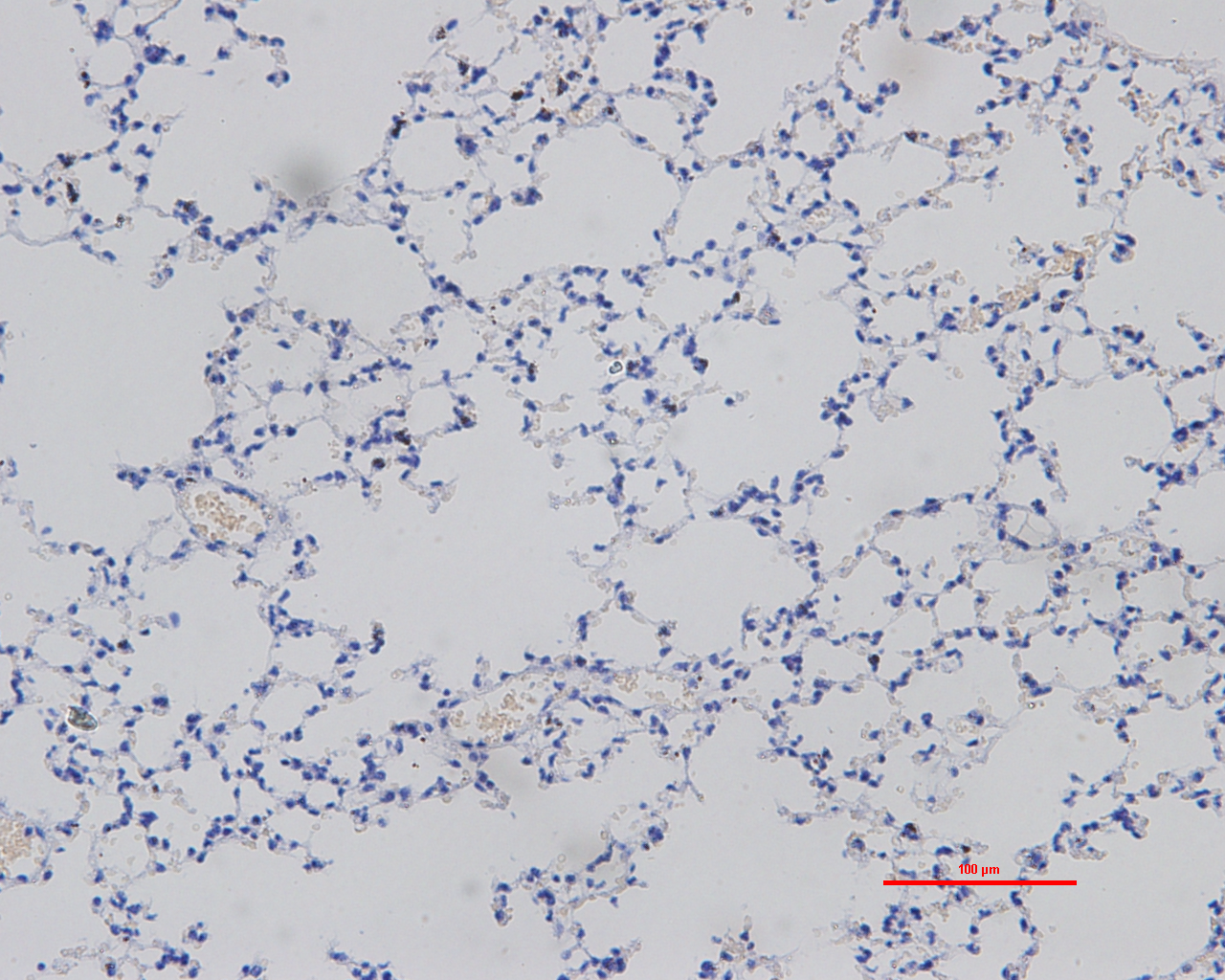

Supplement: S1 File — (ZIP) [file pone.0289818.s001.zip › S1 File. Fig1 Original data/image/1D/NC.tif]

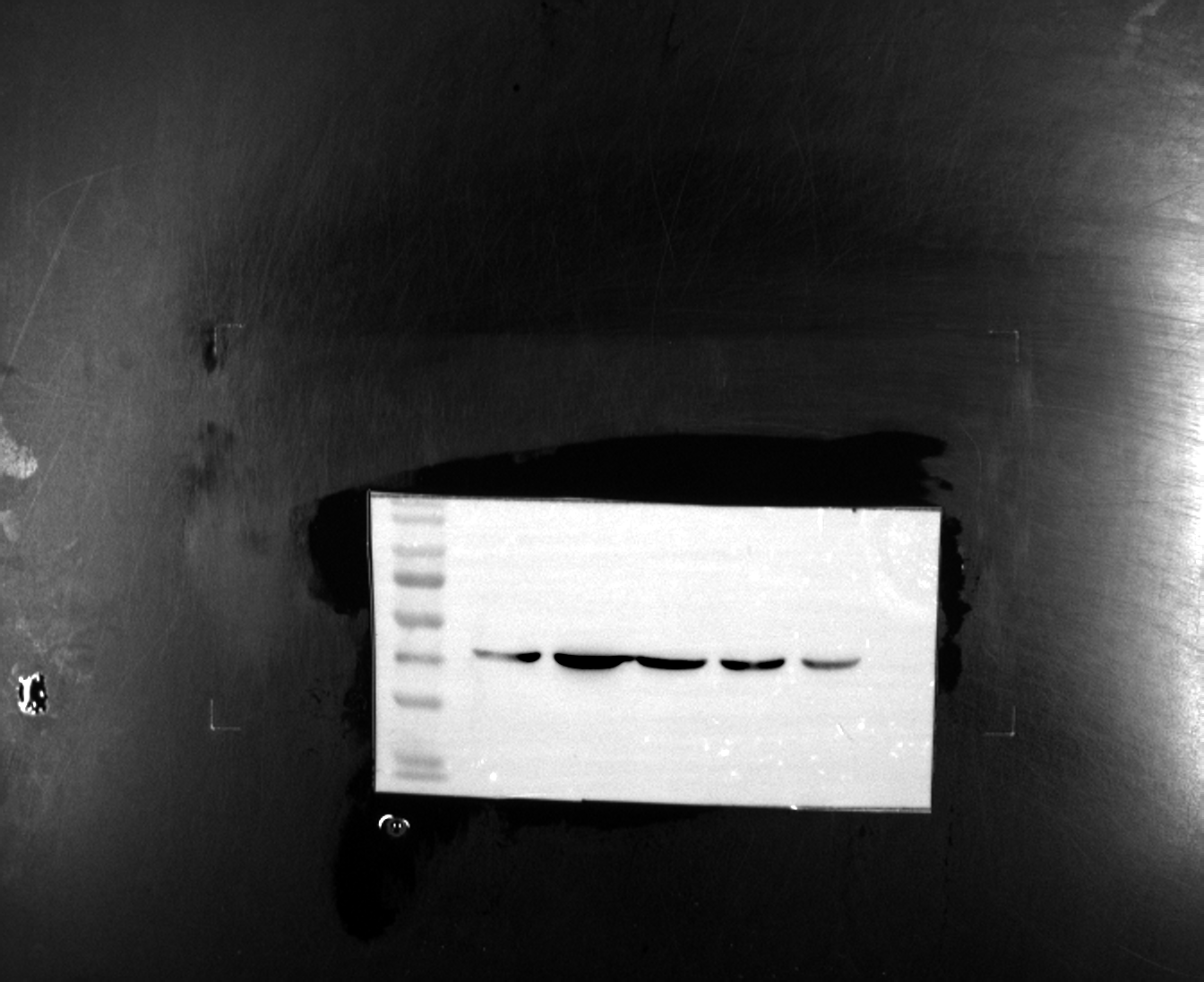

Supplement: S1 File — (ZIP) [file pone.0289818.s001.zip › S1 File. Fig1 Original data/image/1E/1.Caspase1.tif]

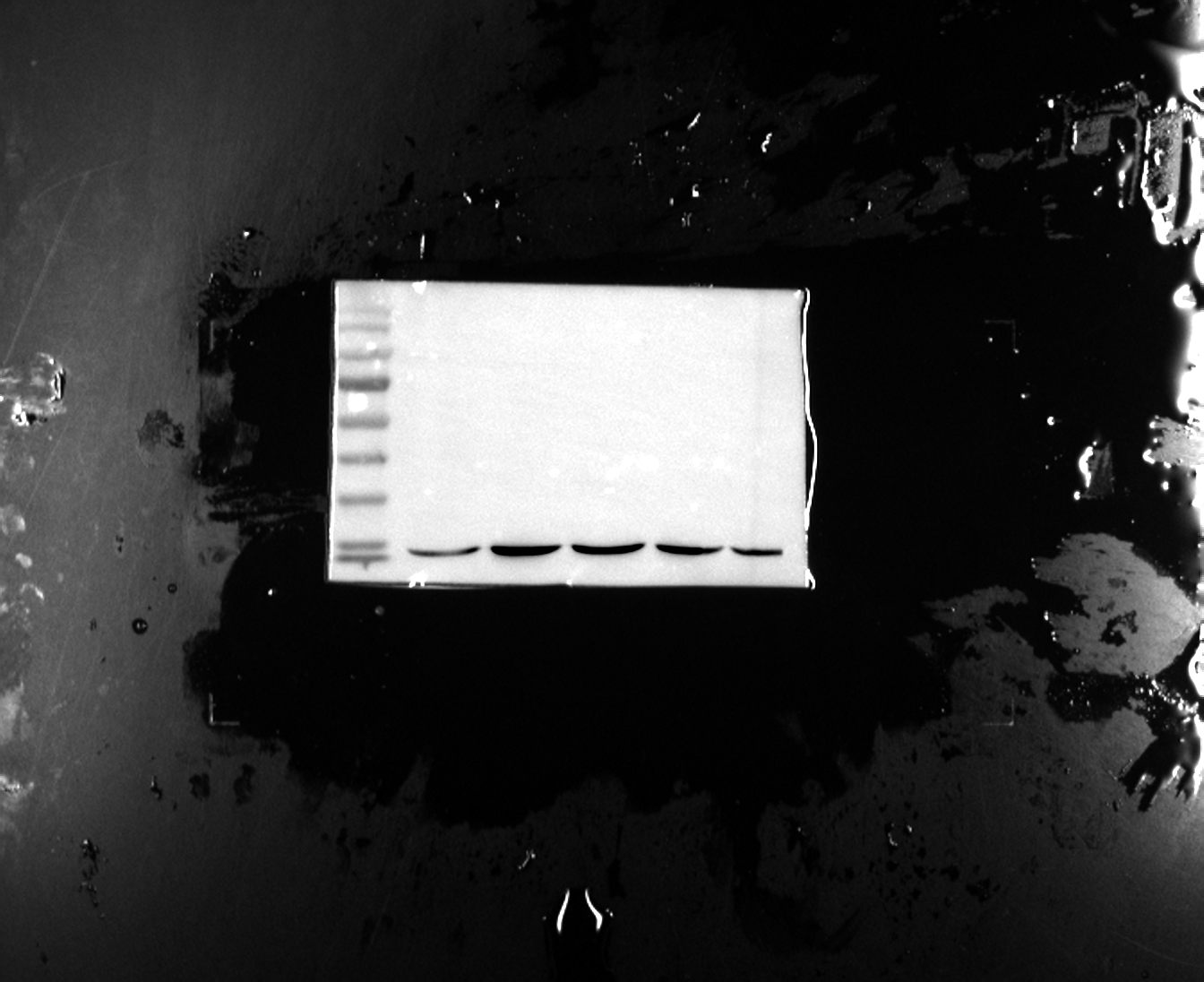

Supplement: S1 File — (ZIP) [file pone.0289818.s001.zip › S1 File. Fig1 Original data/image/1E/2.ASC.tif]

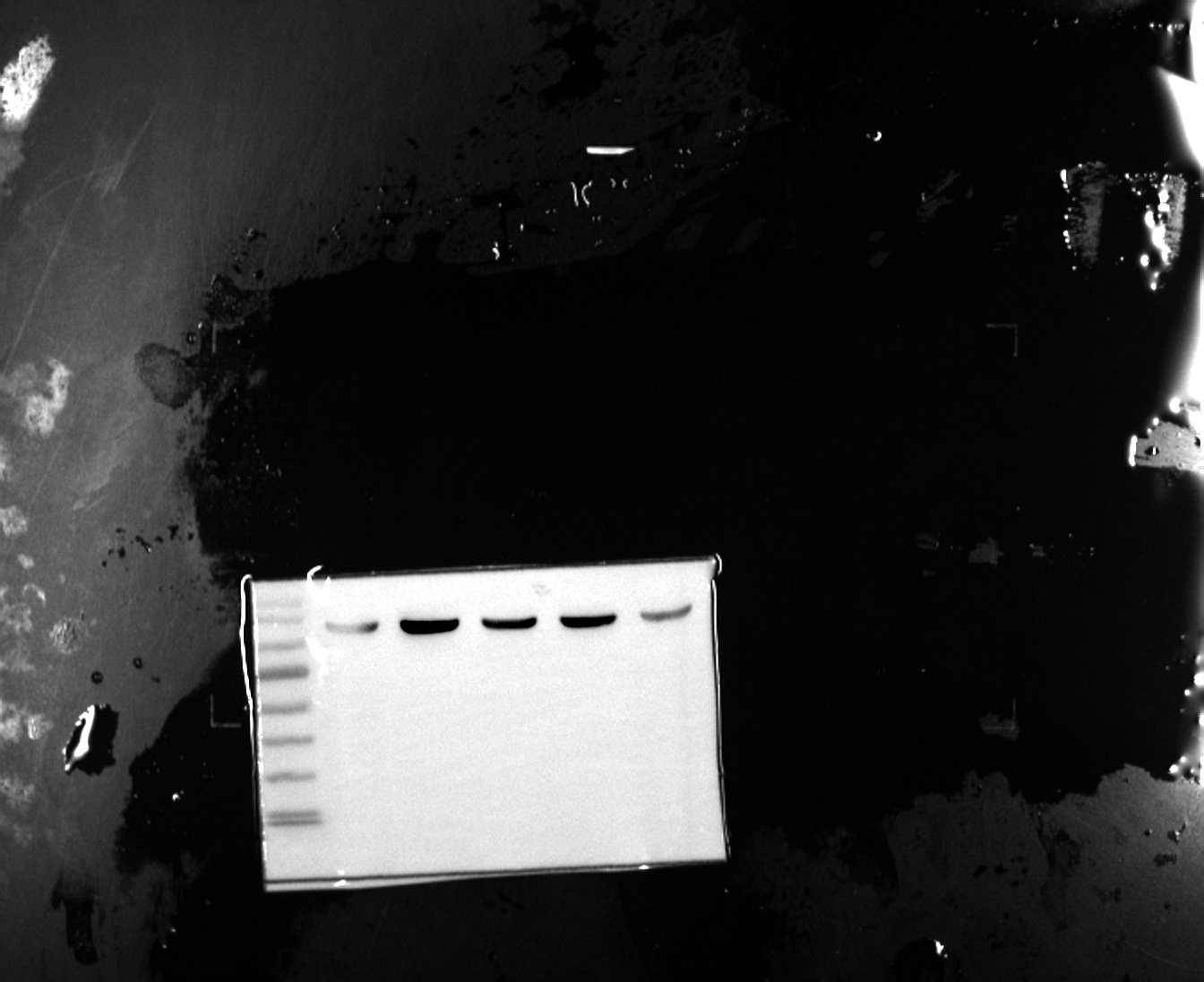

Supplement: S1 File — (ZIP) [file pone.0289818.s001.zip › S1 File. Fig1 Original data/image/1E/3.NLRP3.tif]

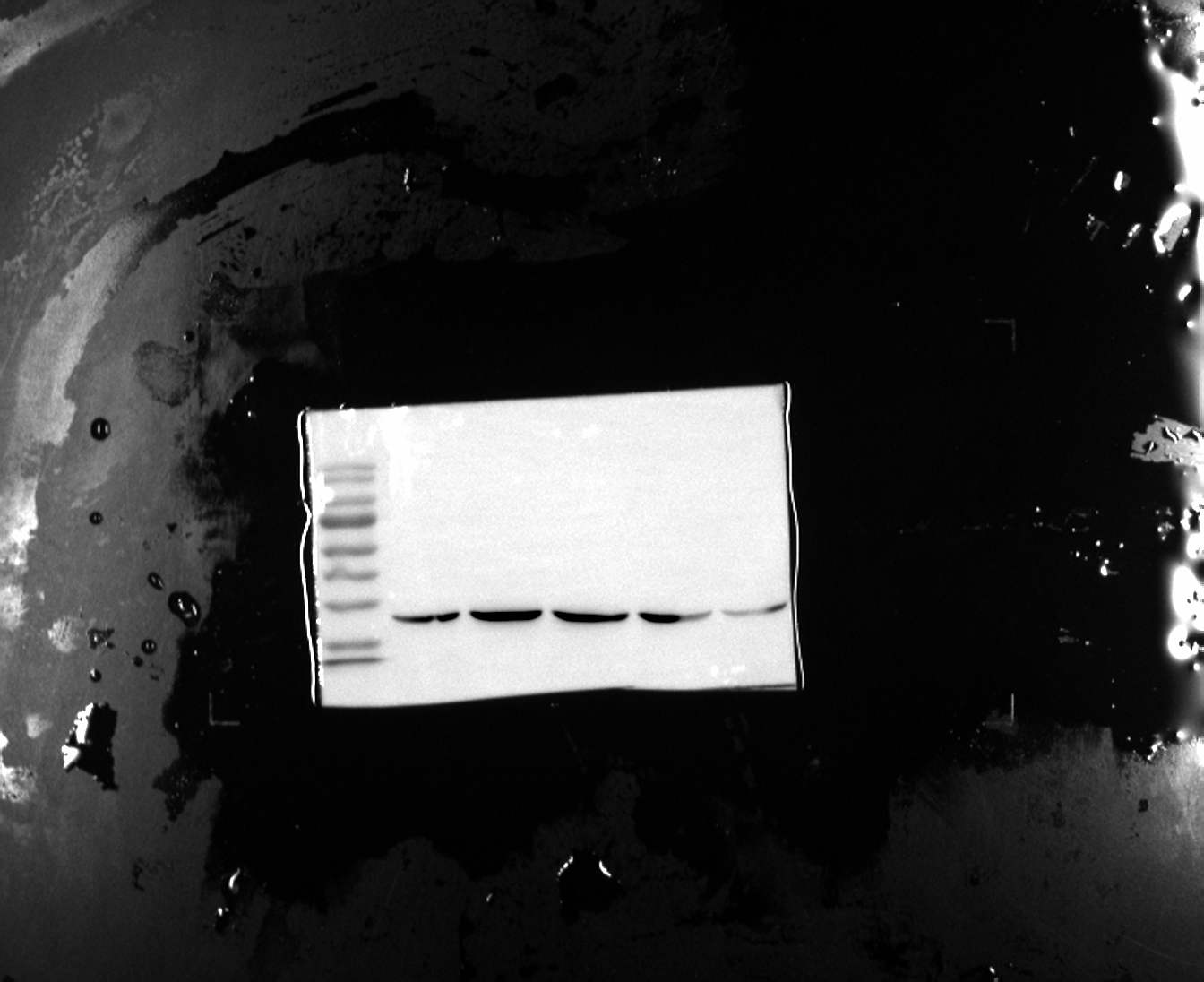

Supplement: S1 File — (ZIP) [file pone.0289818.s001.zip › S1 File. Fig1 Original data/image/1E/4.IL-1β.tif]

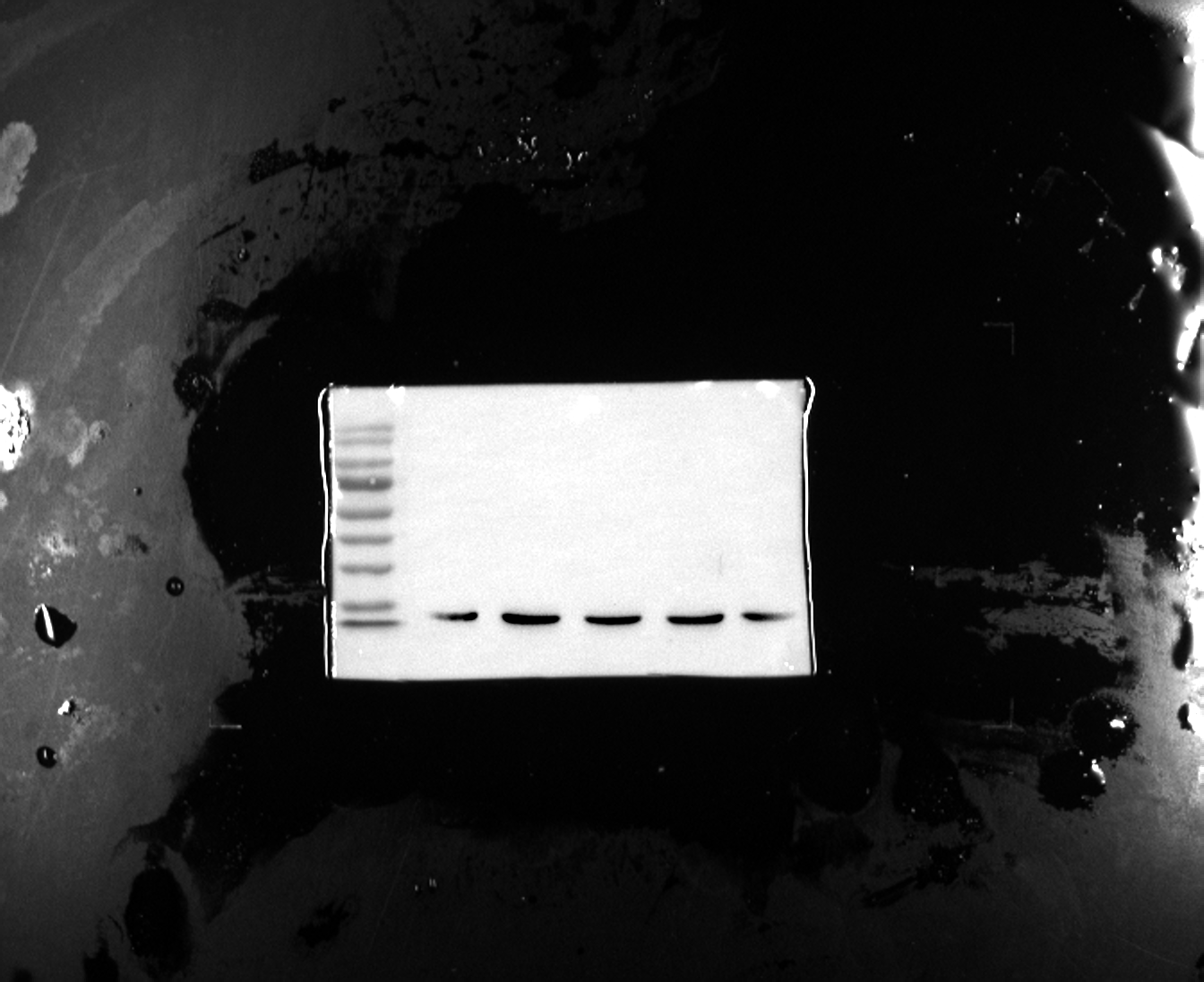

Supplement: S1 File — (ZIP) [file pone.0289818.s001.zip › S1 File. Fig1 Original data/image/1E/5.IL-18.tif]

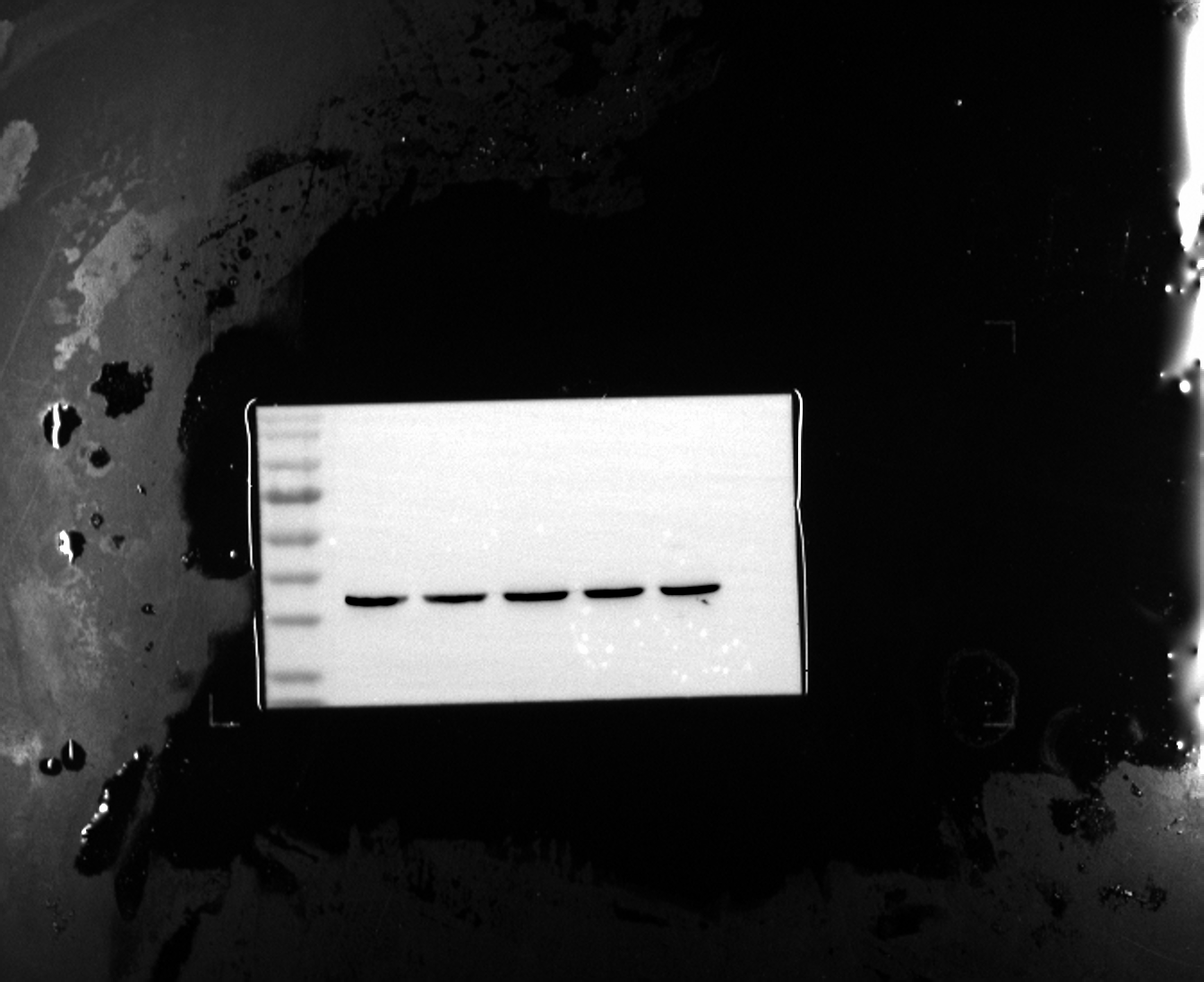

Supplement: S1 File — (ZIP) [file pone.0289818.s001.zip › S1 File. Fig1 Original data/image/1E/6.GAPDH.tif]

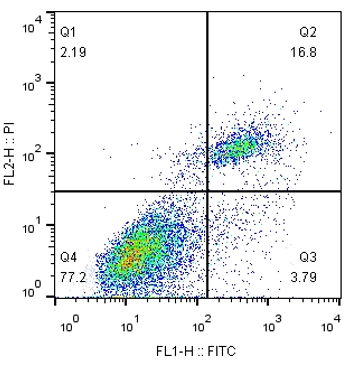

Supplement: S2 File — (ZIP) [file pone.0289818.s002.zip › S2 File. Fig2 Original data/image/2B/LPS/1 (1).jpg]

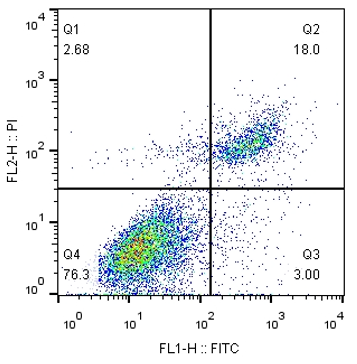

Supplement: S2 File — (ZIP) [file pone.0289818.s002.zip › S2 File. Fig2 Original data/image/2B/LPS/1 (2).jpg]

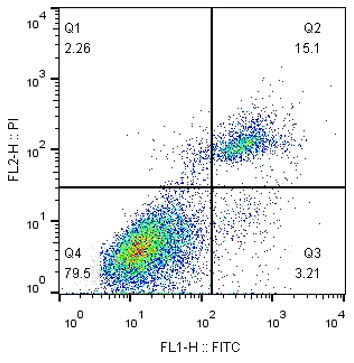

Supplement: S2 File — (ZIP) [file pone.0289818.s002.zip › S2 File. Fig2 Original data/image/2B/LPS/1 (3).jpg]

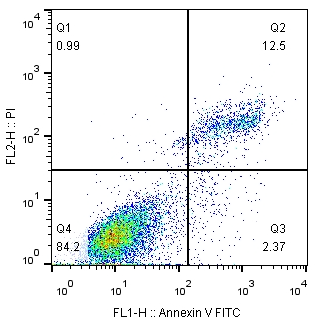

Supplement: S2 File — (ZIP) [file pone.0289818.s002.zip › S2 File. Fig2 Original data/image/2B/LPS+Bud/1 (1).jpg]

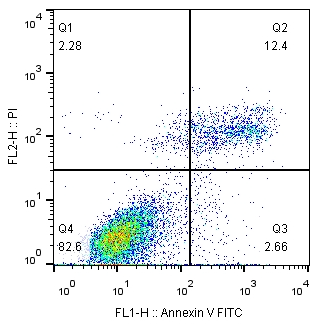

Supplement: S2 File — (ZIP) [file pone.0289818.s002.zip › S2 File. Fig2 Original data/image/2B/LPS+Bud/1 (2).jpg]

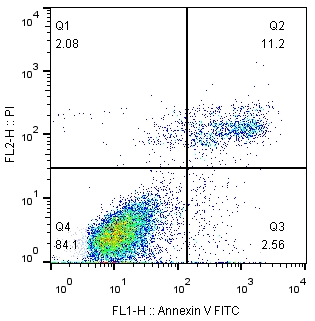

Supplement: S2 File — (ZIP) [file pone.0289818.s002.zip › S2 File. Fig2 Original data/image/2B/LPS+Bud/1 (3).jpg]

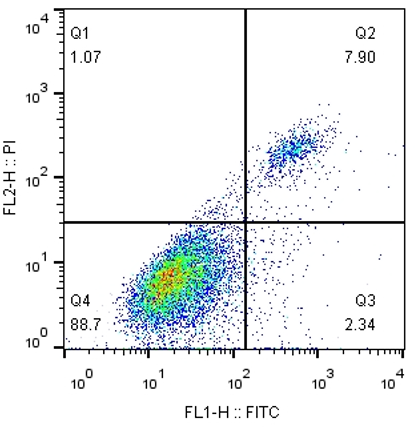

Supplement: S2 File — (ZIP) [file pone.0289818.s002.zip › S2 File. Fig2 Original data/image/2B/LPS+Bud+NAC/1 (1).jpg]

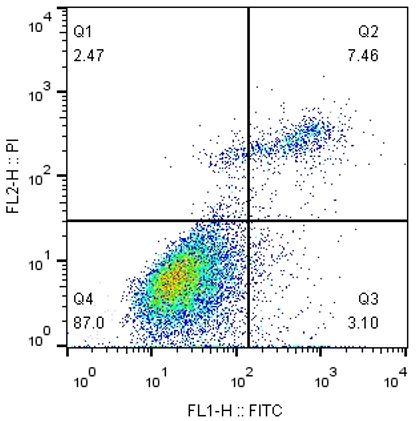

Supplement: S2 File — (ZIP) [file pone.0289818.s002.zip › S2 File. Fig2 Original data/image/2B/LPS+Bud+NAC/1 (2).jpg]

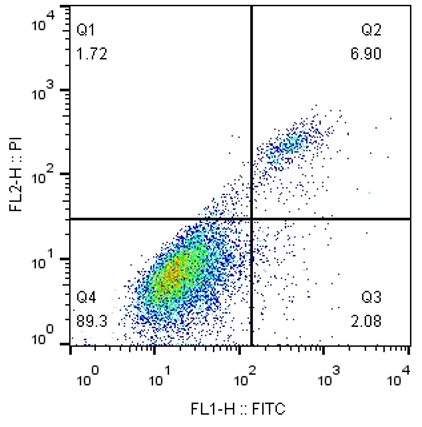

Supplement: S2 File — (ZIP) [file pone.0289818.s002.zip › S2 File. Fig2 Original data/image/2B/LPS+Bud+NAC/1 (3).jpg]

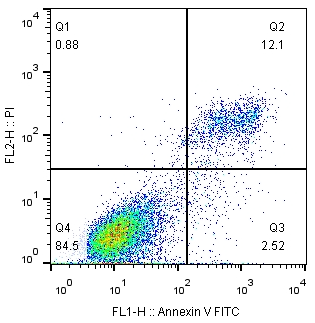

Supplement: S2 File — (ZIP) [file pone.0289818.s002.zip › S2 File. Fig2 Original data/image/2B/LPS+NAC/1 (1).jpg]

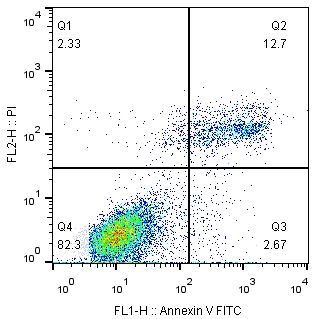

Supplement: S2 File — (ZIP) [file pone.0289818.s002.zip › S2 File. Fig2 Original data/image/2B/LPS+NAC/1 (2).jpg]

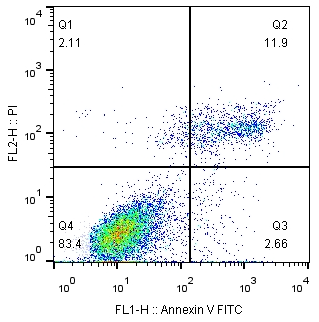

Supplement: S2 File — (ZIP) [file pone.0289818.s002.zip › S2 File. Fig2 Original data/image/2B/LPS+NAC/1 (3).jpg]

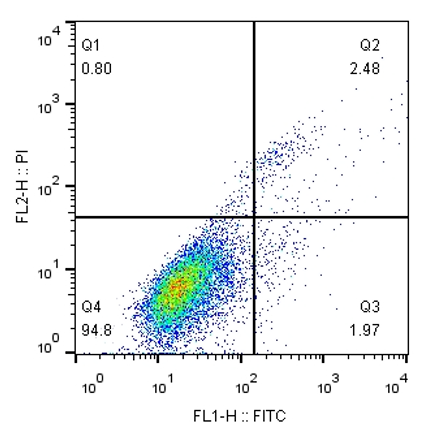

Supplement: S2 File — (ZIP) [file pone.0289818.s002.zip › S2 File. Fig2 Original data/image/2B/NC/1 (1).jpg]

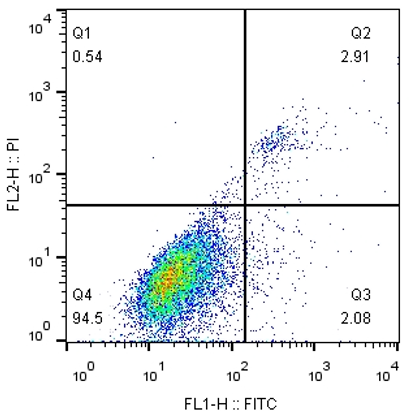

Supplement: S2 File — (ZIP) [file pone.0289818.s002.zip › S2 File. Fig2 Original data/image/2B/NC/1 (2).jpg]

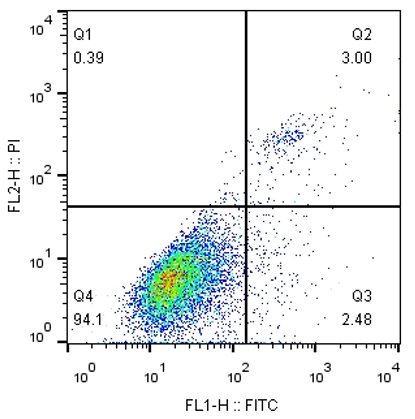

Supplement: S2 File — (ZIP) [file pone.0289818.s002.zip › S2 File. Fig2 Original data/image/2B/NC/1 (3).jpg]

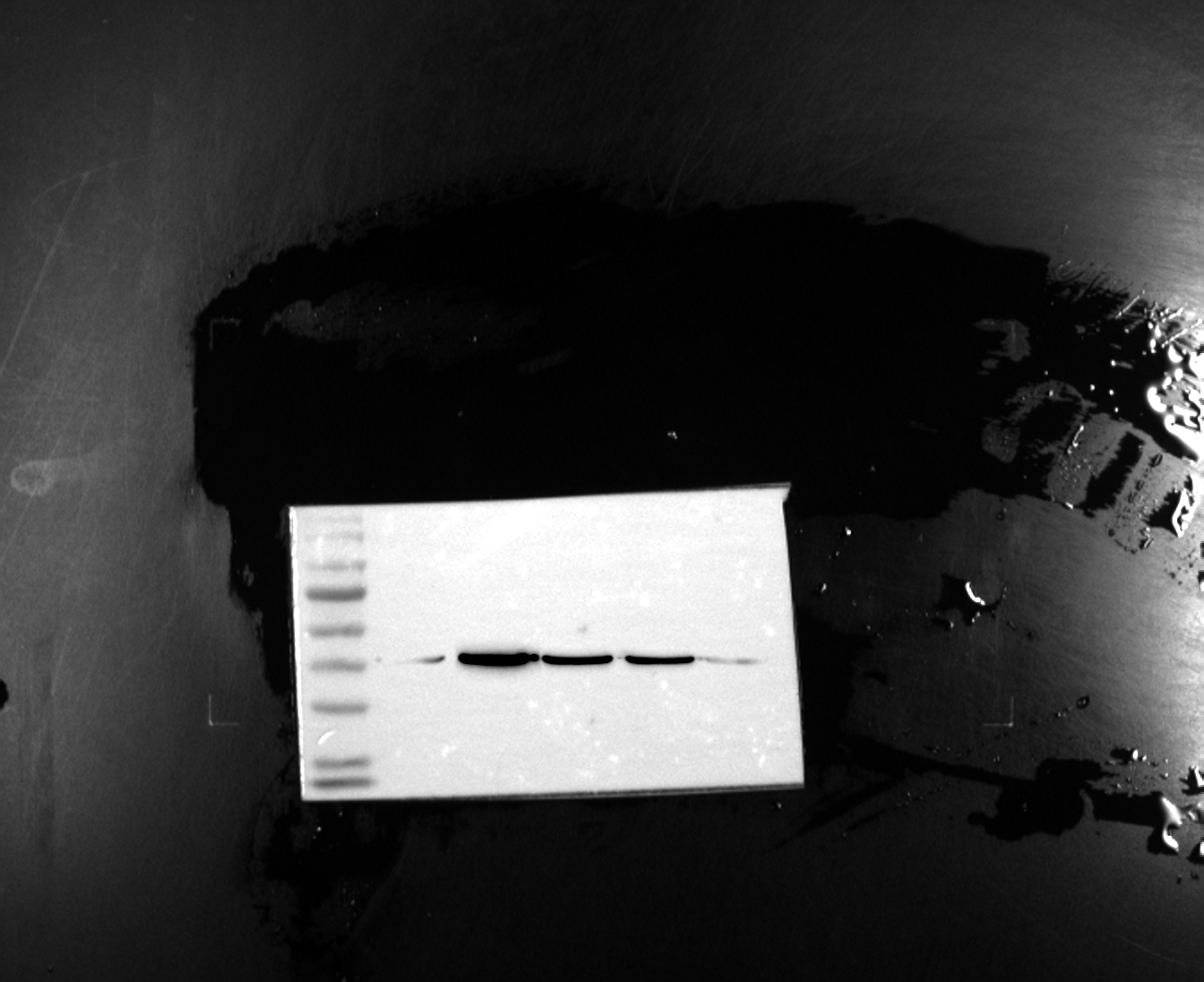

Supplement: S2 File — (ZIP) [file pone.0289818.s002.zip › S2 File. Fig2 Original data/image/2C/1-Caspase1.tif]

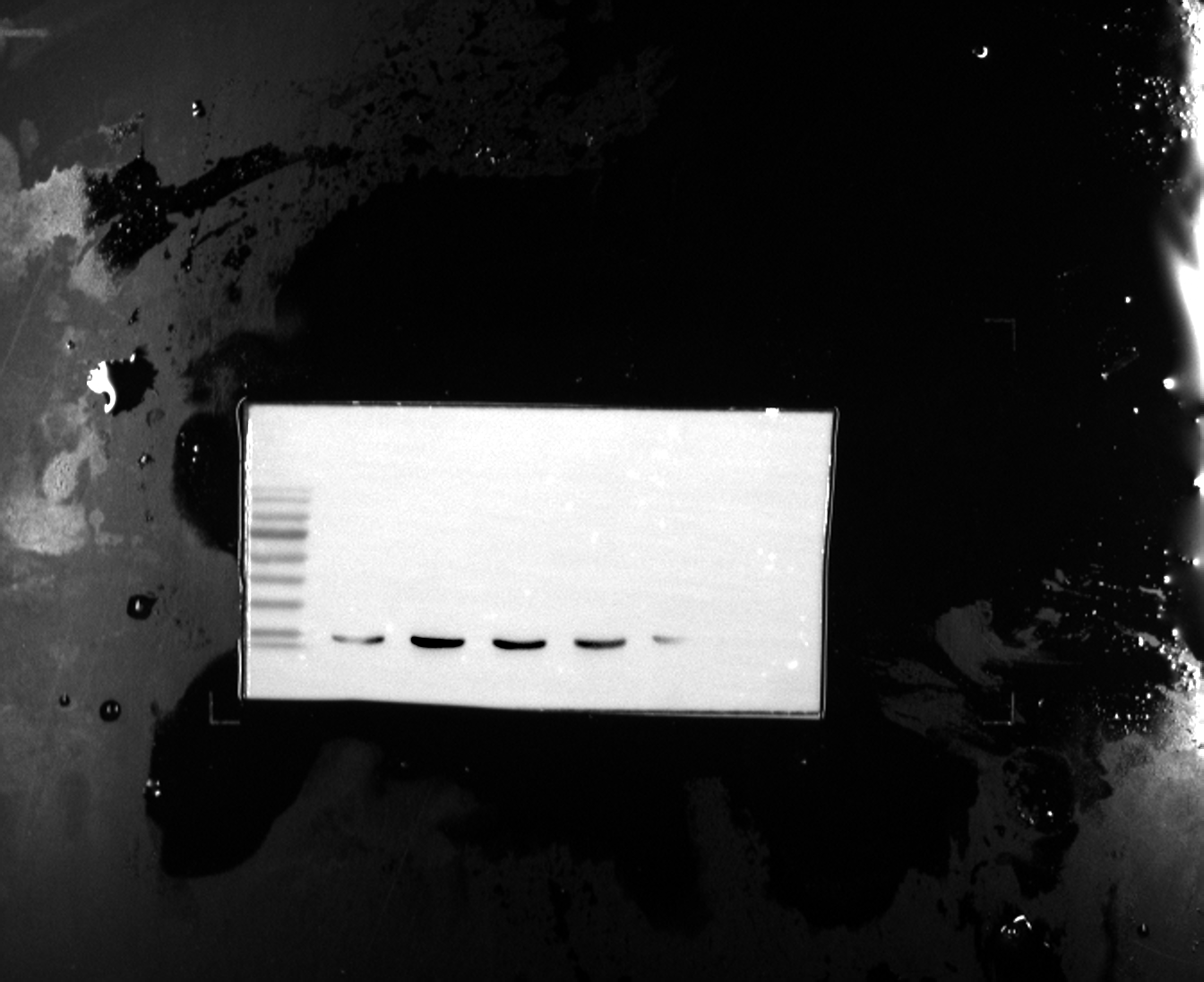

Supplement: S2 File — (ZIP) [file pone.0289818.s002.zip › S2 File. Fig2 Original data/image/2C/2-ASC.tif]

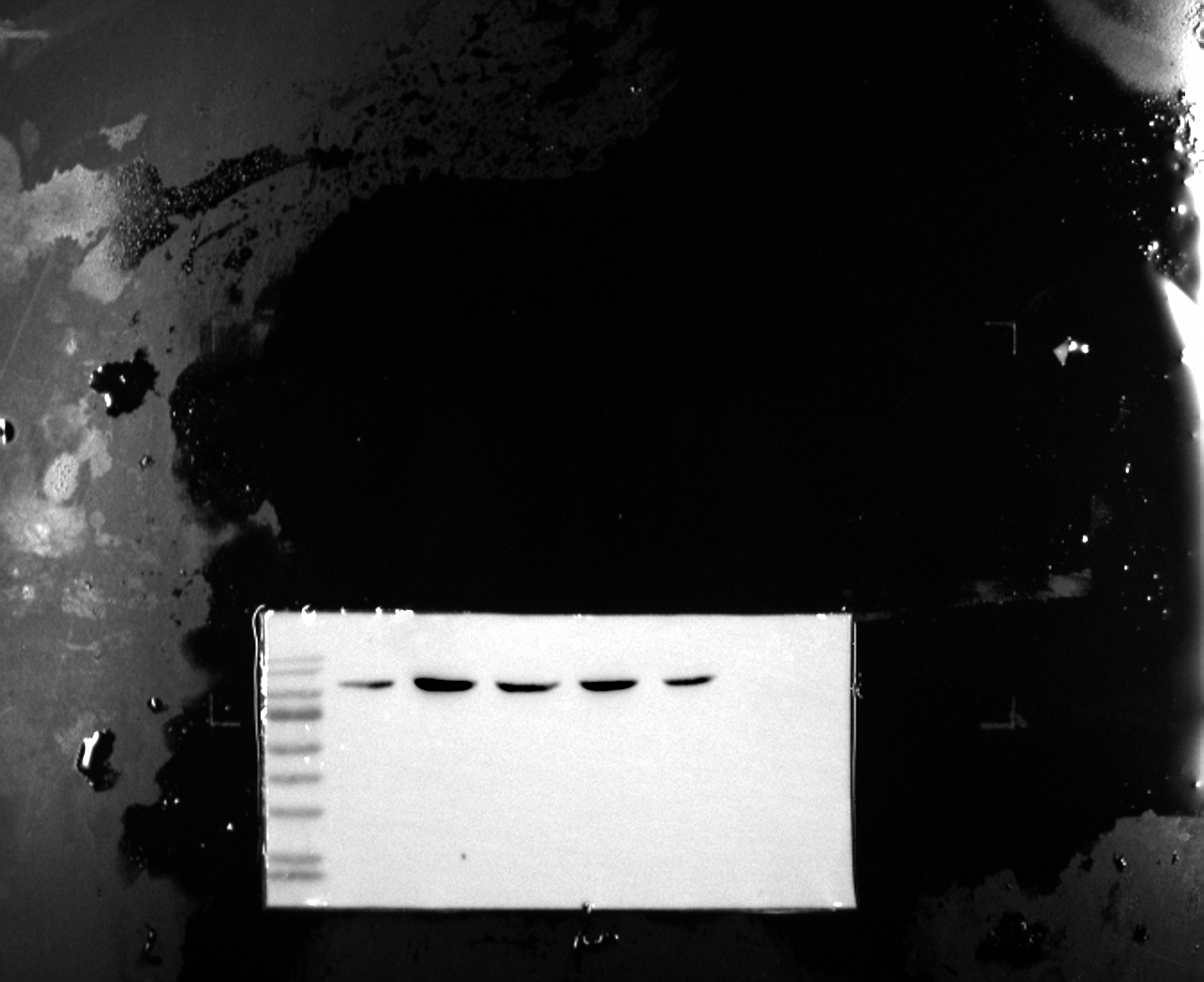

Supplement: S2 File — (ZIP) [file pone.0289818.s002.zip › S2 File. Fig2 Original data/image/2C/3-NLRP3.tif]

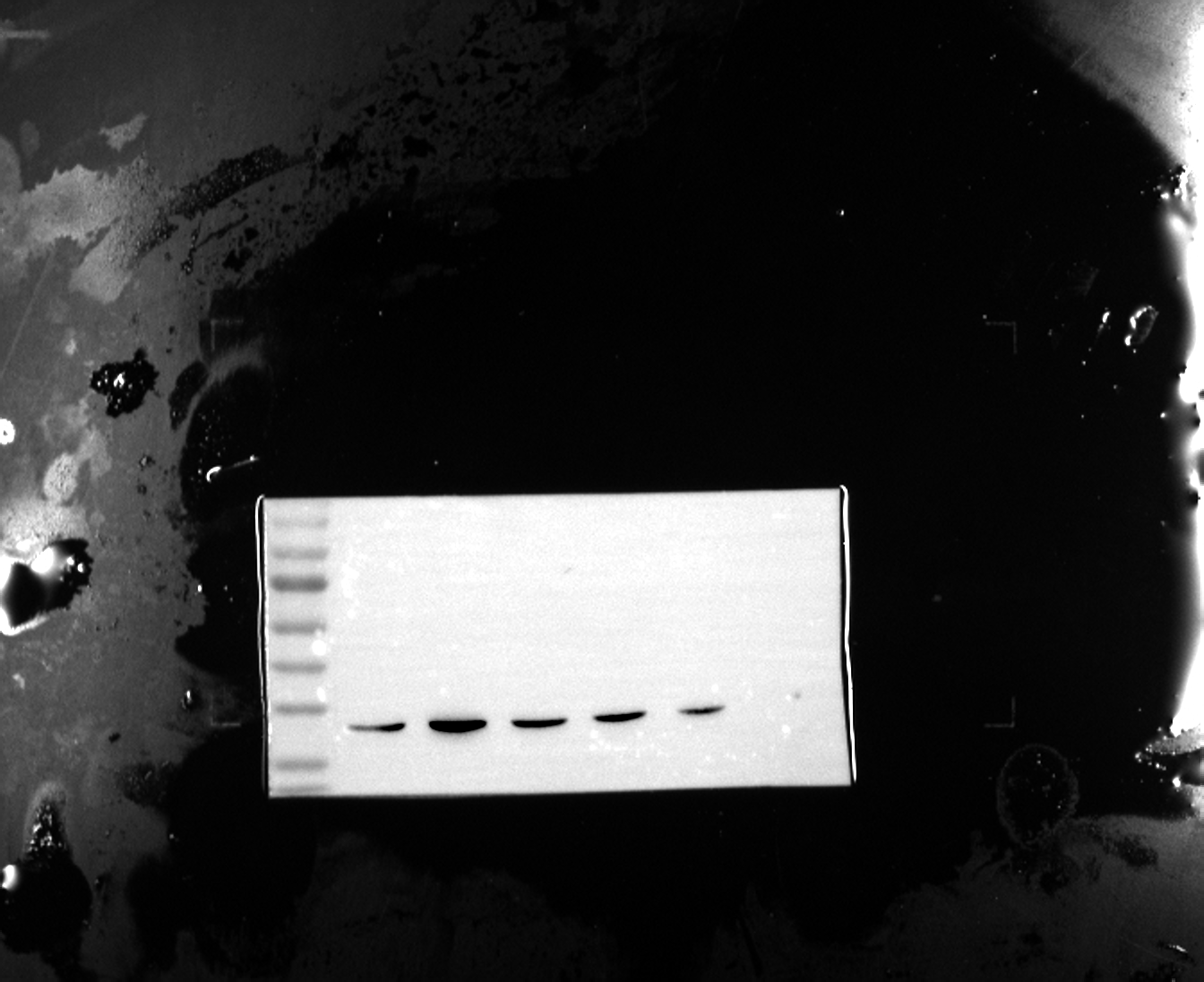

Supplement: S2 File — (ZIP) [file pone.0289818.s002.zip › S2 File. Fig2 Original data/image/2C/4-IL-1β.tif]

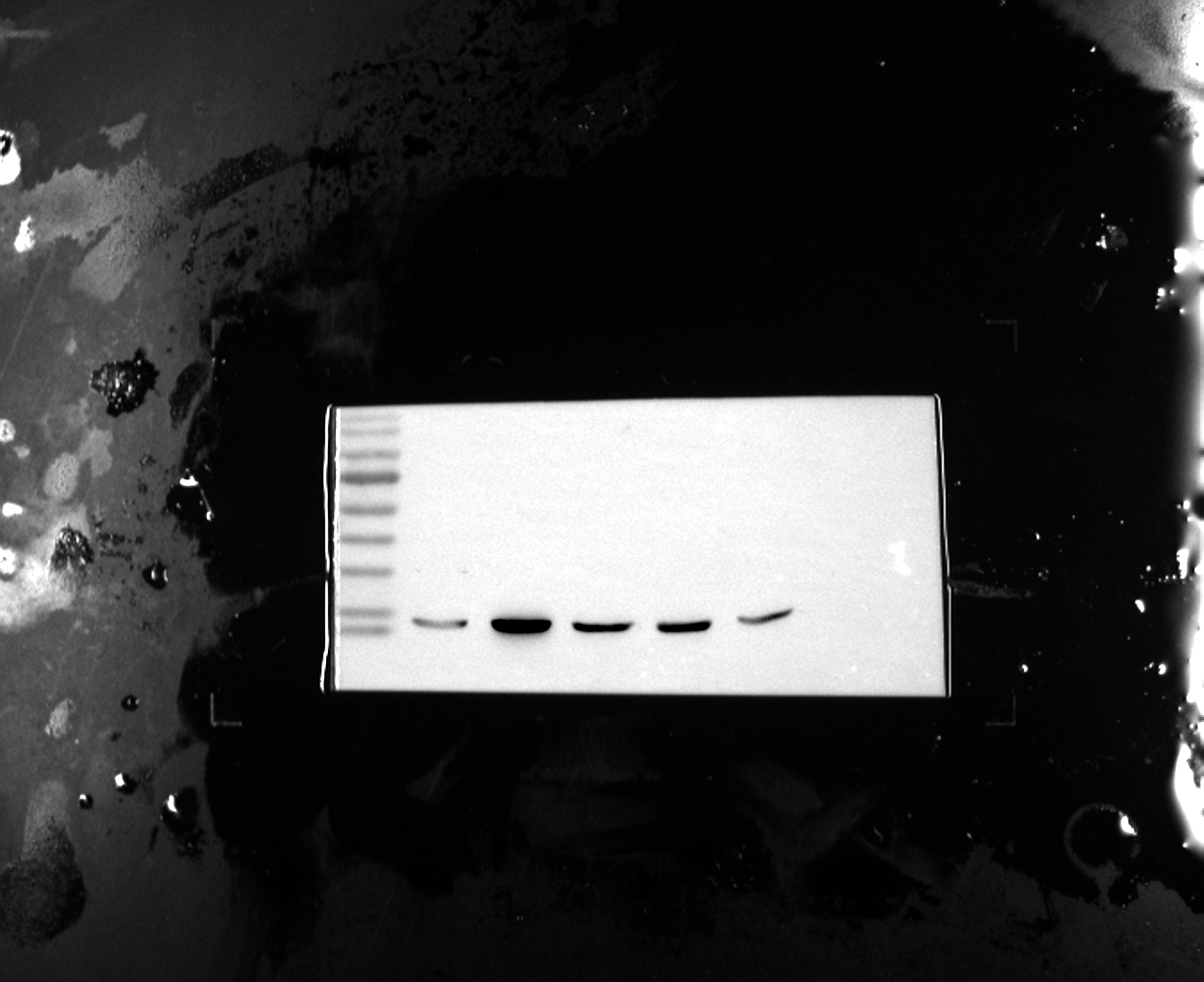

Supplement: S2 File — (ZIP) [file pone.0289818.s002.zip › S2 File. Fig2 Original data/image/2C/5-IL-18.tif]

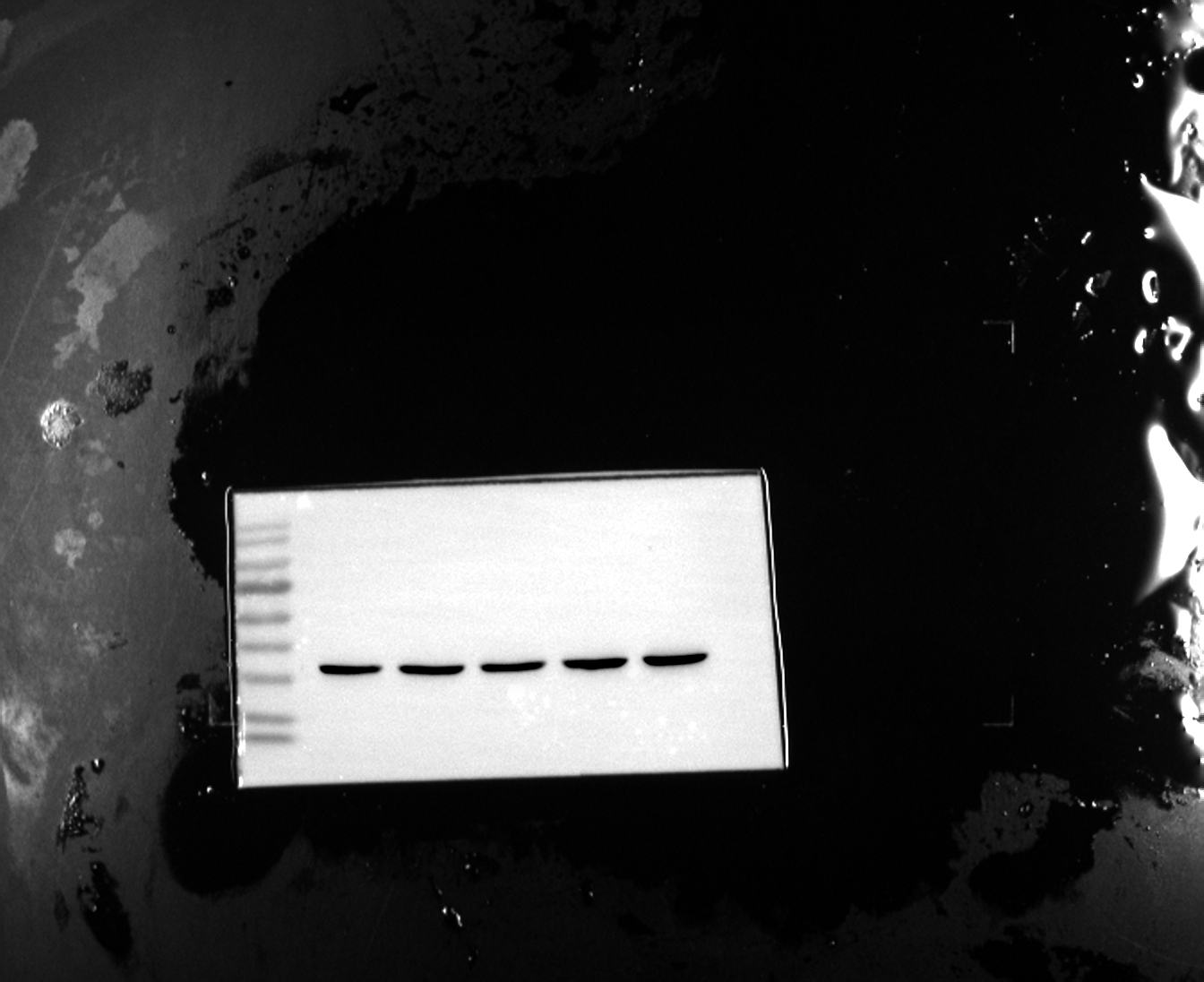

Supplement: S2 File — (ZIP) [file pone.0289818.s002.zip › S2 File. Fig2 Original data/image/2C/6-GAPDH.tif]

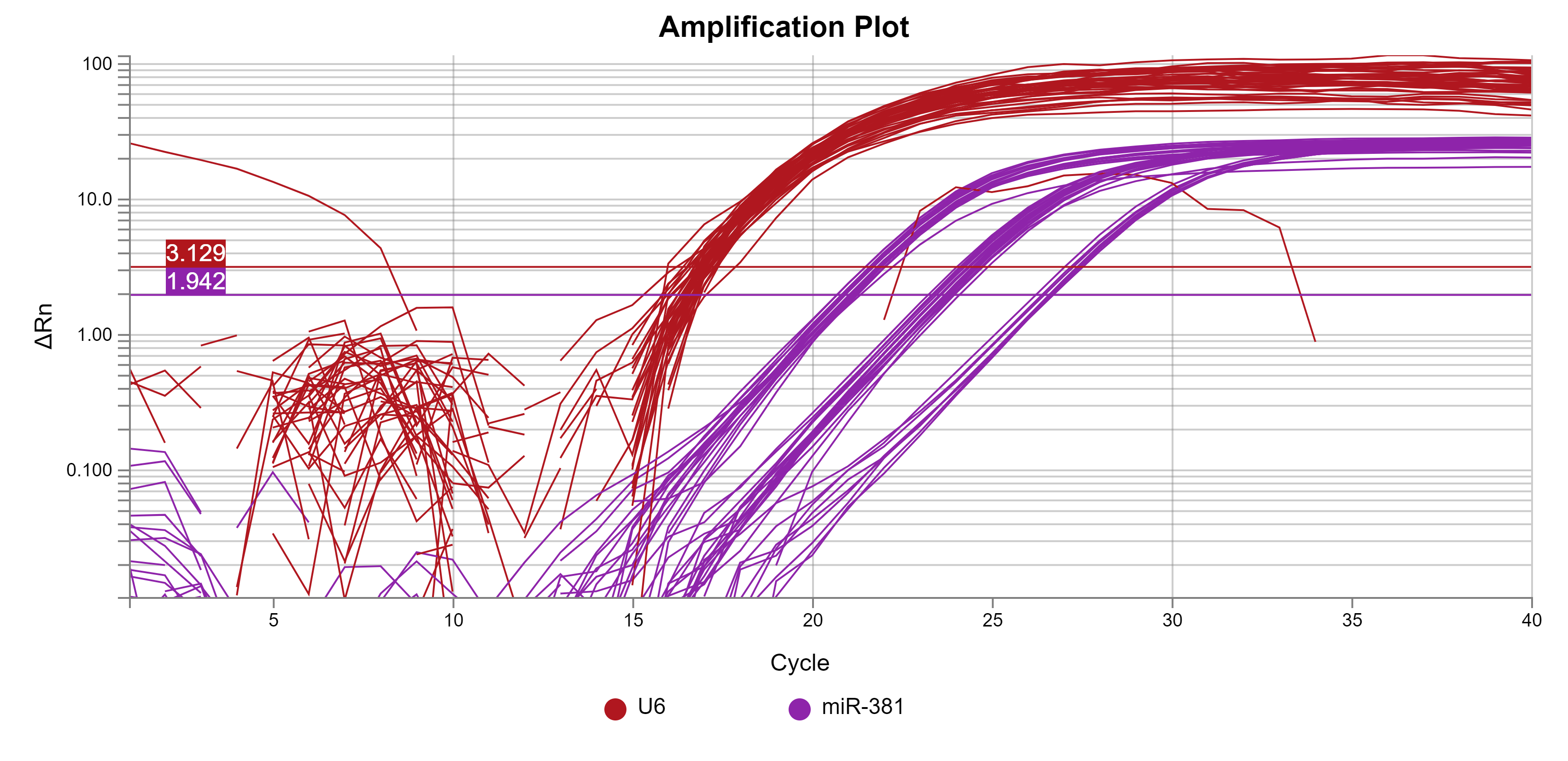

Supplement: S3 File — (ZIP) [file pone.0289818.s003.zip › S3 File. Fig3 Original data/date/3A/Amplification Plot_2023-05-16-11835.png]

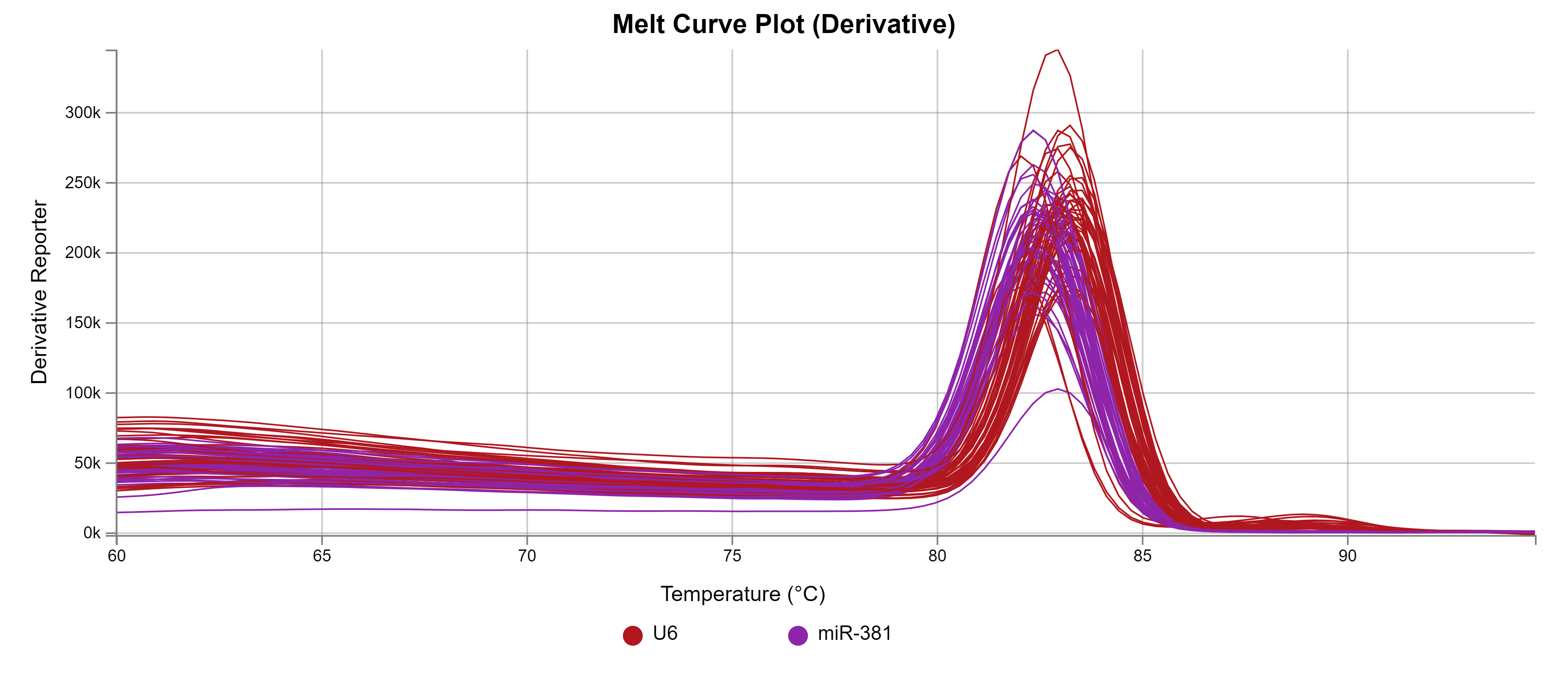

Supplement: S3 File — (ZIP) [file pone.0289818.s003.zip › S3 File. Fig3 Original data/date/3A/Melt Curve Plot_2023-05-16-1196.png]

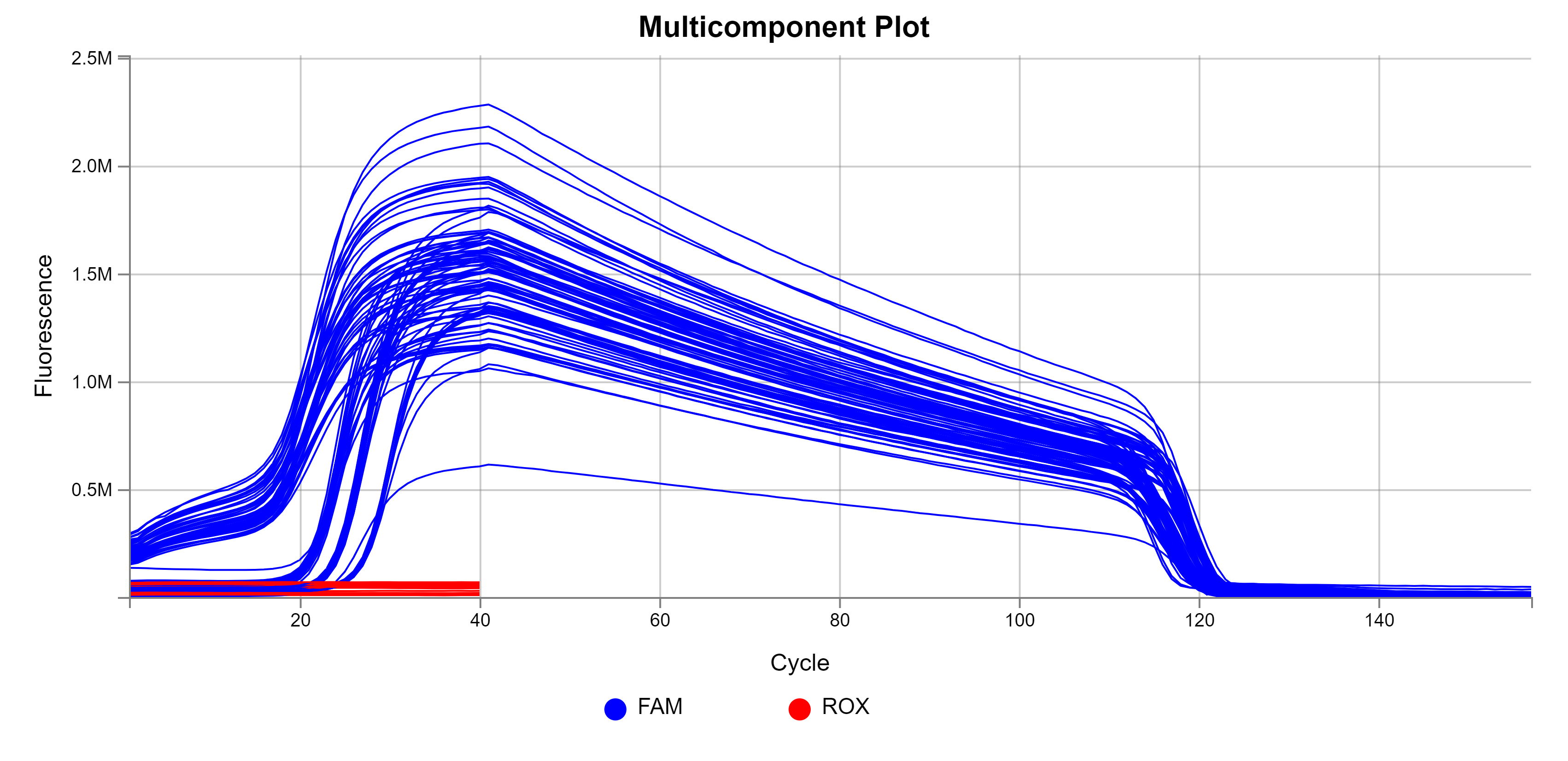

Supplement: S3 File — (ZIP) [file pone.0289818.s003.zip › S3 File. Fig3 Original data/date/3A/Multicomponent Plot_2023-05-16-11846.png]

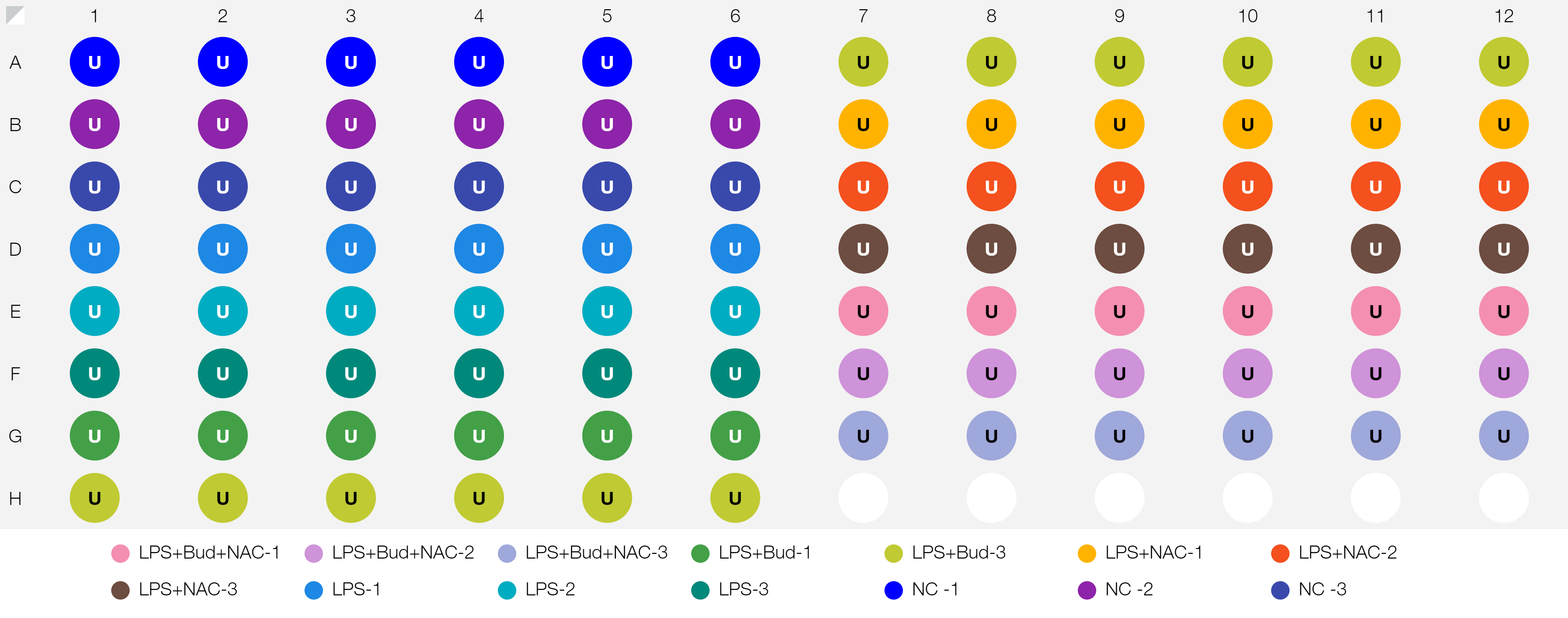

Supplement: S3 File — (ZIP) [file pone.0289818.s003.zip › S3 File. Fig3 Original data/date/3A/Plate_2023-05-16-11925.png]

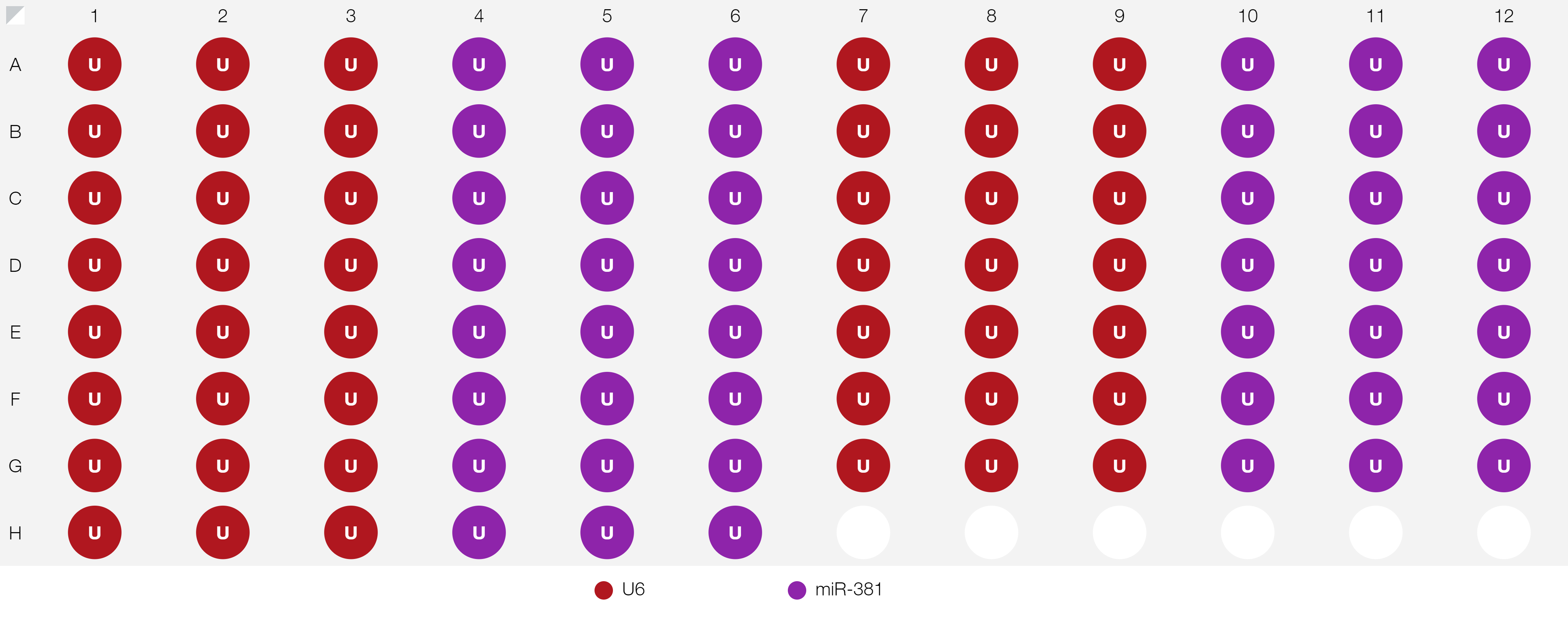

Supplement: S3 File — (ZIP) [file pone.0289818.s003.zip › S3 File. Fig3 Original data/date/3A/Plate_2023-05-16-11938.png]

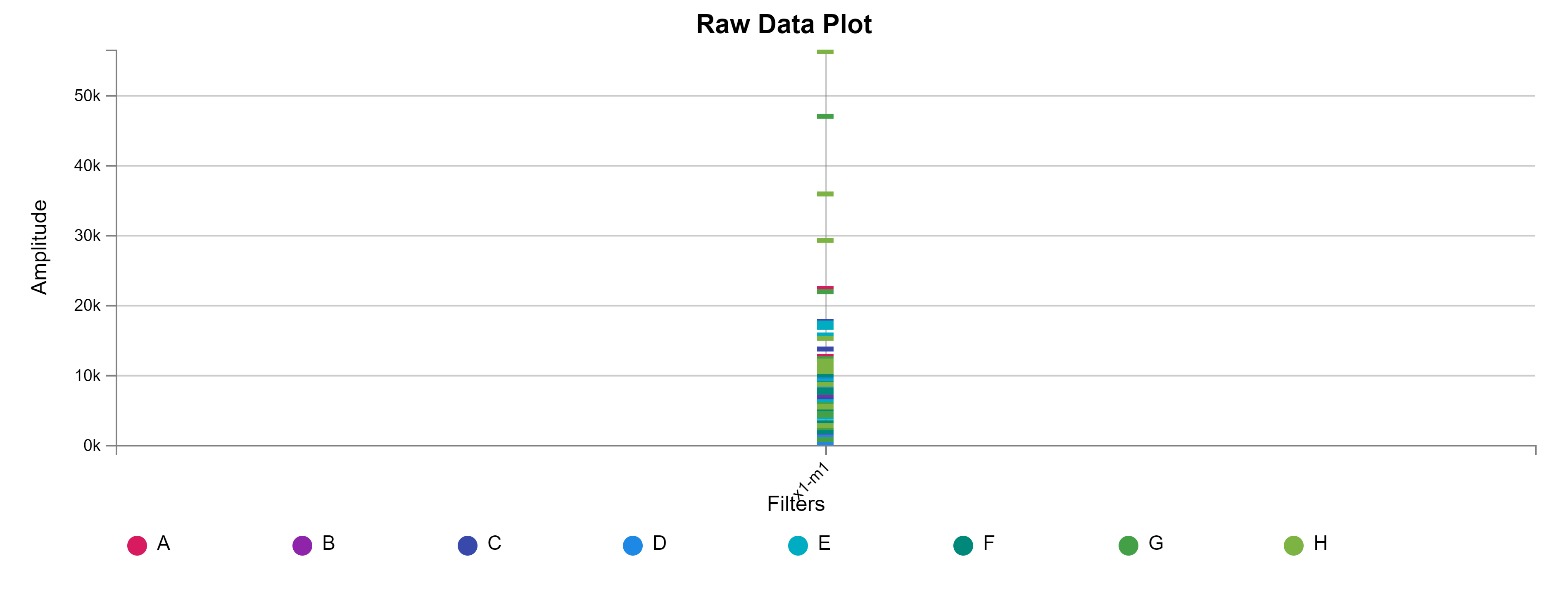

Supplement: S3 File — (ZIP) [file pone.0289818.s003.zip › S3 File. Fig3 Original data/date/3A/Raw Data Plot_2023-05-16-1190.png]

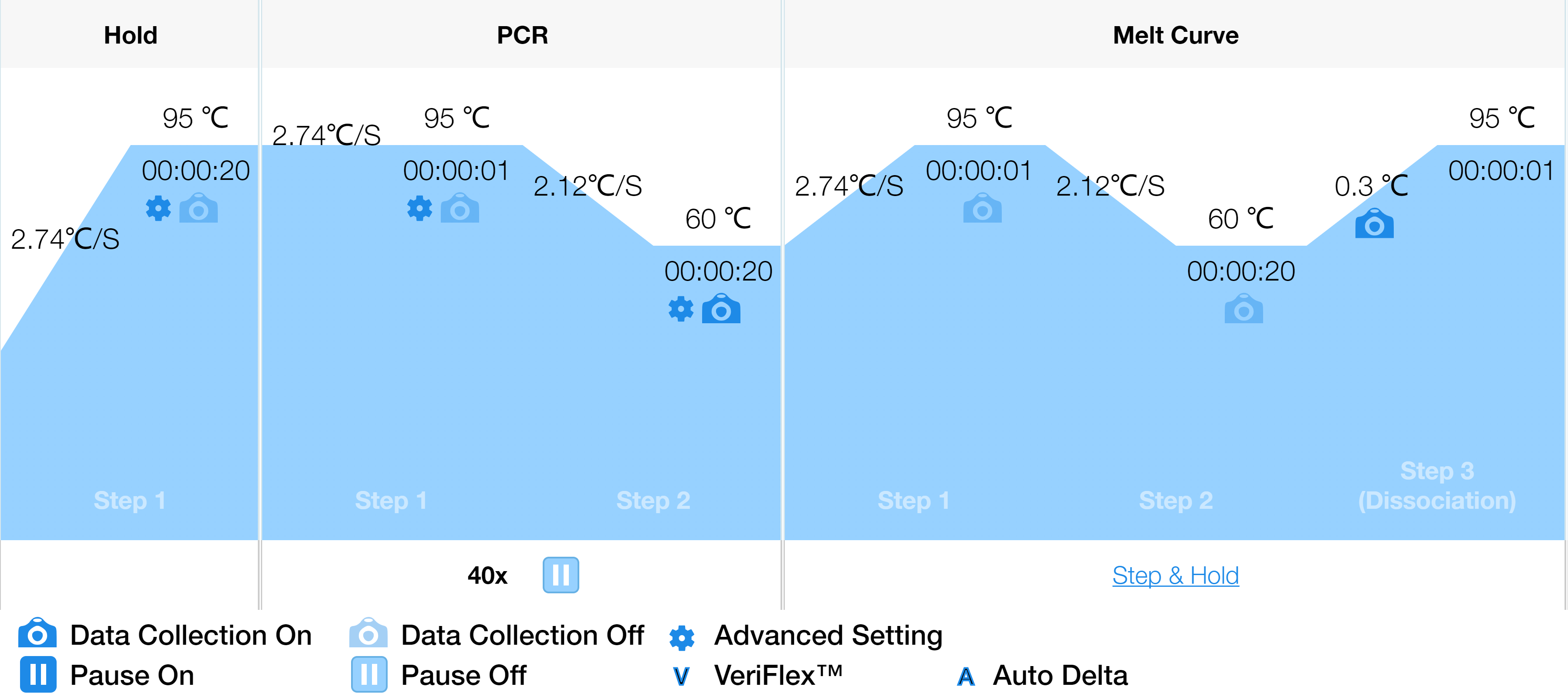

Supplement: S3 File — (ZIP) [file pone.0289818.s003.zip › S3 File. Fig3 Original data/date/3A/Run_Protocol_2023-05-16-11820.png]

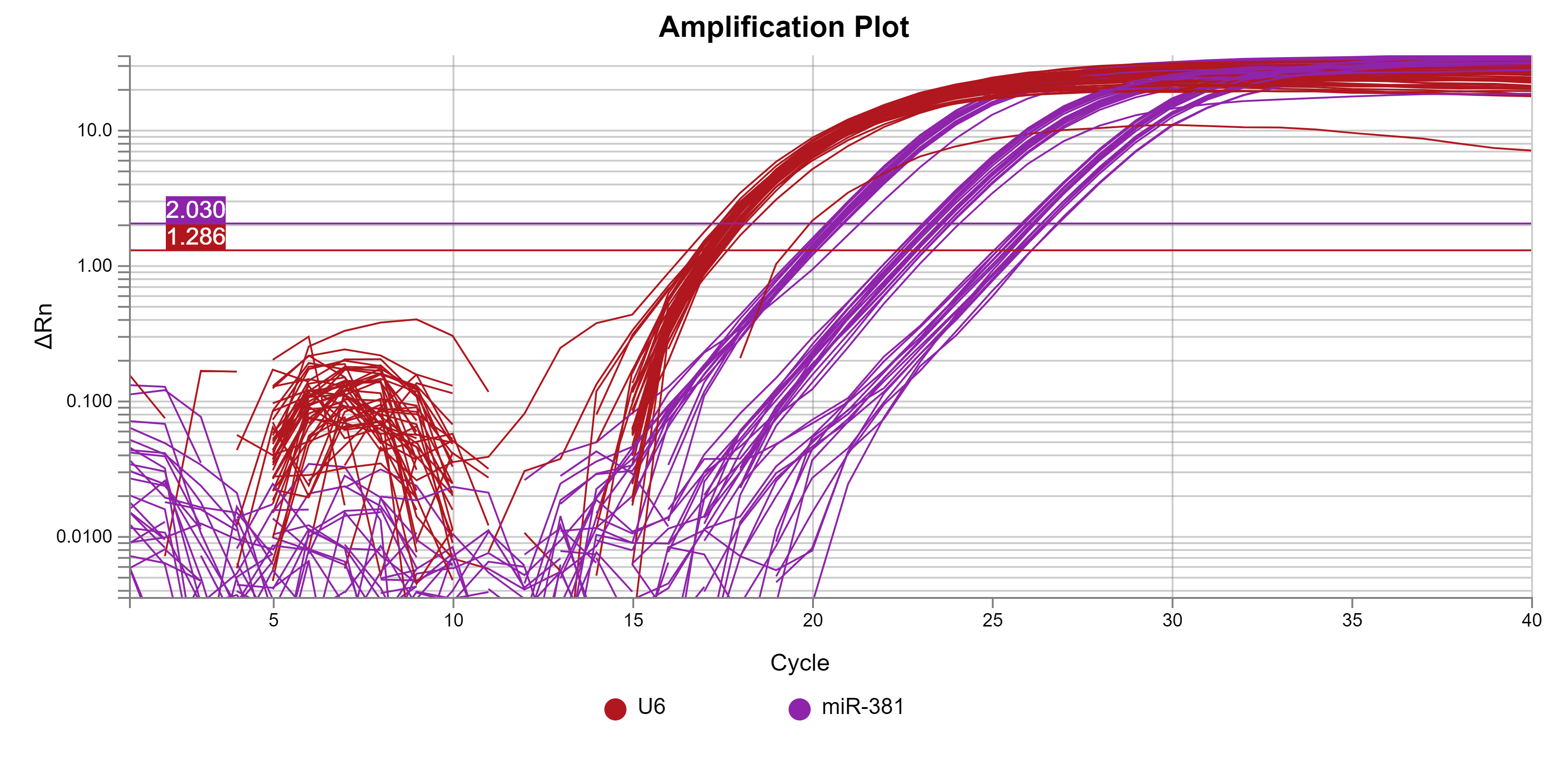

Supplement: S3 File — (ZIP) [file pone.0289818.s003.zip › S3 File. Fig3 Original data/date/3B/1-1/Amplification Plot_2023-05-16-112752.png]

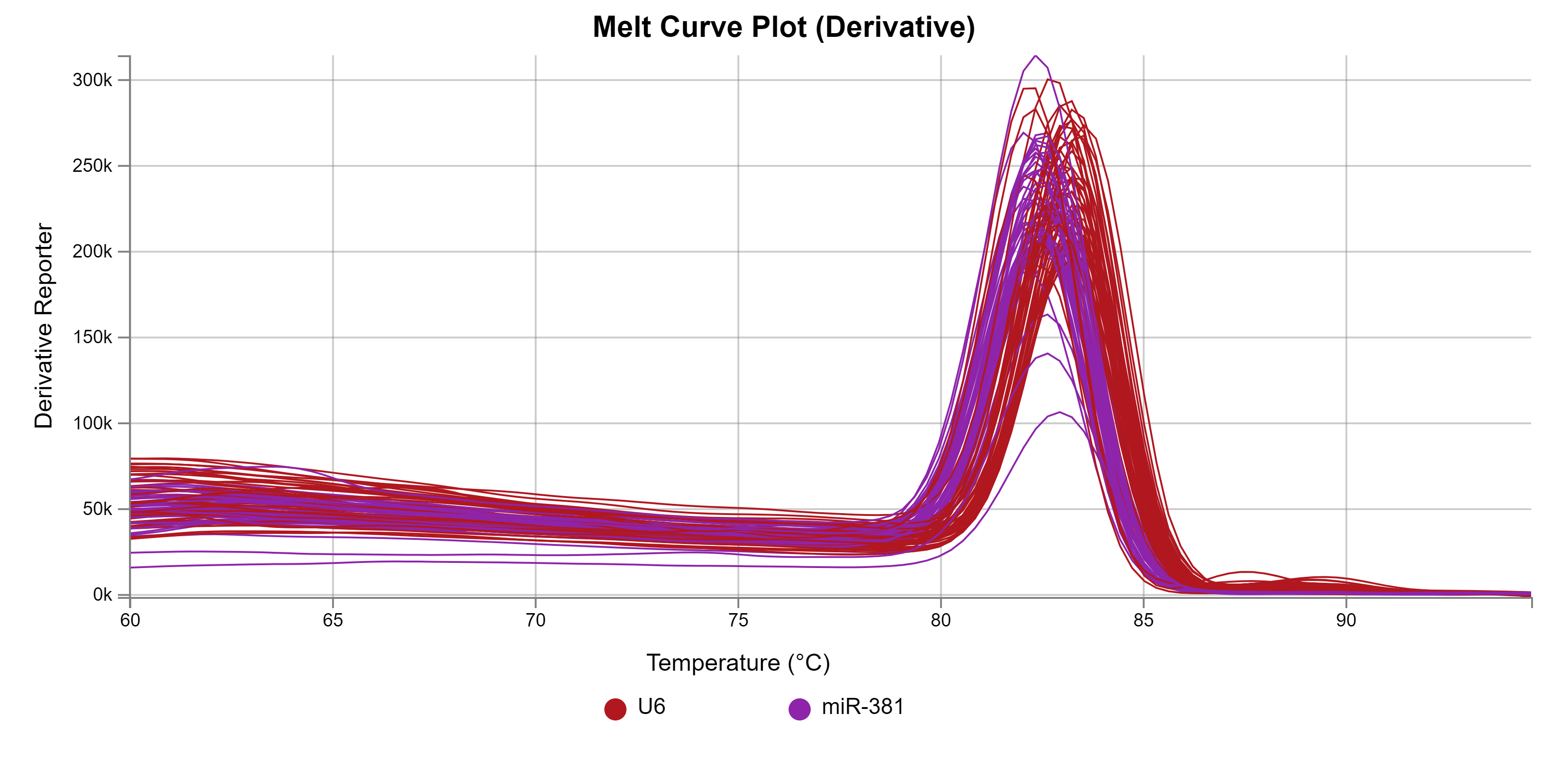

Supplement: S3 File — (ZIP) [file pone.0289818.s003.zip › S3 File. Fig3 Original data/date/3B/1-1/Melt Curve Plot_2023-05-16-112816.png]

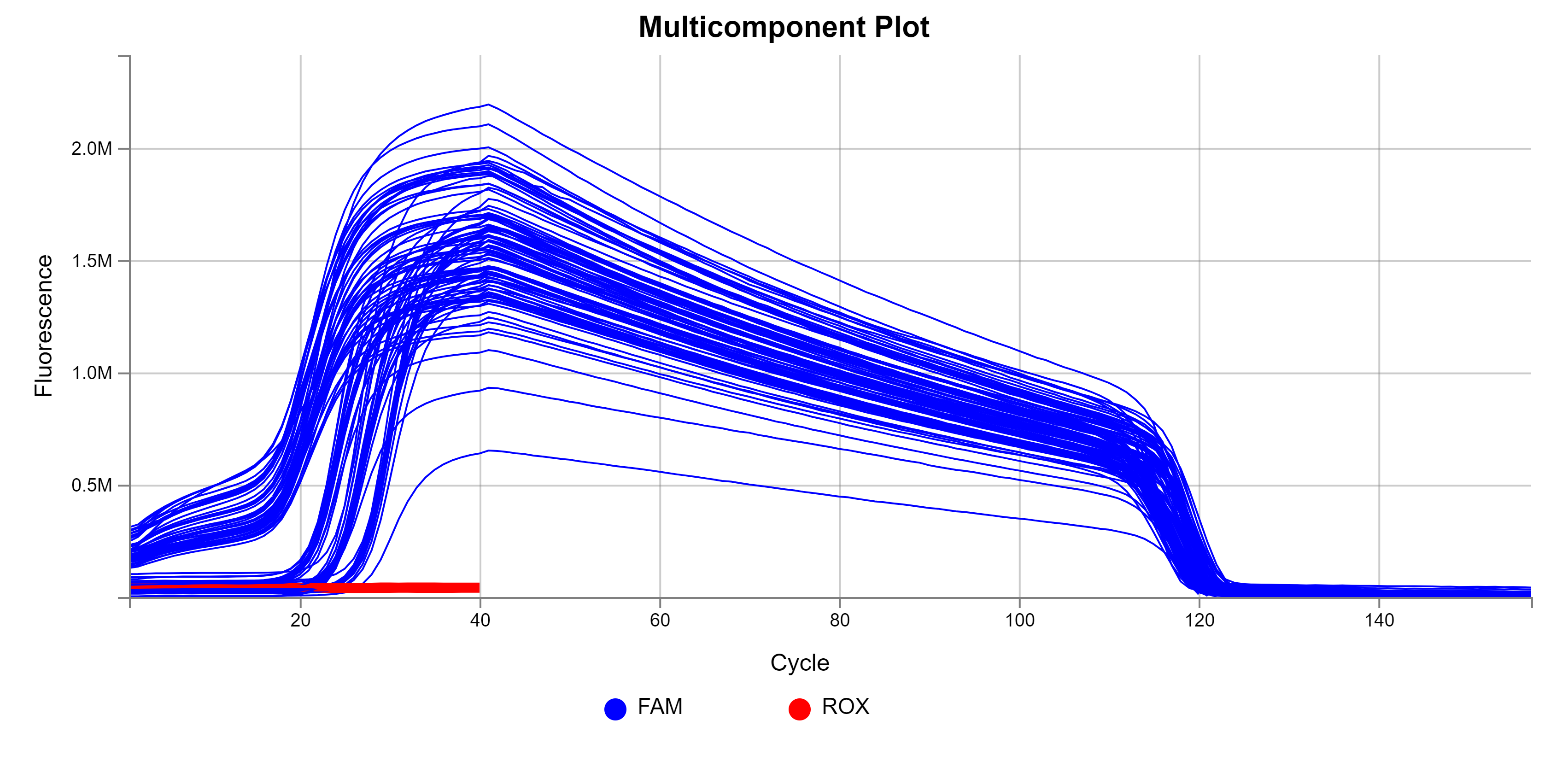

Supplement: S3 File — (ZIP) [file pone.0289818.s003.zip › S3 File. Fig3 Original data/date/3B/1-1/Multicomponent Plot_2023-05-16-11284.png]

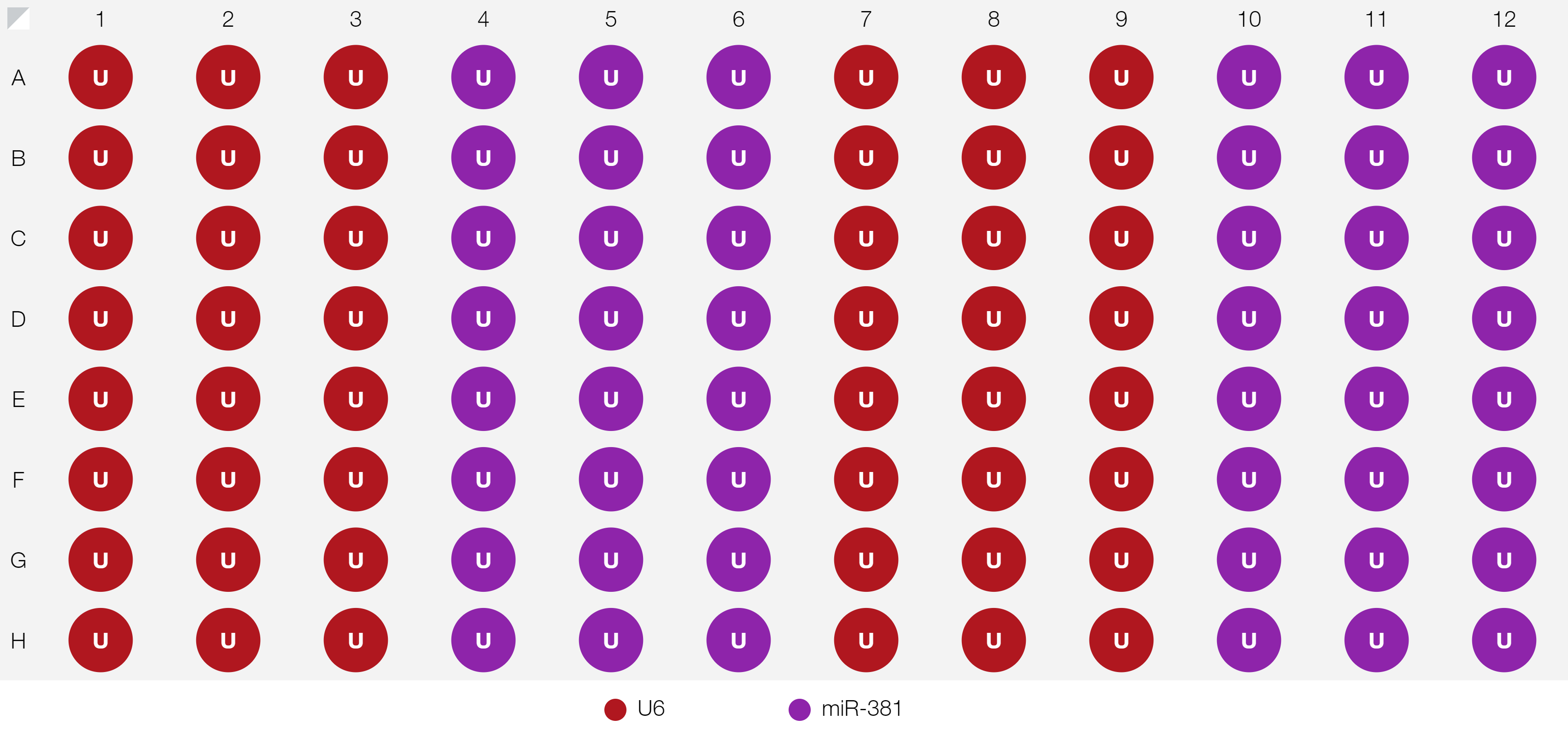

Supplement: S3 File — (ZIP) [file pone.0289818.s003.zip › S3 File. Fig3 Original data/date/3B/1-1/Plate_2023-05-16-112822.png]

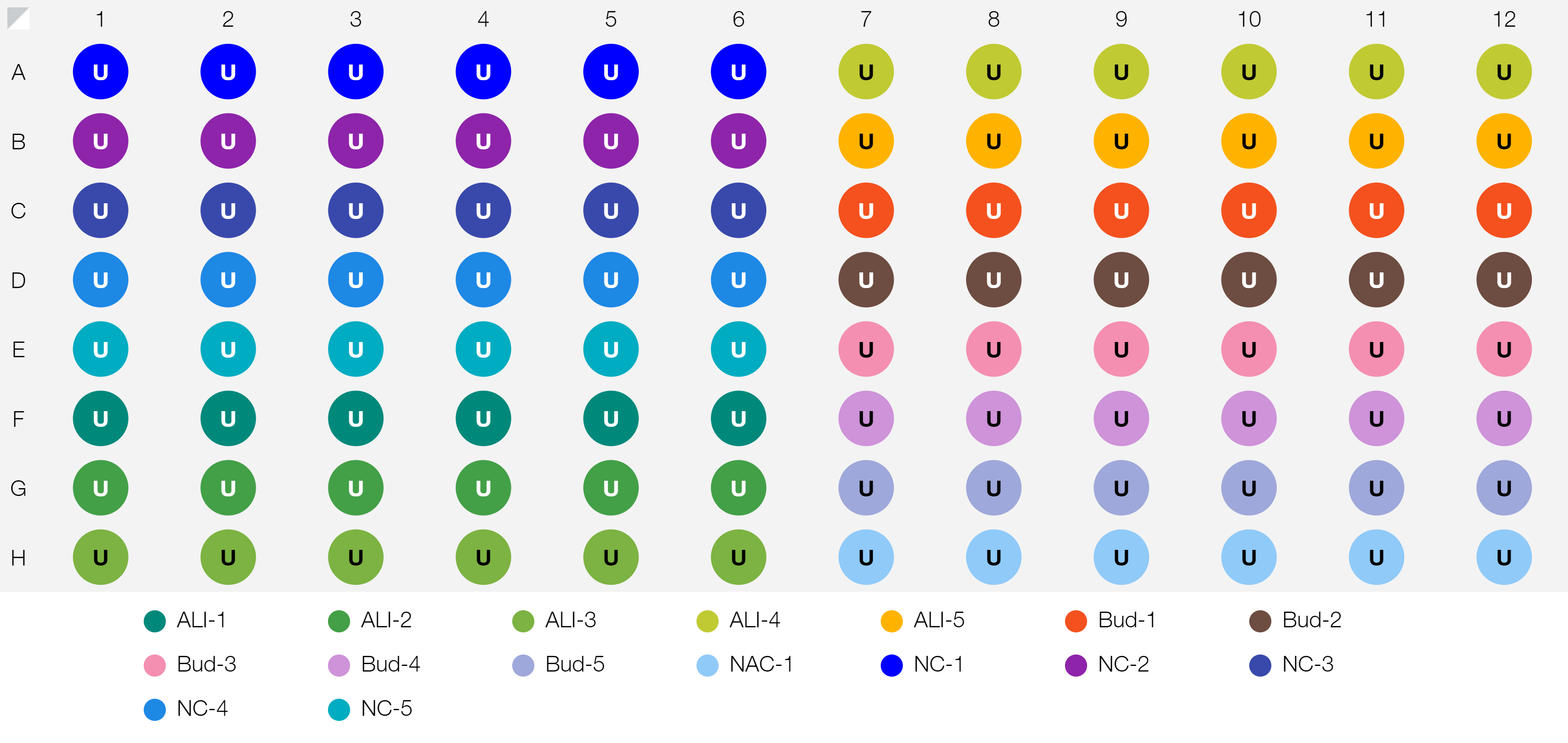

Supplement: S3 File — (ZIP) [file pone.0289818.s003.zip › S3 File. Fig3 Original data/date/3B/1-1/Plate_2023-05-16-112835.png]

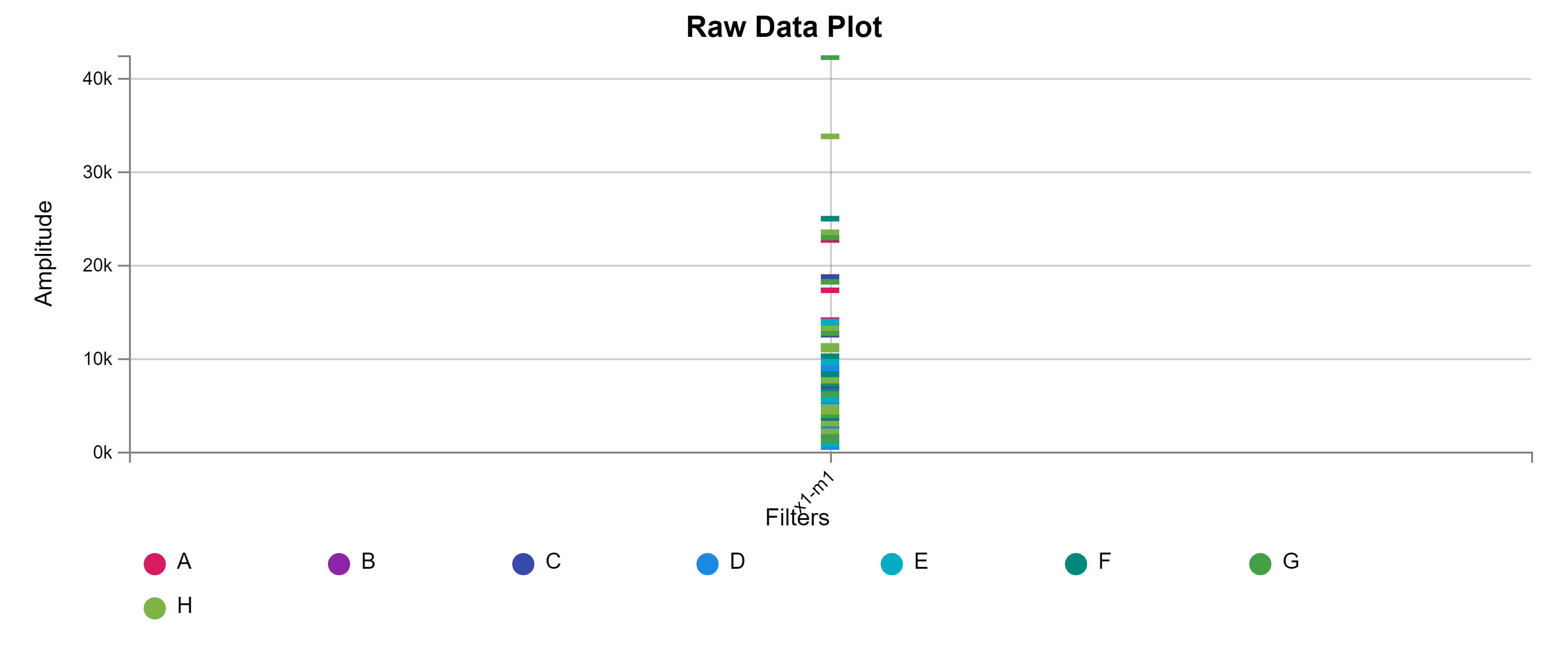

Supplement: S3 File — (ZIP) [file pone.0289818.s003.zip › S3 File. Fig3 Original data/date/3B/1-1/Raw Data Plot_2023-05-16-112811.png]

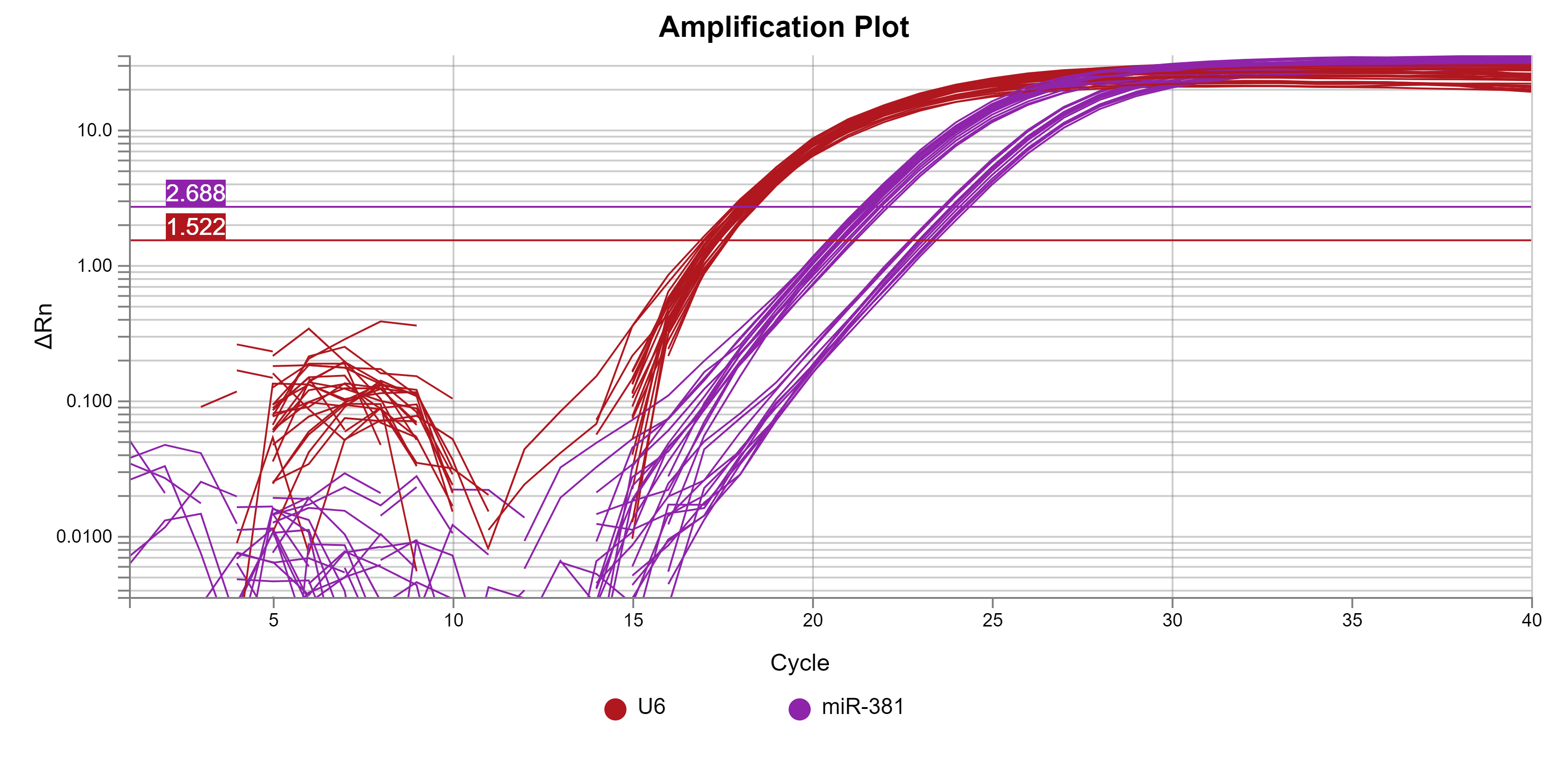

Supplement: S3 File — (ZIP) [file pone.0289818.s003.zip › S3 File. Fig3 Original data/date/3B/1-2/Amplification Plot_2023-05-16-113621.png]

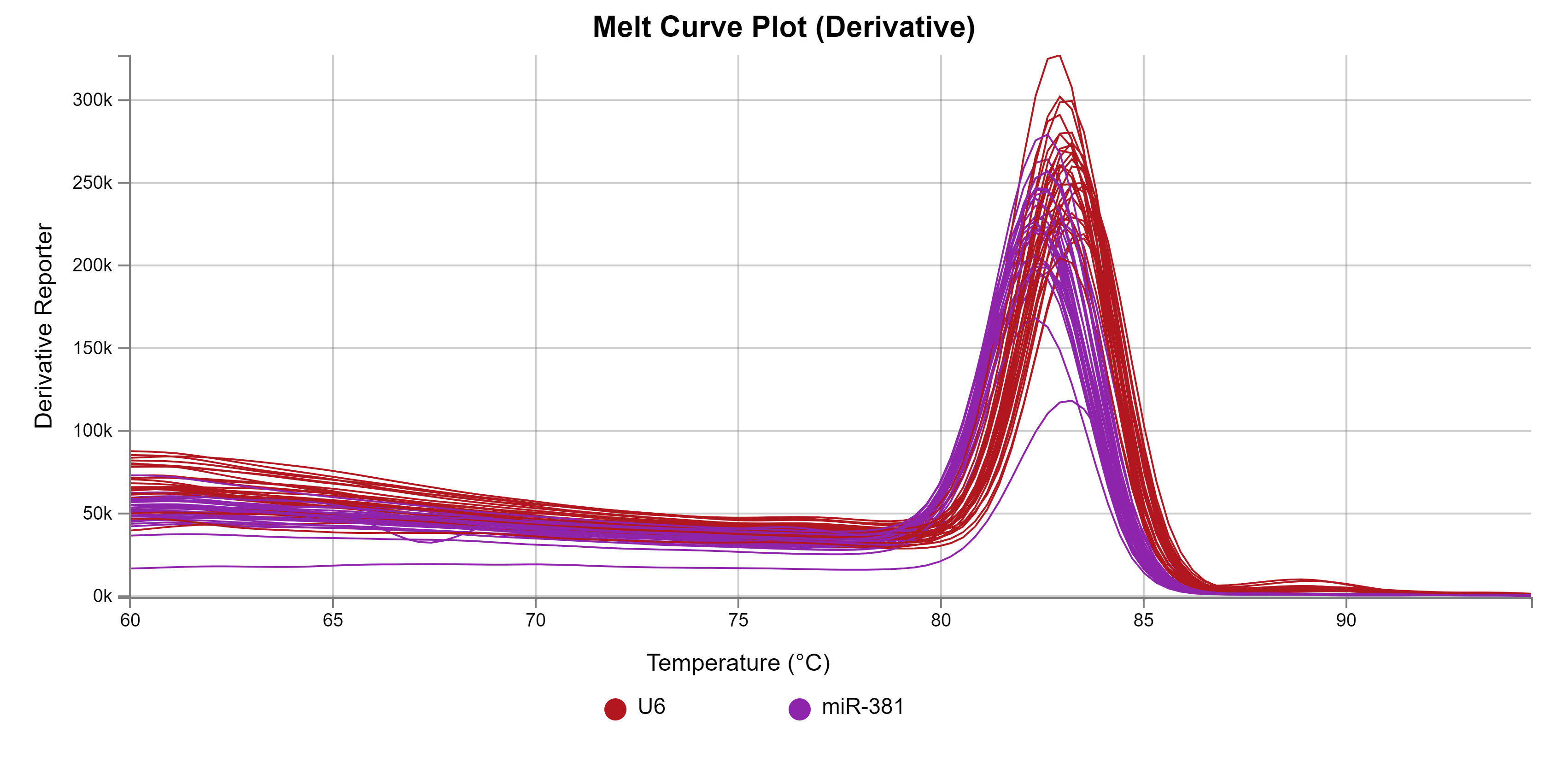

Supplement: S3 File — (ZIP) [file pone.0289818.s003.zip › S3 File. Fig3 Original data/date/3B/1-2/Melt Curve Plot_2023-05-16-113652.png]

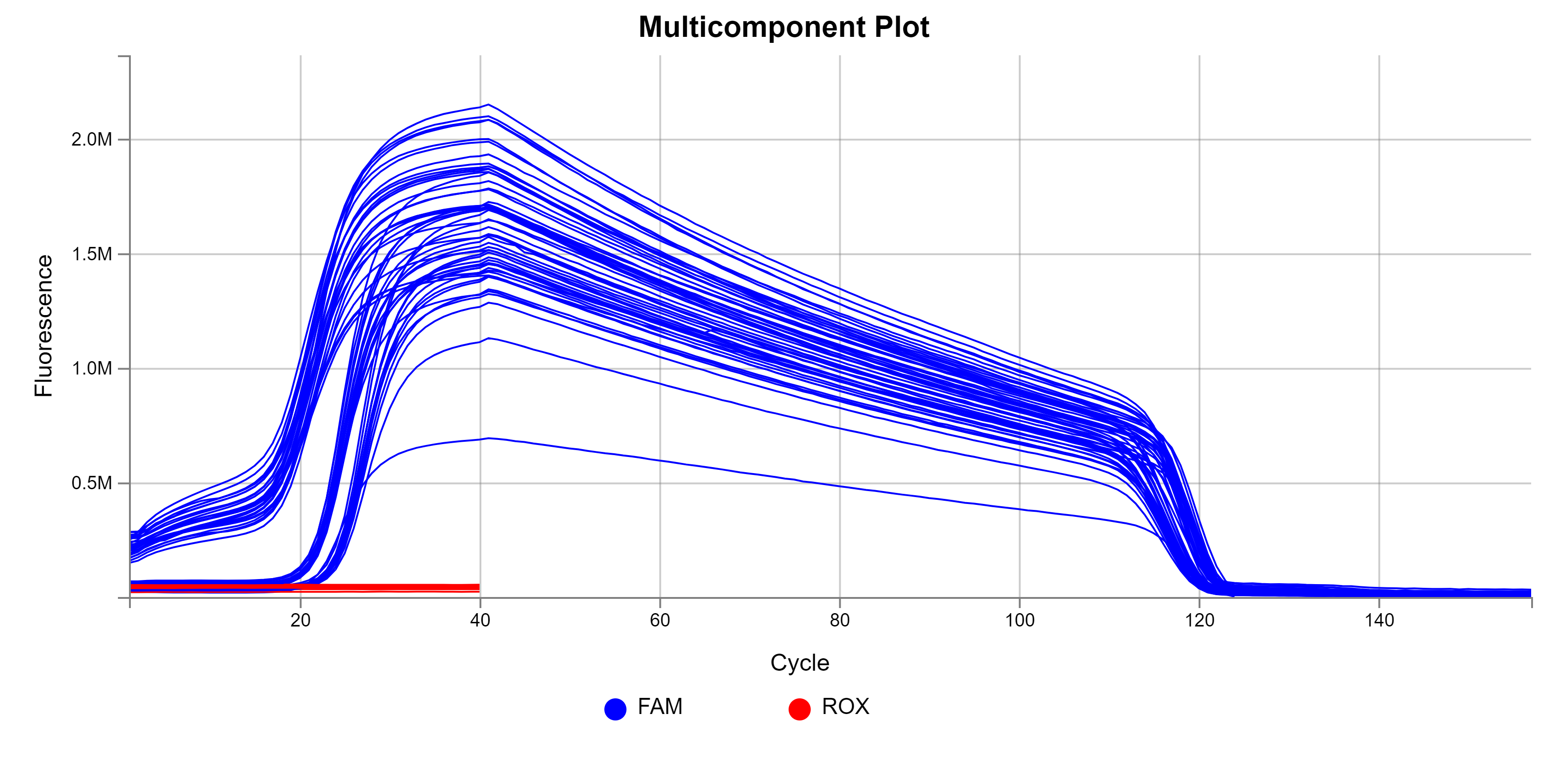

Supplement: S3 File — (ZIP) [file pone.0289818.s003.zip › S3 File. Fig3 Original data/date/3B/1-2/Multicomponent Plot_2023-05-16-113633.png]

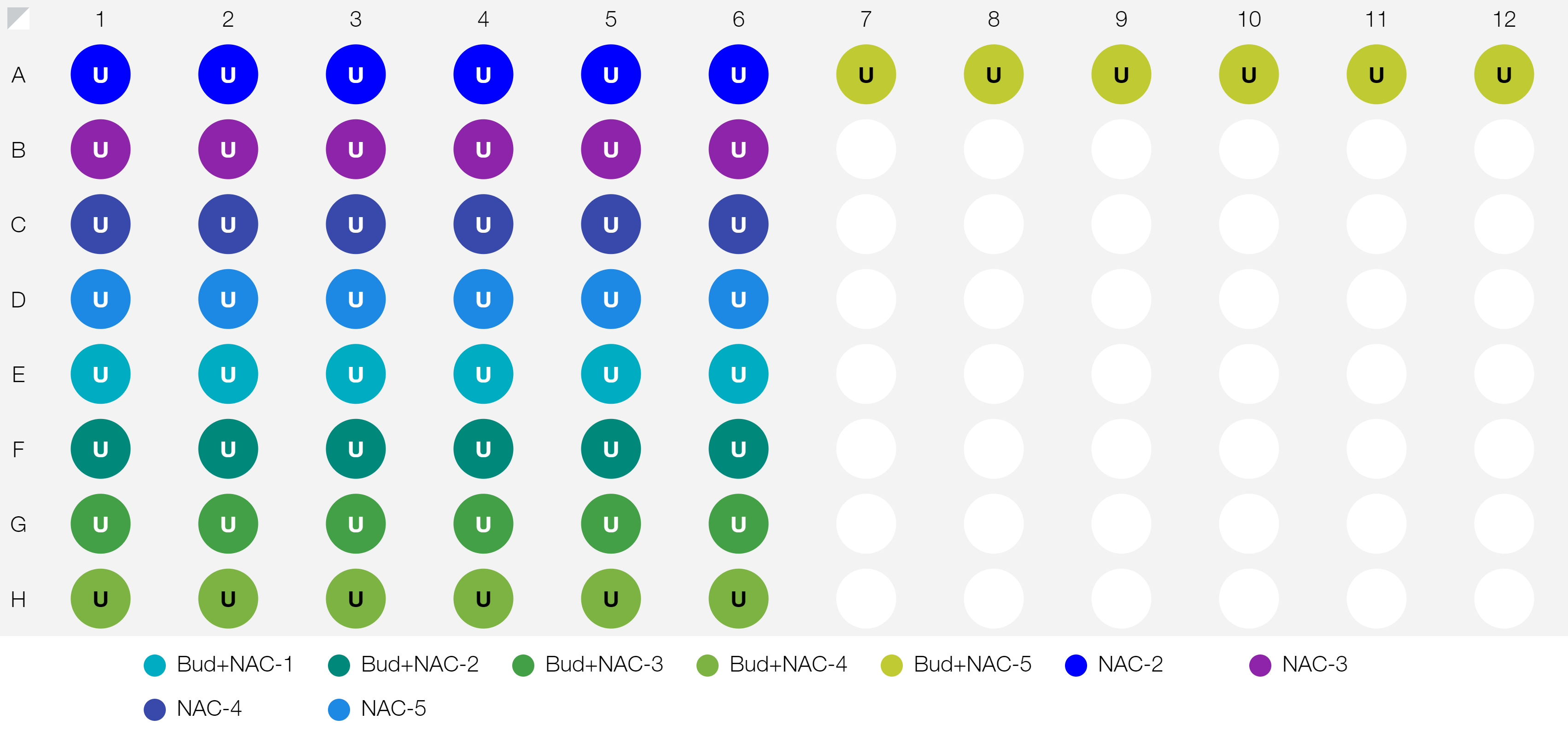

Supplement: S3 File — (ZIP) [file pone.0289818.s003.zip › S3 File. Fig3 Original data/date/3B/1-2/Plate_2023-05-16-113713.png]

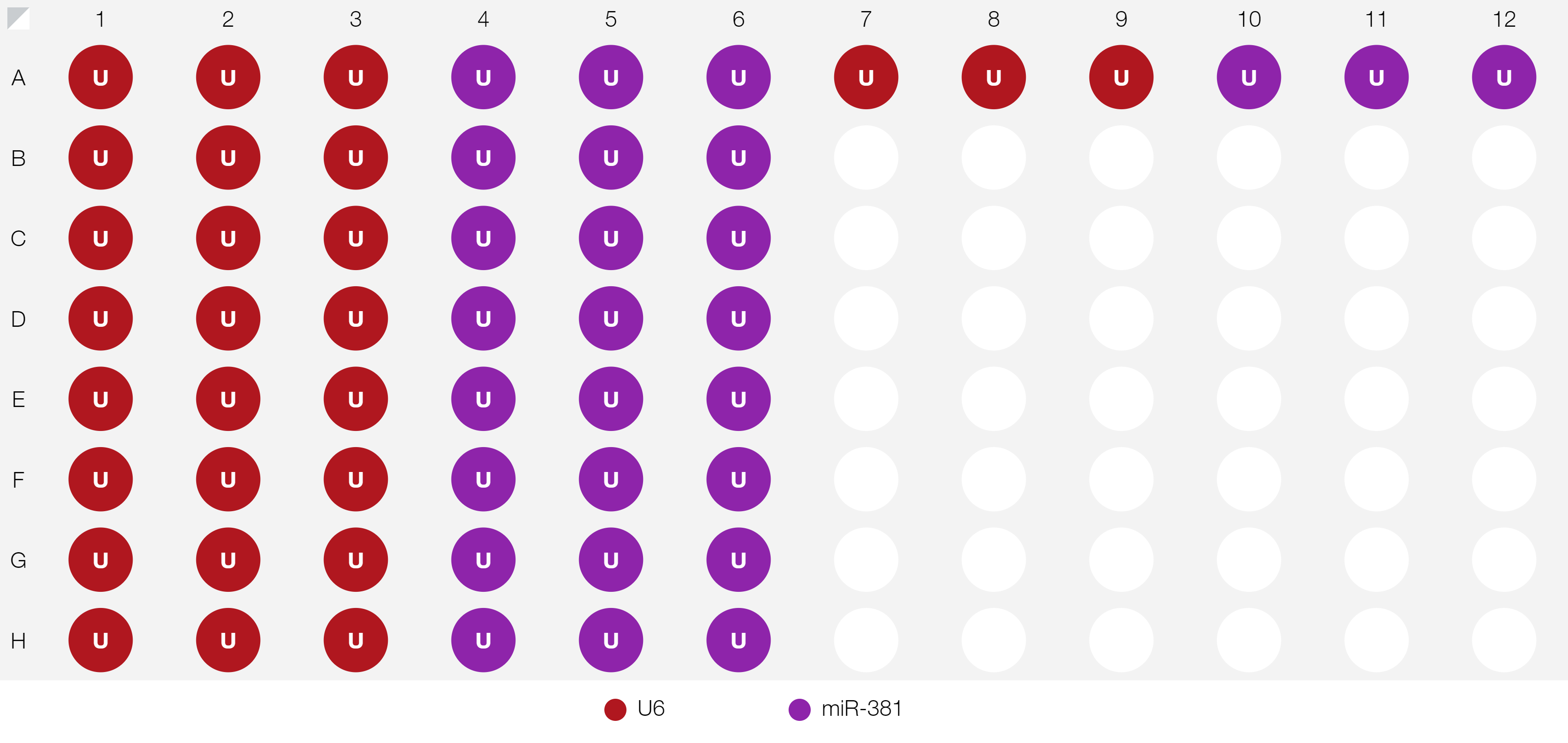

Supplement: S3 File — (ZIP) [file pone.0289818.s003.zip › S3 File. Fig3 Original data/date/3B/1-2/Plate_2023-05-16-113723.png]

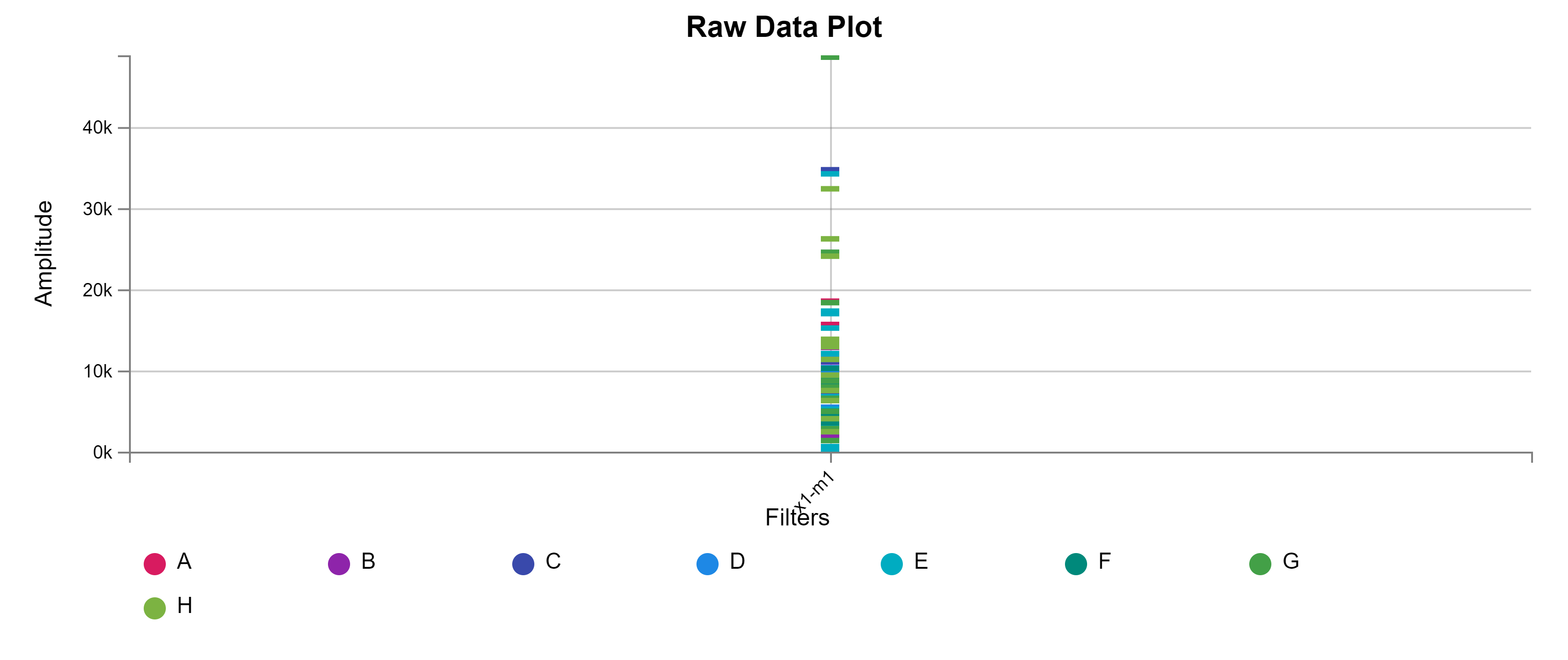

Supplement: S3 File — (ZIP) [file pone.0289818.s003.zip › S3 File. Fig3 Original data/date/3B/1-2/Raw Data Plot_2023-05-16-113644.png]

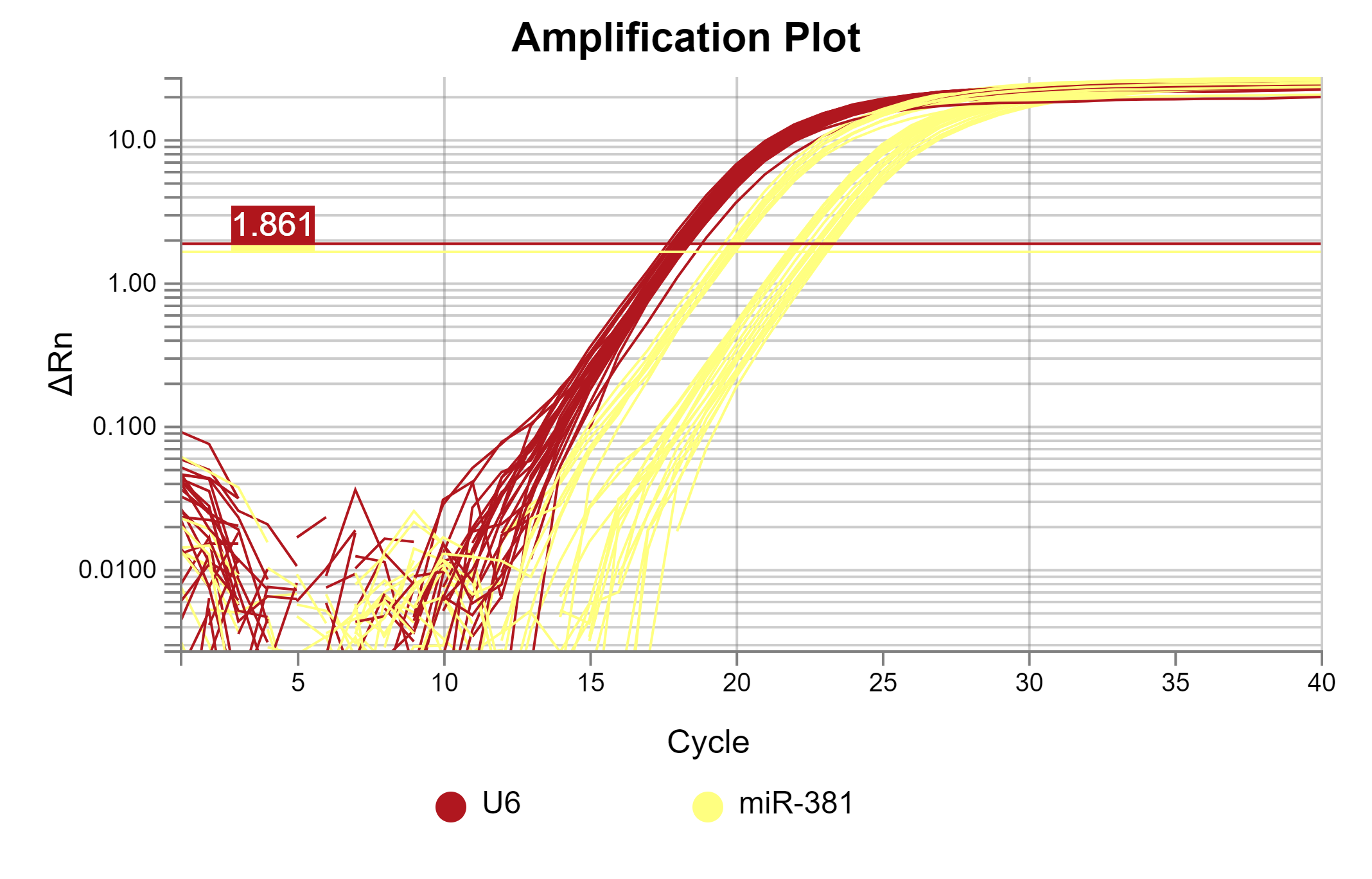

Supplement: S3 File — (ZIP) [file pone.0289818.s003.zip › S3 File. Fig3 Original data/date/3C/Amplification Plot_2023-05-16-114415.png]

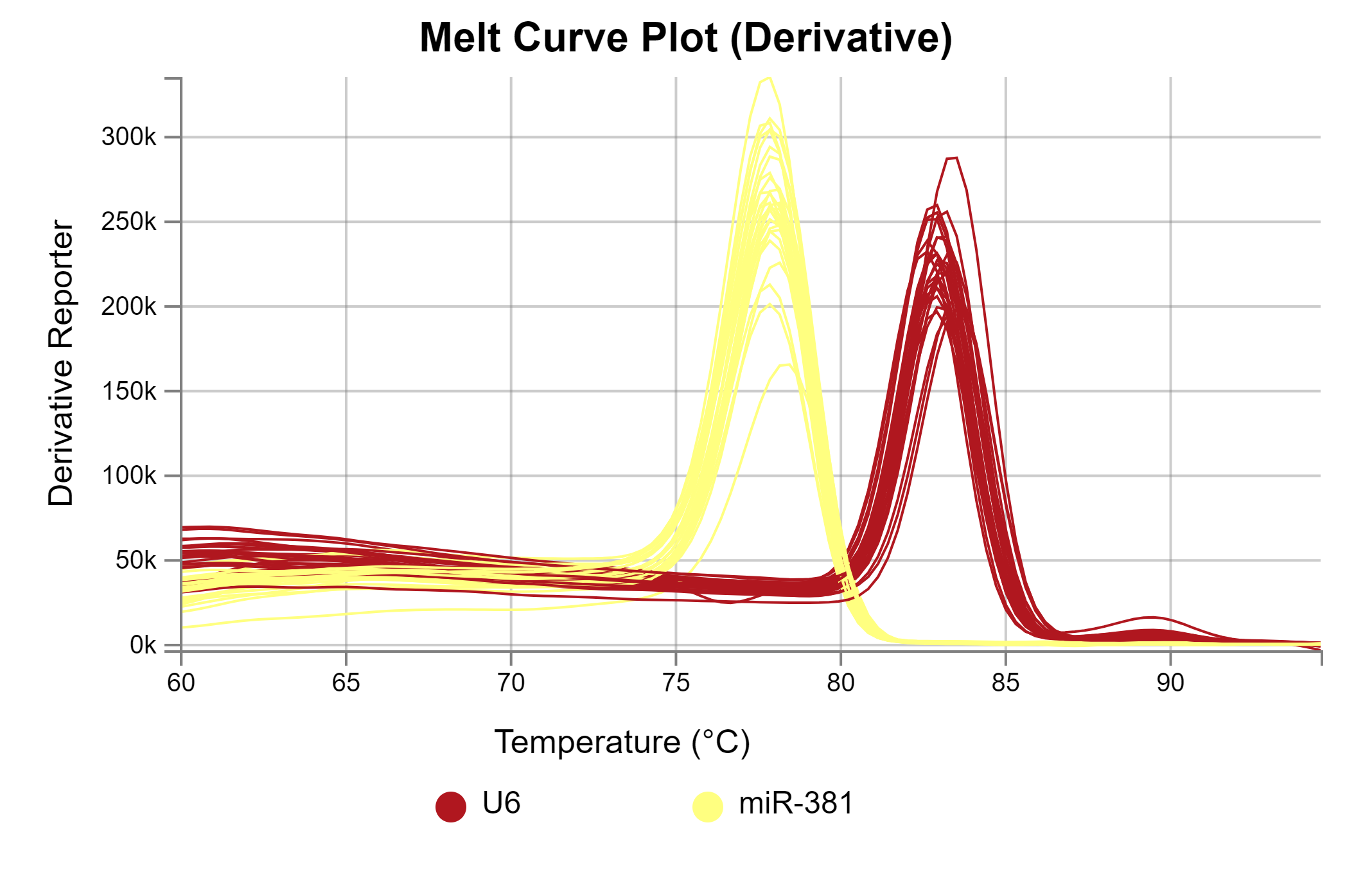

Supplement: S3 File — (ZIP) [file pone.0289818.s003.zip › S3 File. Fig3 Original data/date/3C/Melt Curve Plot_2023-05-16-114439.png]

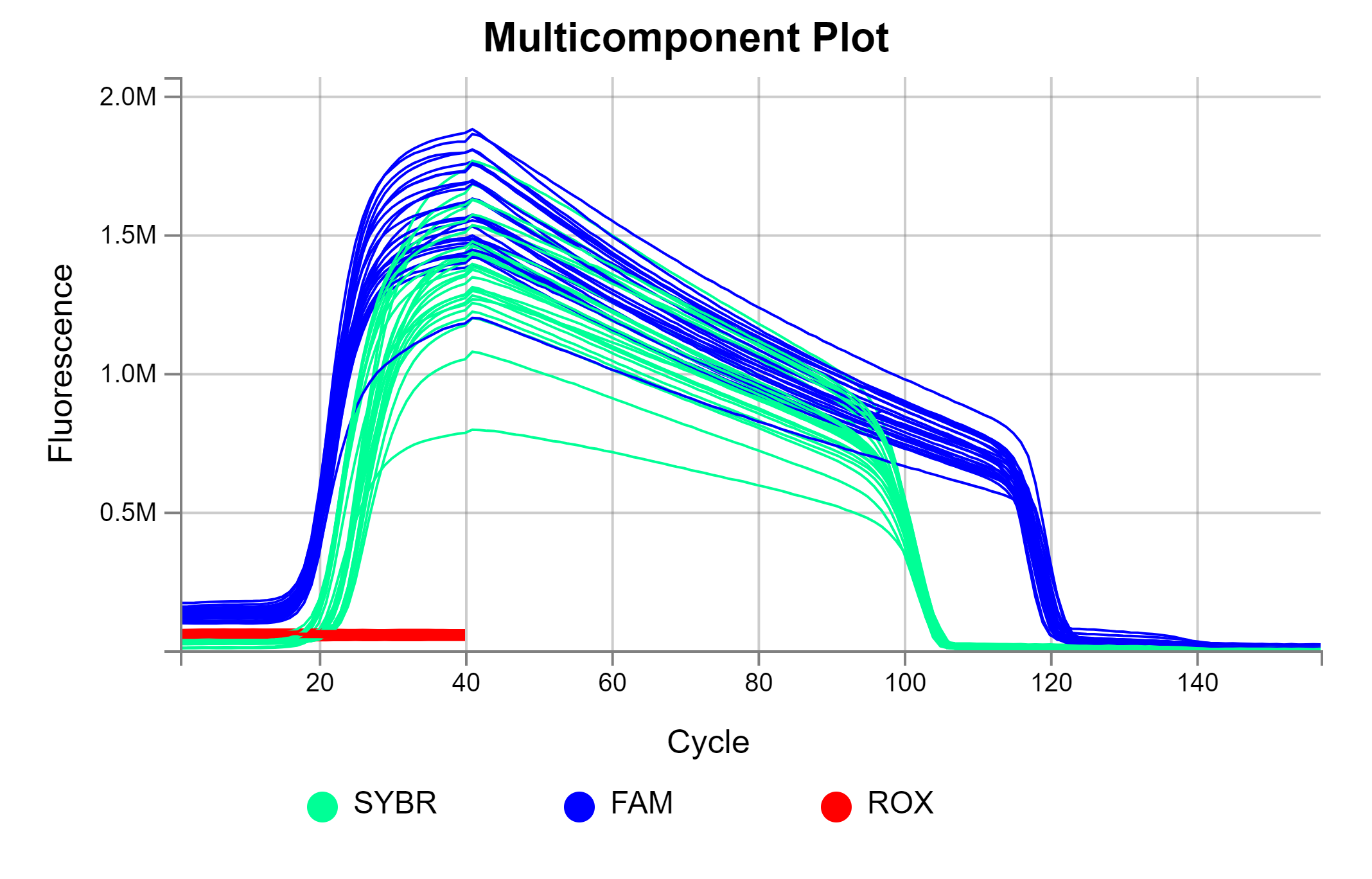

Supplement: S3 File — (ZIP) [file pone.0289818.s003.zip › S3 File. Fig3 Original data/date/3C/Multicomponent Plot_2023-05-16-114426.png]

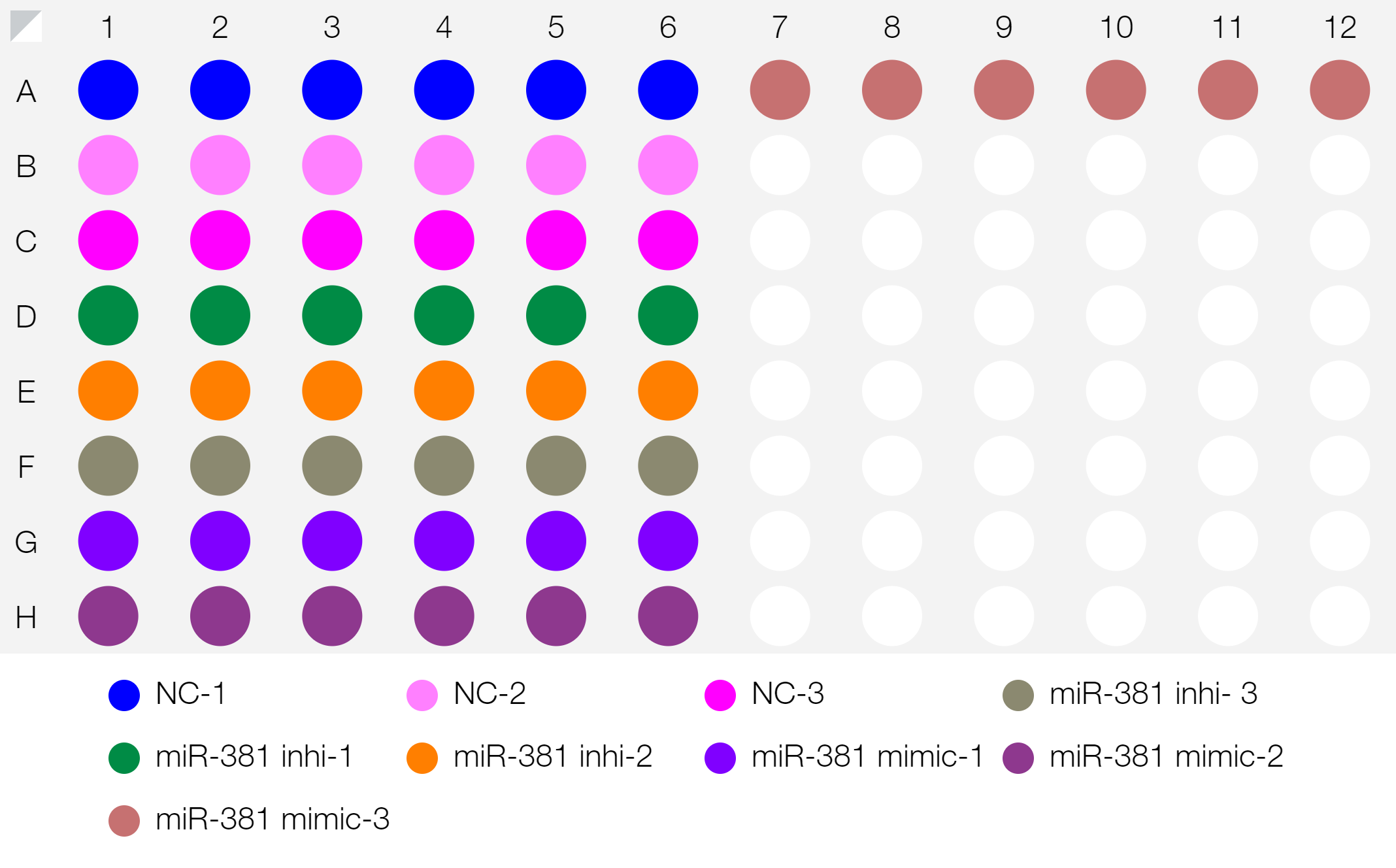

Supplement: S3 File — (ZIP) [file pone.0289818.s003.zip › S3 File. Fig3 Original data/date/3C/Plate_2023-05-16-114453.png]

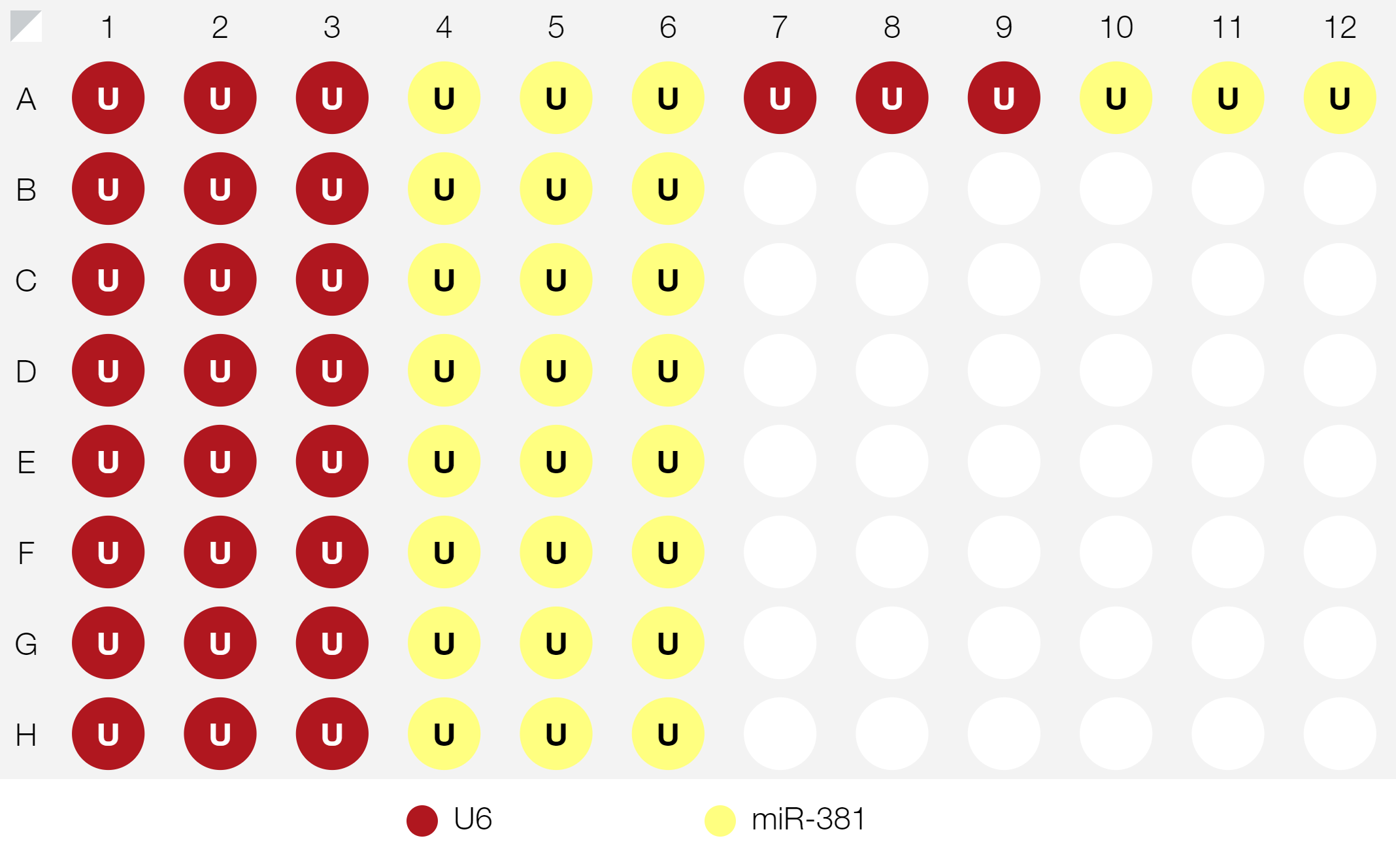

Supplement: S3 File — (ZIP) [file pone.0289818.s003.zip › S3 File. Fig3 Original data/date/3C/Plate_2023-05-16-11452.png]

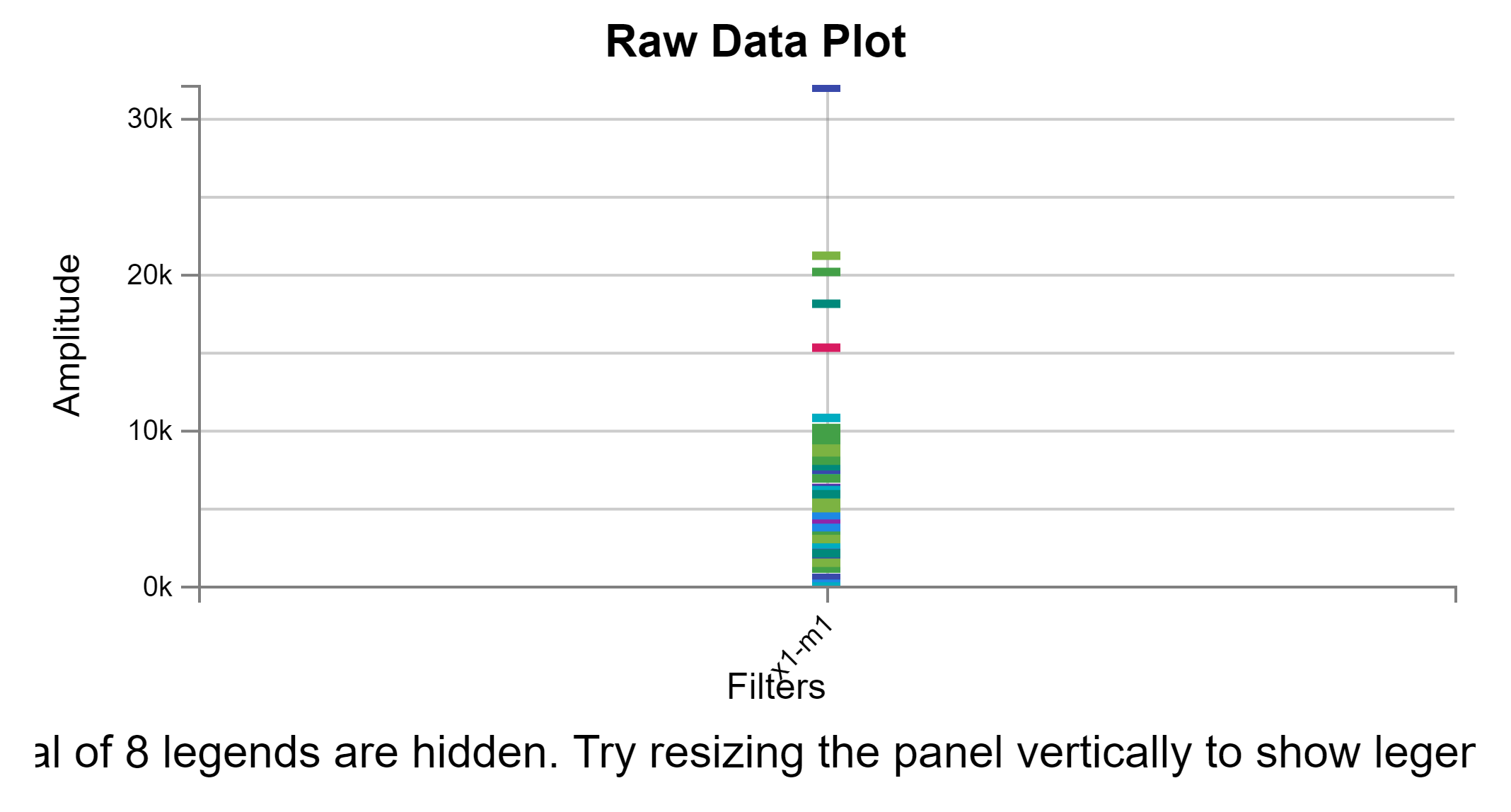

Supplement: S3 File — (ZIP) [file pone.0289818.s003.zip › S3 File. Fig3 Original data/date/3C/Raw Data Plot_2023-05-16-114432.png]

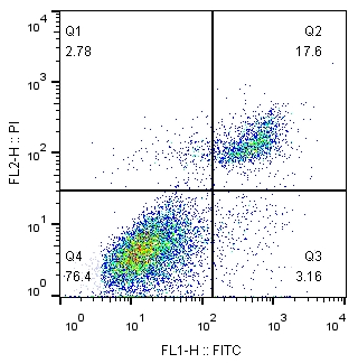

Supplement: S3 File — (ZIP) [file pone.0289818.s003.zip › S3 File. Fig3 Original data/image/3E/LPS/1 (1).jpg]

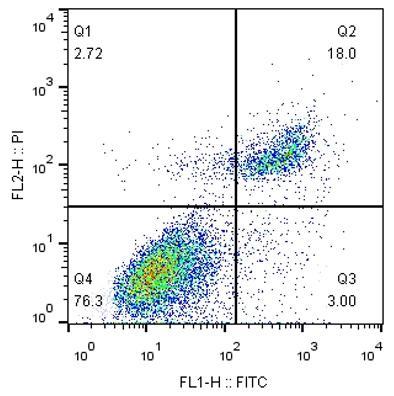

Supplement: S3 File — (ZIP) [file pone.0289818.s003.zip › S3 File. Fig3 Original data/image/3E/LPS/1 (2).jpg]

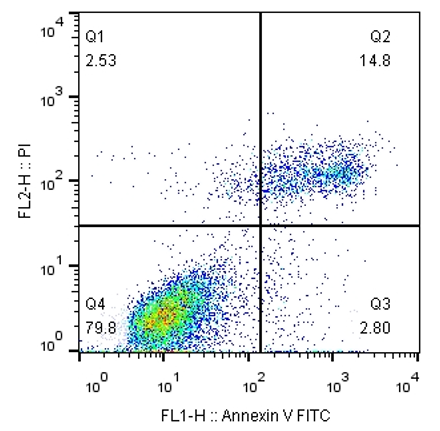

Supplement: S3 File — (ZIP) [file pone.0289818.s003.zip › S3 File. Fig3 Original data/image/3E/LPS/1 (3).jpg]

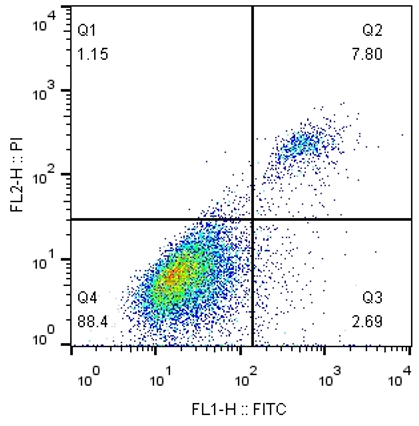

Supplement: S3 File — (ZIP) [file pone.0289818.s003.zip › S3 File. Fig3 Original data/image/3E/LPS+Bud+NAC/1 (1).jpg]

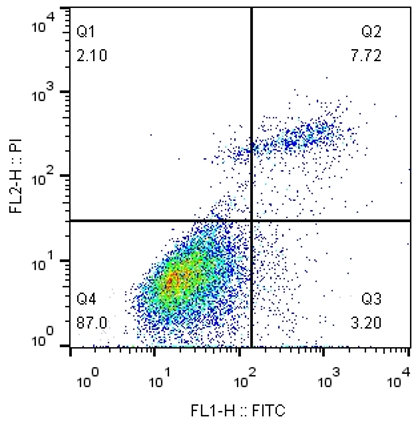

Supplement: S3 File — (ZIP) [file pone.0289818.s003.zip › S3 File. Fig3 Original data/image/3E/LPS+Bud+NAC/1 (2).jpg]

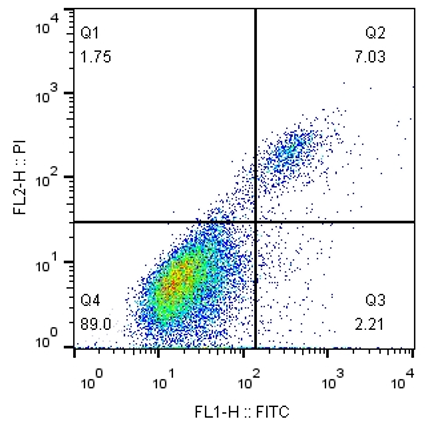

Supplement: S3 File — (ZIP) [file pone.0289818.s003.zip › S3 File. Fig3 Original data/image/3E/LPS+Bud+NAC/1 (3).jpg]

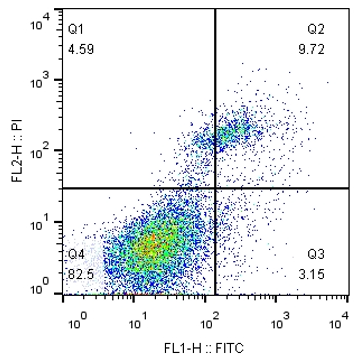

Supplement: S3 File — (ZIP) [file pone.0289818.s003.zip › S3 File. Fig3 Original data/image/3E/LPS+Bud+NAC+miR-381 inhi/1 (1).jpg]

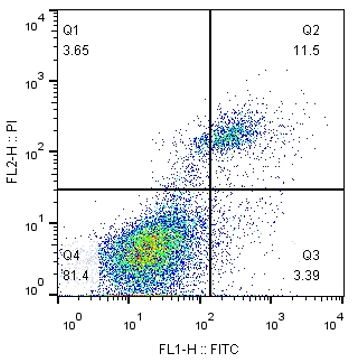

Supplement: S3 File — (ZIP) [file pone.0289818.s003.zip › S3 File. Fig3 Original data/image/3E/LPS+Bud+NAC+miR-381 inhi/1 (2).jpg]

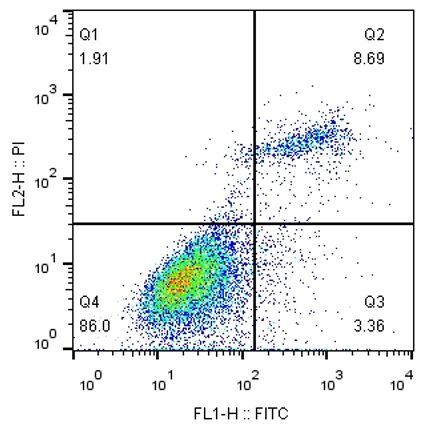

Supplement: S3 File — (ZIP) [file pone.0289818.s003.zip › S3 File. Fig3 Original data/image/3E/LPS+Bud+NAC+miR-381 inhi/1 (3).jpg]

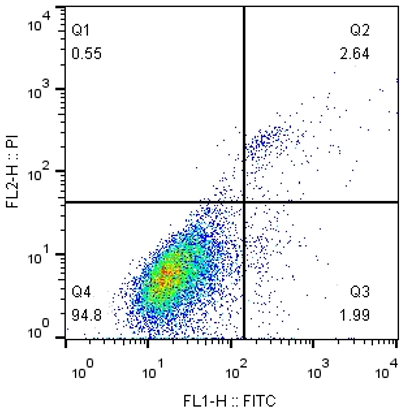

Supplement: S3 File — (ZIP) [file pone.0289818.s003.zip › S3 File. Fig3 Original data/image/3E/NC/4.63-16.134.jpg]

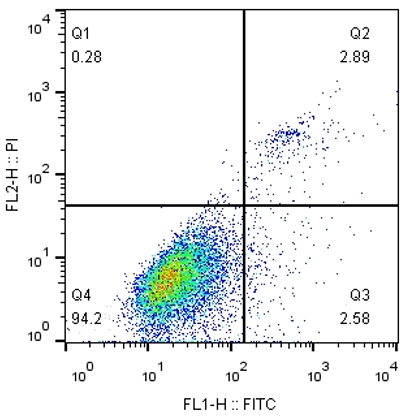

Supplement: S3 File — (ZIP) [file pone.0289818.s003.zip › S3 File. Fig3 Original data/image/3E/NC/5.47-22.140选.jpg]

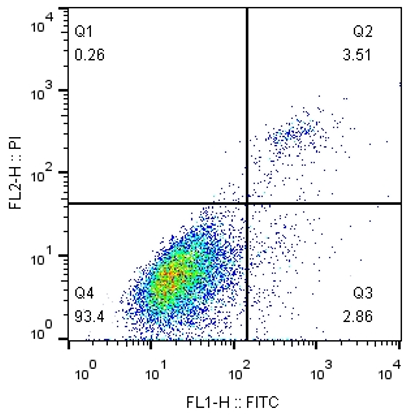

Supplement: S3 File — (ZIP) [file pone.0289818.s003.zip › S3 File. Fig3 Original data/image/3E/NC/6.47-23.141.jpg]

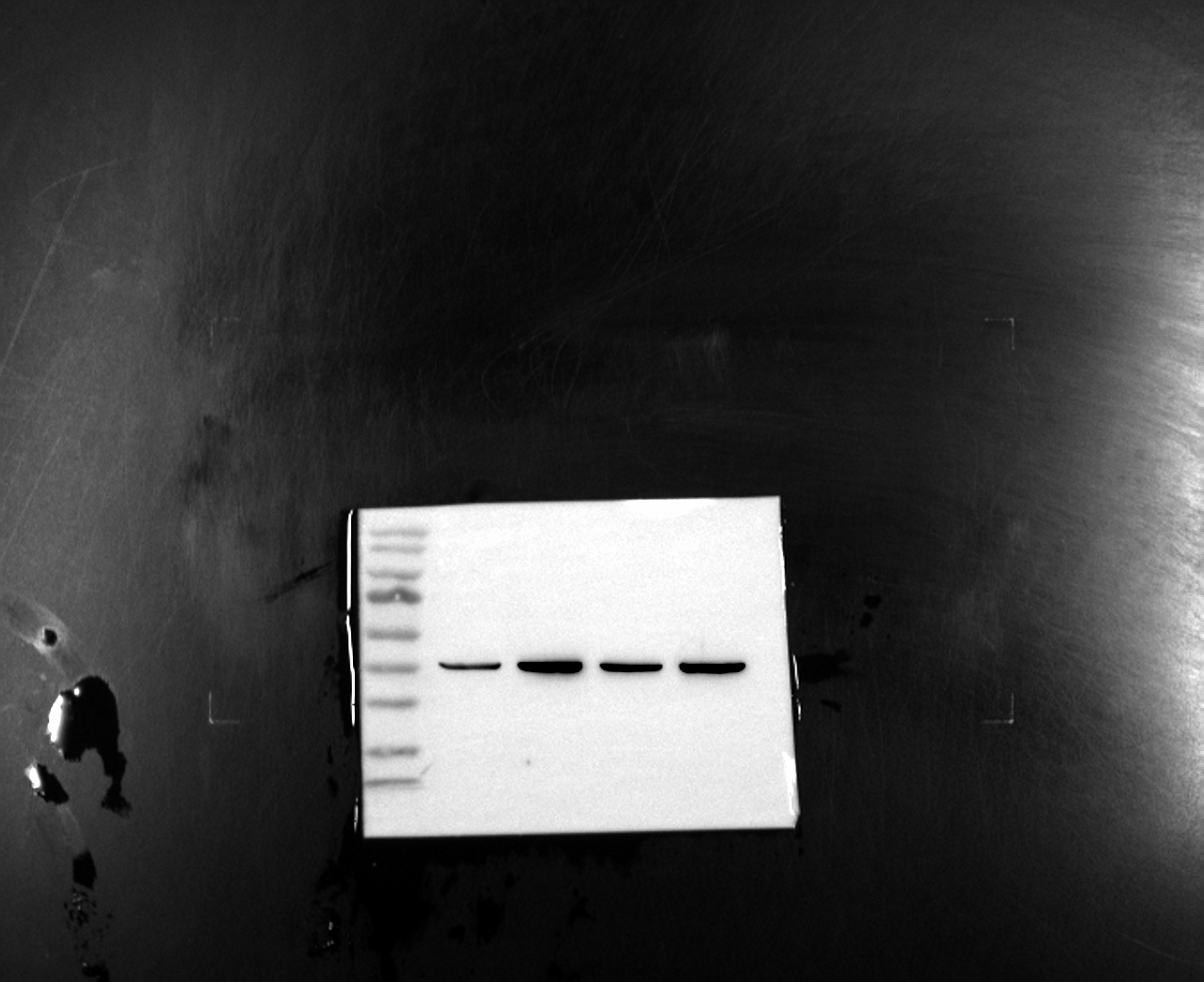

Supplement: S3 File — (ZIP) [file pone.0289818.s003.zip › S3 File. Fig3 Original data/image/3F/1.Caspase 1.tif]

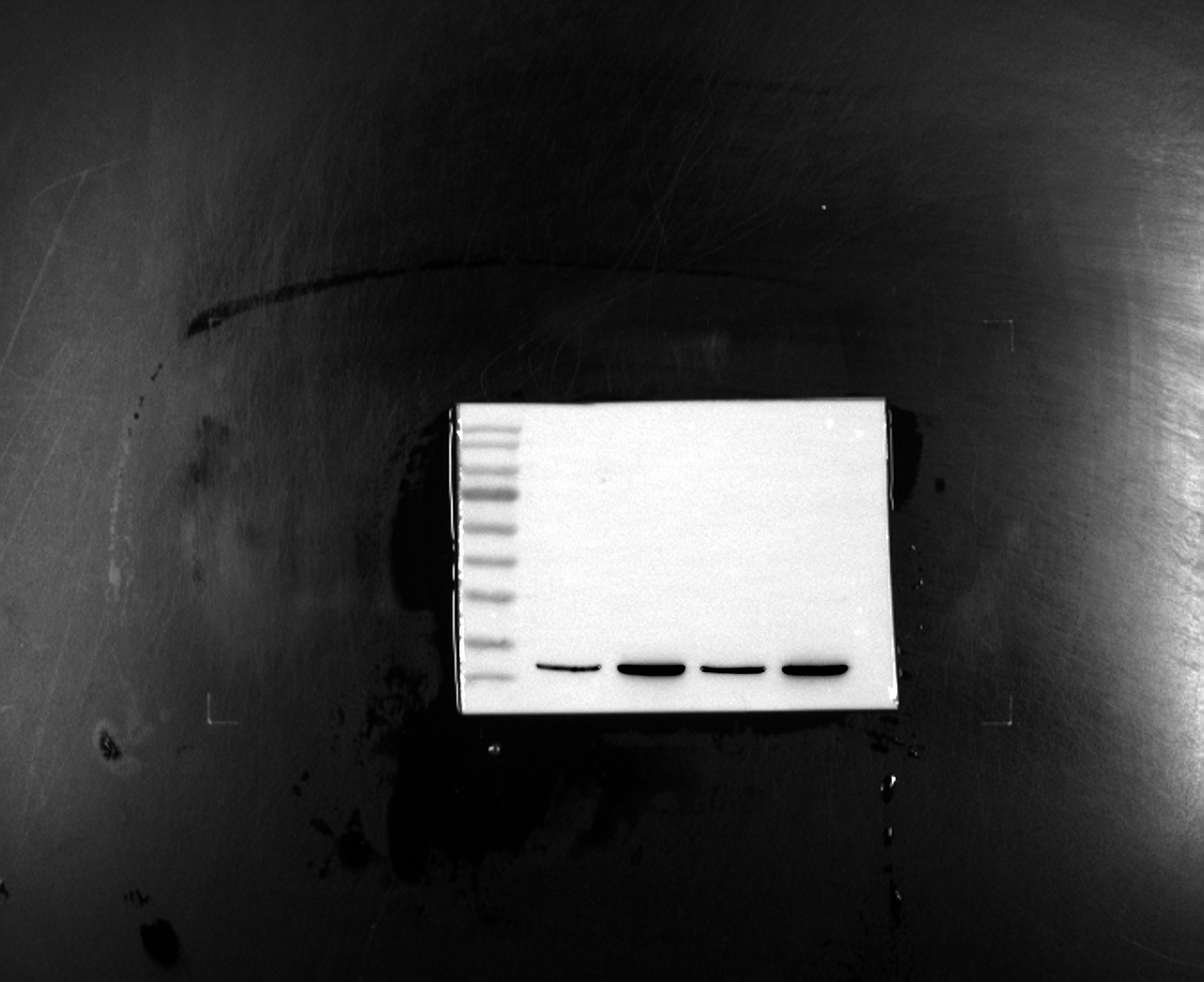

Supplement: S3 File — (ZIP) [file pone.0289818.s003.zip › S3 File. Fig3 Original data/image/3F/2.ASC.tif]

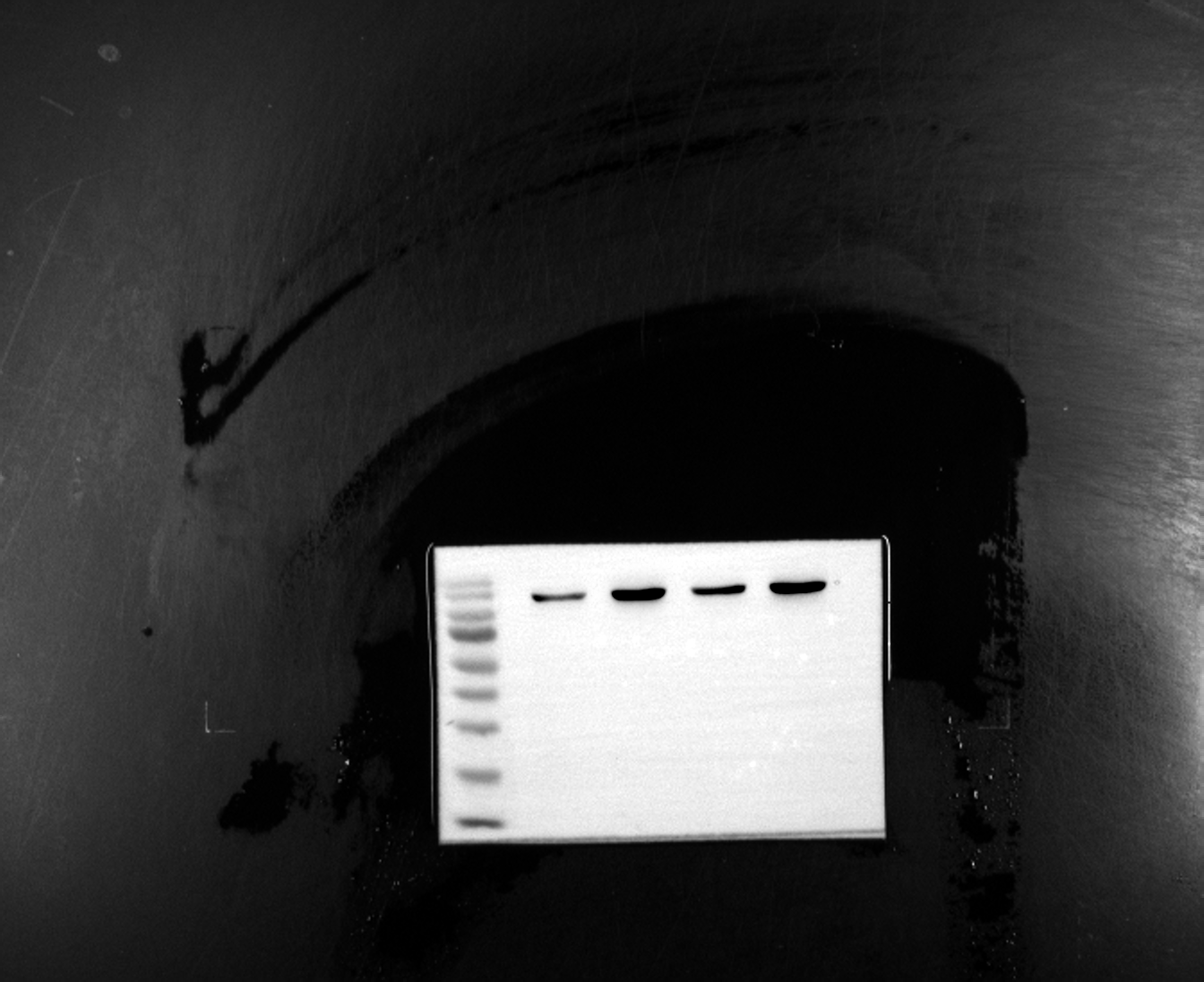

Supplement: S3 File — (ZIP) [file pone.0289818.s003.zip › S3 File. Fig3 Original data/image/3F/3.NLRP3.tif]

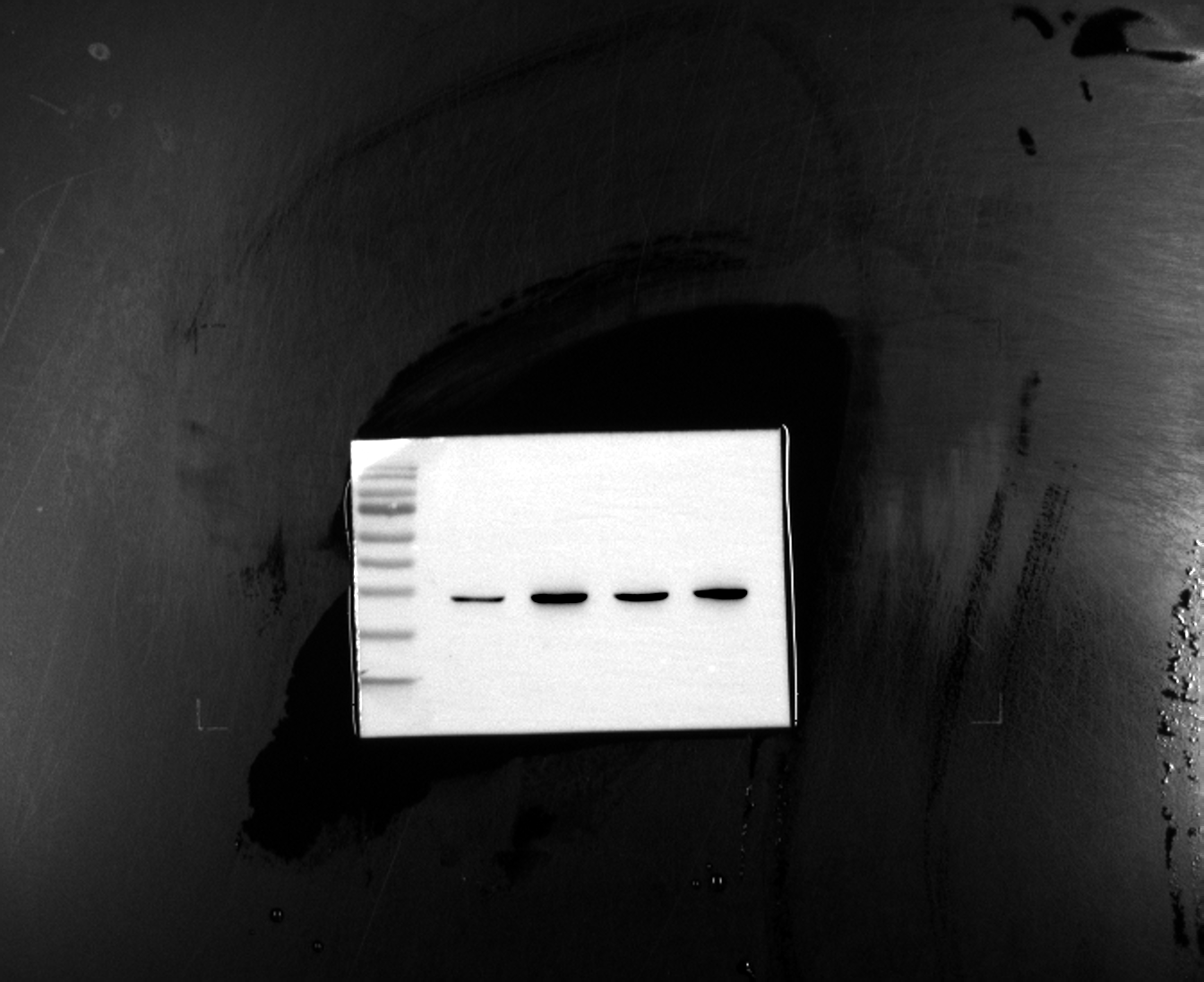

Supplement: S3 File — (ZIP) [file pone.0289818.s003.zip › S3 File. Fig3 Original data/image/3F/4.IL-1β.tif]

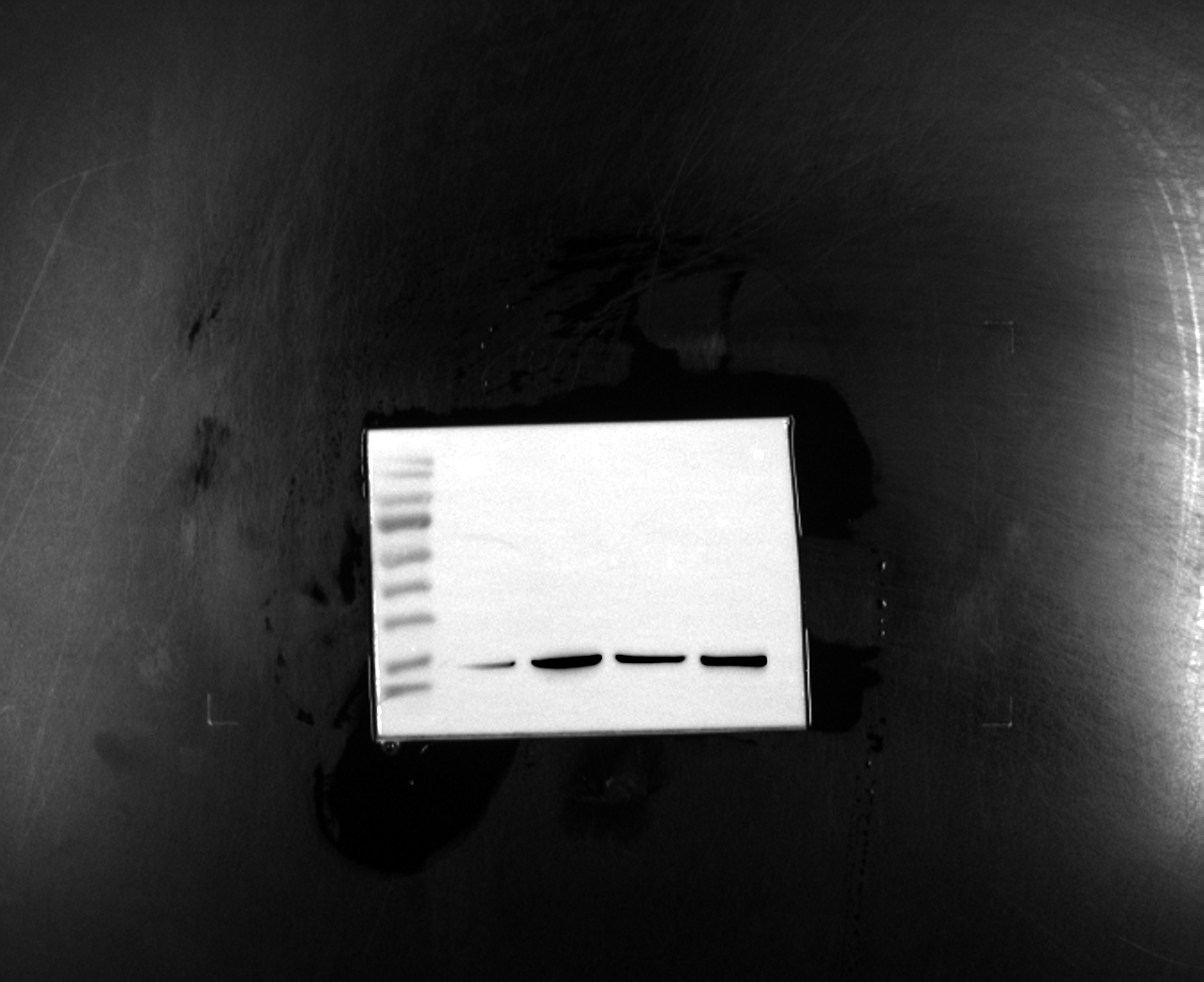

Supplement: S3 File — (ZIP) [file pone.0289818.s003.zip › S3 File. Fig3 Original data/image/3F/5.IL-18.tif]

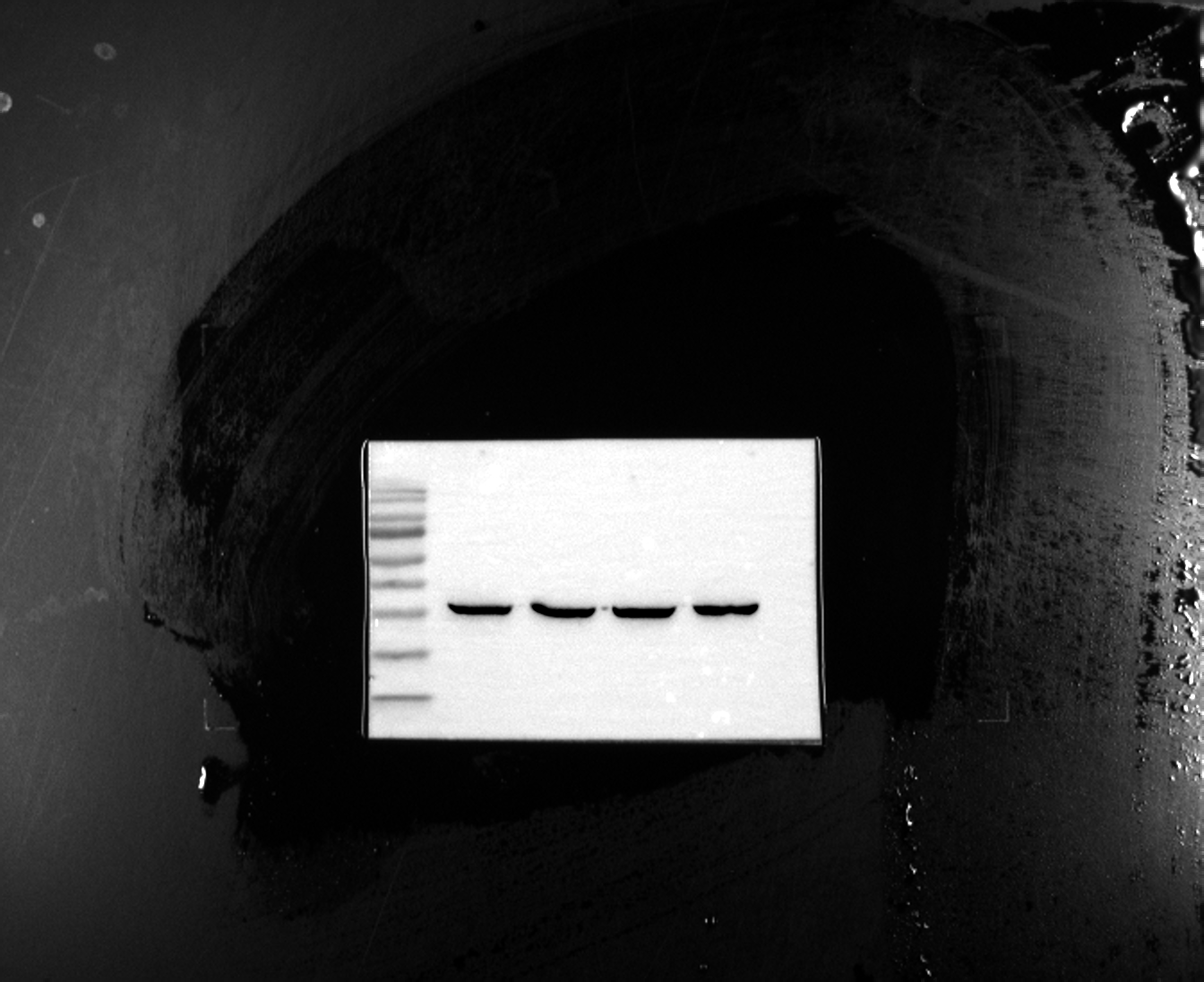

Supplement: S3 File — (ZIP) [file pone.0289818.s003.zip › S3 File. Fig3 Original data/image/3F/6.GAPDH.tif]

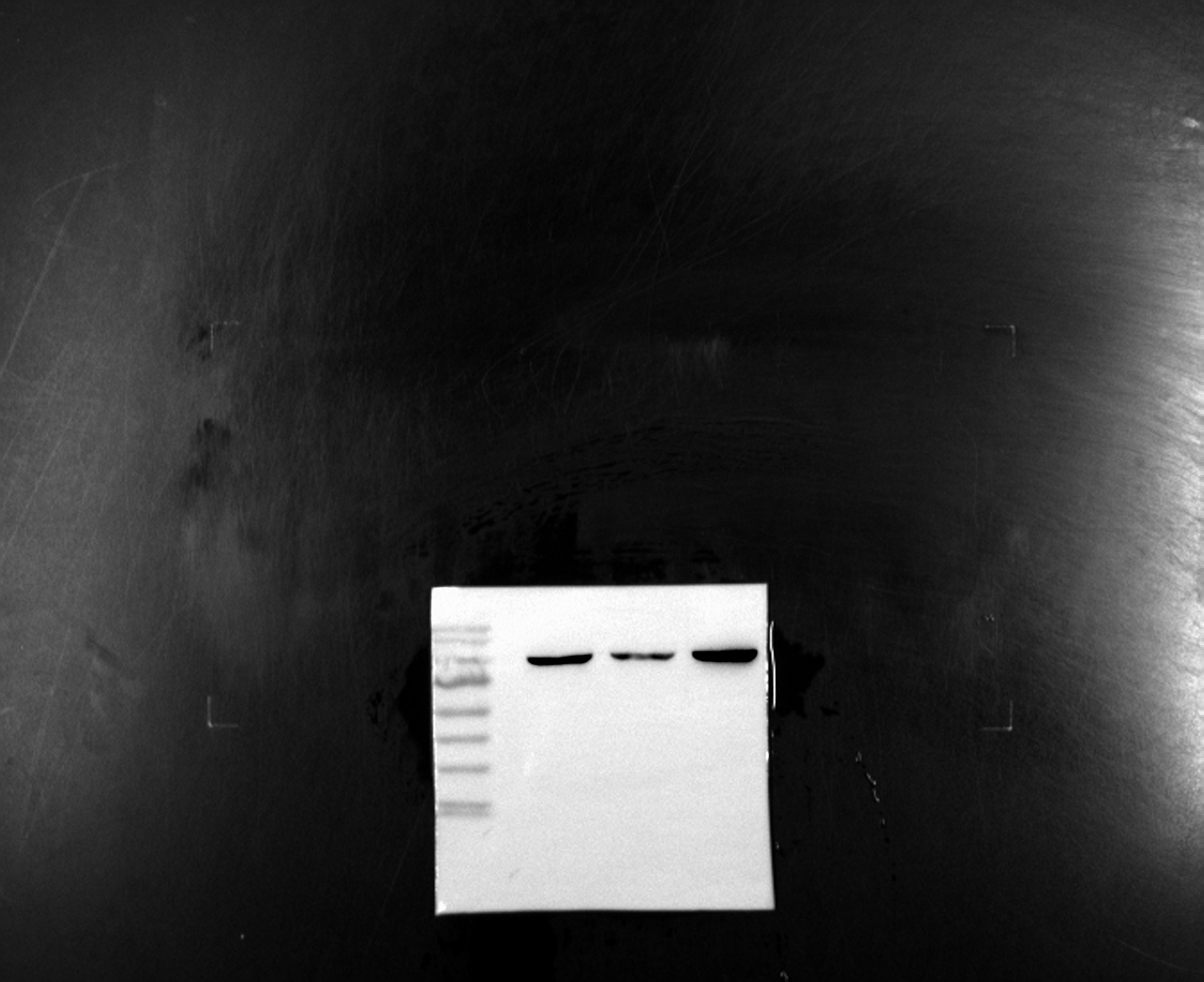

Supplement: S4 File — (ZIP) [file pone.0289818.s004.zip › S4 File. Fig4 Original data/image/4C/1-NLRP3.tif]

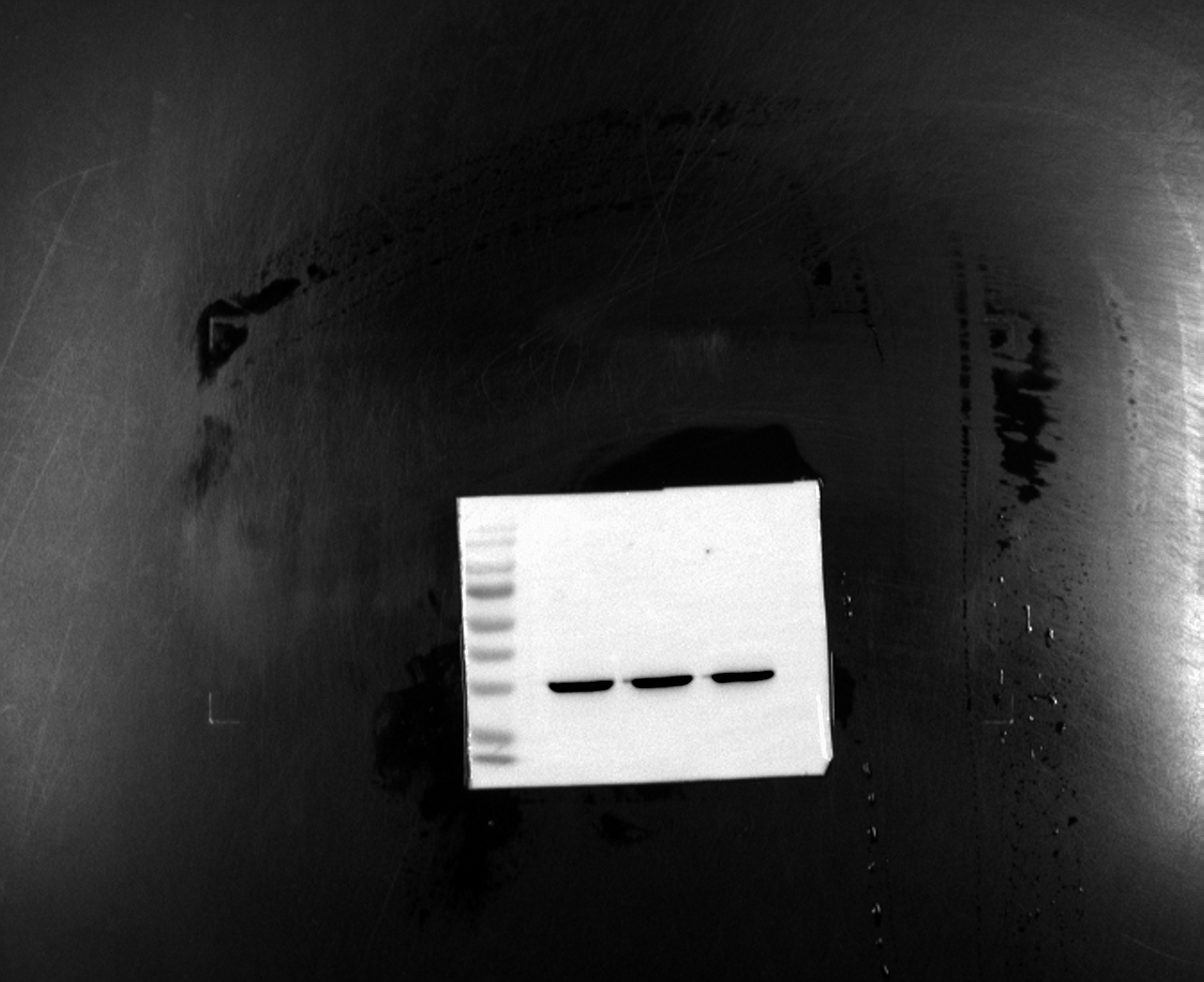

Supplement: S4 File — (ZIP) [file pone.0289818.s004.zip › S4 File. Fig4 Original data/image/4C/2-GAPDH.tif]

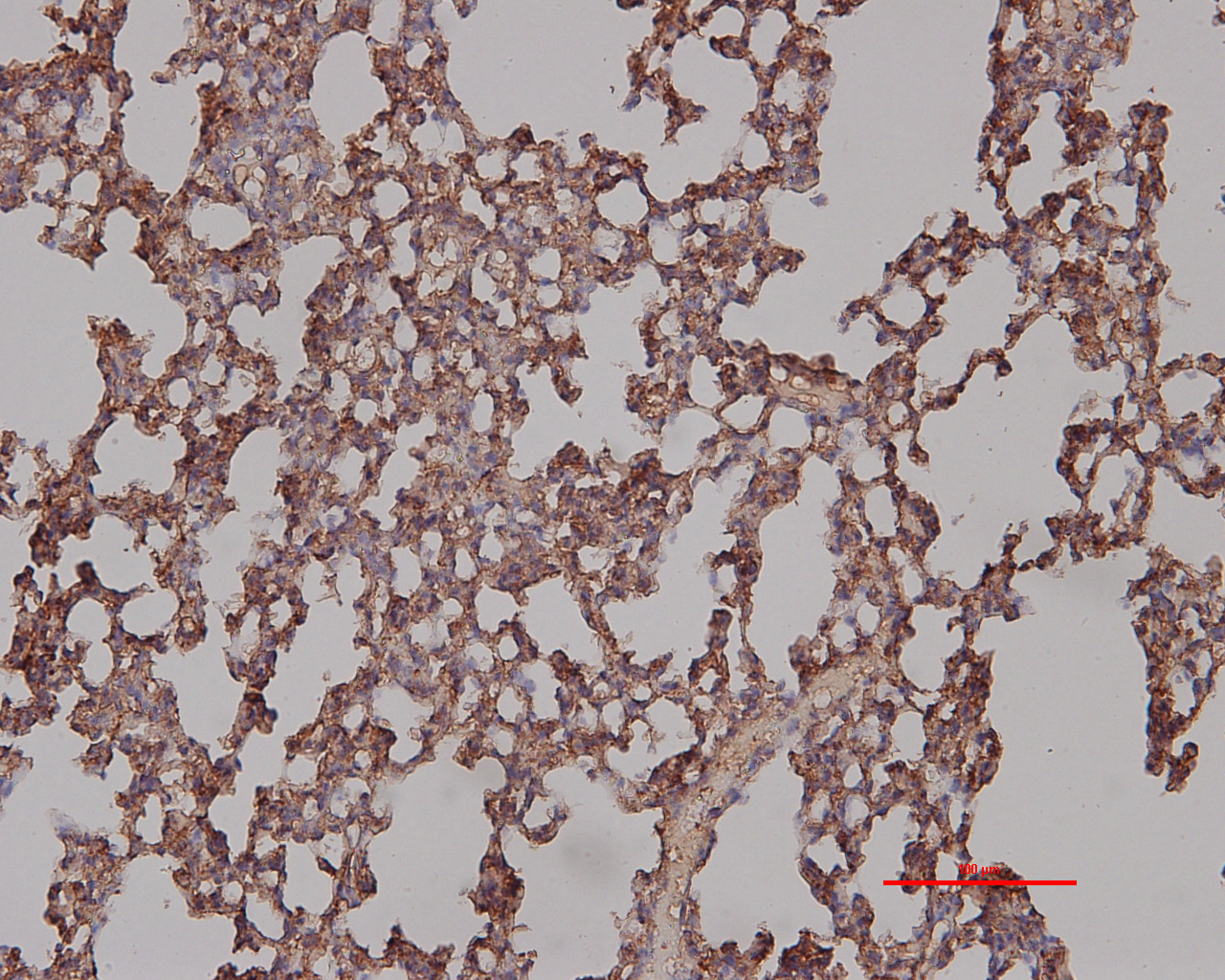

Supplement: S4 File — (ZIP) [file pone.0289818.s004.zip › S4 File. Fig4 Original data/image/4D/ALI.tif]

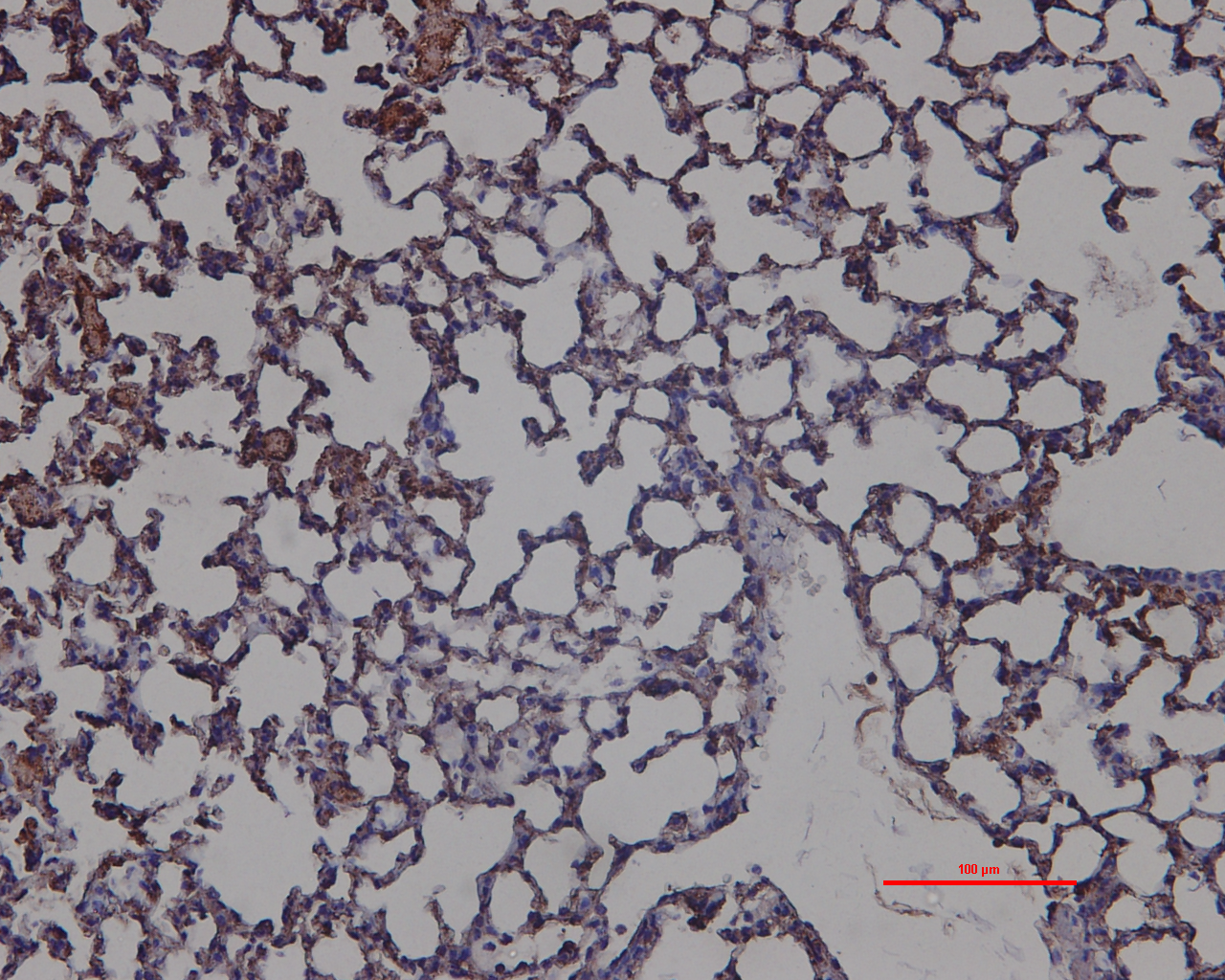

Supplement: S4 File — (ZIP) [file pone.0289818.s004.zip › S4 File. Fig4 Original data/image/4D/Bud+NAC.tif]

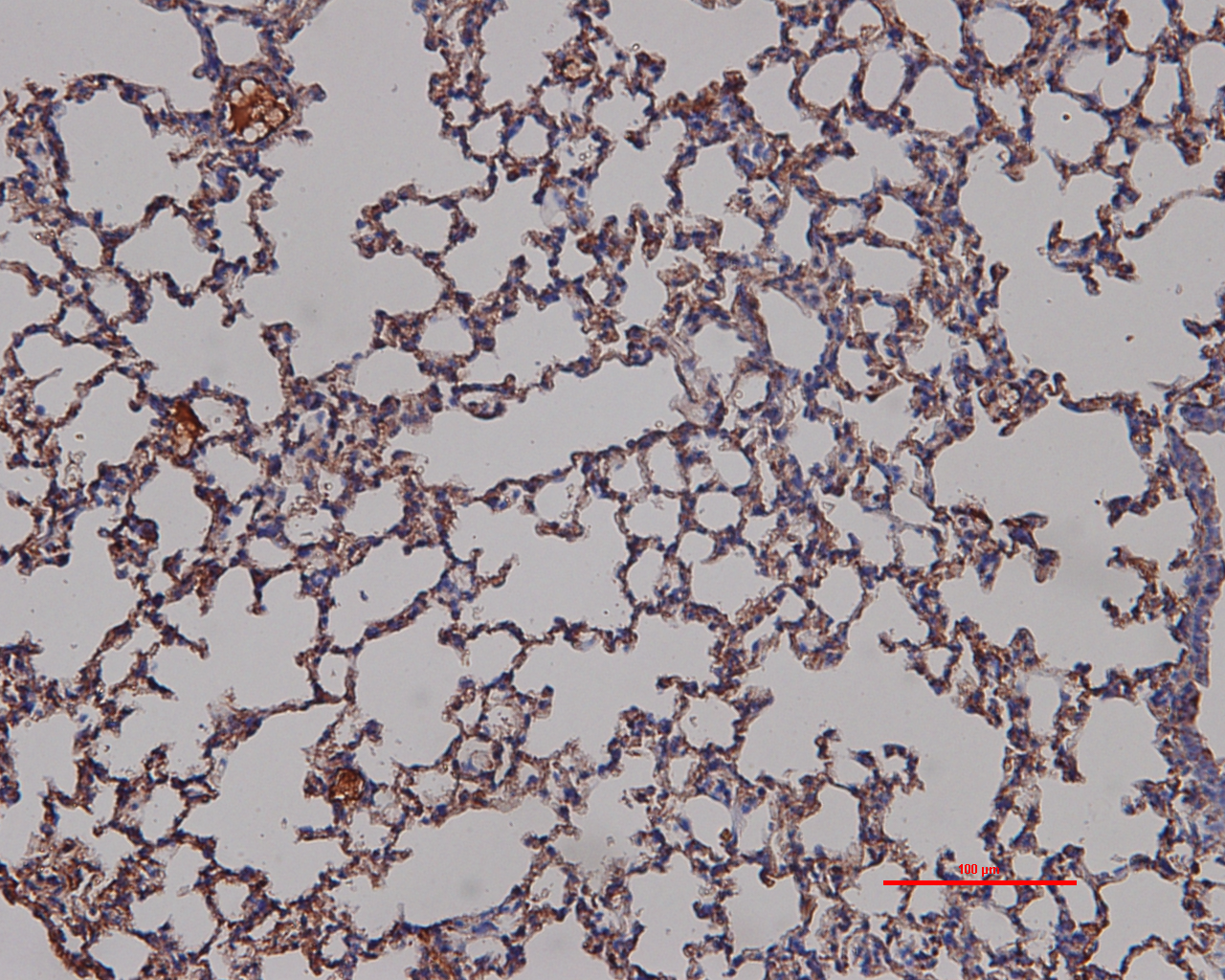

Supplement: S4 File — (ZIP) [file pone.0289818.s004.zip › S4 File. Fig4 Original data/image/4D/Bud.tif]

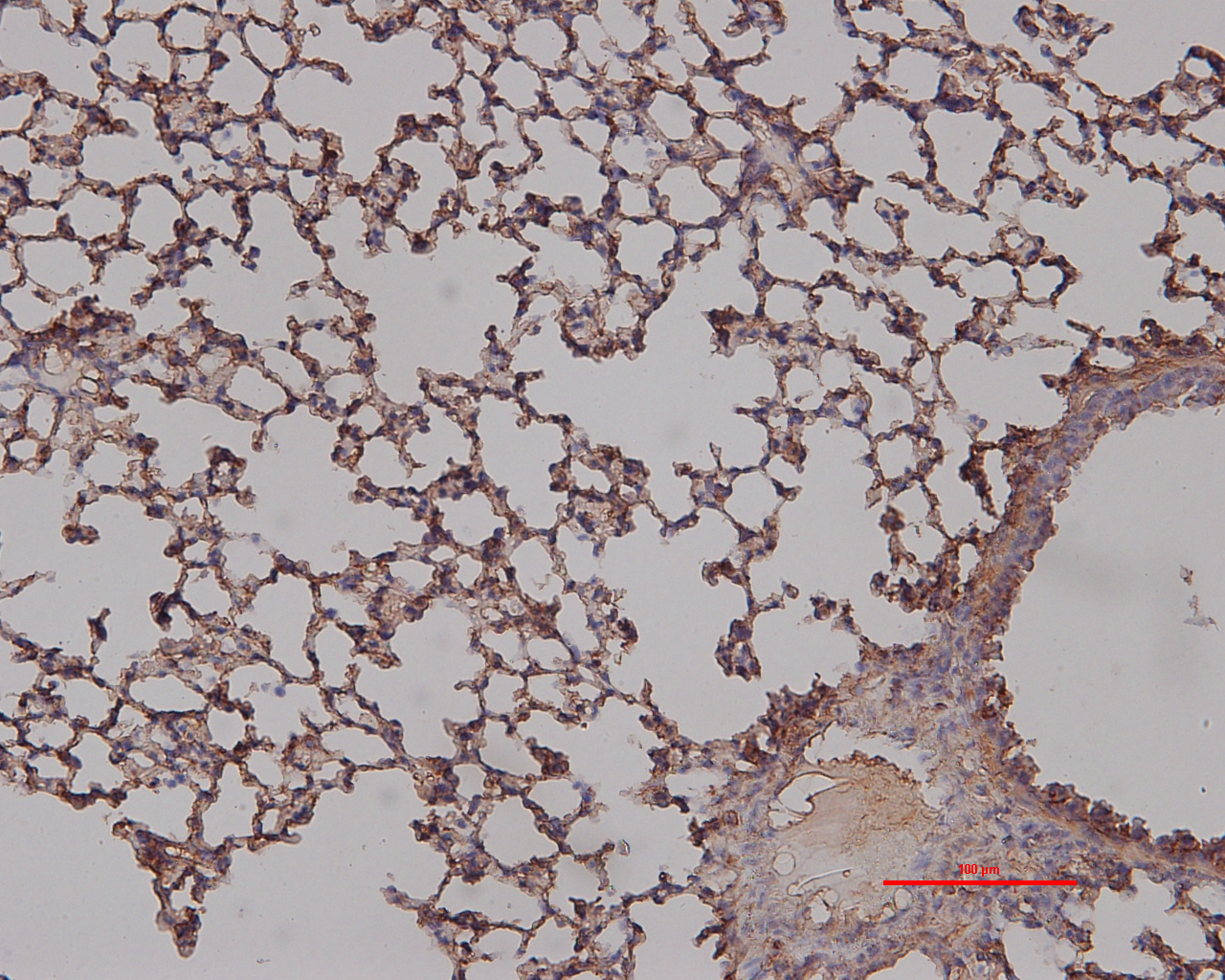

Supplement: S4 File — (ZIP) [file pone.0289818.s004.zip › S4 File. Fig4 Original data/image/4D/NAC.tif]

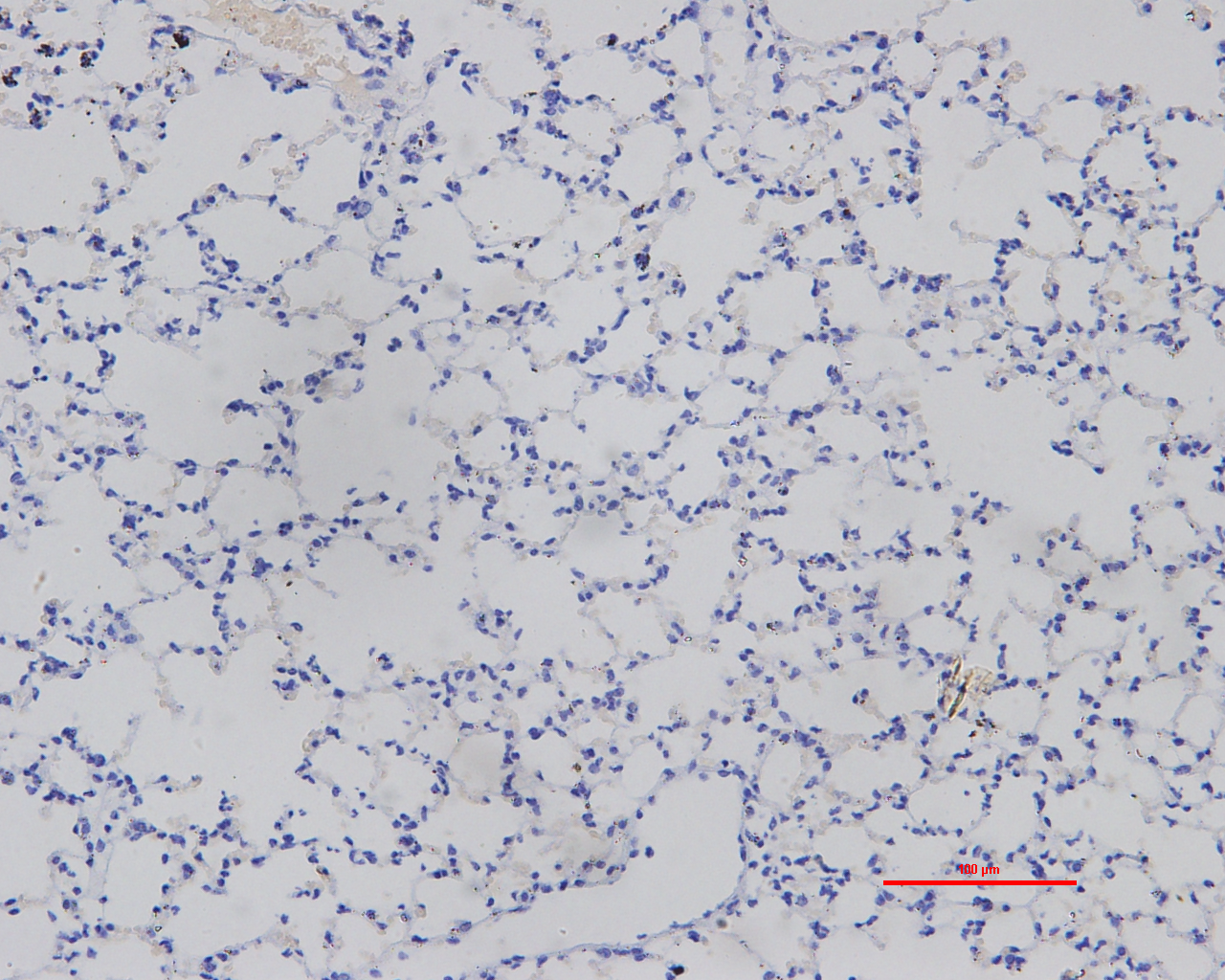

Supplement: S4 File — (ZIP) [file pone.0289818.s004.zip › S4 File. Fig4 Original data/image/4D/NC.tif]

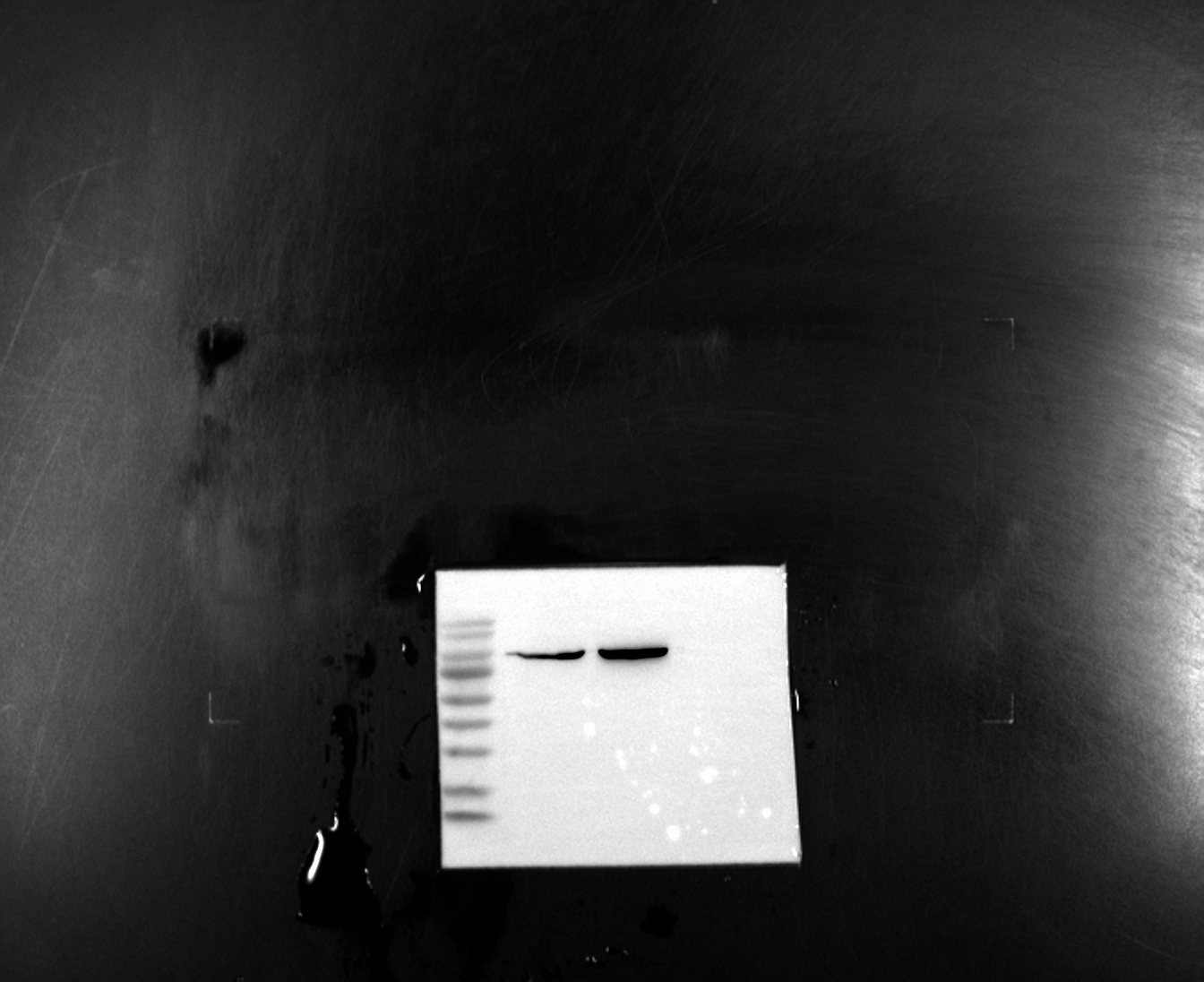

Supplement: S5 File — (ZIP) [file pone.0289818.s005.zip › S5 File. Fig5 Original data/image/5A/1.NLRP3.tif]

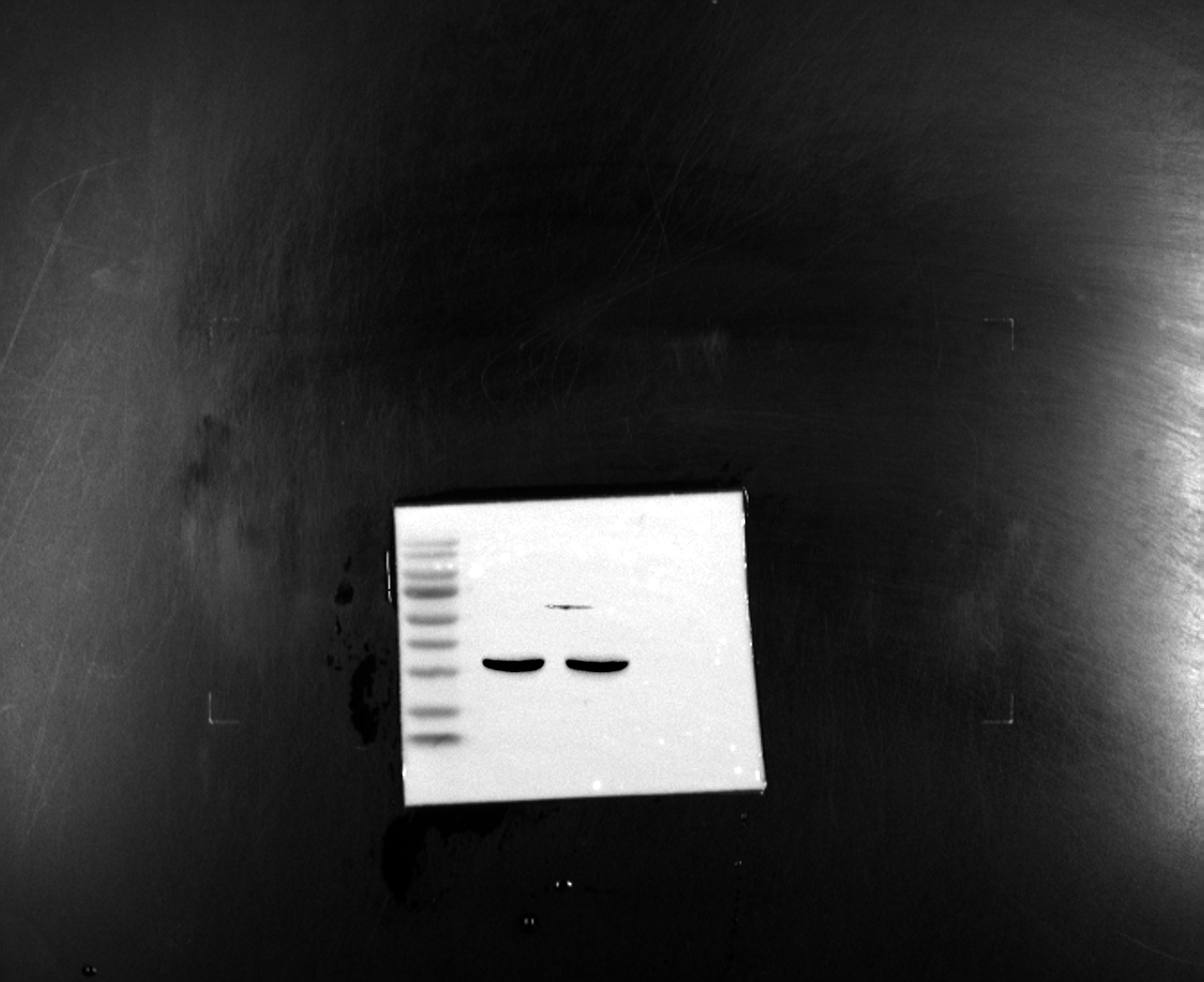

Supplement: S5 File — (ZIP) [file pone.0289818.s005.zip › S5 File. Fig5 Original data/image/5A/2.GAPDH.tif]

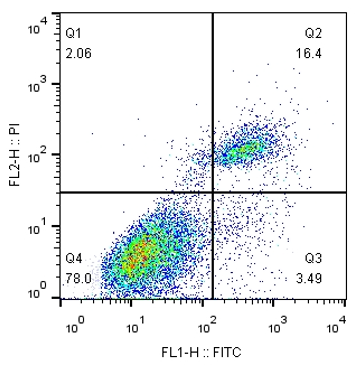

Supplement: S5 File — (ZIP) [file pone.0289818.s005.zip › S5 File. Fig5 Original data/image/5C/LPS/1 (1).jpg]

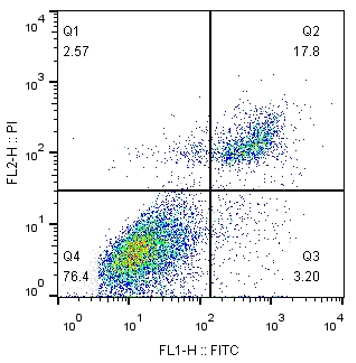

Supplement: S5 File — (ZIP) [file pone.0289818.s005.zip › S5 File. Fig5 Original data/image/5C/LPS/1 (2).jpg]

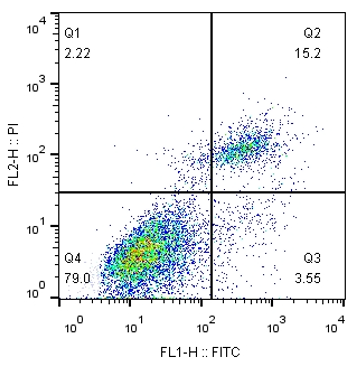

Supplement: S5 File — (ZIP) [file pone.0289818.s005.zip › S5 File. Fig5 Original data/image/5C/LPS/1 (3).jpg]

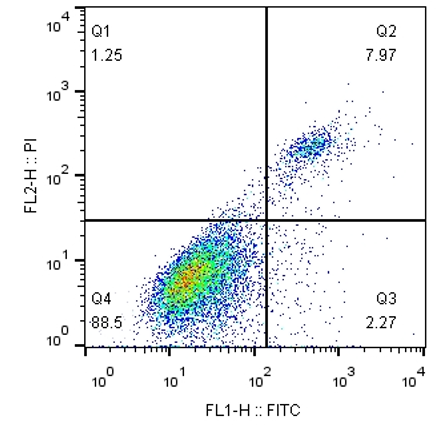

Supplement: S5 File — (ZIP) [file pone.0289818.s005.zip › S5 File. Fig5 Original data/image/5C/LPS+Bud+NAC/1 (1).jpg]

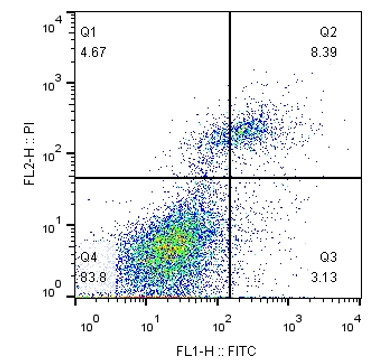

Supplement: S5 File — (ZIP) [file pone.0289818.s005.zip › S5 File. Fig5 Original data/image/5C/LPS+Bud+NAC/1 (2).jpg]

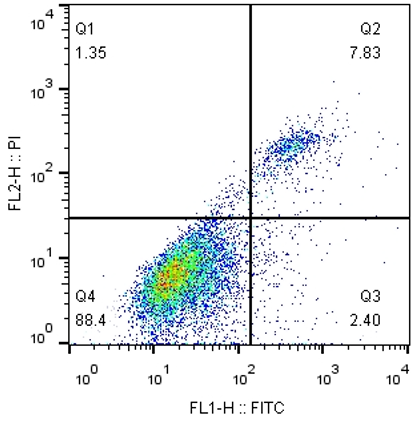

Supplement: S5 File — (ZIP) [file pone.0289818.s005.zip › S5 File. Fig5 Original data/image/5C/LPS+Bud+NAC/1 (3).jpg]

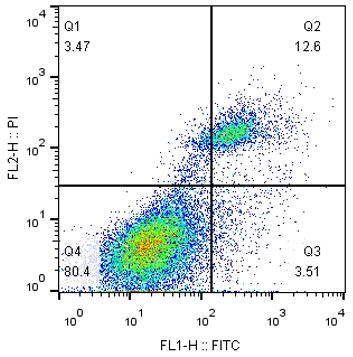

Supplement: S5 File — (ZIP) [file pone.0289818.s005.zip › S5 File. Fig5 Original data/image/5C/LPS+Bud+NAC+OE-NLRP3/1 (1).jpg]

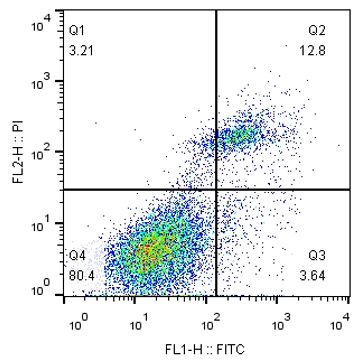

Supplement: S5 File — (ZIP) [file pone.0289818.s005.zip › S5 File. Fig5 Original data/image/5C/LPS+Bud+NAC+OE-NLRP3/1 (2).jpg]

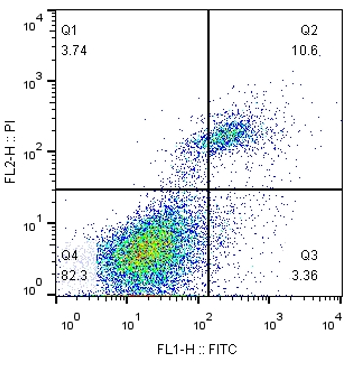

Supplement: S5 File — (ZIP) [file pone.0289818.s005.zip › S5 File. Fig5 Original data/image/5C/LPS+Bud+NAC+OE-NLRP3/1 (3).jpg]

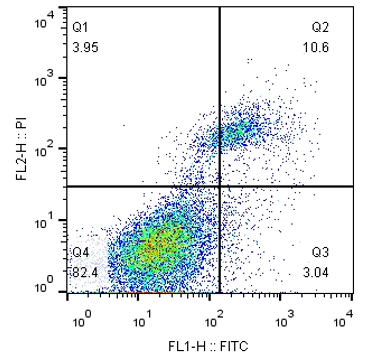

Supplement: S5 File — (ZIP) [file pone.0289818.s005.zip › S5 File. Fig5 Original data/image/5C/LPS+Bud+NAC+miR-381 inhi/1 (1).jpg]

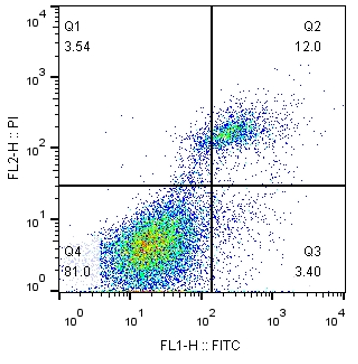

Supplement: S5 File — (ZIP) [file pone.0289818.s005.zip › S5 File. Fig5 Original data/image/5C/LPS+Bud+NAC+miR-381 inhi/1 (2).jpg]
