# Supplementary material for: Combination therapy with budesonide and acetylcysteine alleviates LPS-induced acute lung injury via the miR-381/NLRP3 molecular axis
Source: PLoS One. 2023 Aug 9;18(8):e0289818. doi: 10.1371/journal.pone.0289818 (PMC10411794; doi:10.1371/journal.pone.0289818)
Supplement: S3 File — (ZIP) [file pone.0289818.s003.zip › S3 File. Fig3 Original data/date/3A/Results_Report_2023-05-16-11742.pdf]

Plate Results Report

A229-2.eds

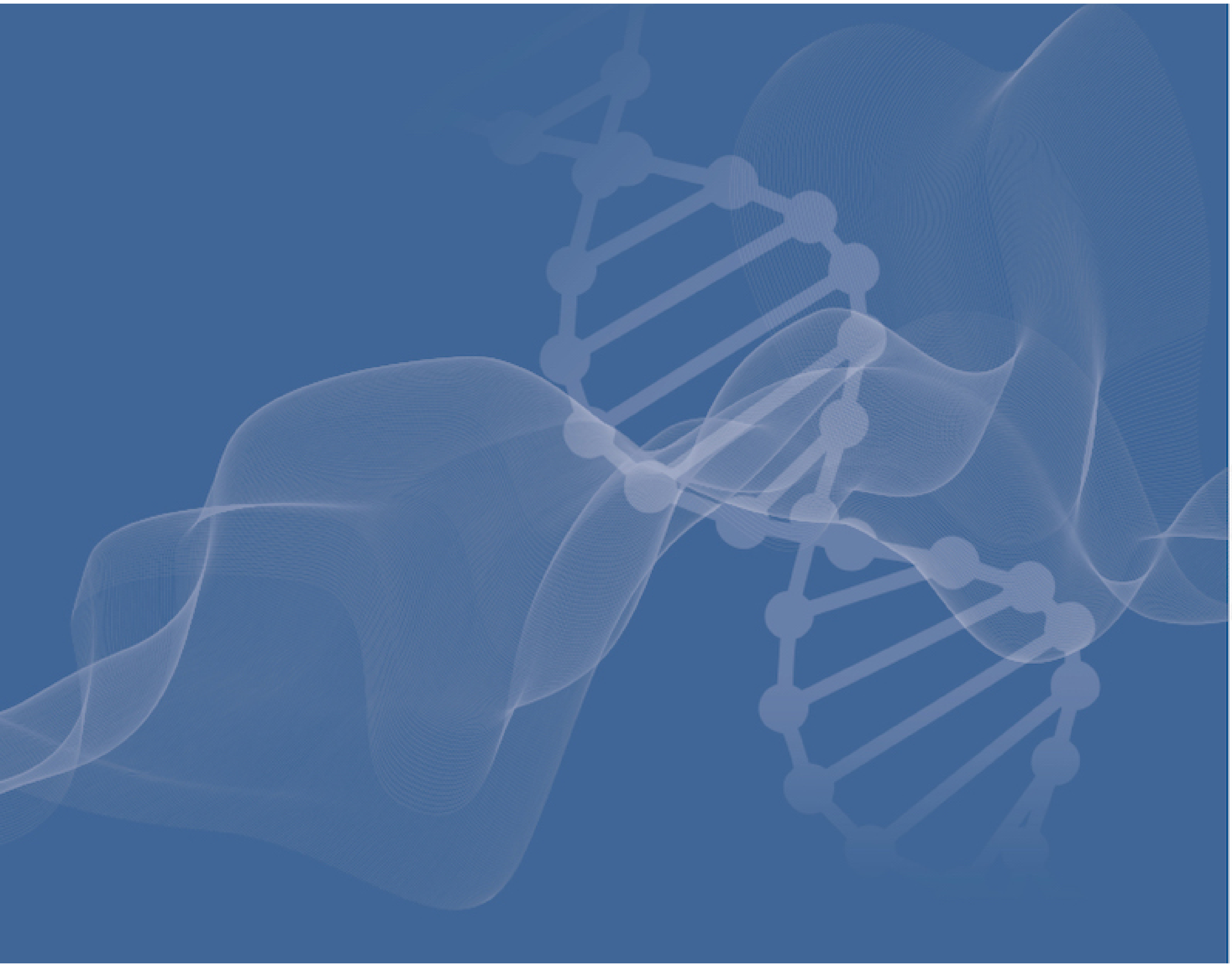

Summary

Property	Details
Bar Code	-
File Name	A229-2.eds
Run Start Date/Time	Mar 23, 2022 3:21:21 PM
Run End Date/Time	Mar 23, 2022 4:17:16 PM
Run Duration	55 minutes, and 55 seconds
Operator	DEFAULT
Instrument Name	SVT004
Instrument Type	QuantStudio™ 3 System
Instrument Serial Number	SVT004
Block Type	96-Well 0.2-mL
Block Serial Number	41145627
Heated Cover Serial Number	N/A
PCR Stage/Step Number	Stage 2, Step 2
Melt Stage Number	Stage 3
Quantification Cycle Method	Baseline Threshold
Comment	-
Software Name and Version	Design & Analysis Software v2.6.0
Plugin Name and Version	Primary Analysis v1.7.0, Relative Quantification v1.5.0
Analysis Date/Time	May 16, 2023 11:07:34 AM

Well Table

Well	Sample	Target	Task	Cq	Cq Confi dence	Amp Score	Amp Status	Cq Thres hold	Baseline Start/End	Melt Temp
A1	NC -1	U6	Unknown	16.939	0.809	2.061	AMP	3.129	3-13	83.4
A2	NC -1	U6	Unknown	16.46	0.772	2.116	AMP	3.129	3-11	83.251
A3	NC -1	U6	Unknown	16.928	0.716	2.104	AMP	3.129	3-13	83.548
A4	NC -1	miR-381	Unknown	21.025	0.991	1.912	AMP	1.942	3-13	82.654
A5	NC -1	miR-381	Unknown	21.111	0.988	1.912	AMP	1.942	3-13	82.504
A6	NC -1	miR-381	Unknown	21.043	0.988	1.913	AMP	1.942	3-13	82.653
A7	LPS+Bud-3	U6	Unknown	17.089	0.902	2.134	AMP	3.129	3-13	83.398
A8	LPS+Bud-3	U6	Unknown	16.943	0.731	2.125	AMP	3.129	3-13	83.398
A9	LPS+Bud-3	U6	Unknown	16.519	0.937	2.078	AMP	3.129	3-12	83.248
A10	LPS+Bud-3	miR-381	Unknown	23.827	0.985	1.895	AMP	1.942	3-15	82.354
A11	LPS+Bud-3	miR-381	Unknown	23.687	0.993	1.903	AMP	1.942	3-15	82.953
A12	LPS+Bud-3	miR-381	Unknown	23.934	0.969	1.846	AMP	1.942	3-17	82.953
B1	NC -2	U6	Unknown	16.684	0.834	2.127	AMP	3.129	3-13	83.251
B2	NC -2	U6	Unknown	16.747	0.657	2.128	AMP	3.129	3-13	83.251
B3	NC -2	U6	Unknown	16.972	0.814	2.137	AMP	3.129	3-14	83.399
B4	NC -2	miR-381	Unknown	20.702	0.991	1.929	AMP	1.942	3-13	82.654
B5	NC -2	miR-381	Unknown	20.847	0.984	1.915	AMP	1.942	3-14	82.653
B6	NC -2	miR-381	Unknown	20.69	0.993	1.922	AMP	1.942	3-12	82.653
B7	LPS+NAC-1	U6	Unknown	16.907	0.677	2.118	AMP	3.129	3-12	83.398
B8	LPS+NAC-1	U6	Unknown	16.852	0.905	2.113	AMP	3.129	3-13	83.249
B9	LPS+NAC-1	U6	Unknown	16.762	0.688	2.145	AMP	3.129	3-13	83.248
B10	LPS+NAC-1	miR-381	Unknown	23.532	0.99	1.906	AMP	1.942	3-16	82.354
B11	LPS+NAC-1	miR-381	Unknown	23.547	0.993	1.906	AMP	1.942	3-15	82.804
B12	LPS+NAC-1	miR-381	Unknown	23.769	0.993	1.891	AMP	1.942	3-14	82.804
C1	NC -3	U6	Unknown	16.671	0.808	2.174	AMP	3.129	3-11	83.102
C2	NC -3	U6	Unknown	16.797	0.806	2.16	AMP	3.129	3-12	83.102
C3	NC -3	U6	Unknown	16.792	0.778	2.138	AMP	3.129	3-13	83.25
C4	NC -3	miR-381	Unknown	20.92	0.994	1.913	AMP	1.942	3-13	82.654
C5	NC -3	miR-381	Unknown	20.815	0.99	1.915	AMP	1.942	3-13	82.653
C6	NC -3	miR-381	Unknown	20.99	0.964	1.881	AMP	1.942	3-14	82.504

Well	Sample	Target	Task	Cq	Cq Confidence	Amp Score	Amp Status	Cq Threshold	Baseline Start/End	Melt Temp
C7	LPS+NAC-2	U6	Unknown	17.355	0.846	2.097	AMP	3.129	3-13	81.907
C8	LPS+NAC-2	U6	Unknown	16.769	0.634	2.126	AMP	3.129	3-13	83.398
C9	LPS+NAC-2	U6	Unknown	16.954	0.81	2.158	AMP	3.129	3-12	83.248
C10	LPS+NAC-2	miR-381	Unknown	23.373	0.991	1.904	AMP	1.942	3-14	82.354
C11	LPS+NAC-2	miR-381	Unknown	23.444	0.992	1.905	AMP	1.942	3-15	82.655
C12	LPS+NAC-2	miR-381	Unknown	23.576	0.993	1.9	AMP	1.942	3-14	82.804
D1	LPS-1	U6	Unknown	16.819	0.85	2.181	AMP	3.129	3-14	83.102
D2	LPS-1	U6	Unknown	16.989	0.689	2.166	AMP	3.129	3-14	82.953
D3	LPS-1	U6	Unknown	16.919	0.743	2.192	AMP	3.129	3-12	83.25
D4	LPS-1	miR-381	Unknown	26.381	0.992	1.913	AMP	1.942	3-15	82.355
D5	LPS-1	miR-381	Unknown	26.209	0.99	1.918	AMP	1.942	3-16	82.504
D6	LPS-1	miR-381	Unknown	26.454	0.991	1.909	AMP	1.942	3-17	82.504
D7	LPS+NAC-3	U6	Unknown	16.68	0.689	2.175	AMP	3.129	3-14	83.398
D8	LPS+NAC-3	U6	Unknown	16.605	0.618	2.163	AMP	3.129	3-14	82.056
D9	LPS+NAC-3	U6	Unknown	Undetermined	-	2.165	Inconclusive	3.129	3-39	83.248
D10	LPS+NAC-3	miR-381	Unknown	23.319	0.989	1.907	AMP	1.942	3-15	82.354
D11	LPS+NAC-3	miR-381	Unknown	23.216	0.992	1.906	AMP	1.942	3-15	82.655
D12	LPS+NAC-3	miR-381	Unknown	23.316	0.987	1.907	AMP	1.942	3-16	82.655
E1	LPS-2	U6	Unknown	17.217	0.815	2.138	AMP	3.129	3-13	82.953
E2	LPS-2	U6	Unknown	17.043	0.855	2.172	AMP	3.129	3-13	82.953
E3	LPS-2	U6	Unknown	17.137	0.882	2.133	AMP	3.129	3-13	83.25
E4	LPS-2	miR-381	Unknown	26.623	0.993	1.905	AMP	1.942	3-17	82.355
E5	LPS-2	miR-381	Unknown	26.58	0.989	1.918	AMP	1.942	3-17	82.355
E6	LPS-2	miR-381	Unknown	26.651	0.991	1.907	AMP	1.942	3-17	82.504
E7	LPS+Bud+NAC-1	U6	Unknown	17.176	0.776	2.076	AMP	3.129	3-12	82.503
E8	LPS+Bud+NAC-1	U6	Unknown	17.285	0.894	2.124	AMP	3.129	3-13	82.354
E9	LPS+Bud+NAC-1	U6	Unknown	17.169	0.643	2.101	AMP	3.129	3-13	83.248
E10	LPS+Bud+NAC-1	miR-381	Unknown	21.117	0.969	1.878	AMP	1.942	3-16	82.354
E11	LPS+Bud+NAC-1	miR-381	Unknown	21.012	0.989	1.885	AMP	1.942	3-14	82.505
E12	LPS+Bud+NAC-1	miR-381	Unknown	21.056	0.989	1.882	AMP	1.942	3-13	82.655

Well	Sample	Target	Task	Cq	Cq Confidence	Amp Score	Amp Status	Cq Threshold	Baseline Start/End	Melt Temp
F1	LPS-3	U6	Unknown	16.681	0.758	2.151	AMP	3.129	3-11	82.953
F2	LPS-3	U6	Unknown	17.207	0.802	2.204	AMP	3.129	3-13	82.804
F3	LPS-3	U6	Unknown	17.11	0.773	2.153	AMP	3.129	3-13	83.25
F4	LPS-3	miR-381	Unknown	26.666	0.993	1.911	AMP	1.942	3-17	82.206
F5	LPS-3	miR-381	Unknown	26.607	0.99	1.906	AMP	1.942	3-16	82.355
F6	LPS-3	miR-381	Unknown	26.715	0.992	1.91	AMP	1.942	3-16	82.355
F7	LPS+Bud+NAC-2	U6	Unknown	16.974	0.767	2.137	AMP	3.129	3-14	83.398
F8	LPS+Bud+NAC-2	U6	Unknown	16.884	0.742	2.086	AMP	3.129	3-13	83.398
F9	LPS+Bud+NAC-2	U6	Unknown	16.797	0.68	2.081	AMP	3.129	3-12	83.248
F10	LPS+Bud+NAC-2	miR-381	Unknown	20.87	0.991	1.911	AMP	1.942	3-11	82.354
F11	LPS+Bud+NAC-2	miR-381	Unknown	21.12	0.989	1.89	AMP	1.942	3-14	82.505
F12	LPS+Bud+NAC-2	miR-381	Unknown	21.041	0.989	1.893	AMP	1.942	3-13	82.505
G1	LPS+Bud-1	U6	Unknown	17.261	0.85	2.164	AMP	3.129	3-14	82.953
G2	LPS+Bud-1	U6	Unknown	17.247	0.796	2.115	AMP	3.129	3-14	82.953
G3	LPS+Bud-1	U6	Unknown	17.017	0.786	2.162	AMP	3.129	3-12	83.25
G4	LPS+Bud-1	miR-381	Unknown	23.69	0.994	1.913	AMP	1.942	3-16	82.355
G5	LPS+Bud-1	miR-381	Unknown	23.894	0.994	1.902	AMP	1.942	3-15	82.355
G6	LPS+Bud-1	miR-381	Unknown	23.898	0.995	1.902	AMP	1.942	3-14	82.355
G7	LPS+Bud+NAC-3	U6	Unknown	16.502	0.619	2.154	AMP	3.129	3-12	82.056
G8	LPS+Bud+NAC-3	U6	Unknown	16.98	0.817	2.032	AMP	3.129	3-13	83.398
G9	LPS+Bud+NAC-3	U6	Unknown	16.02	0.653	2.14	AMP	3.129	3-13	83.397
G10	LPS+Bud+NAC-3	miR-381	Unknown	21.23	0.984	1.899	AMP	1.942	3-14	82.205
G11	LPS+Bud+NAC-3	miR-381	Unknown	21.303	0.983	1.804	AMP	1.942	3-11	82.356
G12	LPS+Bud+NAC-3	miR-381	Unknown	21.19	0.991	1.895	AMP	1.942	3-14	82.505
H1	LPS+Bud-3	U6	Unknown	17.85	0.868	2.059	AMP	3.129	3-13	82.953
H2	LPS+Bud-3	U6	Unknown	16.498	0.79	2.196	AMP	3.129	3-14	82.804
H3	LPS+Bud-3	U6	Unknown	17.129	0.861	2.149	AMP	3.129	3-13	83.25
H4	LPS+Bud-3	miR-381	Unknown	23.681	0.992	1.917	AMP	1.942	3-16	82.355
H5	LPS+Bud-3	miR-381	Unknown	23.746	0.993	1.91	AMP	1.942	3-16	82.504
H6	LPS+Bud-3	miR-381	Unknown	24.067	0.99	1.889	AMP	1.942	3-15	82.355

Replicate Group Table

Sample	Target	No. of Replicates	Cq Mean	Cq SD
LPS+Bud+NAC-1	U6	3	17.21	0.065
LPS+Bud+NAC-1	miR-381	3	21.062	0.053
LPS+Bud+NAC-2	U6	3	16.885	0.088
LPS+Bud+NAC-2	miR-381	3	21.01	0.128
LPS+Bud+NAC-3	U6	3	16.501	0.48
LPS+Bud+NAC-3	miR-381	3	21.241	0.057
LPS+Bud-1	U6	3	17.175	0.137
LPS+Bud-1	miR-381	3	23.827	0.119
LPS+Bud-3	U6	6	17.005	0.497
LPS+Bud-3	miR-381	6	23.824	0.153
LPS+NAC-1	U6	3	16.84	0.073
LPS+NAC-1	miR-381	3	23.616	0.133
LPS+NAC-2	U6	3	17.026	0.3
LPS+NAC-2	miR-381	3	23.464	0.103
LPS+NAC-3	U6	3	16.643	0.053
LPS+NAC-3	miR-381	3	23.284	0.058
LPS-1	U6	3	16.909	0.085
LPS-1	miR-381	3	26.348	0.125
LPS-2	U6	3	17.132	0.087
LPS-2	miR-381	3	26.618	0.036
LPS-3	U6	3	17	0.28
LPS-3	miR-381	3	26.663	0.054
NC -1	U6	3	16.776	0.273
NC -1	miR-381	3	21.06	0.046
NC -2	U6	3	16.801	0.151
NC -2	miR-381	3	20.746	0.087
NC -3	U6	3	16.753	0.072
NC -3	miR-381	3	20.908	0.088

Plate Layout

	1	2	3	4	5	6	7	8	9	10	11	12
A	NC -1<br>U6 (16.939)	NC -1<br>U6 (16.46)	NC -1<br>U6 (16.928)	NC -1<br>miR-381 (21.025)	NC -1<br>miR-381 (21.111)	NC -1<br>miR-381 (21.043)	LPS+Bud-3<br>U6 (17.089)	LPS+Bud-3<br>U6 (16.943)	LPS+Bud-3<br>U6 (16.519)	LPS+Bud-3<br>miR-381 (23.827)	LPS+Bud-3<br>miR-381 (23.687)	LPS+Bud-3<br>miR-381 (23.934)
B	NC -2<br>U6 (16.684)	NC -2<br>U6 (16.747)	NC -2<br>U6 (16.972)	NC -2<br>miR-381 (20.702)	NC -2<br>miR-381 (20.847)	NC -2<br>miR-381 (20.69)	LPS+NAC-1<br>U6 (16.907)	LPS+NAC-1<br>U6 (16.852)	LPS+NAC-1<br>U6 (16.762)	LPS+NAC-1<br>miR-381 (23.532)	LPS+NAC-1<br>miR-381 (23.547)	LPS+NAC-1<br>miR-381 (23.769)
C	NC -3<br>U6 (16.671)	NC -3<br>U6 (16.797)	NC -3<br>U6 (16.792)	NC -3<br>miR-381 (20.92)	NC -3<br>miR-381 (20.815)	NC -3<br>miR-381 (20.99)	LPS+NAC-2<br>U6 (17.355)	LPS+NAC-2<br>U6 (16.769)	LPS+NAC-2<br>U6 (16.954)	LPS+NAC-2<br>miR-381 (23.373)	LPS+NAC-2<br>miR-381 (23.444)	LPS+NAC-2<br>miR-381 (23.576)
D	LPS-1<br>U6 (16.819)	LPS-1<br>U6 (16.989)	LPS-1<br>U6 (16.919)	LPS-1<br>miR-381 (26.381)	LPS-1<br>miR-381 (26.209)	LPS-1<br>miR-381 (26.454)	LPS+NAC-3<br>U6 (16.68)	LPS+NAC-3<br>U6 (16.605)	LPS+NAC-3<br>U6 (n/a)	LPS+NAC-3<br>miR-381 (23.319)	LPS+NAC-3<br>miR-381 (23.216)	LPS+NAC-3<br>miR-381 (23.316)
E	LPS-2<br>U6 (17.217)	LPS-2<br>U6 (17.043)	LPS-2<br>U6 (17.137)	LPS-2<br>miR-381 (26.623)	LPS-2<br>miR-381 (26.58)	LPS-2<br>miR-381 (26.651)	LPS+Bud+NAC-1<br>U6 (17.176)	LPS+Bud+NAC-1<br>U6 (17.285)	LPS+Bud+NAC-1<br>U6 (17.169)	LPS+Bud+NAC-1<br>miR-381 (21.117)	LPS+Bud+NAC-1<br>miR-381 (21.012)	LPS+Bud+NAC-1<br>miR-381 (21.056)
F	LPS-3<br>U6 (16.681)	LPS-3<br>U6 (17.207)	LPS-3<br>U6 (17.11)	LPS-3<br>miR-381 (26.666)	LPS-3<br>miR-381 (26.607)	LPS-3<br>miR-381 (26.715)	LPS+Bud+NAC-2<br>U6 (16.974)	LPS+Bud+NAC-2<br>U6 (16.884)	LPS+Bud+NAC-2<br>U6 (16.797)	LPS+Bud+NAC-2<br>miR-381 (20.87)	LPS+Bud+NAC-2<br>miR-381 (21.12)	LPS+Bud+NAC-2<br>miR-381 (21.041)
G	LPS+Bud-1<br>U6 (17.261)	LPS+Bud-1<br>U6 (17.247)	LPS+Bud-1<br>U6 (17.017)	LPS+Bud-1<br>miR-381 (23.69)	LPS+Bud-1<br>miR-381 (23.894)	LPS+Bud-1<br>miR-381 (23.898)	LPS+Bud+NAC-3<br>U6 (16.502)	LPS+Bud+NAC-3<br>U6 (16.98)	LPS+Bud+NAC-3<br>U6 (16.02)	LPS+Bud+NAC-3<br>miR-381 (21.23)	LPS+Bud+NAC-3<br>miR-381 (21.303)	LPS+Bud+NAC-3<br>miR-381 (21.19)
H	LPS+Bud-3<br>U6 (17.85)	LPS+Bud-3<br>U6 (16.498)	LPS+Bud-3<br>U6 (17.129)	LPS+Bud-3<br>miR-381 (23.681)	LPS+Bud-3<br>miR-381 (23.746)	LPS+Bud-3<br>miR-381 (24.067)						

Amplification Plot (dRn)

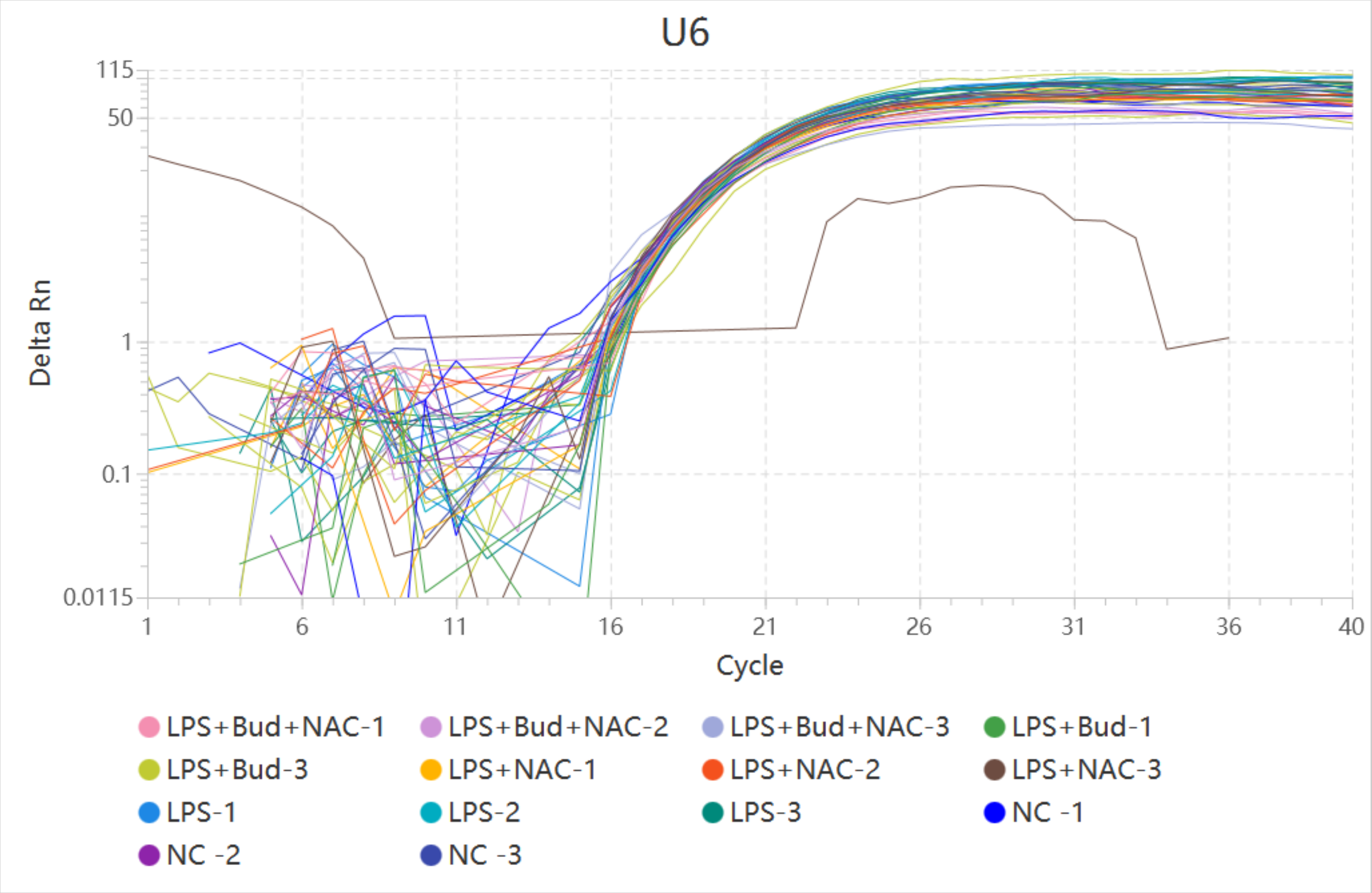

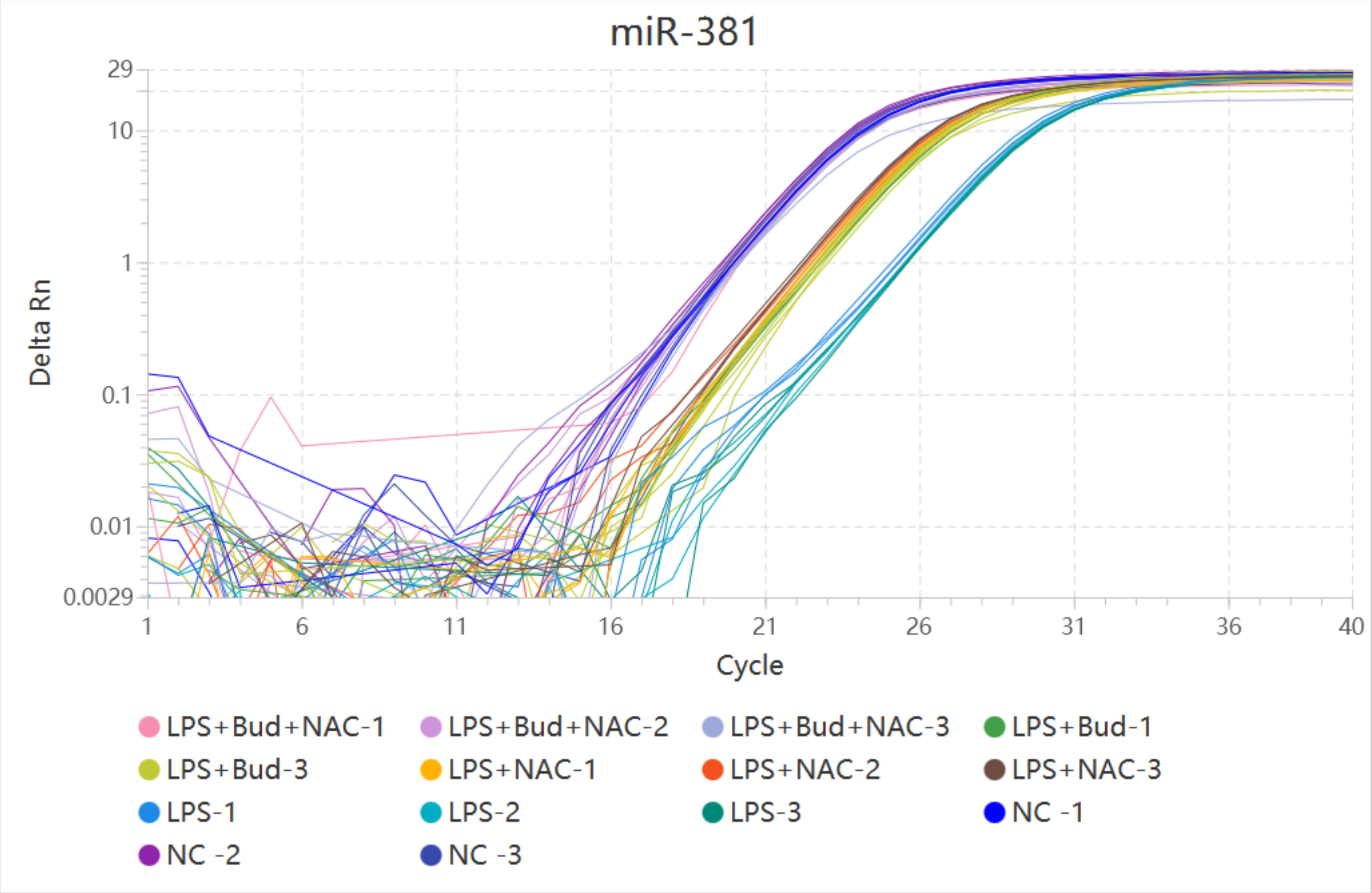

Amplification Plot (Rn)

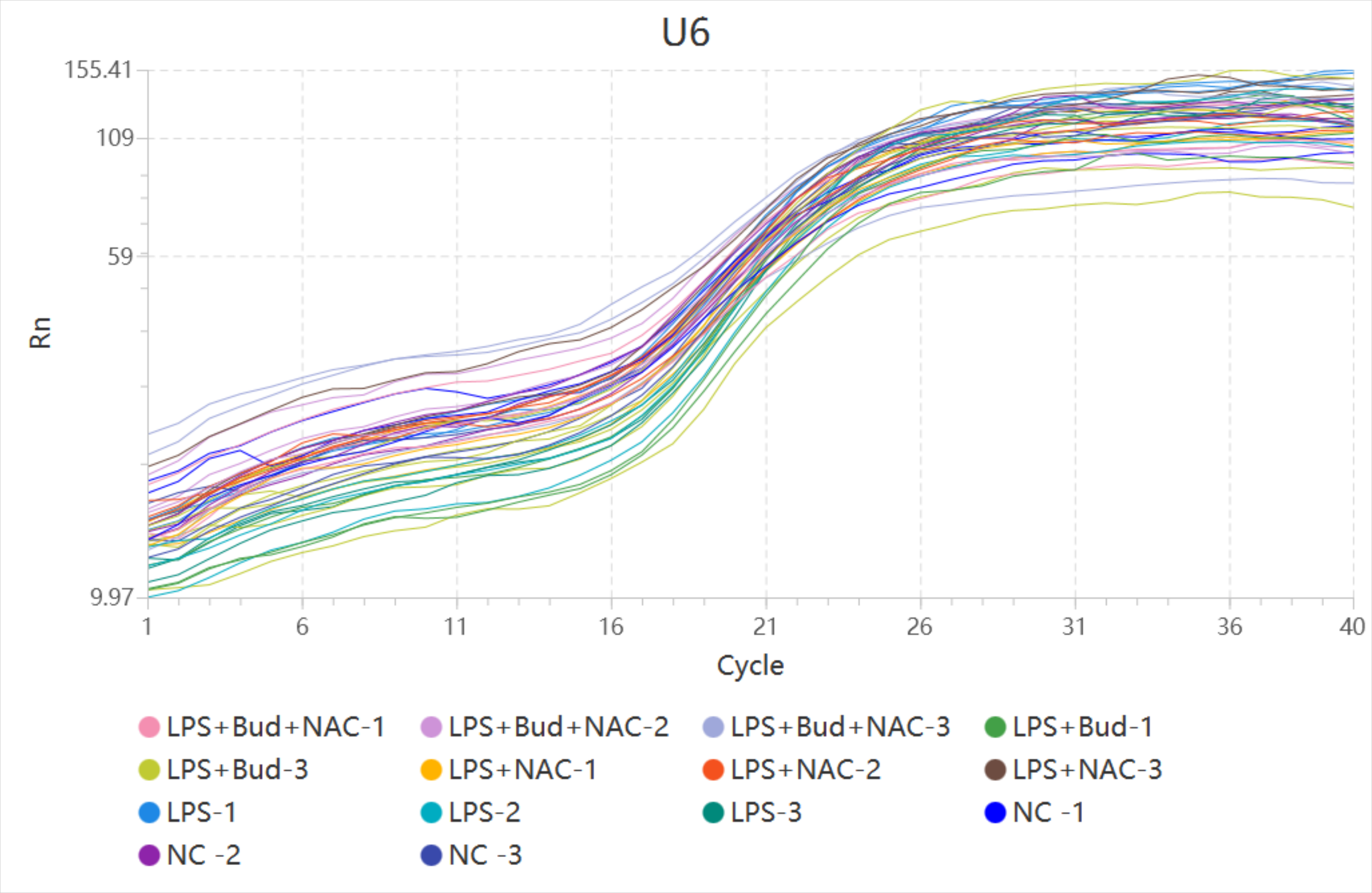

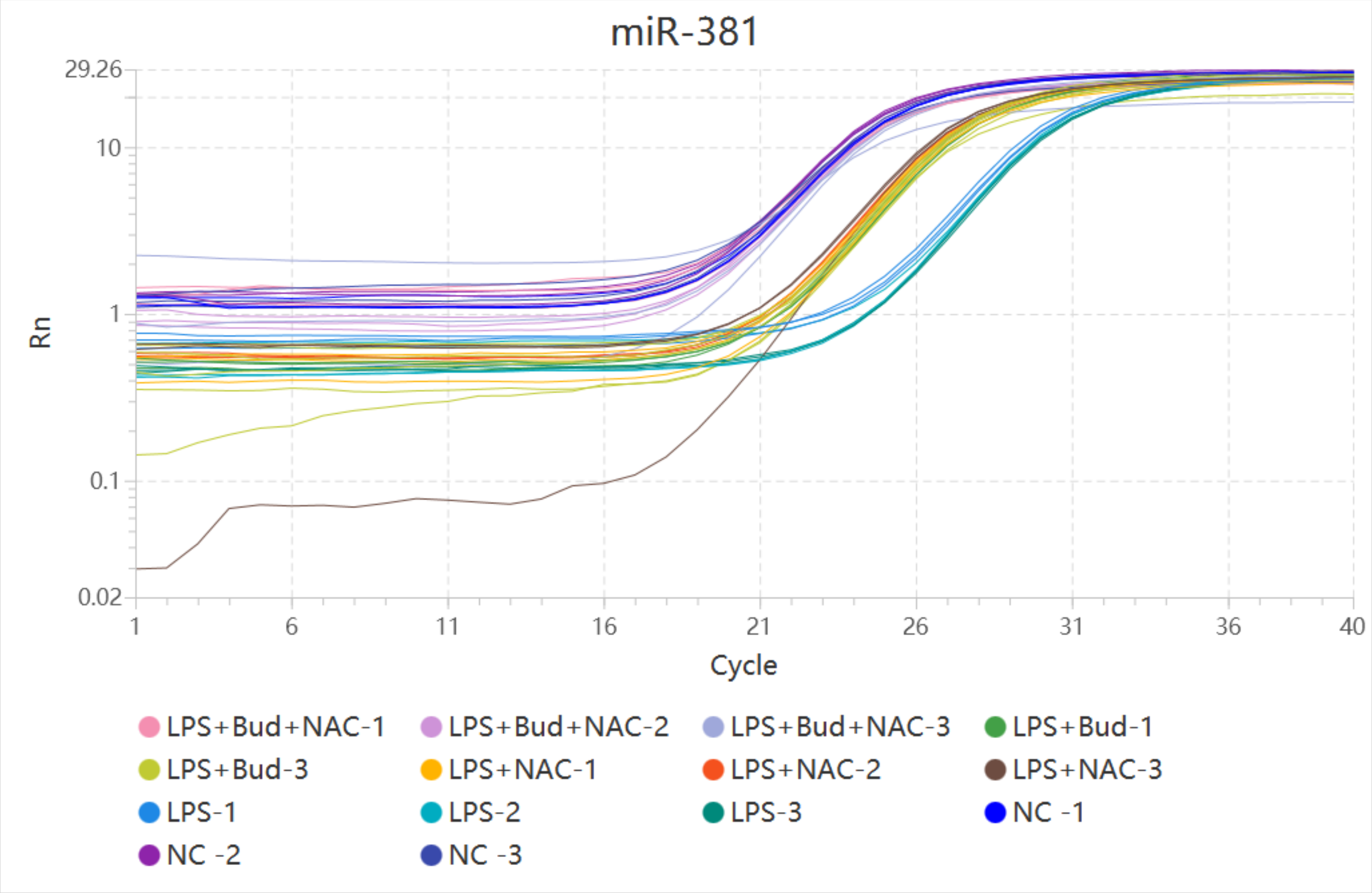

Melt Curve Plot

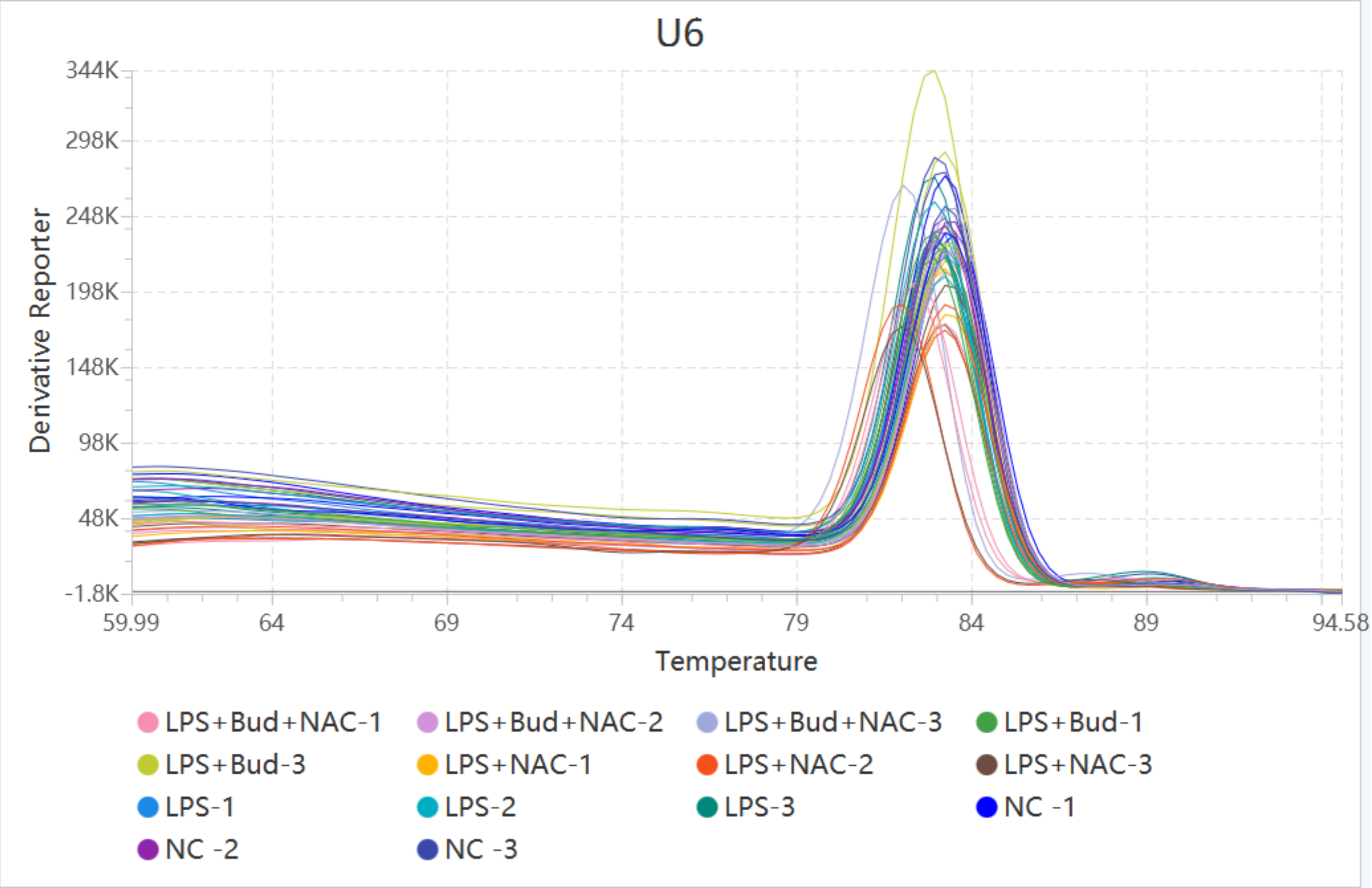

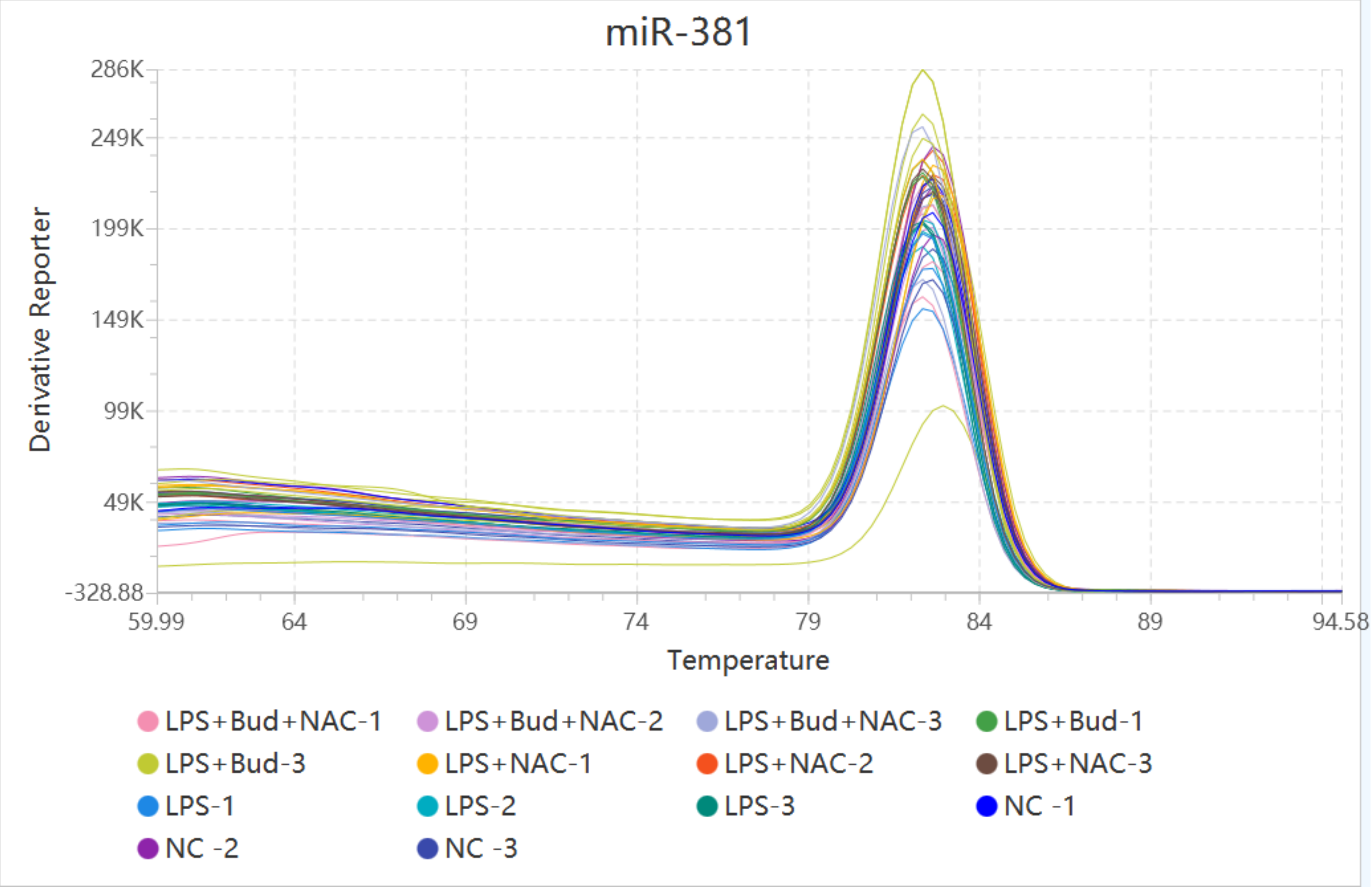

Run Method

Block Type	96-Well 0.2-mL Block
Sample Volume	20.0
Cover Temperature	105.0
Run mode	FAST

Stage	Collection Flag	Ramp Rate	Temperature	Hold Time	Starting Cycle	Auto Delta Temperature	Auto Delta Hold Time
Hold Stage	false	2.74°C/sec	95.0°C	20	-	-	-
PCR Stage (40 cycles)	false	2.74°C/sec	95.0°C	1	-	-	-
	true	2.12°C/sec	60.0°C	20	-	-	-
Melt Stage	false	2.74°C/sec	95.0°C	1	-	-	-
	false	2.12°C/sec	60.0°C	20	-	-	-
	true	0.15°C/sec	95.0°C	1	-	-	-

Primary Analysis Settings

General

PCR Stage/Step	Stage 2, Step 2
Quantification Cycle Method	Baseline Threshold

Target	Auto Threshold	Threshold	Auto Baseline	Baseline Start	Baseline End
DEFAULT	Yes	AUTO	Yes	AUTO	AUTO

Melt

Melt Stage/Step	Stage 3, Step 3

Target	Multi Peak	Threshold Type	Peak Level (%)	Peak Height
DEFAULT	Yes	Percentage	10	-
U6	Yes	Percentage	10	-

QC Alerts

Curve Quality Alert Enabled	No
Results Quality Alert Enabled	Yes

Advanced

Set the Delta-Rn below which curves will be considered Non-Amplified	No
Primary Analysis Variant	N/A

Relative Quantification Settings

General

RQ Min/Max Calculations	Confidence Level (95.0)
Max Allowed EqCq Mean	40
Include Adjusted EqCq Mean	No
Analysis Type	Singleplex

Endo Controls

Normalization Type	Specific endogenous control

Target	Endogenous Control	Auto	Efficiency(%)
U6	Yes	Yes	AUTO

References

Reference Sample	NC -1

Relative Quantification Results (Sample)

Sample	Target	EqCq Mean	Adjusted EqCq Mean	ΔEqCq Mean	ΔEqCq SD	ΔEqCq SE	ΔΔEqCq	RQ	RQ Min	RQ Max
NC -1	U6	16.776	16.776	-	-	-	-	-	-	-
NC -1	miR-381	21.06	21.06	4.284	0.277	0.16	-	1	0.735	1.361
LPS+Bud-3	U6	17.005	17.005	-	-	-	-	-	-	-
LPS+Bud-3	miR-381	23.824	23.824	6.819	0.52	0.212	2.535	0.173	0.124	0.239
NC -2	U6	16.801	16.801	-	-	-	-	-	-	-
NC -2	miR-381	20.746	20.746	3.946	0.175	0.101	-0.338	1.264	1.041	1.535
LPS+NAC-1	U6	16.84	16.84	-	-	-	-	-	-	-
LPS+NAC-1	miR-381	23.616	23.616	6.775	0.152	0.088	2.491	0.178	0.15	0.21
NC -3	U6	16.753	16.753	-	-	-	-	-	-	-
NC -3	miR-381	20.908	20.908	4.155	0.114	0.066	-0.129	1.093	0.964	1.24
LPS+NAC-2	U6	17.026	17.026	-	-	-	-	-	-	-
LPS+NAC-2	miR-381	23.464	23.464	6.438	0.317	0.183	2.154	0.225	0.158	0.319
LPS-1	U6	16.909	16.909	-	-	-	-	-	-	-
LPS-1	miR-381	26.348	26.348	9.439	0.152	0.088	5.155	0.028	0.024	0.033
LPS+NAC-3	U6	16.643	16.643	-	-	-	-	-	-	-
LPS+NAC-3	miR-381	23.284	23.284	6.641	0.079	0.05	2.357	0.195	0.175	0.218
LPS-2	U6	17.132	17.132	-	-	-	-	-	-	-
LPS-2	miR-381	26.618	26.618	9.486	0.094	0.054	5.202	0.027	0.024	0.03
LPS+Bud+NAC-1	U6	17.21	17.21	-	-	-	-	-	-	-
LPS+Bud+NAC-1	miR-381	21.062	21.062	3.851	0.084	0.048	-0.432	1.349	1.23	1.481
LPS-3	U6	17	17	-	-	-	-	-	-	-
LPS-3	miR-381	26.663	26.663	9.663	0.285	0.165	5.379	0.024	0.018	0.033
LPS+Bud+NAC-2	U6	16.885	16.885	-	-	-	-	-	-	-
LPS+Bud+NAC-2	miR-381	21.01	21.01	4.125	0.156	0.09	-0.158	1.116	0.939	1.327
LPS+Bud-1	U6	17.175	17.175	-	-	-	-	-	-	-
LPS+Bud-1	miR-381	23.827	23.827	6.652	0.181	0.105	2.369	0.194	0.158	0.237
LPS+Bud+NAC-3	U6	16.501	16.501	-	-	-	-	-	-	-
LPS+Bud+NAC-3	miR-381	21.241	21.241	4.74	0.484	0.279	0.456	0.729	0.426	1.248

Relative Quantification Plot

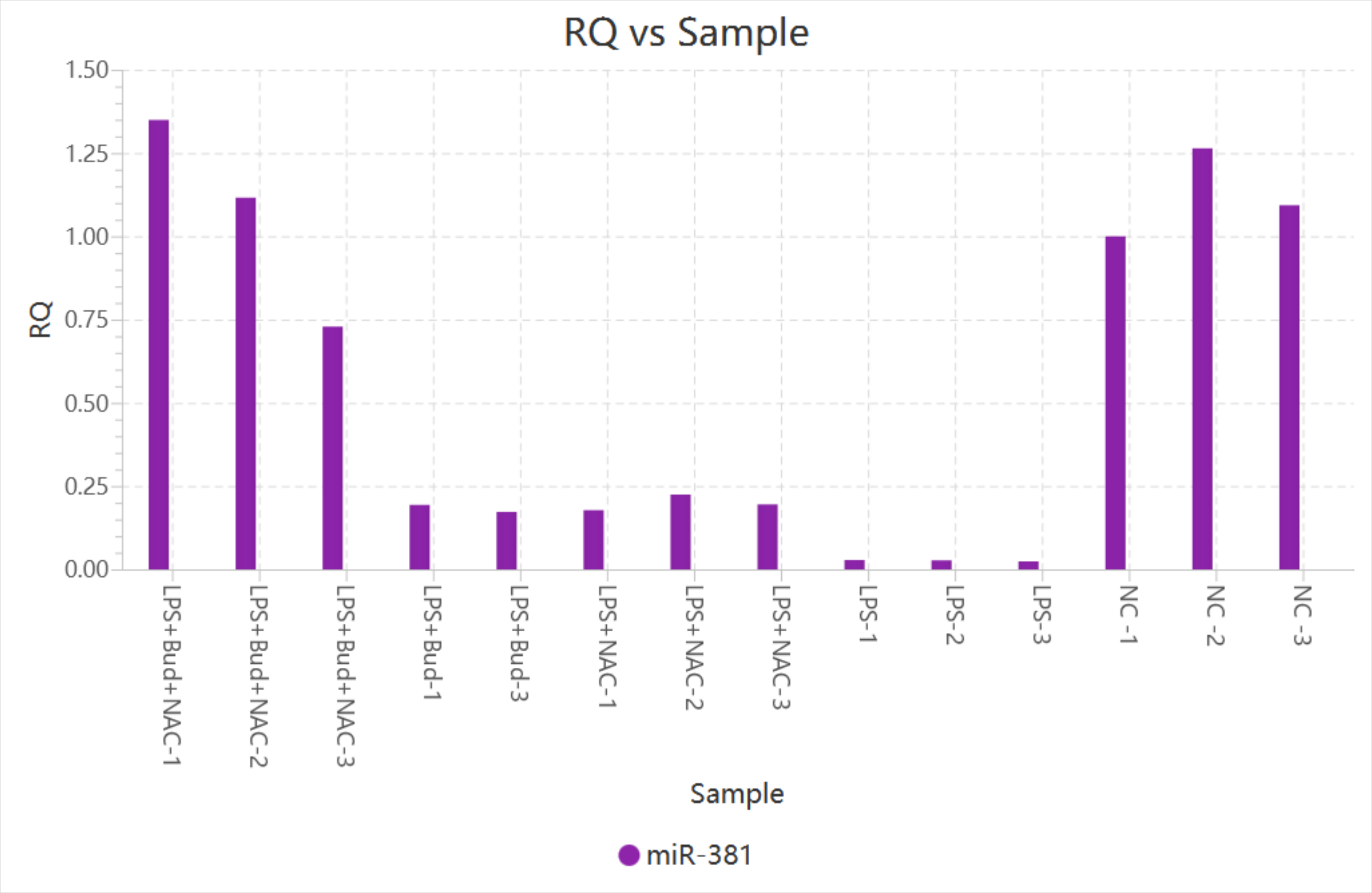

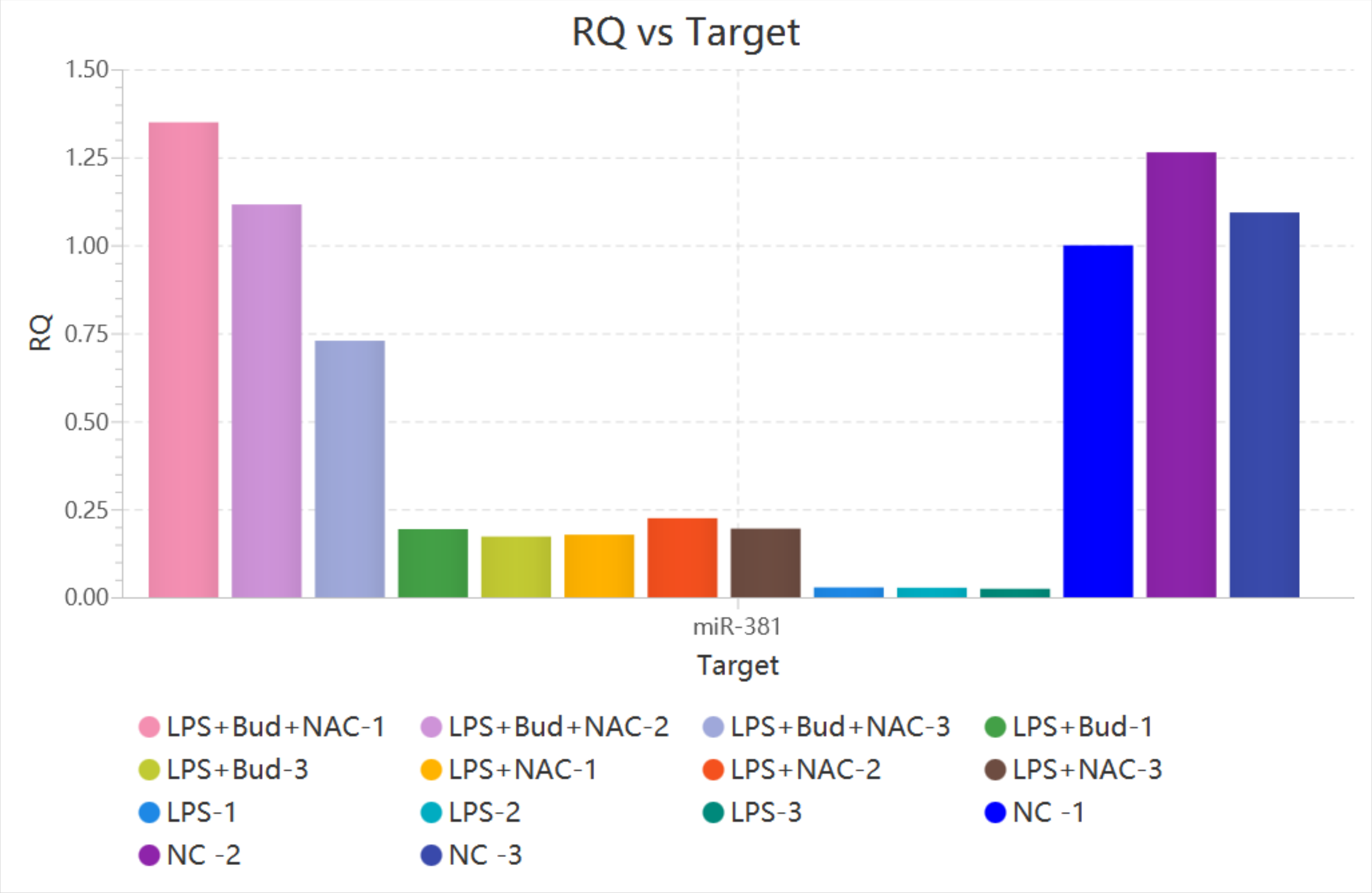

- End of Report -
