# Supplementary material for: Combination therapy with budesonide and acetylcysteine alleviates LPS-induced acute lung injury via the miR-381/NLRP3 molecular axis
Source: PLoS One. 2023 Aug 9;18(8):e0289818. doi: 10.1371/journal.pone.0289818 (PMC10411794; doi:10.1371/journal.pone.0289818)
Supplement: S3 File — (ZIP) [file pone.0289818.s003.zip › S3 File. Fig3 Original data/date/3B/1-1/Results_Report_2023-05-16-112614.pdf]

Plate Results Report

A229-1-1.eds

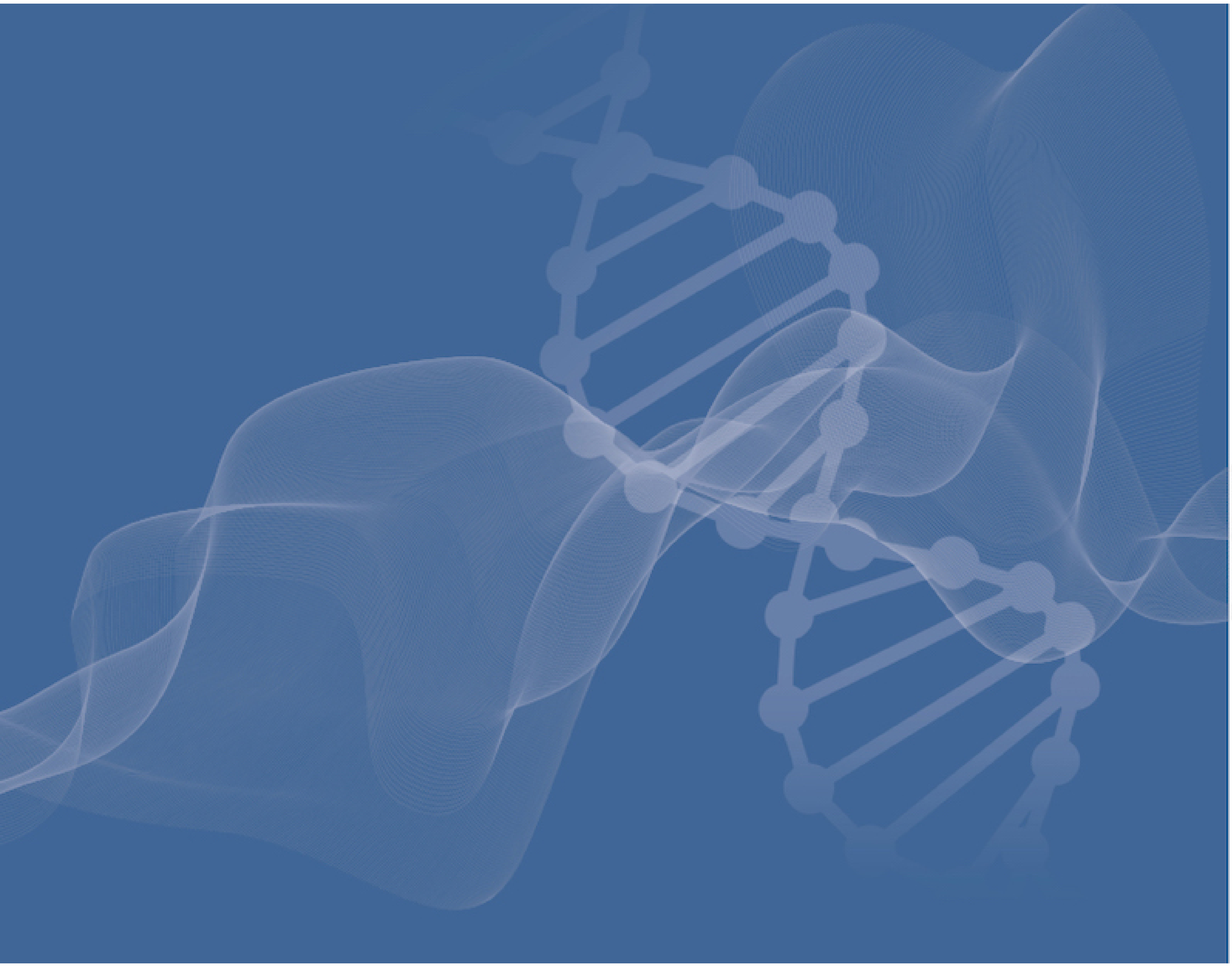

Summary

Property	Details
Bar Code	-
File Name	A229-1-1.eds
Run Start Date/Time	Mar 23, 2022 4:19:32 PM
Run End Date/Time	Mar 23, 2022 5:15:30 PM
Run Duration	55 minutes, and 57 seconds
Operator	DEFAULT
Instrument Name	SVT004
Instrument Type	QuantStudio™ 3 System
Instrument Serial Number	SVT004
Block Type	96-Well 0.2-mL
Block Serial Number	41145627
Heated Cover Serial Number	N/A
PCR Stage/Step Number	Stage 2, Step 2
Melt Stage Number	Stage 3
Quantification Cycle Method	Baseline Threshold
Comment	-
Software Name and Version	Design & Analysis Software v2.6.0
Plugin Name and Version	Primary Analysis v1.7.0, Relative Quantification v1.5.0
Analysis Date/Time	May 16, 2023 11:26:10 AM

Well Table

Well	Sample	Target	Task	Cq	Cq Confidence	Amp Score	Amp Status	Cq Threshold	Baseline Start/End	Melt Temp
A1	NC-1	U6	Unknown	16.876	0.773	1.849	AMP	1.286	3-12	83.4
A2	NC-1	U6	Unknown	16.966	0.781	1.879	AMP	1.286	3-13	83.25
A3	NC-1	U6	Unknown	16.985	0.774	1.876	AMP	1.286	3-13	83.548
A4	NC-1	miR-381	Unknown	20.585	0.969	1.957	AMP	2.03	3-14	82.803
A5	NC-1	miR-381	Unknown	20.559	0.99	1.961	AMP	2.03	3-14	82.801
A6	NC-1	miR-381	Unknown	20.463	0.992	1.973	AMP	2.03	3-14	82.801
A7	ALI-4	U6	Unknown	17.265	0.812	1.914	AMP	1.286	3-13	83.248
A8	ALI-4	U6	Unknown	17.26	0.771	1.915	AMP	1.286	3-12	83.248
A9	ALI-4	U6	Unknown	17.284	0.759	1.893	AMP	1.286	3-13	83.099
A10	ALI-4	miR-381	Unknown	26.392	0.993	1.941	AMP	2.03	3-19	82.353
A11	ALI-4	miR-381	Unknown	26.484	0.993	1.952	AMP	2.03	3-18	82.804
A12	ALI-4	miR-381	Unknown	26.82	0.984	1.922	AMP	2.03	3-19	82.953
B1	NC-2	U6	Unknown	16.857	0.871	1.906	AMP	1.286	3-13	83.25
B2	NC-2	U6	Unknown	16.894	0.821	1.905	AMP	1.286	3-13	83.25
B3	NC-2	U6	Unknown	16.9	0.805	1.908	AMP	1.286	3-13	83.399
B4	NC-2	miR-381	Unknown	20.476	0.993	1.974	AMP	2.03	3-13	82.653
B5	NC-2	miR-381	Unknown	20.64	0.987	1.959	AMP	2.03	3-14	82.652
B6	NC-2	miR-381	Unknown	20.409	0.99	1.967	AMP	2.03	3-13	82.801
B7	ALI-5	U6	Unknown	17.243	0.956	1.91	AMP	1.286	3-13	83.248
B8	ALI-5	U6	Unknown	17.306	0.92	1.91	AMP	1.286	3-13	83.248
B9	ALI-5	U6	Unknown	17.112	0.859	1.914	AMP	1.286	3-13	83.099
B10	ALI-5	miR-381	Unknown	26.387	0.993	1.958	AMP	2.03	3-17	82.204
B11	ALI-5	miR-381	Unknown	26.566	0.994	1.947	AMP	2.03	3-18	82.506
B12	ALI-5	miR-381	Unknown	26.85	0.993	1.921	AMP	2.03	3-18	82.506
C1	NC-3	U6	Unknown	16.526	0.834	1.915	AMP	1.286	3-11	83.101
C2	NC-3	U6	Unknown	16.775	0.932	1.912	AMP	1.286	3-12	83.101
C3	NC-3	U6	Unknown	17.118	0.772	1.908	AMP	1.286	3-12	83.399
C4	NC-3	miR-381	Unknown	20.596	0.994	1.964	AMP	2.03	3-14	82.653
C5	NC-3	miR-381	Unknown	20.722	0.994	1.967	AMP	2.03	3-14	82.652
C6	NC-3	miR-381	Unknown	20.633	0.99	1.953	AMP	2.03	3-14	82.652

Well	Sample	Target	Task	Cq	Cq Confidence	Amp Score	Amp Status	Cq Threshold	Baseline Start/End	Melt Temp
C7	Bud-1	U6	Unknown	17.413	0.787	1.893	AMP	1.286	3-14	82.503
C8	Bud-1	U6	Unknown	17.169	0.895	1.913	AMP	1.286	3-14	83.248
C9	Bud-1	U6	Unknown	17.262	0.752	1.913	AMP	1.286	3-14	83.099
C10	Bud-1	miR-381	Unknown	23.647	0.994	1.936	AMP	2.03	3-14	82.204
C11	Bud-1	miR-381	Unknown	23.842	0.985	1.925	AMP	2.03	3-16	82.357
C12	Bud-1	miR-381	Unknown	23.757	0.994	1.93	AMP	2.03	3-16	82.506
D1	NC-4	U6	Unknown	17.315	0.773	1.93	AMP	1.286	3-13	83.101
D2	NC-4	U6	Unknown	16.814	0.825	1.928	AMP	1.286	3-13	82.952
D3	NC-4	U6	Unknown	17.039	0.878	1.92	AMP	1.286	3-12	83.25
D4	NC-4	miR-381	Unknown	20.675	0.994	1.952	AMP	2.03	3-14	82.504
D5	NC-4	miR-381	Unknown	20.886	0.994	1.952	AMP	2.03	3-13	82.503
D6	NC-4	miR-381	Unknown	20.763	0.993	1.946	AMP	2.03	3-12	82.652
D7	Bud-2	U6	Unknown	17.093	0.857	1.903	AMP	1.286	3-13	83.248
D8	Bud-2	U6	Unknown	17.046	0.694	1.925	AMP	1.286	3-13	83.248
D9	Bud-2	U6	Unknown	17.042	0.755	1.915	AMP	1.286	3-13	83.099
D10	Bud-2	miR-381	Unknown	23.754	0.994	1.944	AMP	2.03	3-15	82.055
D11	Bud-2	miR-381	Unknown	23.618	0.989	1.941	AMP	2.03	3-16	82.357
D12	Bud-2	miR-381	Unknown	23.87	0.989	1.913	AMP	2.03	3-15	82.357
E1	NC-5	U6	Unknown	17.184	0.789	1.919	AMP	1.286	3-14	83.101
E2	NC-5	U6	Unknown	17.088	0.72	1.928	AMP	1.286	3-14	82.952
E3	NC-5	U6	Unknown	17.348	0.822	1.901	AMP	1.286	3-13	83.25
E4	NC-5	miR-381	Unknown	20.859	0.994	1.938	AMP	2.03	3-12	82.504
E5	NC-5	miR-381	Unknown	21.33	0.987	1.942	AMP	2.03	3-13	82.205
E6	NC-5	miR-381	Unknown	20.837	0.994	1.945	AMP	2.03	3-11	82.503
E7	Bud-3	U6	Unknown	17.051	0.745	1.888	AMP	1.286	3-14	82.801
E8	Bud-3	U6	Unknown	17.079	0.846	1.881	AMP	1.286	3-14	82.652
E9	Bud-3	U6	Unknown	17.065	0.865	1.896	AMP	1.286	3-13	83.099
E10	Bud-3	miR-381	Unknown	23.544	0.993	1.939	AMP	2.03	3-14	82.204
E11	Bud-3	miR-381	Unknown	23.585	0.991	1.922	AMP	2.03	3-15	82.357
E12	Bud-3	miR-381	Unknown	23.568	0.993	1.931	AMP	2.03	3-15	82.357
F1	ALI-1	U6	Unknown	16.865	0.809	1.914	AMP	1.286	3-12	82.952

Well	Sample	Target	Task	Cq	Cq Confidence	Amp Score	Amp Status	Cq Threshold	Baseline Start/End	Melt Temp
F2	ALI-1	U6	Unknown	17.333	0.85	1.922	AMP	1.286	3-13	82.952
F3	ALI-1	U6	Unknown	17.278	0.778	1.91	AMP	1.286	3-12	83.25
F4	ALI-1	miR-381	Unknown	25.881	0.974	1.972	AMP	2.03	3-18	82.504
F5	ALI-1	miR-381	Unknown	25.833	0.993	1.975	AMP	2.03	3-16	82.503
F6	ALI-1	miR-381	Unknown	26.203	0.993	1.962	AMP	2.03	3-18	82.503
F7	Bud-4	U6	Unknown	17.061	0.917	1.891	AMP	1.286	3-13	83.397
F8	Bud-4	U6	Unknown	17.044	0.882	1.875	AMP	1.286	3-12	83.397
F9	Bud-4	U6	Unknown	17.073	0.794	1.874	AMP	1.286	3-12	83.248
F10	Bud-4	miR-381	Unknown	23.264	0.978	1.95	AMP	2.03	3-16	82.204
F11	Bud-4	miR-381	Unknown	23.259	0.992	1.944	AMP	2.03	3-16	82.357
F12	Bud-4	miR-381	Unknown	23.349	0.99	1.939	AMP	2.03	3-15	82.357
G1	ALI-2	U6	Unknown	17.258	0.796	1.9	AMP	1.286	3-13	82.952
G2	ALI-2	U6	Unknown	17.394	0.823	1.898	AMP	1.286	3-12	82.803
G3	ALI-2	U6	Unknown	16.968	0.806	1.927	AMP	1.286	3-12	83.101
G4	ALI-2	miR-381	Unknown	25.897	0.995	1.976	AMP	2.03	3-17	82.504
G5	ALI-2	miR-381	Unknown	26.122	0.993	1.973	AMP	2.03	3-18	82.503
G6	ALI-2	miR-381	Unknown	26.029	0.99	1.975	AMP	2.03	3-16	82.503
G7	Bud-5	U6	Unknown	19.232	0.763	1.799	AMP	1.286	3-14	82.204
G8	Bud-5	U6	Unknown	17.026	0.87	1.859	AMP	1.286	3-13	83.397
G9	Bud-5	U6	Unknown	17.07	0.772	1.892	AMP	1.286	3-13	83.248
G10	Bud-5	miR-381	Unknown	23.075	0.991	1.959	AMP	2.03	3-15	82.353
G11	Bud-5	miR-381	Unknown	23.421	0.992	1.921	AMP	2.03	3-15	82.506
G12	Bud-5	miR-381	Unknown	23.289	0.988	1.953	AMP	2.03	3-15	82.506
H1	ALI-3	U6	Unknown	17.617	0.822	1.878	AMP	1.286	3-13	82.803
H2	ALI-3	U6	Unknown	17.509	0.841	1.946	AMP	1.286	3-13	82.803
H3	ALI-3	U6	Unknown	17.295	0.867	1.927	AMP	1.286	3-13	83.101
H4	ALI-3	miR-381	Unknown	25.994	0.993	1.979	AMP	2.03	3-18	82.504
H5	ALI-3	miR-381	Unknown	26.182	0.992	1.968	AMP	2.03	3-18	82.503
H6	ALI-3	miR-381	Unknown	26.492	0.992	1.954	AMP	2.03	3-17	82.503
H7	NAC-1	U6	Unknown	17.03	0.842	1.874	AMP	1.286	3-13	82.353
H8	NAC-1	U6	Unknown	17.358	0.826	1.87	AMP	1.286	3-13	82.204

Well	Sample	Target	Task	Cq	Cq Confidence	Amp Score	Amp Status	Cq Threshold	Baseline Start/End	Melt Temp
H9	NAC-1	U6	Unknown	16.919	0.839	1.863	AMP	1.286	3-11	83.248
H10	NAC-1	miR-381	Unknown	23.101	0.992	1.95	AMP	2.03	3-14	82.353
H11	NAC-1	miR-381	Unknown	23.175	0.993	1.948	AMP	2.03	3-16	82.506
H12	NAC-1	miR-381	Unknown	24.059	0.978	1.837	AMP	2.03	3-16	82.655

Replicate Group Table

Sample	Target	No. of Replicates	Cq Mean	Cq SD
ALI-1	U6	3	17.159	0.255
ALI-1	miR-381	3	25.972	0.201
ALI-2	U6	3	17.206	0.218
ALI-2	miR-381	3	26.016	0.113
ALI-3	U6	3	17.474	0.164
ALI-3	miR-381	3	26.223	0.252
ALI-4	U6	3	17.27	0.012
ALI-4	miR-381	3	26.565	0.225
ALI-5	U6	3	17.22	0.099
ALI-5	miR-381	3	26.601	0.233
Bud-1	U6	3	17.281	0.124
Bud-1	miR-381	3	23.749	0.098
Bud-2	U6	3	17.061	0.028
Bud-2	miR-381	3	23.747	0.126
Bud-3	U6	3	17.065	0.014
Bud-3	miR-381	3	23.566	0.021
Bud-4	U6	3	17.059	0.014
Bud-4	miR-381	3	23.291	0.051
Bud-5	U6	3	17.776	1.261
Bud-5	miR-381	3	23.262	0.175
NAC-1	U6	3	17.103	0.228
NAC-1	miR-381	3	23.445	0.533
NC-1	U6	3	16.942	0.058
NC-1	miR-381	3	20.536	0.064
NC-2	U6	3	16.884	0.023
NC-2	miR-381	3	20.508	0.119
NC-3	U6	3	16.806	0.297
NC-3	miR-381	3	20.65	0.064
NC-4	U6	3	17.056	0.251
NC-4	miR-381	3	20.775	0.106

Sample	Target	No. of Replicates	Cq Mean	Cq SD
NC-5	U6	3	17.207	0.131
NC-5	miR-381	3	21.009	0.278

Plate Layout

	1	2	3	4	5	6	7	8	9	10	11	12
A	NC-1<br>U6 (16.876)	NC-1<br>U6 (16.966)	NC-1<br>U6 (16.985)	NC-1<br>miR-381 (20.585)	NC-1<br>miR-381 (20.559)	NC-1<br>miR-381 (20.463)	ALI-4<br>U6 (17.265)	ALI-4<br>U6 (17.26)	ALI-4<br>U6 (17.284)	ALI-4<br>miR-381 (26.392)	ALI-4<br>miR-381 (26.484)	ALI-4<br>miR-381 (26.82)
B	NC-2<br>U6 (16.857)	NC-2<br>U6 (16.894)	NC-2<br>U6 (16.9)	NC-2<br>miR-381 (20.476)	NC-2<br>miR-381 (20.64)	NC-2<br>miR-381 (20.409)	ALI-5<br>U6 (17.243)	ALI-5<br>U6 (17.306)	ALI-5<br>U6 (17.112)	ALI-5<br>miR-381 (26.387)	ALI-5<br>miR-381 (26.566)	ALI-5<br>miR-381 (26.85)
C	NC-3<br>U6 (16.526)	NC-3<br>U6 (16.775)	NC-3<br>U6 (17.118)	NC-3<br>miR-381 (20.596)	NC-3<br>miR-381 (20.722)	NC-3<br>miR-381 (20.633)	Bud-1<br>U6 (17.413)	Bud-1<br>U6 (17.169)	Bud-1<br>U6 (17.262)	Bud-1<br>miR-381 (23.647)	Bud-1<br>miR-381 (23.842)	Bud-1<br>miR-381 (23.757)
D	NC-4<br>U6 (17.315)	NC-4<br>U6 (16.814)	NC-4<br>U6 (17.039)	NC-4<br>miR-381 (20.675)	NC-4<br>miR-381 (20.886)	NC-4<br>miR-381 (20.763)	Bud-2<br>U6 (17.093)	Bud-2<br>U6 (17.046)	Bud-2<br>U6 (17.042)	Bud-2<br>miR-381 (23.754)	Bud-2<br>miR-381 (23.618)	Bud-2<br>miR-381 (23.87)
E	NC-5<br>U6 (17.184)	NC-5<br>U6 (17.088)	NC-5<br>U6 (17.348)	NC-5<br>miR-381 (20.859)	NC-5<br>miR-381 (21.33)	NC-5<br>miR-381 (20.837)	Bud-3<br>U6 (17.051)	Bud-3<br>U6 (17.079)	Bud-3<br>U6 (17.065)	Bud-3<br>miR-381 (23.544)	Bud-3<br>miR-381 (23.585)	Bud-3<br>miR-381 (23.568)
F	ALI-1<br>U6 (16.865)	ALI-1<br>U6 (17.333)	ALI-1<br>U6 (17.278)	ALI-1<br>miR-381 (25.881)	ALI-1<br>miR-381 (25.833)	ALI-1<br>miR-381 (26.203)	Bud-4<br>U6 (17.061)	Bud-4<br>U6 (17.044)	Bud-4<br>U6 (17.073)	Bud-4<br>miR-381 (23.264)	Bud-4<br>miR-381 (23.259)	Bud-4<br>miR-381 (23.349)
G	ALI-2<br>U6 (17.258)	ALI-2<br>U6 (17.394)	ALI-2<br>U6 (16.968)	ALI-2<br>miR-381 (25.897)	ALI-2<br>miR-381 (26.122)	ALI-2<br>miR-381 (26.029)	Bud-5<br>U6 (19.232)	Bud-5<br>U6 (17.026)	Bud-5<br>U6 (17.07)	Bud-5<br>miR-381 (23.075)	Bud-5<br>miR-381 (23.421)	Bud-5<br>miR-381 (23.289)
H	ALI-3<br>U6 (17.617)	ALI-3<br>U6 (17.509)	ALI-3<br>U6 (17.295)	ALI-3<br>miR-381 (25.994)	ALI-3<br>miR-381 (26.182)	ALI-3<br>miR-381 (26.492)	NAC-1<br>U6 (17.03)	NAC-1<br>U6 (17.358)	NAC-1<br>U6 (16.919)	NAC-1<br>miR-381 (23.101)	NAC-1<br>miR-381 (23.175)	NAC-1<br>miR-381 (24.059)

Amplification Plot (dRn)

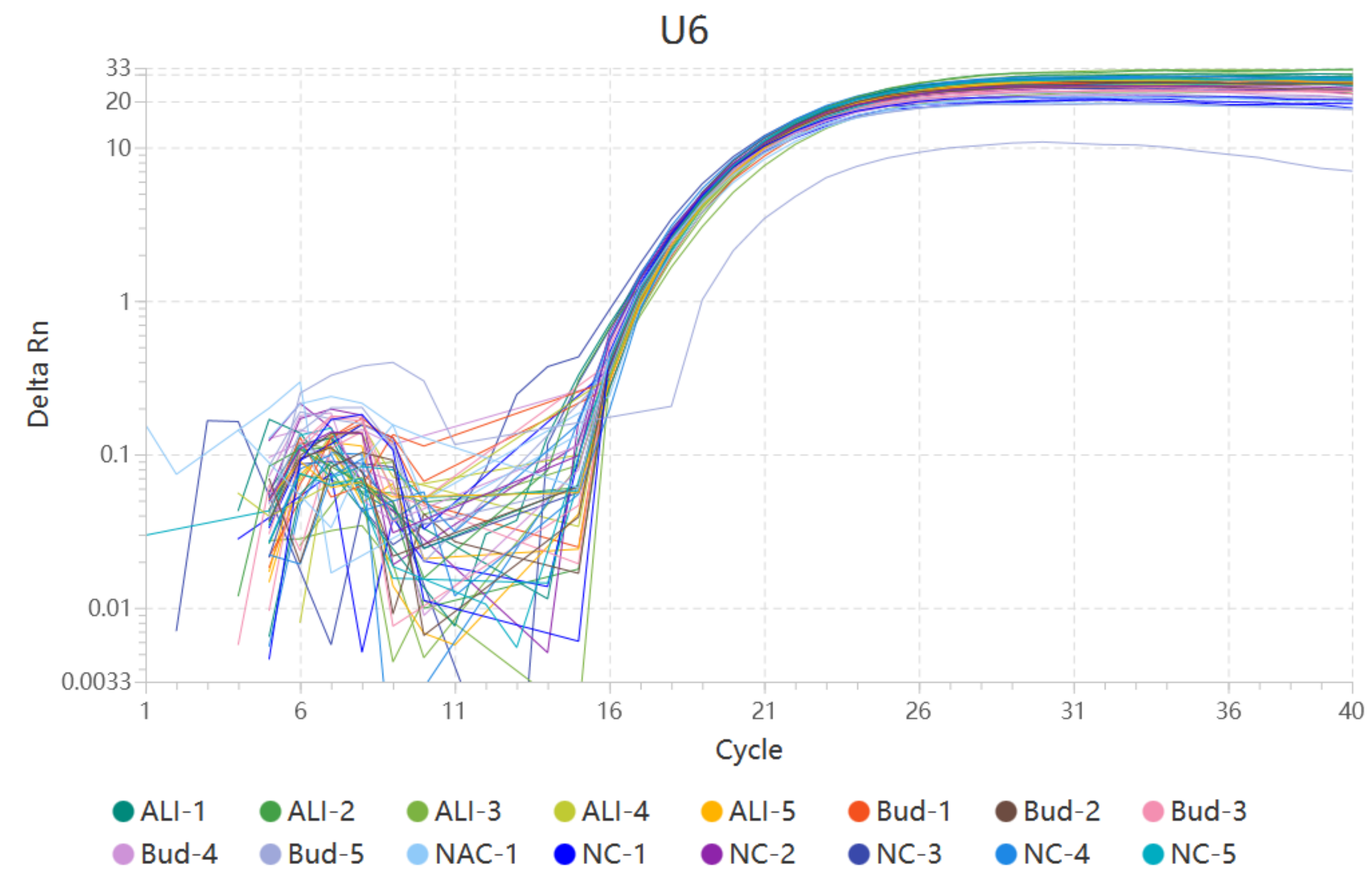

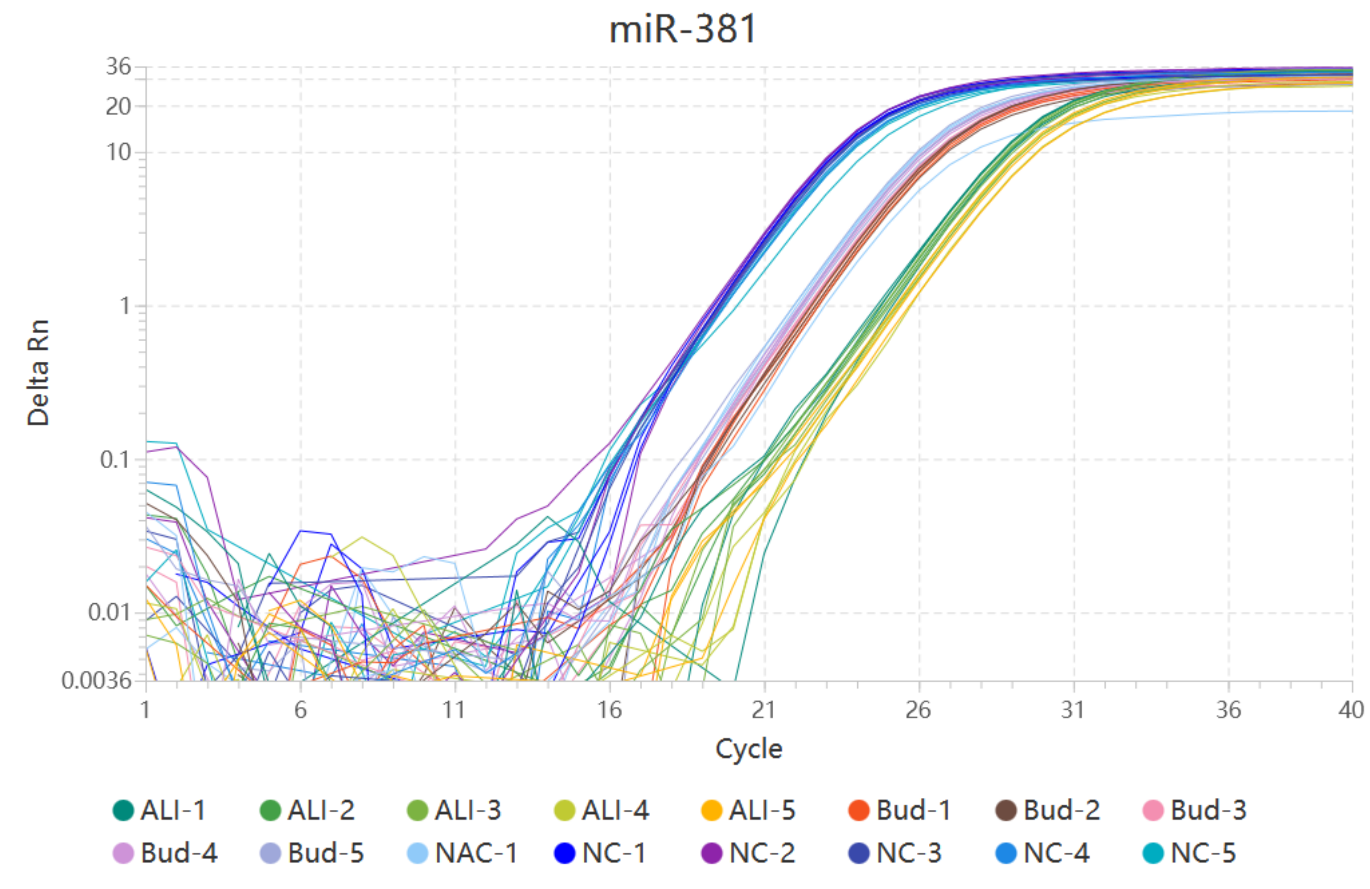

Amplification Plot (Rn)

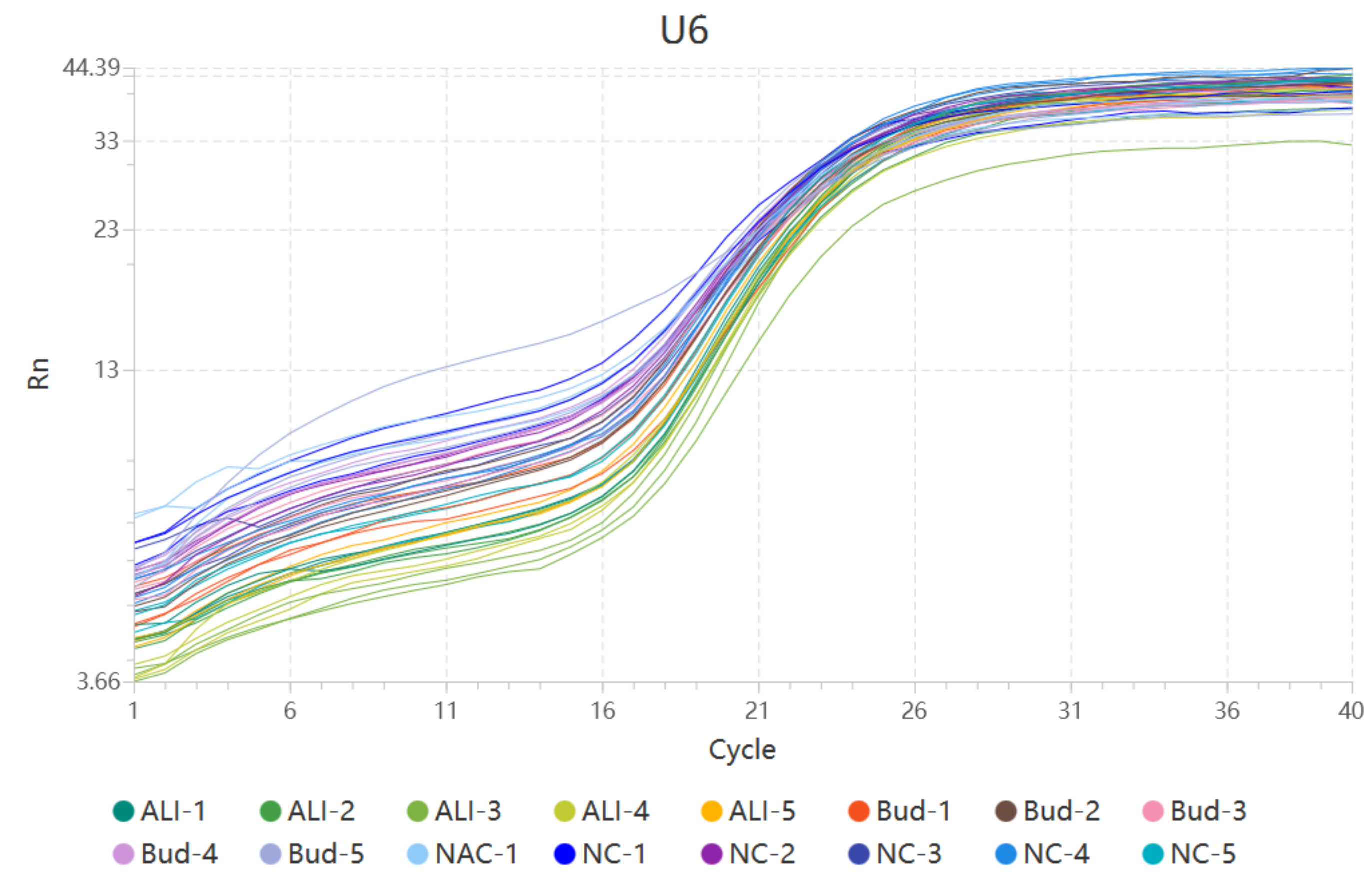

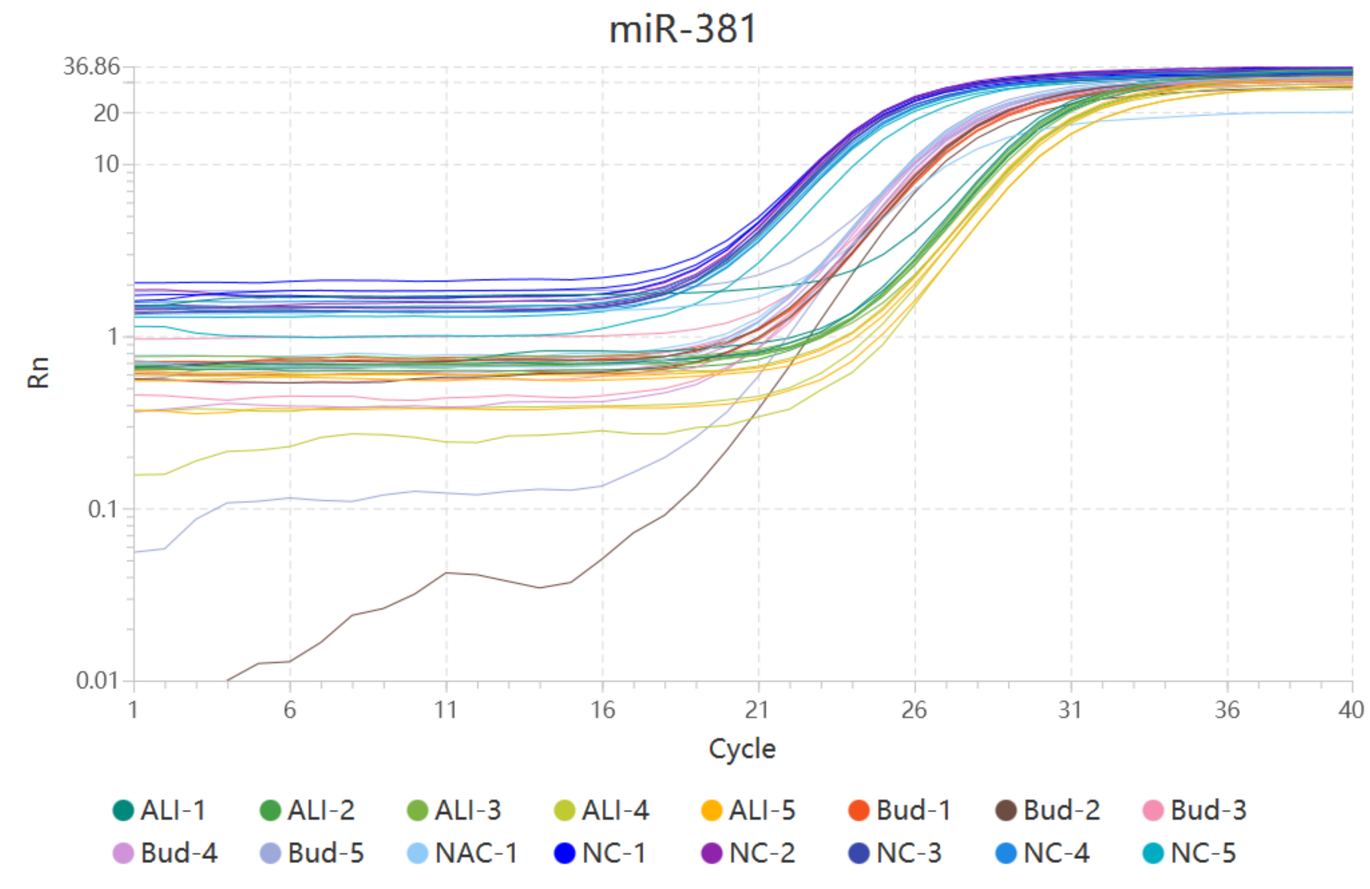

Melt Curve Plot

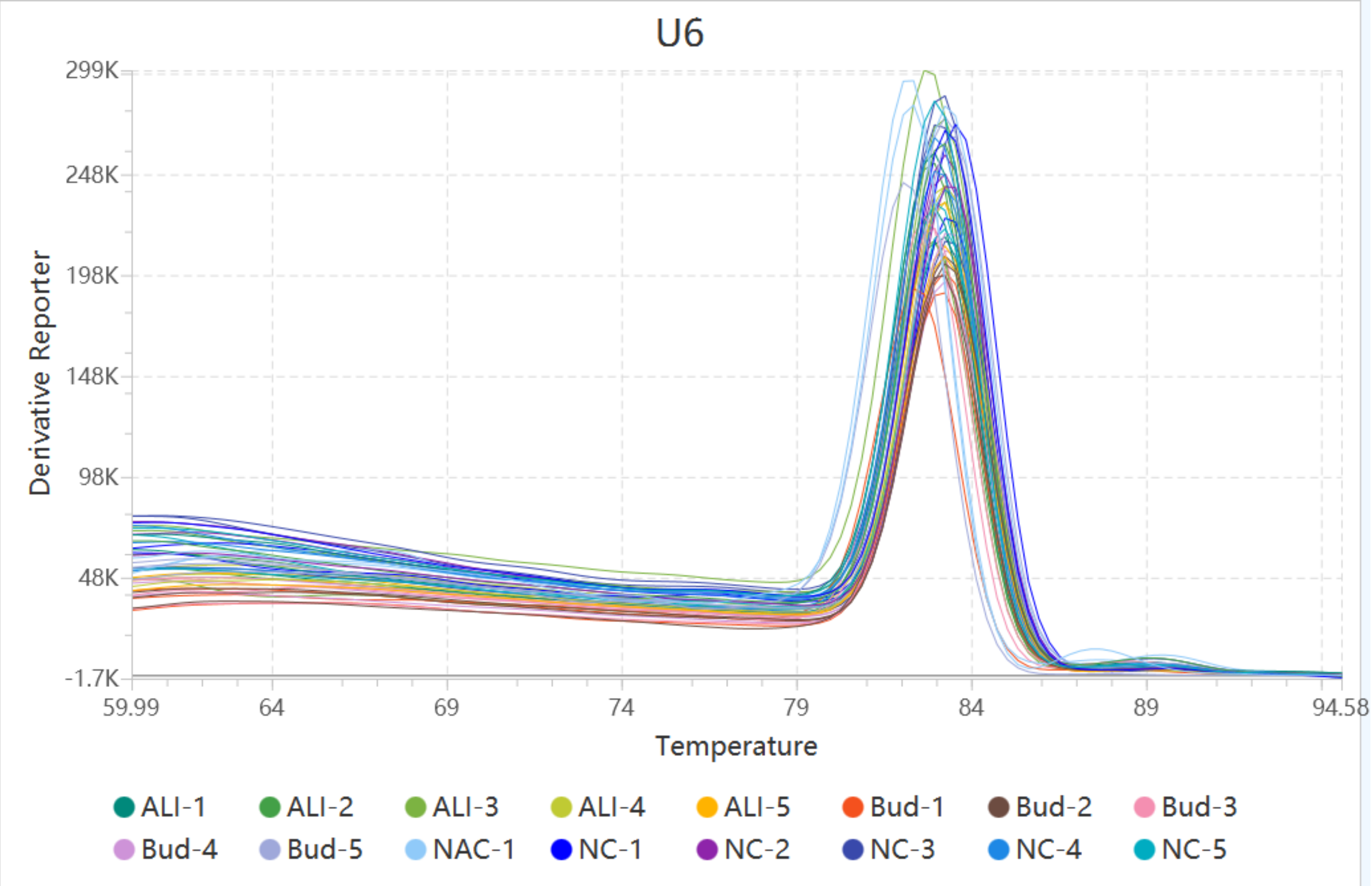

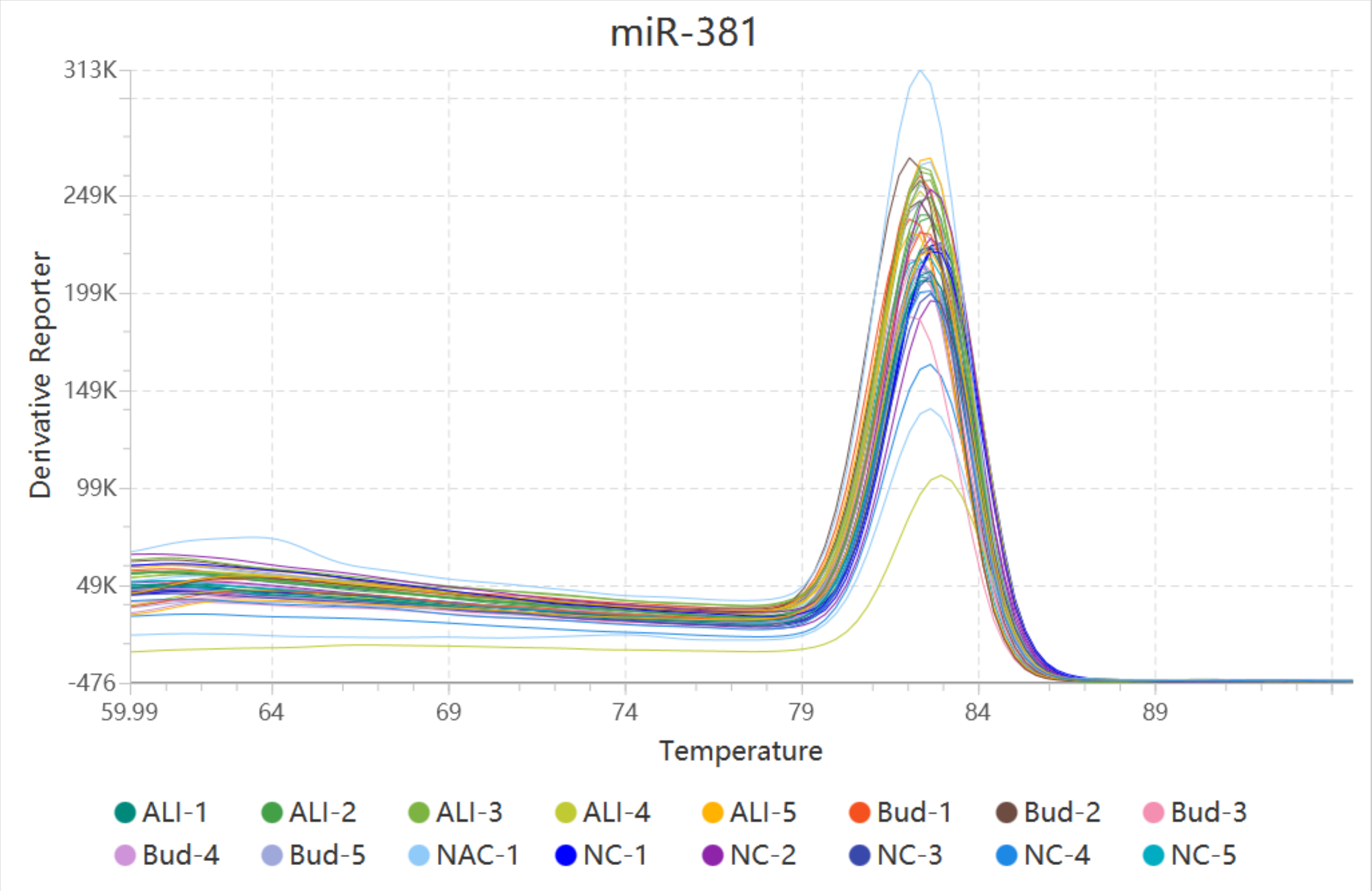

Run Method

Block Type	96-Well 0.2-mL Block
Sample Volume	20.0
Cover Temperature	105.0
Run mode	FAST

Stage	Collection Flag	Ramp Rate	Temperature	Hold Time	Starting Cycle	Auto Delta Temperature	Auto Delta Hold Time
Hold Stage	false	2.74°C/sec	95.0°C	20	-	-	-
PCR Stage (40 cycles)	false	2.74°C/sec	95.0°C	1	-	-	-
	true	2.12°C/sec	60.0°C	20	-	-	-
Melt Stage	false	2.74°C/sec	95.0°C	1	-	-	-
	false	2.12°C/sec	60.0°C	20	-	-	-
	true	0.15°C/sec	95.0°C	1	-	-	-

Primary Analysis Settings

General

PCR Stage/Step	Stage 2, Step 2
Quantification Cycle Method	Baseline Threshold

Target	Auto Threshold	Threshold	Auto Baseline	Baseline Start	Baseline End
DEFAULT	Yes	AUTO	Yes	AUTO	AUTO

Melt

Melt Stage/Step	Stage 3, Step 3

Target	Multi Peak	Threshold Type	Peak Level (%)	Peak Height
DEFAULT	Yes	Percentage	10	-
U6	Yes	Percentage	10	-

QC Alerts

Curve Quality Alert Enabled	No
Results Quality Alert Enabled	Yes

Advanced

Set the Delta-Rn below which curves will be considered Non-Amplified	No
Primary Analysis Variant	N/A

Relative Quantification Settings

General

RQ Min/Max Calculations	Confidence Level (95.0)
Max Allowed EqCq Mean	40
Include Adjusted EqCq Mean	No
Analysis Type	Singleplex

Endo Controls

Normalization Type	Specific endogenous control

Target	Endogenous Control	Auto	Efficiency(%)
U6	Yes	Yes	AUTO

References

Reference Sample	NC-1

Relative Quantification Results (Sample)

Sample	Target	EqCq Mean	Adjusted EqCq Mean	ΔEqCq Mean	ΔEqCq SD	ΔEqCq SE	ΔΔEqCq	RQ	RQ Min	RQ Max
NC-1	U6	16.942	16.942	-	-	-	-	-	-	-
NC-1	miR-381	20.536	20.536	3.593	0.087	0.05	-	1	0.908	1.101
ALI-4	U6	17.27	17.27	-	-	-	-	-	-	-
ALI-4	miR-381	26.565	26.565	9.296	0.226	0.13	5.702	0.019	0.015	0.025
NC-2	U6	16.884	16.884	-	-	-	-	-	-	-
NC-2	miR-381	20.508	20.508	3.624	0.121	0.07	0.031	0.978	0.856	1.119
ALI-5	U6	17.22	17.22	-	-	-	-	-	-	-
ALI-5	miR-381	26.601	26.601	9.381	0.254	0.146	5.788	0.018	0.014	0.024
NC-3	U6	16.806	16.806	-	-	-	-	-	-	-
NC-3	miR-381	20.65	20.65	3.844	0.304	0.176	0.251	0.84	0.599	1.178
Bud-1	U6	17.281	17.281	-	-	-	-	-	-	-
Bud-1	miR-381	23.749	23.749	6.468	0.158	0.091	2.875	0.136	0.114	0.162
NC-4	U6	17.056	17.056	-	-	-	-	-	-	-
NC-4	miR-381	20.775	20.775	3.719	0.273	0.157	0.126	0.917	0.677	1.241
Bud-2	U6	17.061	17.061	-	-	-	-	-	-	-
Bud-2	miR-381	23.747	23.747	6.686	0.129	0.075	3.093	0.117	0.102	0.135
NC-5	U6	17.207	17.207	-	-	-	-	-	-	-
NC-5	miR-381	21.009	21.009	3.802	0.308	0.178	0.209	0.865	0.614	1.218
Bud-3	U6	17.065	17.065	-	-	-	-	-	-	-
Bud-3	miR-381	23.566	23.566	6.501	0.025	0.014	2.908	0.133	0.13	0.137
ALI-1	U6	17.159	17.159	-	-	-	-	-	-	-
ALI-1	miR-381	25.972	25.972	8.813	0.325	0.188	5.22	0.027	0.019	0.038
Bud-4	U6	17.059	17.059	-	-	-	-	-	-	-
Bud-4	miR-381	23.291	23.291	6.231	0.053	0.03	2.638	0.161	0.152	0.17
ALI-2	U6	17.206	17.206	-	-	-	-	-	-	-
ALI-2	miR-381	26.016	26.016	8.81	0.245	0.142	5.217	0.027	0.02	0.035
Bud-5	U6	17.776	17.776	-	-	-	-	-	-	-
Bud-5	miR-381	23.262	23.262	5.486	1.273	0.735	1.893	0.269	0.065	1.108
ALI-3	U6	17.474	17.474	-	-	-	-	-	-	-

Sample	Target	EqCq Mean	Adjusted EqCq Mean	ΔEqCq Mean	ΔEqCq SD	ΔEqCq SE	ΔΔEqCq	RQ	RQ Min	RQ Max
ALI-3	miR-381	26.223	26.223	8.749	0.3	0.173	5.156	0.028	0.02	0.039
NAC-1	U6	17.103	17.103	-	-	-	-	-	-	-
NAC-1	miR-381	23.445	23.445	6.342	0.58	0.335	2.749	0.149	0.078	0.283

Relative Quantification Plot

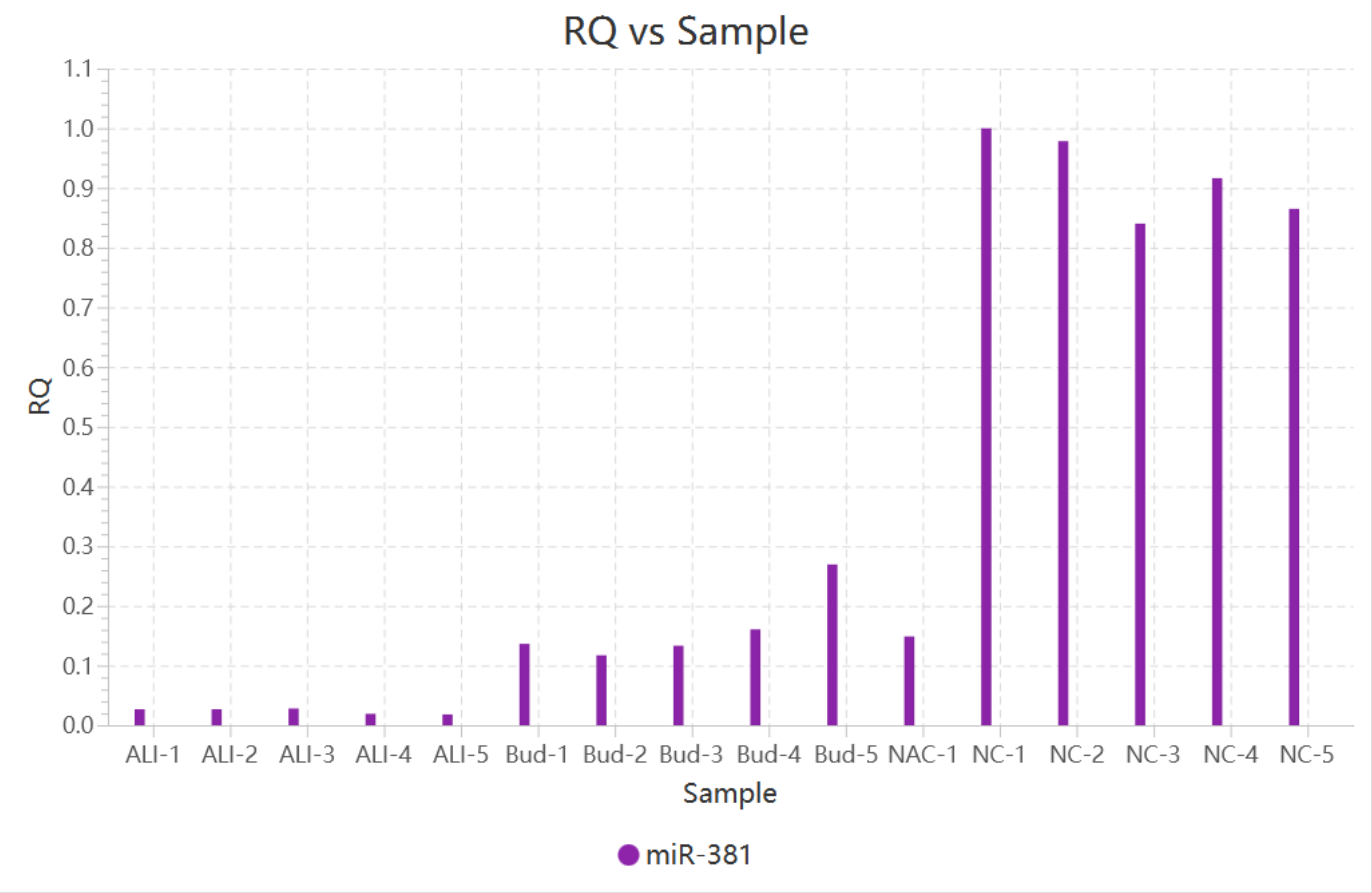

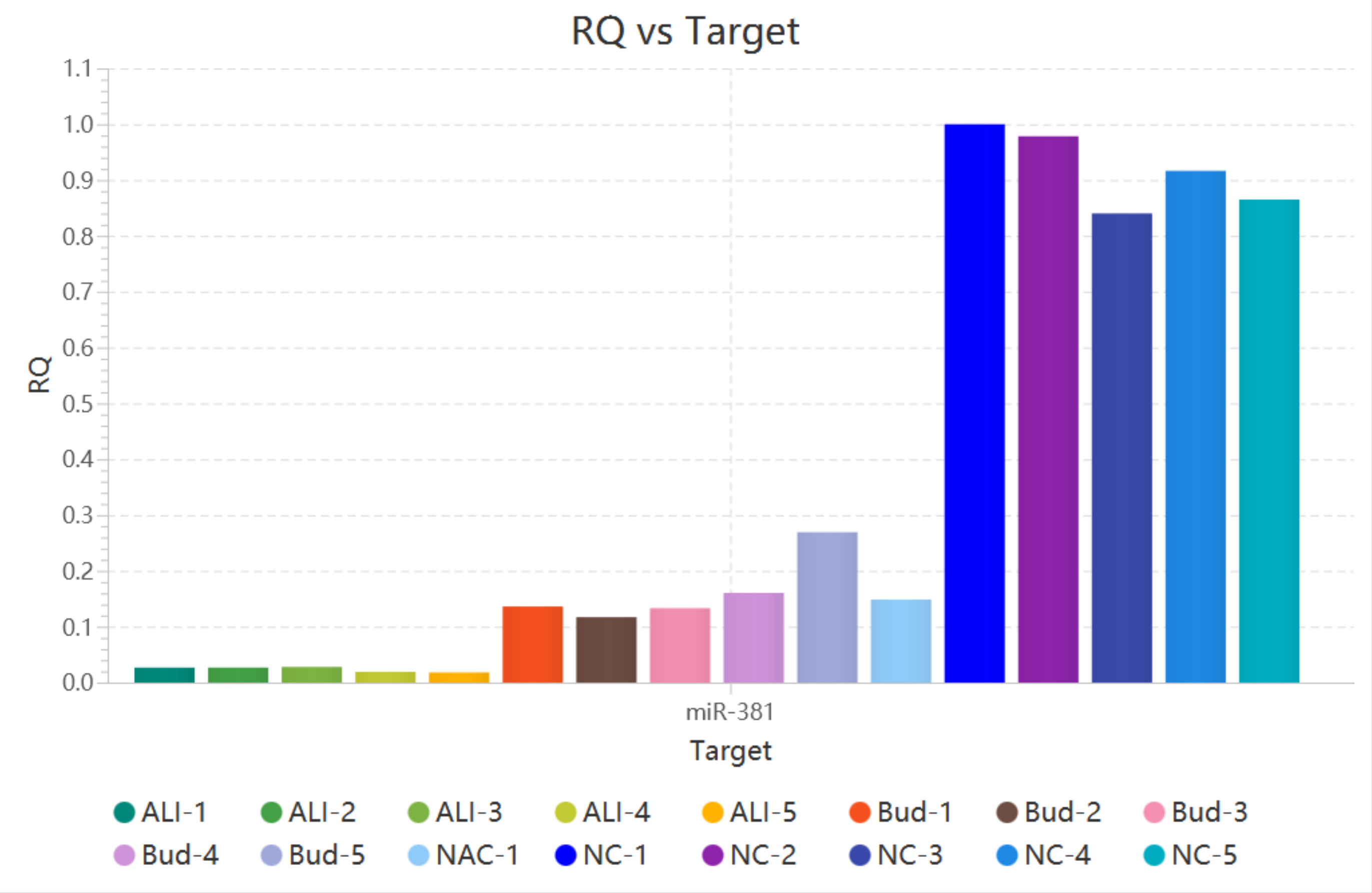

- End of Report -
