# Supplementary material for: Combination therapy with budesonide and acetylcysteine alleviates LPS-induced acute lung injury via the miR-381/NLRP3 molecular axis
Source: PLoS One. 2023 Aug 9;18(8):e0289818. doi: 10.1371/journal.pone.0289818 (PMC10411794; doi:10.1371/journal.pone.0289818)
Supplement: S3 File — (ZIP) [file pone.0289818.s003.zip › S3 File. Fig3 Original data/date/3B/1-2/Results_Report_2023-05-16-113518.pdf]

Plate Results Report

A229-1-2.eds

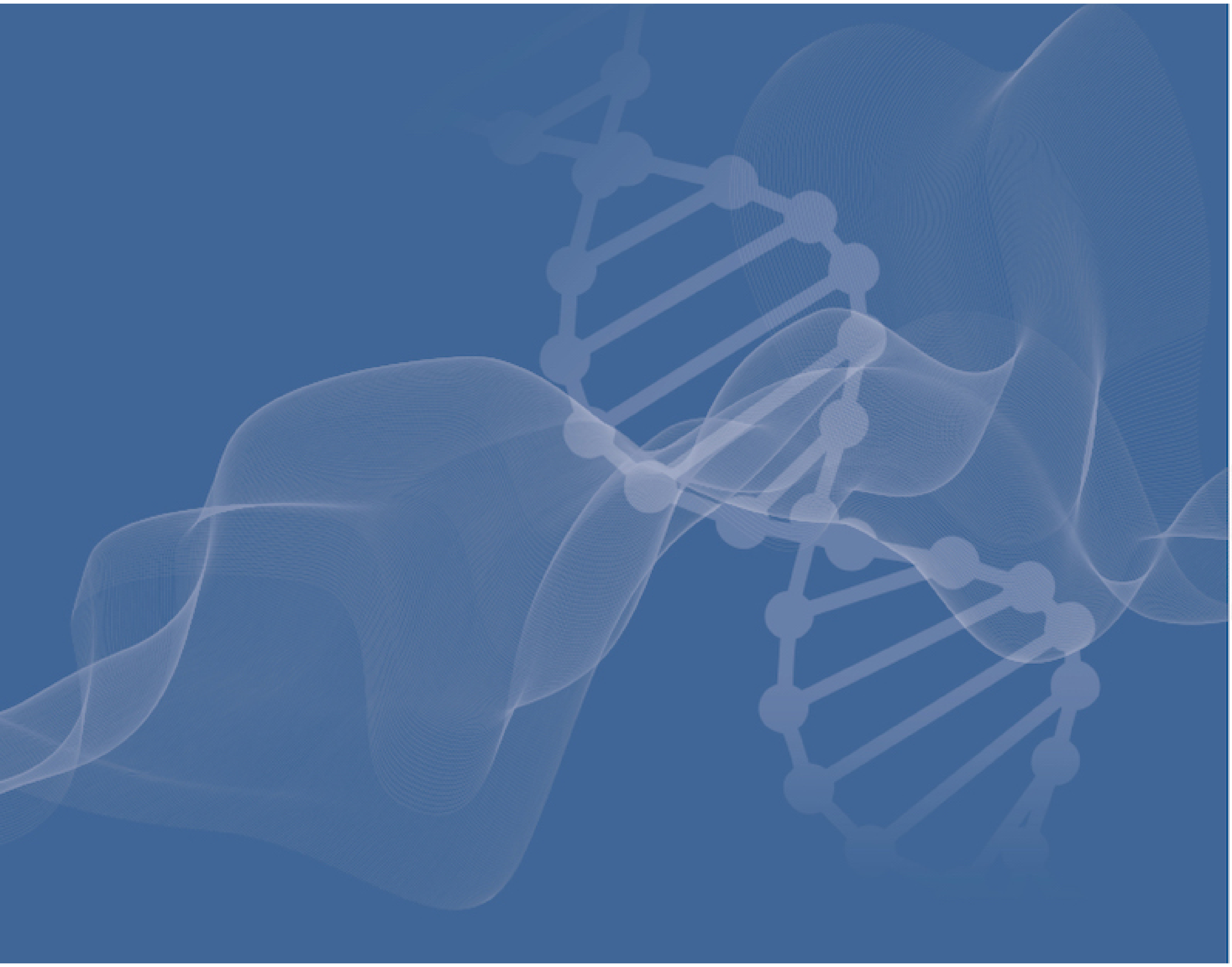

Summary

Property	Details
Bar Code	-
File Name	A229-1-2.eds
Run Start Date/Time	Mar 23, 2022 2:20:58 PM
Run End Date/Time	Mar 23, 2022 3:16:53 PM
Run Duration	55 minutes, and 55 seconds
Operator	DEFAULT
Instrument Name	SVT004
Instrument Type	QuantStudio™ 3 System
Instrument Serial Number	SVT004
Block Type	96-Well 0.2-mL
Block Serial Number	41145627
Heated Cover Serial Number	N/A
PCR Stage/Step Number	Stage 2, Step 2
Melt Stage Number	Stage 3
Quantification Cycle Method	Baseline Threshold
Comment	-
Software Name and Version	Design & Analysis Software v2.6.0
Plugin Name and Version	Primary Analysis v1.7.0, Relative Quantification v1.5.0
Analysis Date/Time	May 16, 2023 11:35:09 AM

Well Table

Well	Sample	Target	Task	Cq	Cq Confidence	Amp Score	Amp Status	Cq Threshold	Baseline Start/End	Melt Temp
A1	NAC-2	U6	Unknown	17.203	0.798	1.879	AMP	1.522	3-12	83.25
A2	NAC-2	U6	Unknown	17.056	0.754	1.887	AMP	1.522	3-12	83.25
A3	NAC-2	U6	Unknown	17.064	0.838	1.881	AMP	1.522	3-12	83.548
A4	NAC-2	miR-381	Unknown	23.621	0.991	1.957	AMP	2.688	3-16	82.654
A5	NAC-2	miR-381	Unknown	23.694	0.992	1.962	AMP	2.688	3-14	82.503
A6	NAC-2	miR-381	Unknown	23.679	0.994	1.968	AMP	2.688	3-15	82.652
A7	Bud+NAC-5	U6	Unknown	17.336	0.899	1.893	AMP	1.522	3-13	83.398
A8	Bud+NAC-5	U6	Unknown	17.18	0.941	1.891	AMP	1.522	3-13	83.398
A9	Bud+NAC-5	U6	Unknown	17.55	0.845	1.879	AMP	1.522	3-13	83.399
A10	Bud+NAC-5	miR-381	Unknown	21.368	0.984	1.938	AMP	2.688	3-13	82.504
A11	Bud+NAC-5	miR-381	Unknown	21.408	0.991	1.937	AMP	2.688	3-12	82.951
A12	Bud+NAC-5	miR-381	Unknown	21.516	0.991	1.927	AMP	2.688	3-13	83.1
B1	NAC-3	U6	Unknown	16.878	0.776	1.899	AMP	1.522	3-13	83.25
B2	NAC-3	U6	Unknown	17.229	0.924	1.913	AMP	1.522	3-13	83.101
B3	NAC-3	U6	Unknown	17.094	0.82	1.895	AMP	1.522	3-13	83.399
B4	NAC-3	miR-381	Unknown	23.856	0.989	1.962	AMP	2.688	3-17	82.505
B5	NAC-3	miR-381	Unknown	23.915	0.991	1.952	AMP	2.688	3-14	82.503
B6	NAC-3	miR-381	Unknown	23.882	0.994	1.954	AMP	2.688	3-16	82.503
C1	NAC-4	U6	Unknown	17.095	0.847	1.922	AMP	1.522	3-12	83.101
C2	NAC-4	U6	Unknown	17.375	0.859	1.91	AMP	1.522	3-12	83.101
C3	NAC-4	U6	Unknown	17.332	0.77	1.907	AMP	1.522	3-13	83.25
C4	NAC-4	miR-381	Unknown	23.988	0.988	1.949	AMP	2.688	3-17	82.355
C5	NAC-4	miR-381	Unknown	24.073	0.99	1.945	AMP	2.688	3-15	82.354
C6	NAC-4	miR-381	Unknown	24.163	0.988	1.932	AMP	2.688	3-15	82.354
D1	NAC-5	U6	Unknown	17.152	0.932	1.925	AMP	1.522	3-13	82.952
D2	NAC-5	U6	Unknown	17.262	0.838	1.929	AMP	1.522	3-12	82.952
D3	NAC-5	U6	Unknown	17.34	0.797	1.92	AMP	1.522	3-12	83.25
D4	NAC-5	miR-381	Unknown	24.261	0.99	1.931	AMP	2.688	3-16	82.355
D5	NAC-5	miR-381	Unknown	24.233	0.986	1.923	AMP	2.688	3-17	82.354
D6	NAC-5	miR-381	Unknown	24.337	0.99	1.918	AMP	2.688	3-16	82.354

Well	Sample	Target	Task	Cq	Cq Confidence	Amp Score	Amp Status	Cq Threshold	Baseline Start/End	Melt Temp
E1	Bud+NAC-1	U6	Unknown	16.974	0.918	1.921	AMP	1.522	3-12	83.101
E2	Bud+NAC-1	U6	Unknown	17.513	0.831	1.919	AMP	1.522	3-13	82.803
E3	Bud+NAC-1	U6	Unknown	17.522	0.763	1.905	AMP	1.522	3-13	83.101
E4	Bud+NAC-1	miR-381	Unknown	21.905	0.992	1.932	AMP	2.688	3-14	82.355
E5	Bud+NAC-1	miR-381	Unknown	21.986	0.992	1.922	AMP	2.688	3-14	82.354
E6	Bud+NAC-1	miR-381	Unknown	22.068	0.989	1.924	AMP	2.688	3-14	82.354
F1	Bud+NAC-2	U6	Unknown	17.567	0.807	1.913	AMP	1.522	3-12	82.952
F2	Bud+NAC-2	U6	Unknown	17.612	0.873	1.938	AMP	1.522	3-13	82.803
F3	Bud+NAC-2	U6	Unknown	17.609	0.885	1.912	AMP	1.522	3-13	83.101
F4	Bud+NAC-2	miR-381	Unknown	21.747	0.991	1.949	AMP	2.688	3-12	82.355
F5	Bud+NAC-2	miR-381	Unknown	21.838	0.992	1.945	AMP	2.688	3-13	82.503
F6	Bud+NAC-2	miR-381	Unknown	21.666	0.98	1.951	AMP	2.688	3-14	82.503
G1	Bud+NAC-3	U6	Unknown	17.377	0.775	1.891	AMP	1.522	3-12	82.952
G2	Bud+NAC-3	U6	Unknown	17.513	0.894	1.893	AMP	1.522	3-13	82.952
G3	Bud+NAC-3	U6	Unknown	17.313	0.817	1.924	AMP	1.522	3-12	83.25
G4	Bud+NAC-3	miR-381	Unknown	21.502	0.985	1.962	AMP	2.688	3-13	82.505
G5	Bud+NAC-3	miR-381	Unknown	21.597	0.984	1.95	AMP	2.688	3-15	82.503
G6	Bud+NAC-3	miR-381	Unknown	21.582	0.982	1.956	AMP	2.688	3-13	82.503
H1	Bud+NAC-4	U6	Unknown	17.35	0.83	1.857	AMP	1.522	3-13	83.101
H2	Bud+NAC-4	U6	Unknown	17.212	0.835	1.915	AMP	1.522	3-12	82.952
H3	Bud+NAC-4	U6	Unknown	17.324	0.81	1.913	AMP	1.522	3-12	83.399
H4	Bud+NAC-4	miR-381	Unknown	21.327	0.988	1.97	AMP	2.688	3-13	82.505
H5	Bud+NAC-4	miR-381	Unknown	21.464	0.99	1.965	AMP	2.688	3-14	82.652
H6	Bud+NAC-4	miR-381	Unknown	21.651	0.989	1.949	AMP	2.688	3-14	82.652

Replicate Group Table

Sample	Target	No. of Replicates	Cq Mean	Cq SD
Bud+NAC-1	U6	3	17.336	0.314
Bud+NAC-1	miR-381	3	21.986	0.081
Bud+NAC-2	U6	3	17.596	0.025
Bud+NAC-2	miR-381	3	21.75	0.086
Bud+NAC-3	U6	3	17.401	0.102
Bud+NAC-3	miR-381	3	21.56	0.051
Bud+NAC-4	U6	3	17.296	0.074
Bud+NAC-4	miR-381	3	21.48	0.163
Bud+NAC-5	U6	3	17.355	0.186
Bud+NAC-5	miR-381	3	21.431	0.076
NAC-2	U6	3	17.108	0.083
NAC-2	miR-381	3	23.665	0.039
NAC-3	U6	3	17.067	0.177
NAC-3	miR-381	3	23.884	0.03
NAC-4	U6	3	17.267	0.151
NAC-4	miR-381	3	24.075	0.088
NAC-5	U6	3	17.251	0.094
NAC-5	miR-381	3	24.277	0.054

Plate Layout

	1	2	3	4	5	6	7	8	9	10	11	12
A	NAC-2<br>U6 (17.203)	NAC-2<br>U6 (17.056)	NAC-2<br>U6 (17.064)	NAC-2<br>miR-381 (23.621)	NAC-2<br>miR-381 (23.694)	NAC-2<br>miR-381 (23.679)	Bud+NAC-5<br>U6 (17.336)	Bud+NAC-5<br>U6 (17.18)	Bud+NAC-5<br>U6 (17.55)	Bud+NAC-5<br>miR-381 (21.368)	Bud+NAC-5<br>miR-381 (21.408)	Bud+NAC-5<br>miR-381 (21.516)
B	NAC-3<br>U6 (16.878)	NAC-3<br>U6 (17.229)	NAC-3<br>U6 (17.094)	NAC-3<br>miR-381 (23.856)	NAC-3<br>miR-381 (23.915)	NAC-3<br>miR-381 (23.882)						
C	NAC-4<br>U6 (17.095)	NAC-4<br>U6 (17.375)	NAC-4<br>U6 (17.332)	NAC-4<br>miR-381 (23.988)	NAC-4<br>miR-381 (24.073)	NAC-4<br>miR-381 (24.163)						
D	NAC-5<br>U6 (17.152)	NAC-5<br>U6 (17.262)	NAC-5<br>U6 (17.34)	NAC-5<br>miR-381 (24.261)	NAC-5<br>miR-381 (24.233)	NAC-5<br>miR-381 (24.337)						
E	Bud+NAC-1<br>U6 (16.974)	Bud+NAC-1<br>U6 (17.513)	Bud+NAC-1<br>U6 (17.522)	Bud+NAC-1<br>miR-381 (21.905)	Bud+NAC-1<br>miR-381 (21.986)	Bud+NAC-1<br>miR-381 (22.068)						
F	Bud+NAC-2<br>U6 (17.567)	Bud+NAC-2<br>U6 (17.612)	Bud+NAC-2<br>U6 (17.609)	Bud+NAC-2<br>miR-381 (21.747)	Bud+NAC-2<br>miR-381 (21.838)	Bud+NAC-2<br>miR-381 (21.666)						
G	Bud+NAC-3<br>U6 (17.377)	Bud+NAC-3<br>U6 (17.513)	Bud+NAC-3<br>U6 (17.313)	Bud+NAC-3<br>miR-381 (21.502)	Bud+NAC-3<br>miR-381 (21.597)	Bud+NAC-3<br>miR-381 (21.582)						
H	Bud+NAC-4<br>U6 (17.35)	Bud+NAC-4<br>U6 (17.212)	Bud+NAC-4<br>U6 (17.324)	Bud+NAC-4<br>miR-381 (21.327)	Bud+NAC-4<br>miR-381 (21.464)	Bud+NAC-4<br>miR-381 (21.651)						

Amplification Plot (dRn)

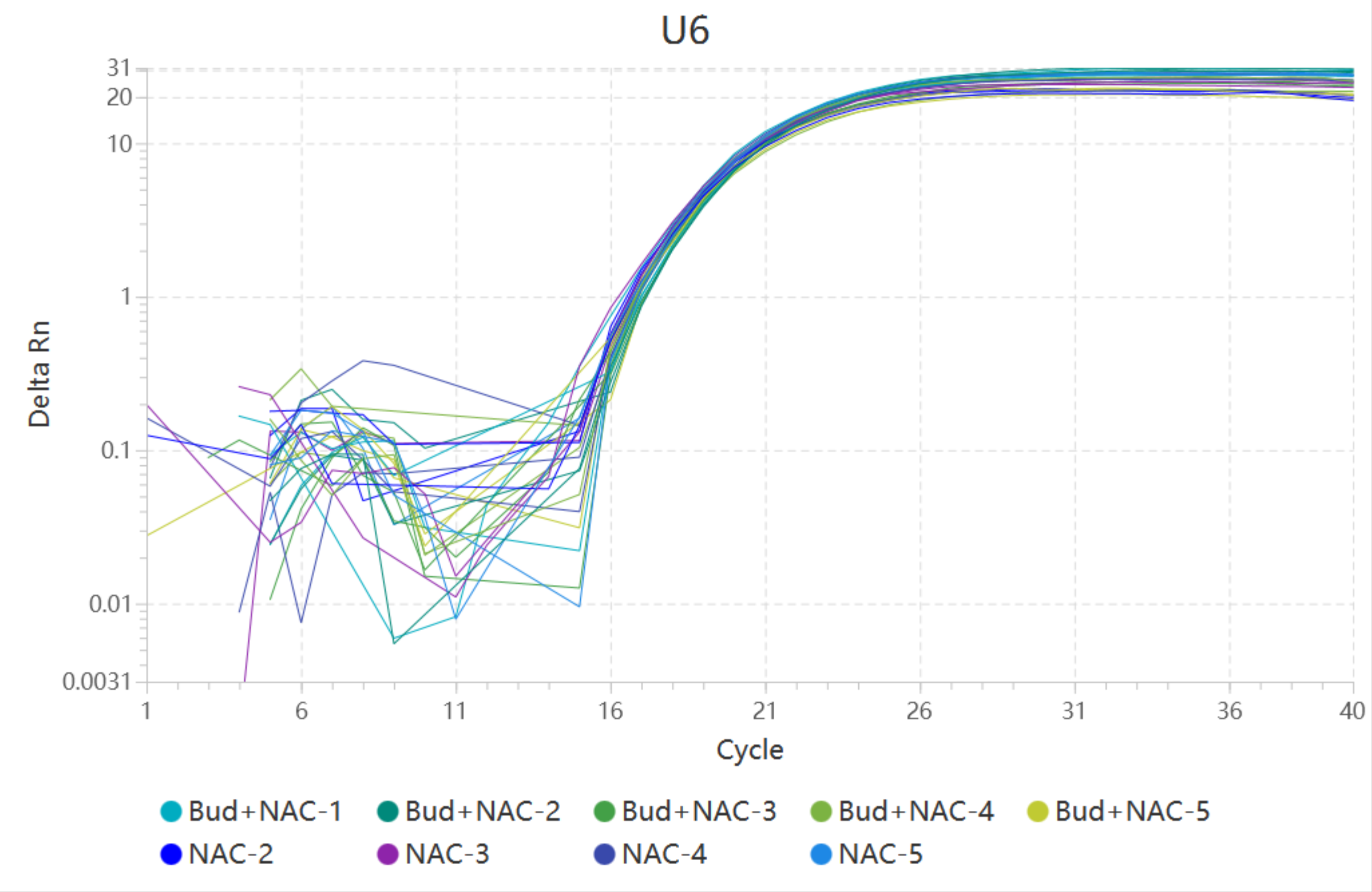

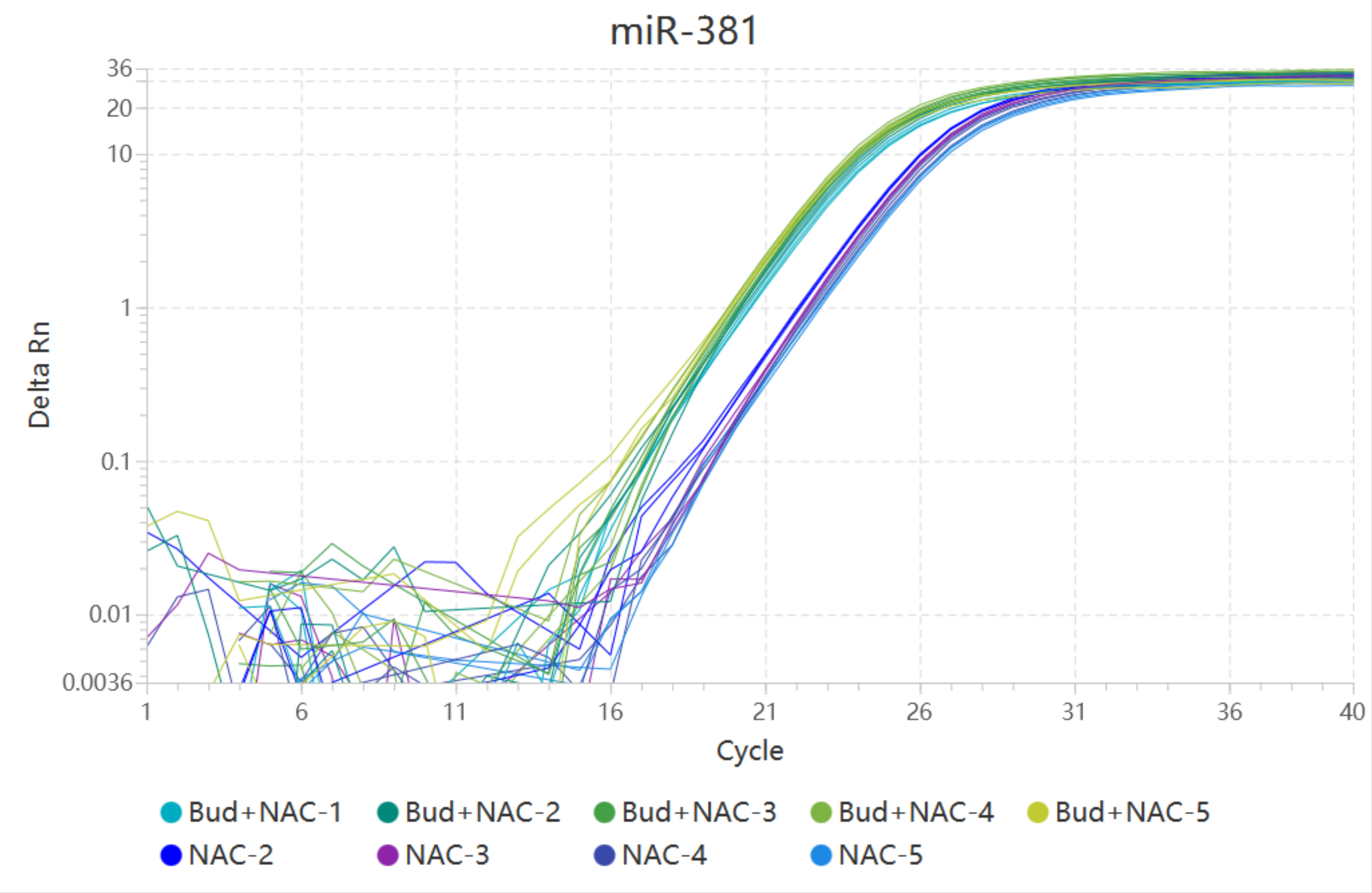

Amplification Plot (Rn)

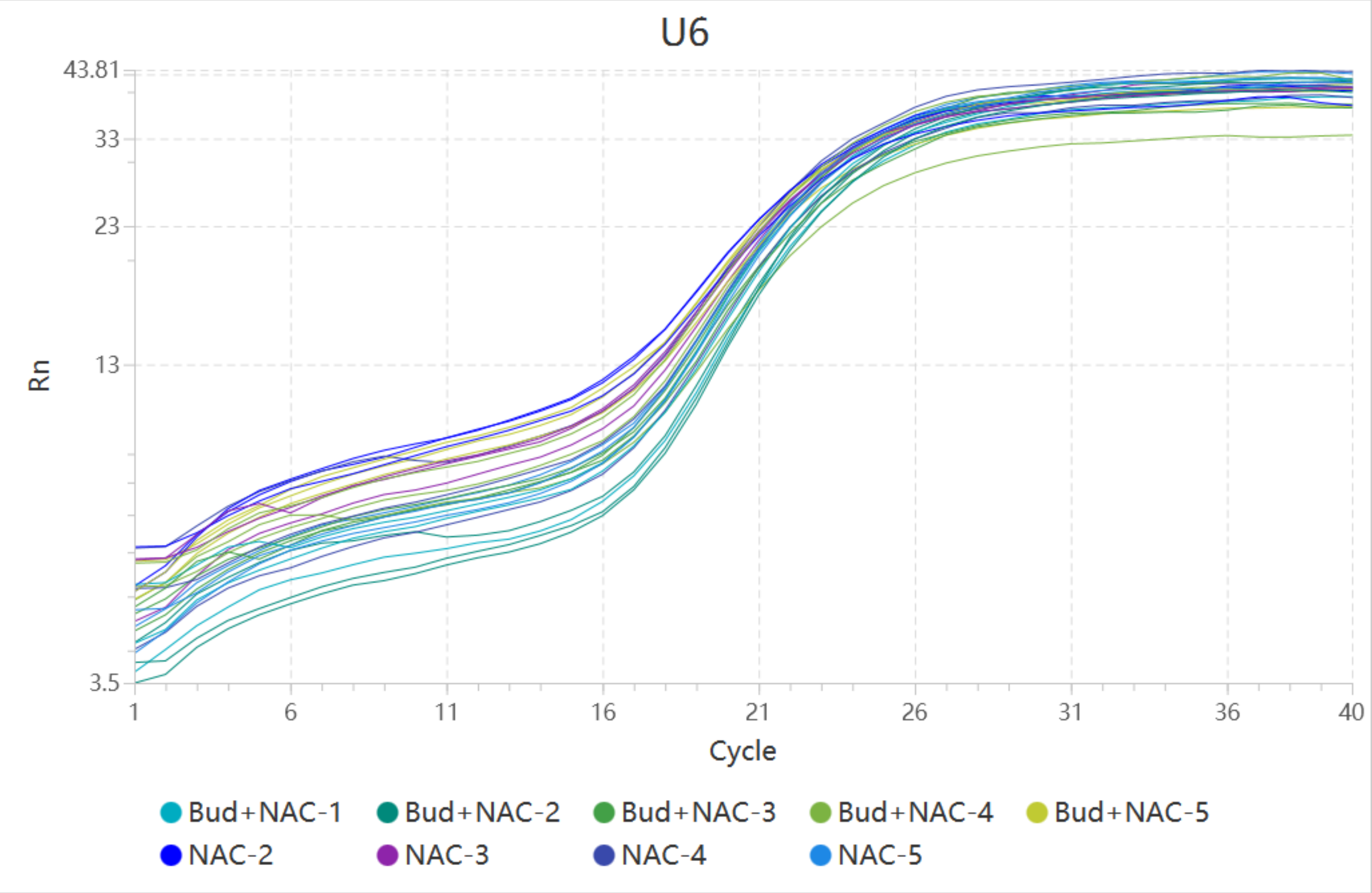

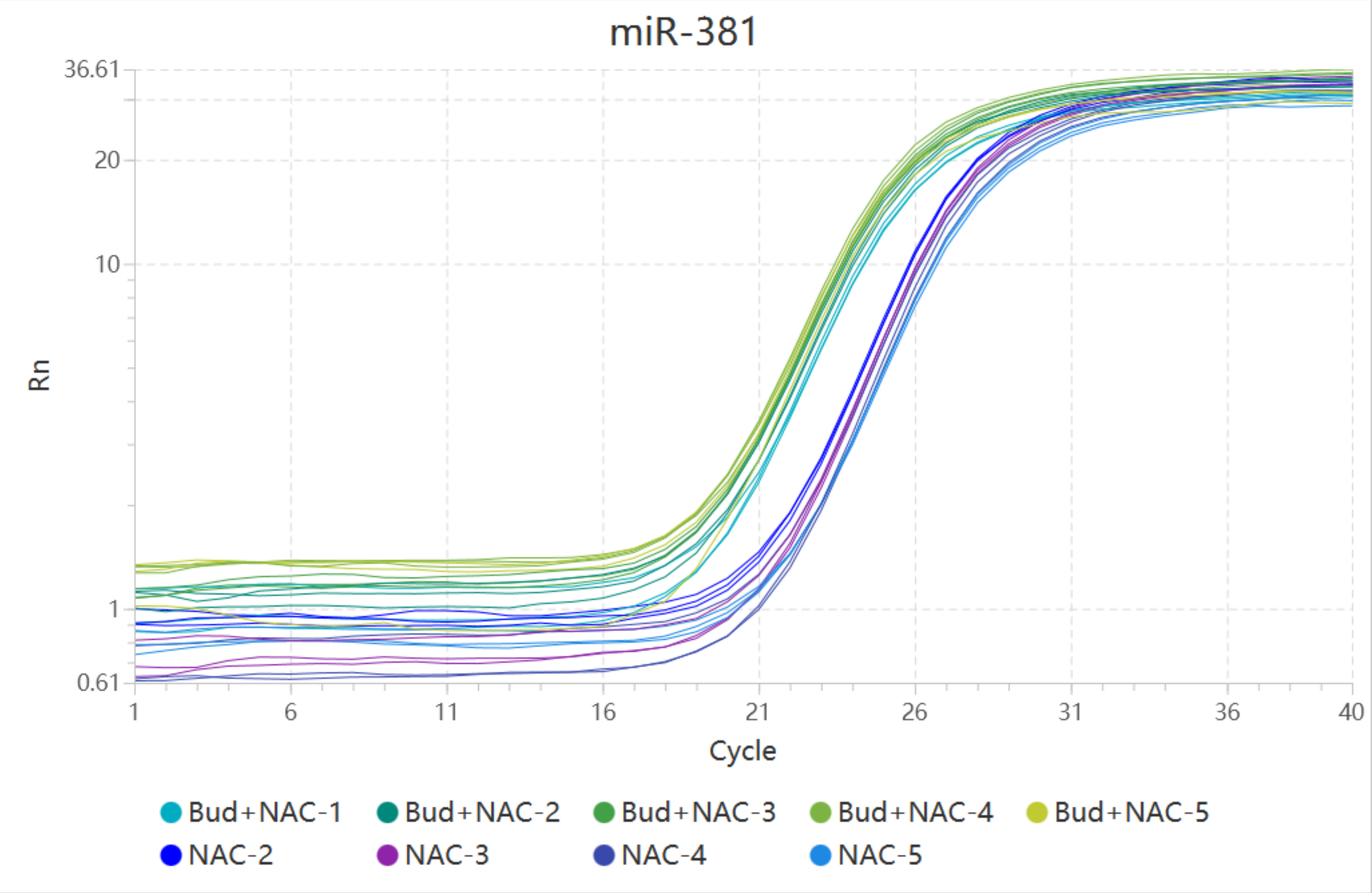

Melt Curve Plot

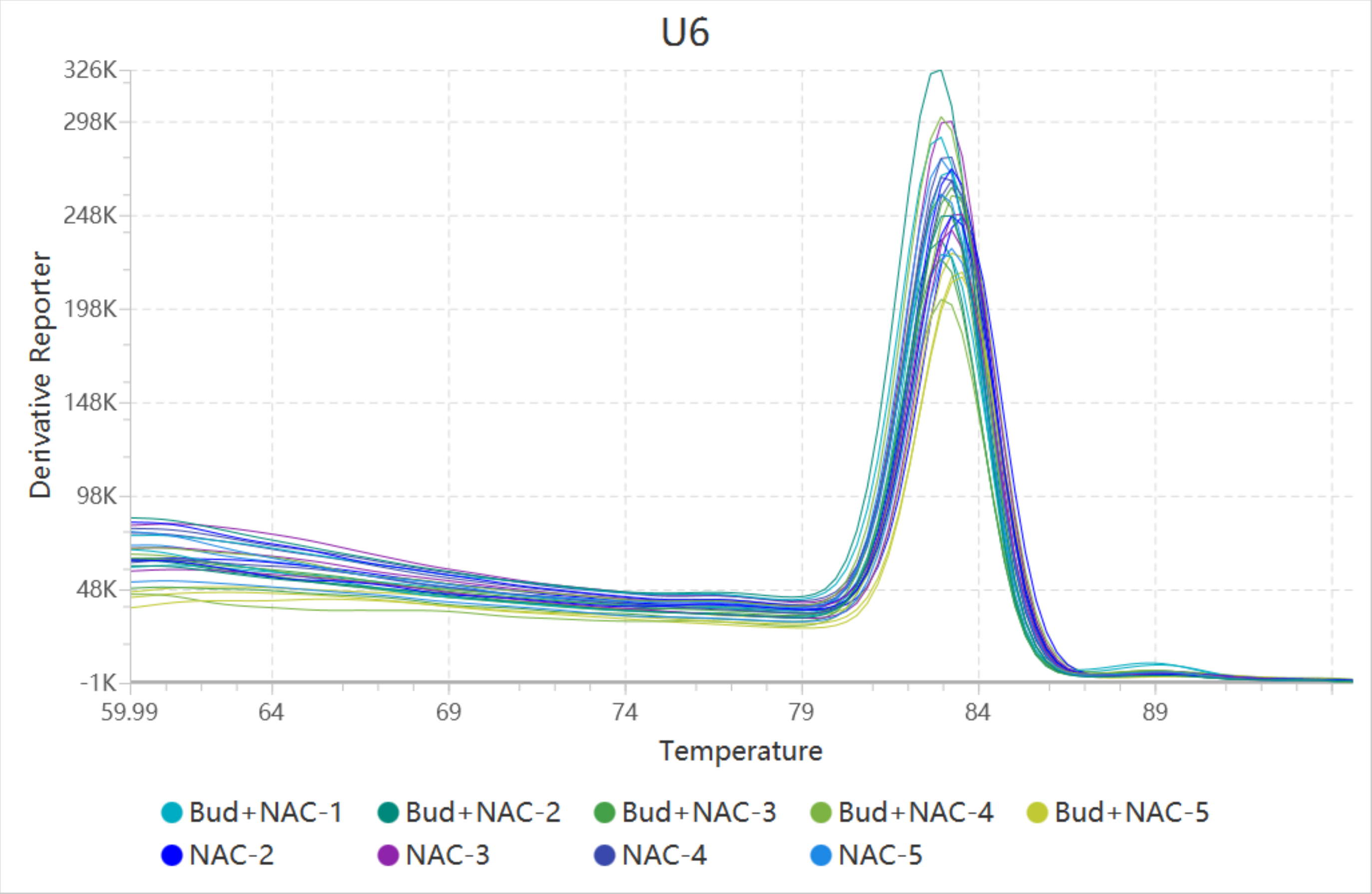

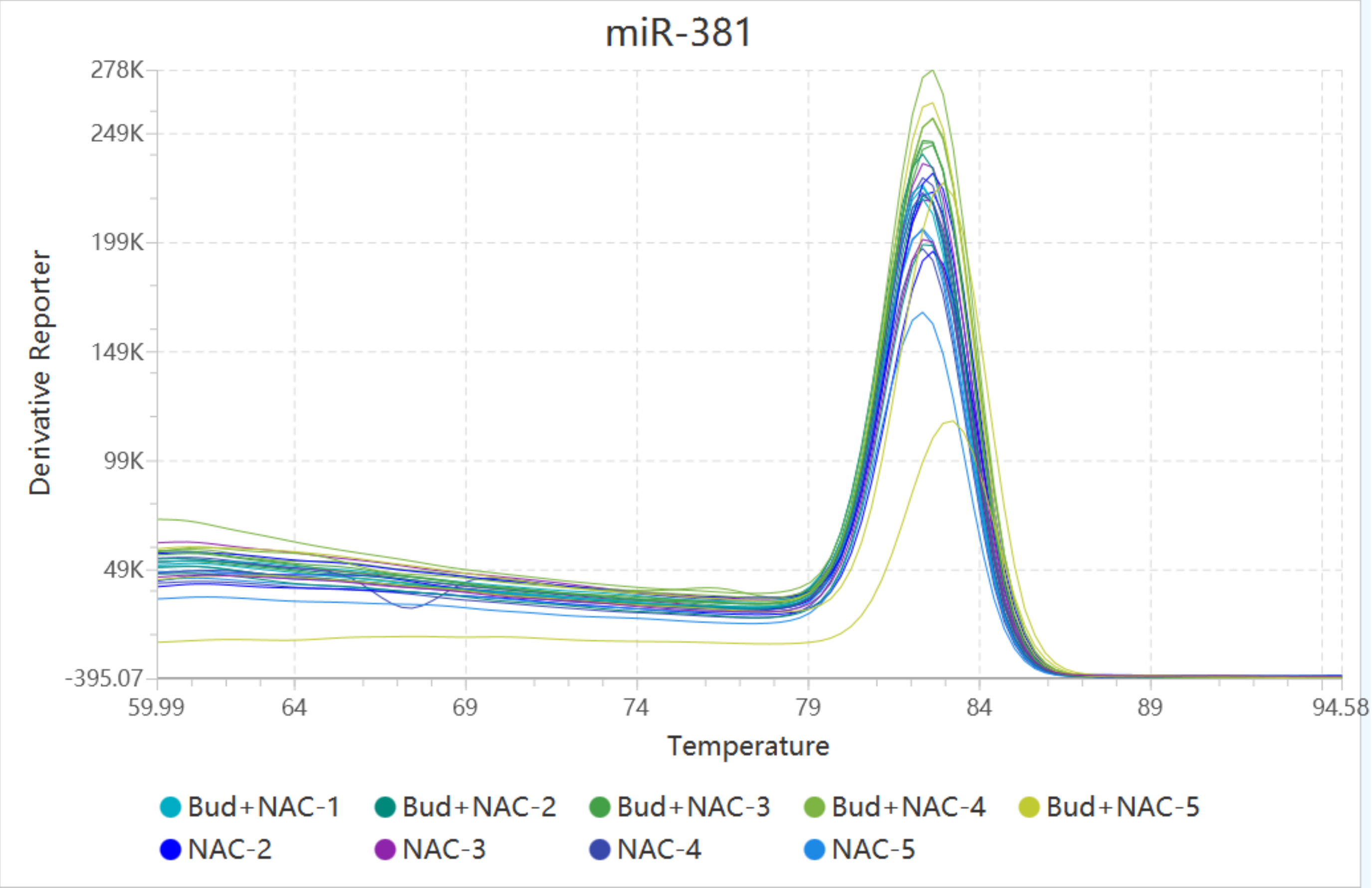

Run Method

Block Type	96-Well 0.2-mL Block
Sample Volume	20.0
Cover Temperature	105.0
Run mode	FAST

Stage	Collection Flag	Ramp Rate	Temperature	Hold Time	Starting Cycle	Auto Delta Temperature	Auto Delta Hold Time
Hold Stage	false	2.74°C/sec	95.0°C	20	-	-	-
PCR Stage (40 cycles)	false	2.74°C/sec	95.0°C	1	-	-	-
	true	2.12°C/sec	60.0°C	20	-	-	-
Melt Stage	false	2.74°C/sec	95.0°C	1	-	-	-
	false	2.12°C/sec	60.0°C	20	-	-	-
	true	0.15°C/sec	95.0°C	1	-	-	-

Primary Analysis Settings

General

PCR Stage/Step	Stage 2, Step 2
Quantification Cycle Method	Baseline Threshold

Target	Auto Threshold	Threshold	Auto Baseline	Baseline Start	Baseline End
DEFAULT	Yes	AUTO	Yes	AUTO	AUTO

Melt

Melt Stage/Step	Stage 3, Step 3

Target	Multi Peak	Threshold Type	Peak Level (%)	Peak Height
DEFAULT	Yes	Percentage	10	-
U6	Yes	Percentage	10	-

QC Alerts

Curve Quality Alert Enabled	No
Results Quality Alert Enabled	Yes

Advanced

Set the Delta-Rn below which curves will be considered Non-Amplified	No
Primary Analysis Variant	N/A

Relative Quantification Settings

General

RQ Min/Max Calculations	Confidence Level (95.0)
Max Allowed EqCq Mean	40
Include Adjusted EqCq Mean	No
Analysis Type	Singleplex

Endo Controls

Normalization Type	Specific endogenous control

Target	Endogenous Control	Auto	Efficiency(%)
U6	Yes	Yes	AUTO

References

Reference Sample	NAC-2

Relative Quantification Results (Sample)

Sample	Target	EqCq Mean	Adjusted EqCq Mean	ΔEqCq Mean	ΔEqCq SD	ΔEqCq SE	ΔΔEqCq	RQ	RQ Min	RQ Max
NAC-2	U6	17.108	17.108	-	-	-	-	-	-	-
NAC-2	miR-381	23.665	23.665	6.557	0.091	0.053	-	1	0.904	1.107
Bud+NAC-5	U6	17.355	17.355	-	-	-	-	-	-	-
Bud+NAC-5	miR-381	21.431	21.431	4.075	0.201	0.116	-2.482	5.587	4.468	6.986
NAC-3	U6	17.067	17.067	-	-	-	-	-	-	-
NAC-3	miR-381	23.884	23.884	6.817	0.18	0.104	0.26	0.835	0.684	1.02
NAC-4	U6	17.267	17.267	-	-	-	-	-	-	-
NAC-4	miR-381	24.075	24.075	6.808	0.174	0.101	0.25	0.841	0.693	1.021
NAC-5	U6	17.251	17.251	-	-	-	-	-	-	-
NAC-5	miR-381	24.277	24.277	7.026	0.109	0.063	0.468	0.723	0.641	0.815
Bud+NAC-1	U6	17.336	17.336	-	-	-	-	-	-	-
Bud+NAC-1	miR-381	21.986	21.986	4.65	0.324	0.187	-1.907	3.752	2.617	5.379
Bud+NAC-2	U6	17.596	17.596	-	-	-	-	-	-	-
Bud+NAC-2	miR-381	21.75	21.75	4.154	0.09	0.052	-2.403	5.29	4.789	5.843
Bud+NAC-3	U6	17.401	17.401	-	-	-	-	-	-	-
Bud+NAC-3	miR-381	21.56	21.56	4.16	0.114	0.066	-2.398	5.27	4.643	5.982
Bud+NAC-4	U6	17.296	17.296	-	-	-	-	-	-	-
Bud+NAC-4	miR-381	21.48	21.48	4.185	0.178	0.103	-2.373	5.179	4.247	6.315

Relative Quantification Plot

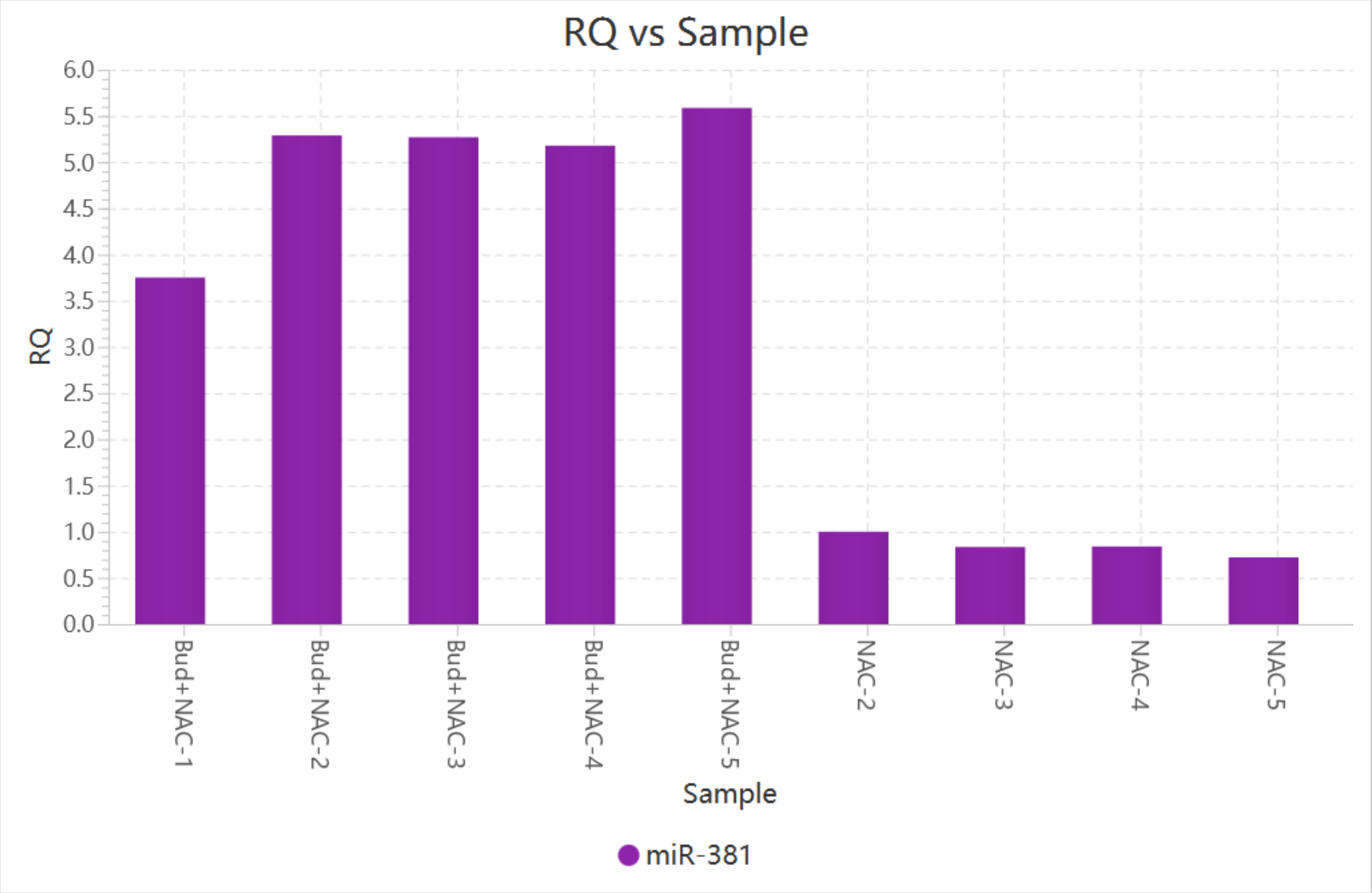

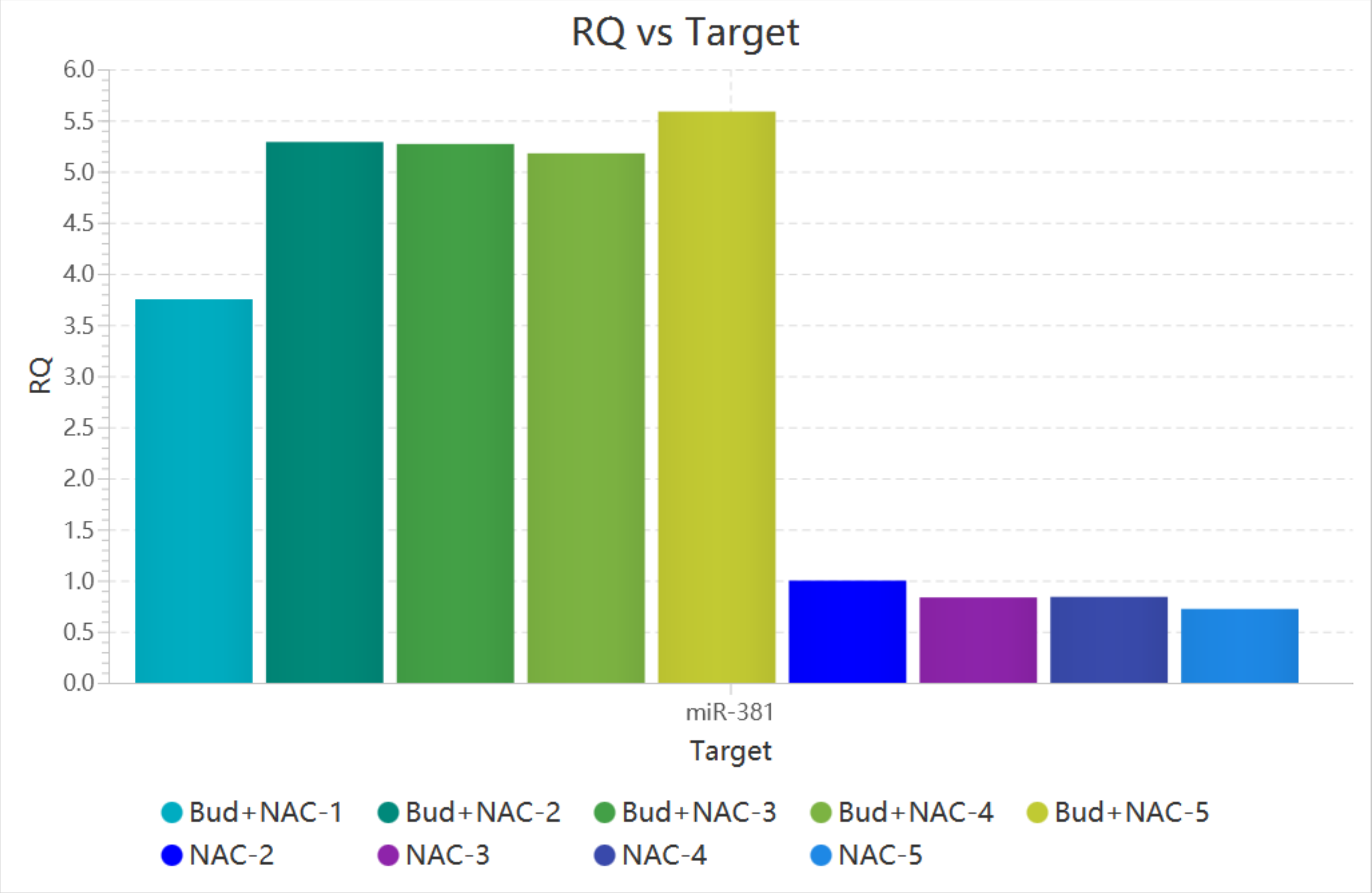

- End of Report -
