# Supplementary material for: Combination therapy with budesonide and acetylcysteine alleviates LPS-induced acute lung injury via the miR-381/NLRP3 molecular axis
Source: PLoS One. 2023 Aug 9;18(8):e0289818. doi: 10.1371/journal.pone.0289818 (PMC10411794; doi:10.1371/journal.pone.0289818)
Supplement: S3 File — (ZIP) [file pone.0289818.s003.zip › S3 File. Fig3 Original data/date/3C/Results_Report_2023-05-16-114331.pdf]

Plate Results Report

A113-3.eds

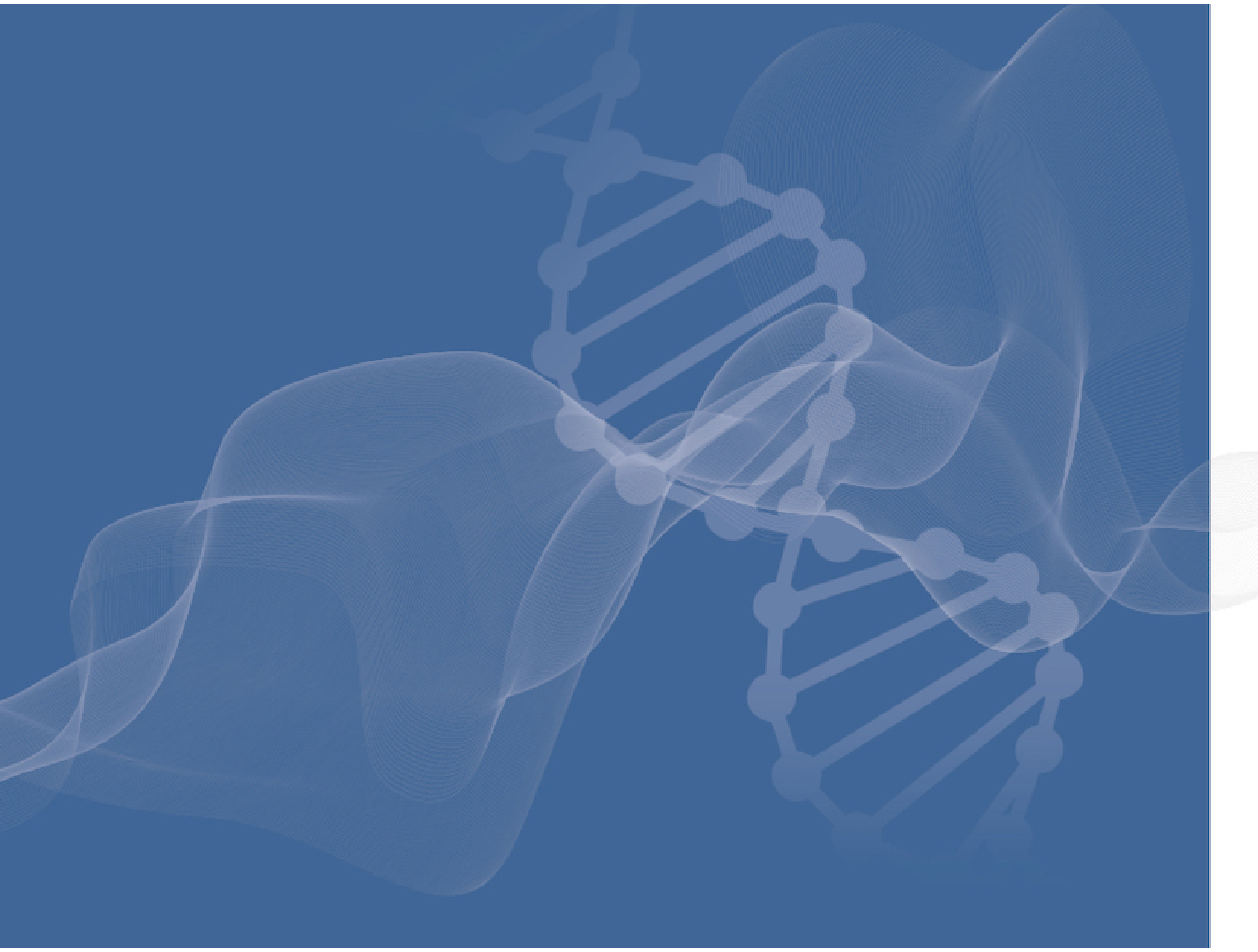

Summary

Property	Details
Bar Code	-
File Name	A113-3.eds
Run Start Date/Time	Jan 17, 2022 11:11:06 PM
Run End Date/Time	Jan 18, 2022 12:07:50 AM
Run Duration	56 minutes, and 44 seconds
Operator	DEFAULT
Instrument Name	SVT004
Instrument Type	QuantStudio™ 3 System
Instrument Serial Number	SVT004
Block Type	96-Well 0.2-mL
Block Serial Number	41145627
Heated Cover Serial Number	N/A
PCR Stage/Step Number	Stage 2, Step 2
Melt Stage Number	Stage 3
Quantification Cycle Method	Baseline Threshold
Comment	-
Software Name and Version	Design & Analysis Software v2.6.0
Plugin Name and Version	Primary Analysis v1.7.0, Relative Quantification v1.5.0
Analysis Date/Time	May 16, 2023 11:43:23 AM

Well Table

Well	Sample	Target	Task	Cq	Cq Confi dence	Amp Score	Amp Status	Cq Thres hold	Baseline Start/End	Melt Temp
A1	NC-1	U6	Unknown	18.826	0.989	1.833	AMP	1.861	3-11	82.658
A2	NC-1	U6	Unknown	18.386	0.989	1.861	AMP	1.861	3-10	82.807
A3	NC-1	U6	Unknown	18.403	0.991	1.858	AMP	1.861	3-11	82.954
A4	NC-1	miR-381	Unknown	22.227	0.992	1.85	AMP	1.633	3-14	77.736
A5	NC-1	miR-381	Unknown	21.785	0.993	1.885	AMP	1.633	3-14	78.034
A6	NC-1	miR-381	Unknown	21.902	0.995	1.871	AMP	1.633	3-13	78.034
A7	miR-381 mimic-3	U6	Unknown	17.992	0.994	1.873	AMP	1.861	3-11	83.103
A8	miR-381 mimic-3	U6	Unknown	17.884	0.992	1.874	AMP	1.861	3-10	83.103
A9	miR-381 mimic-3	U6	Unknown	17.804	0.992	1.872	AMP	1.861	3-11	83.252
A10	miR-381 mimic-3	miR-381	Unknown	19.938	0.992	1.857	AMP	1.633	3-10	77.885
A11	miR-381 mimic-3	miR-381	Unknown	19.883	0.991	1.855	AMP	1.633	3-11	78.036
A12	miR-381 mimic-3	miR-381	Unknown	19.966	0.99	1.828	AMP	1.633	3-12	78.334
B1	NC-2	U6	Unknown	18.301	0.989	1.849	AMP	1.861	3-12	82.956
B2	NC-2	U6	Unknown	18.296	0.991	1.867	AMP	1.861	3-12	82.807
B3	NC-2	U6	Unknown	18.299	0.99	1.86	AMP	1.861	3-10	82.805
B4	NC-2	miR-381	Unknown	21.874	0.995	1.858	AMP	1.633	3-13	77.736
B5	NC-2	miR-381	Unknown	22.009	0.991	1.865	AMP	1.633	3-14	77.885
B6	NC-2	miR-381	Unknown	21.752	0.994	1.875	AMP	1.633	3-13	77.885
C1	NC-3	U6	Unknown	18.308	0.987	1.855	AMP	1.861	3-9	82.658
C2	NC-3	U6	Unknown	18.253	0.988	1.867	AMP	1.861	3-10	82.807
C3	NC-3	U6	Unknown	18.127	0.991	1.868	AMP	1.861	3-11	82.954
C4	NC-3	miR-381	Unknown	21.974	0.991	1.852	AMP	1.633	3-14	77.736
C5	NC-3	miR-381	Unknown	21.793	0.989	1.861	AMP	1.633	3-14	77.885
C6	NC-3	miR-381	Unknown	21.76	0.991	1.856	AMP	1.633	3-14	77.885
D1	miR-381 inhi-1	U6	Unknown	18.114	0.99	1.873	AMP	1.861	3-11	83.105
D2	miR-381 inhi-1	U6	Unknown	18.198	0.991	1.873	AMP	1.861	3-11	82.956
D3	miR-381 inhi-1	U6	Unknown	18.256	0.983	1.873	AMP	1.861	3-13	82.805
D4	miR-381 inhi-1	miR-381	Unknown	23.085	0.979	1.843	AMP	1.633	3-16	77.736
D5	miR-381 inhi-1	miR-381	Unknown	23	0.992	1.859	AMP	1.633	3-15	77.885

Well	Sample	Target	Task	Cq	Cq Confidence	Amp Score	Amp Status	Cq Threshold	Baseline Start/End	Melt Temp
D6	miR-381 inhi-1	miR-381	Unknown	23.012	0.987	1.854	AMP	1.633	3-15	77.885
E1	miR-381 inhi-2	U6	Unknown	18.221	0.987	1.854	AMP	1.861	3-10	82.956
E2	miR-381 inhi-2	U6	Unknown	17.995	0.995	1.871	AMP	1.861	3-10	83.105
E3	miR-381 inhi-2	U6	Unknown	18.028	0.988	1.858	AMP	1.861	3-9	82.954
E4	miR-381 inhi-2	miR-381	Unknown	22.839	0.993	1.86	AMP	1.633	3-14	77.885
E5	miR-381 inhi-2	miR-381	Unknown	22.931	0.991	1.848	AMP	1.633	3-15	77.885
E6	miR-381 inhi-2	miR-381	Unknown	22.767	0.99	1.872	AMP	1.633	3-15	78.034
F1	miR-381 inhi- 3	U6	Unknown	18.036	0.987	1.877	AMP	1.861	3-10	83.403
F2	miR-381 inhi- 3	U6	Unknown	18.202	0.983	1.866	AMP	1.861	3-11	83.105
F3	miR-381 inhi- 3	U6	Unknown	17.655	0.99	1.872	AMP	1.861	3-11	82.954
F4	miR-381 inhi- 3	miR-381	Unknown	22.617	0.994	1.874	AMP	1.633	3-14	77.885
F5	miR-381 inhi- 3	miR-381	Unknown	22.565	0.993	1.883	AMP	1.633	3-15	78.034
F6	miR-381 inhi- 3	miR-381	Unknown	22.481	0.989	1.885	AMP	1.633	3-15	78.034
G1	miR-381 mimic-1	U6	Unknown	17.866	0.985	1.85	AMP	1.861	3-12	83.552
G2	miR-381 mimic-1	U6	Unknown	17.792	0.991	1.869	AMP	1.861	3-9	83.254
G3	miR-381 mimic-1	U6	Unknown	17.949	0.991	1.873	AMP	1.861	3-12	82.954
G4	miR-381 mimic-1	miR-381	Unknown	19.78	0.957	1.849	AMP	1.633	3-13	77.736
G5	miR-381 mimic-1	miR-381	Unknown	19.594	0.993	1.865	AMP	1.633	3-11	77.885
G6	miR-381 mimic-1	miR-381	Unknown	19.381	0.983	1.886	AMP	1.633	3-12	78.034
H1	miR-381 mimic-2	U6	Unknown	18.172	0.976	1.824	AMP	1.861	3-11	83.552
H2	miR-381 mimic-2	U6	Unknown	17.984	0.993	1.882	AMP	1.861	3-11	83.403
H3	miR-381 mimic-2	U6	Unknown	17.799	0.993	1.876	AMP	1.861	3-10	83.103
H4	miR-381 mimic-2	miR-381	Unknown	19.592	0.984	1.883	AMP	1.633	3-13	77.885
H5	miR-381 mimic-2	miR-381	Unknown	19.675	0.99	1.878	AMP	1.633	3-11	77.885
H6	miR-381 mimic-2	miR-381	Unknown	19.683	0.991	1.869	AMP	1.633	3-12	78.034

Replicate Group Table

Sample	Target	No. of Replicates	Cq Mean	Cq SD
NC-1	U6	3	18.538	0.25
NC-1	miR-381	3	21.971	0.229
NC-2	U6	3	18.299	0.003
NC-2	miR-381	3	21.878	0.129
NC-3	U6	3	18.229	0.092
NC-3	miR-381	3	21.842	0.115
miR-381 inhi-1	U6	3	18.19	0.071
miR-381 inhi-1	miR-381	3	23.032	0.046
miR-381 inhi-2	U6	3	18.082	0.122
miR-381 inhi-2	miR-381	3	22.846	0.082
miR-381 inhi- 3	U6	3	17.964	0.281
miR-381 inhi- 3	miR-381	3	22.554	0.069
miR-381 mimic-1	U6	3	17.869	0.078
miR-381 mimic-1	miR-381	3	19.585	0.2
miR-381 mimic-2	U6	3	17.985	0.187
miR-381 mimic-2	miR-381	3	19.65	0.05
miR-381 mimic-3	U6	3	17.893	0.094
miR-381 mimic-3	miR-381	3	19.929	0.043

Plate Layout

	1	2	3	4	5	6	7	8	9	10	11	12
A	NC-1<br>U6 (18.826)	NC-1<br>U6 (18.386)	NC-1<br>U6 (18.403)	NC-1<br>miR-381 (22.227)	NC-1<br>miR-381 (21.785)	NC-1<br>miR-381 (21.902)	miR-381 mimic-3<br>U6 (17.992)	miR-381 mimic-3<br>U6 (17.884)	miR-381 mimic-3<br>U6 (17.804)	miR-381 mimic-3<br>miR-381 (19.938)	miR-381 mimic-3<br>miR-381 (19.883)	miR-381 mimic-3<br>miR-381 (19.966)
B	NC-2<br>U6 (18.301)	NC-2<br>U6 (18.296)	NC-2<br>U6 (18.299)	NC-2<br>miR-381 (21.874)	NC-2<br>miR-381 (22.009)	NC-2<br>miR-381 (21.752)						
C	NC-3<br>U6 (18.308)	NC-3<br>U6 (18.253)	NC-3<br>U6 (18.127)	NC-3<br>miR-381 (21.974)	NC-3<br>miR-381 (21.793)	NC-3<br>miR-381 (21.76)						
D	miR-381 inhi-1<br>U6 (18.114)	miR-381 inhi-1<br>U6 (18.198)	miR-381 inhi-1<br>U6 (18.256)	miR-381 inhi-1<br>miR-381 (23.085)	miR-381 inhi-1<br>miR-381 (23)	miR-381 inhi-1<br>miR-381 (23.012)						
E	miR-381 inhi-2<br>U6 (18.221)	miR-381 inhi-2<br>U6 (17.995)	miR-381 inhi-2<br>U6 (18.028)	miR-381 inhi-2<br>miR-381 (22.839)	miR-381 inhi-2<br>miR-381 (22.931)	miR-381 inhi-2<br>miR-381 (22.767)						
F	miR-381 inhi- 3<br>U6 (18.036)	miR-381 inhi- 3<br>U6 (18.202)	miR-381 inhi- 3<br>U6 (17.655)	miR-381 inhi- 3<br>miR-381 (22.617)	miR-381 inhi- 3<br>miR-381 (22.565)	miR-381 inhi- 3<br>miR-381 (22.481)						
G	miR-381 mimic-1<br>U6 (17.866)	miR-381 mimic-1<br>U6 (17.792)	miR-381 mimic-1<br>U6 (17.949)	miR-381 mimic-1<br>miR-381 (19.78)	miR-381 mimic-1<br>miR-381 (19.594)	miR-381 mimic-1<br>miR-381 (19.381)						
H	miR-381 mimic-2<br>U6 (18.172)	miR-381 mimic-2<br>U6 (17.984)	miR-381 mimic-2<br>U6 (17.799)	miR-381 mimic-2<br>miR-381 (19.592)	miR-381 mimic-2<br>miR-381 (19.675)	miR-381 mimic-2<br>miR-381 (19.683)						

Amplification Plot (dRn)

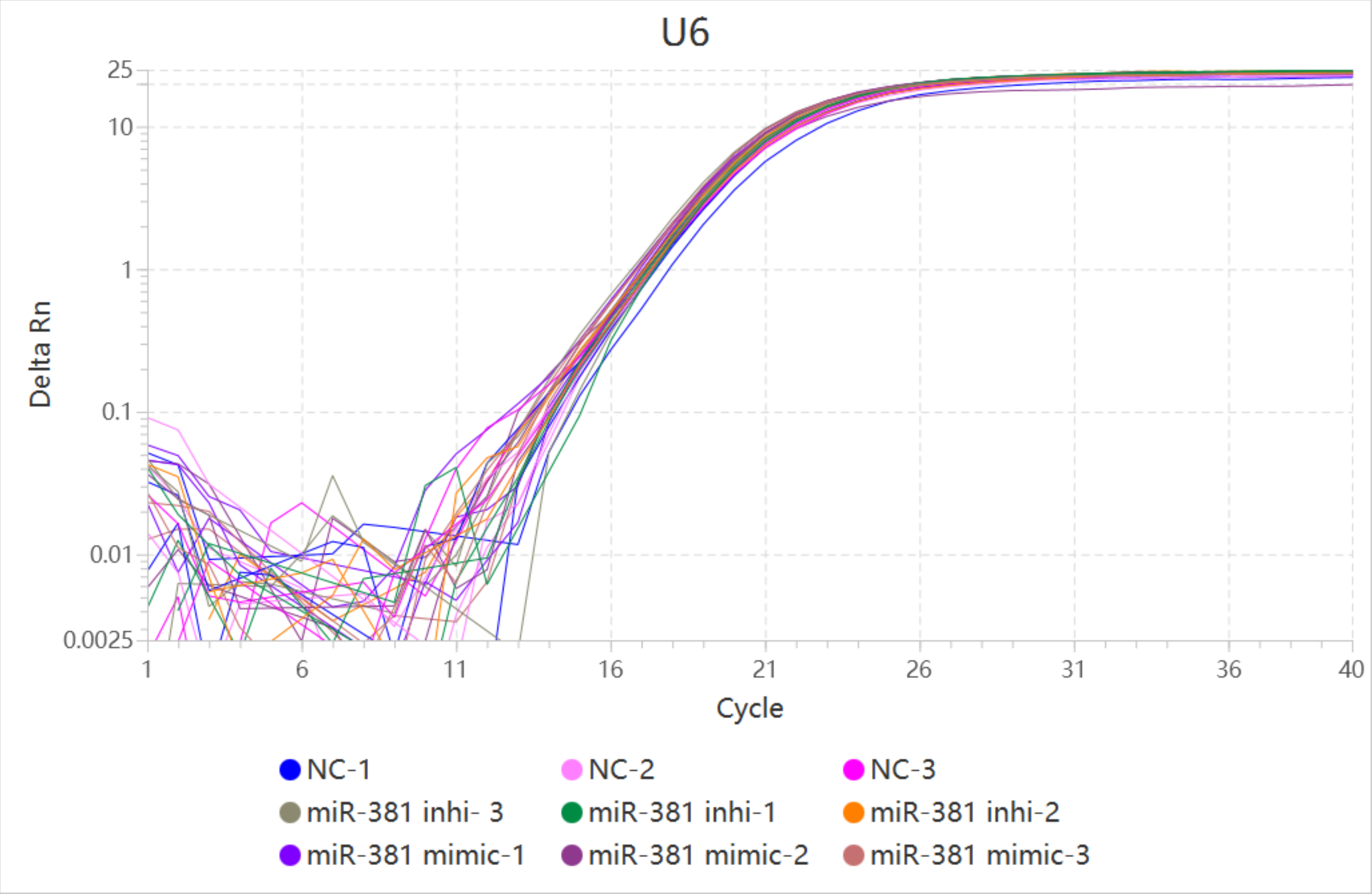

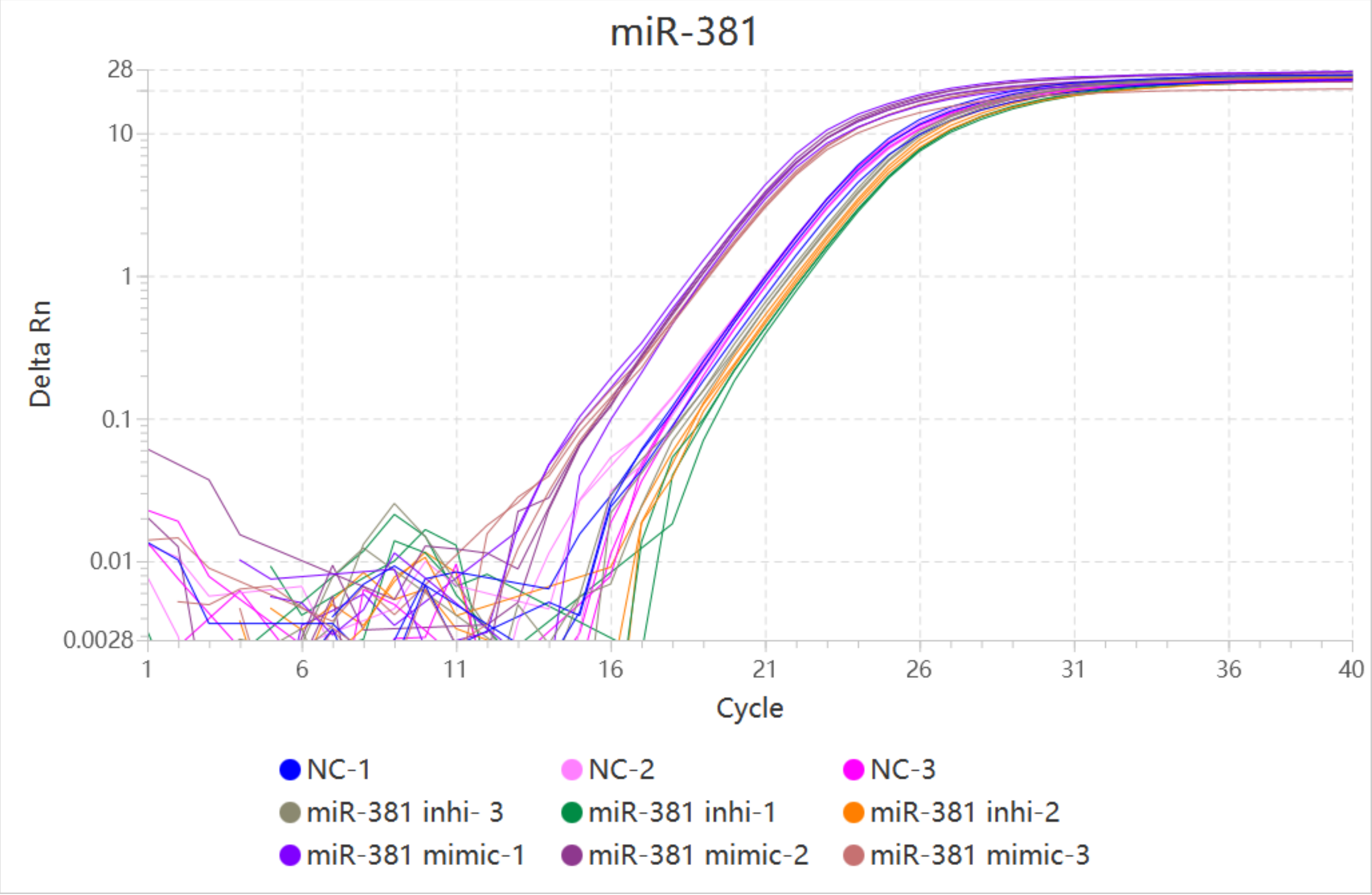

Amplification Plot (Rn)

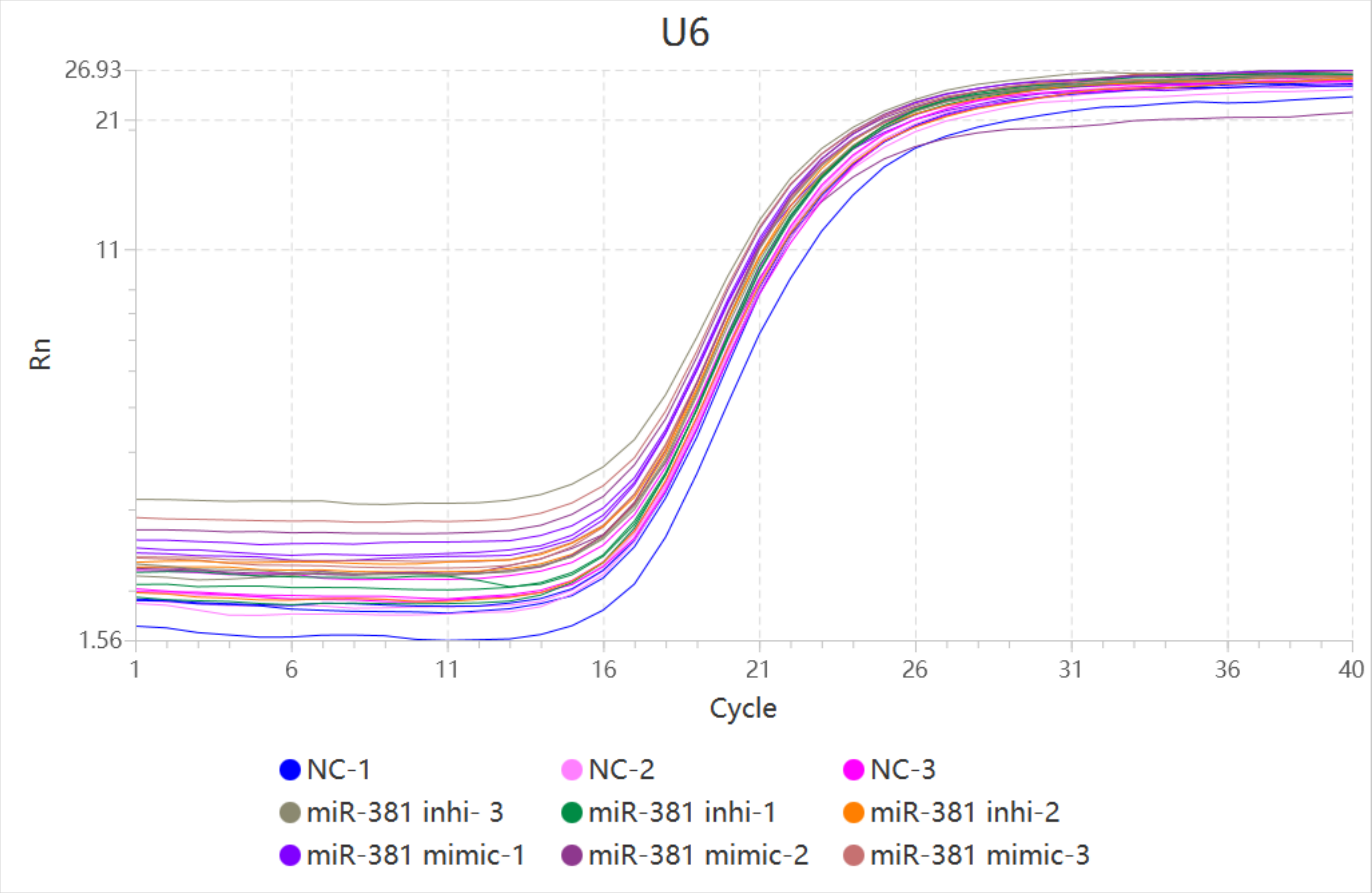

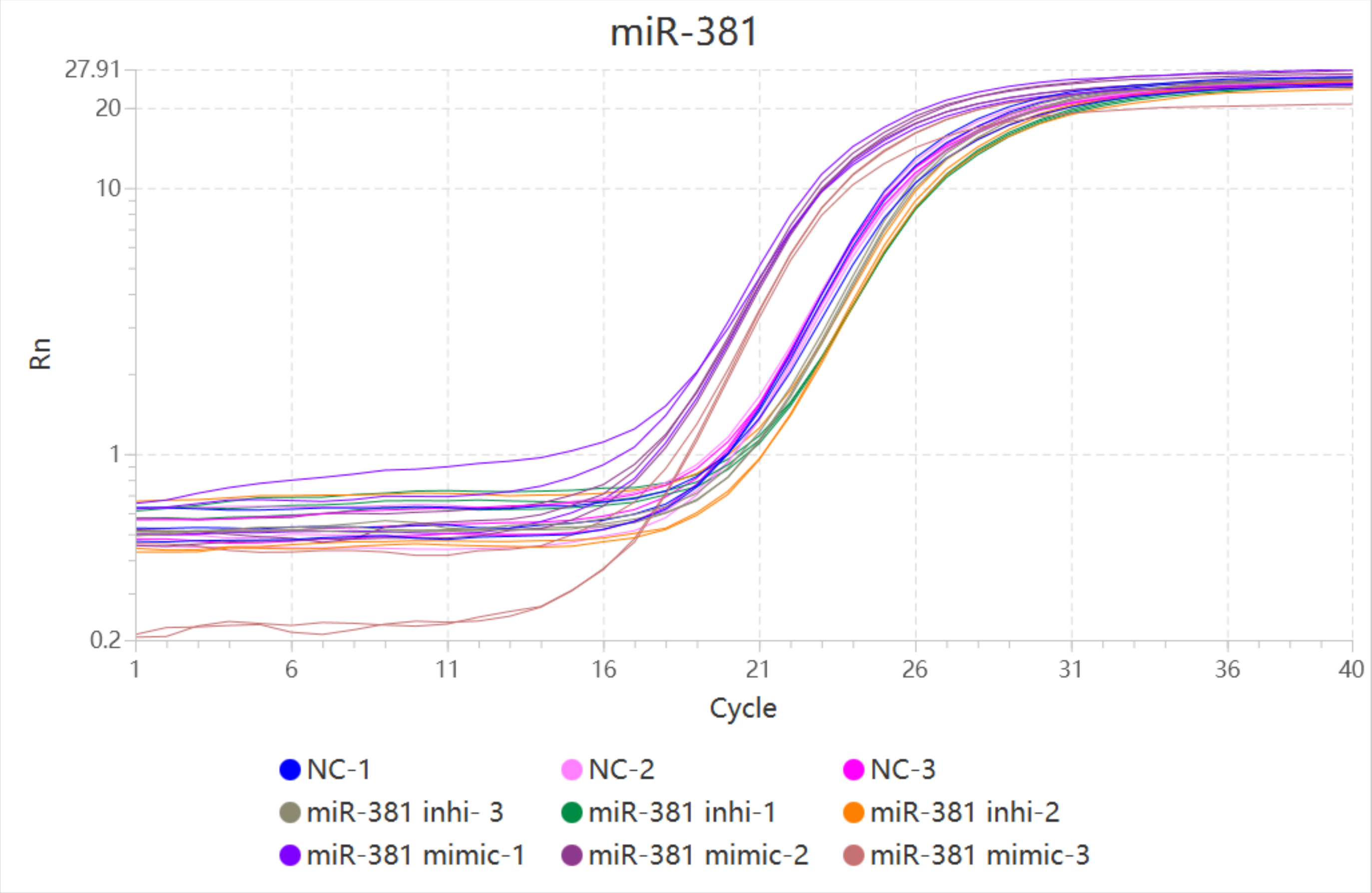

Melt Curve Plot

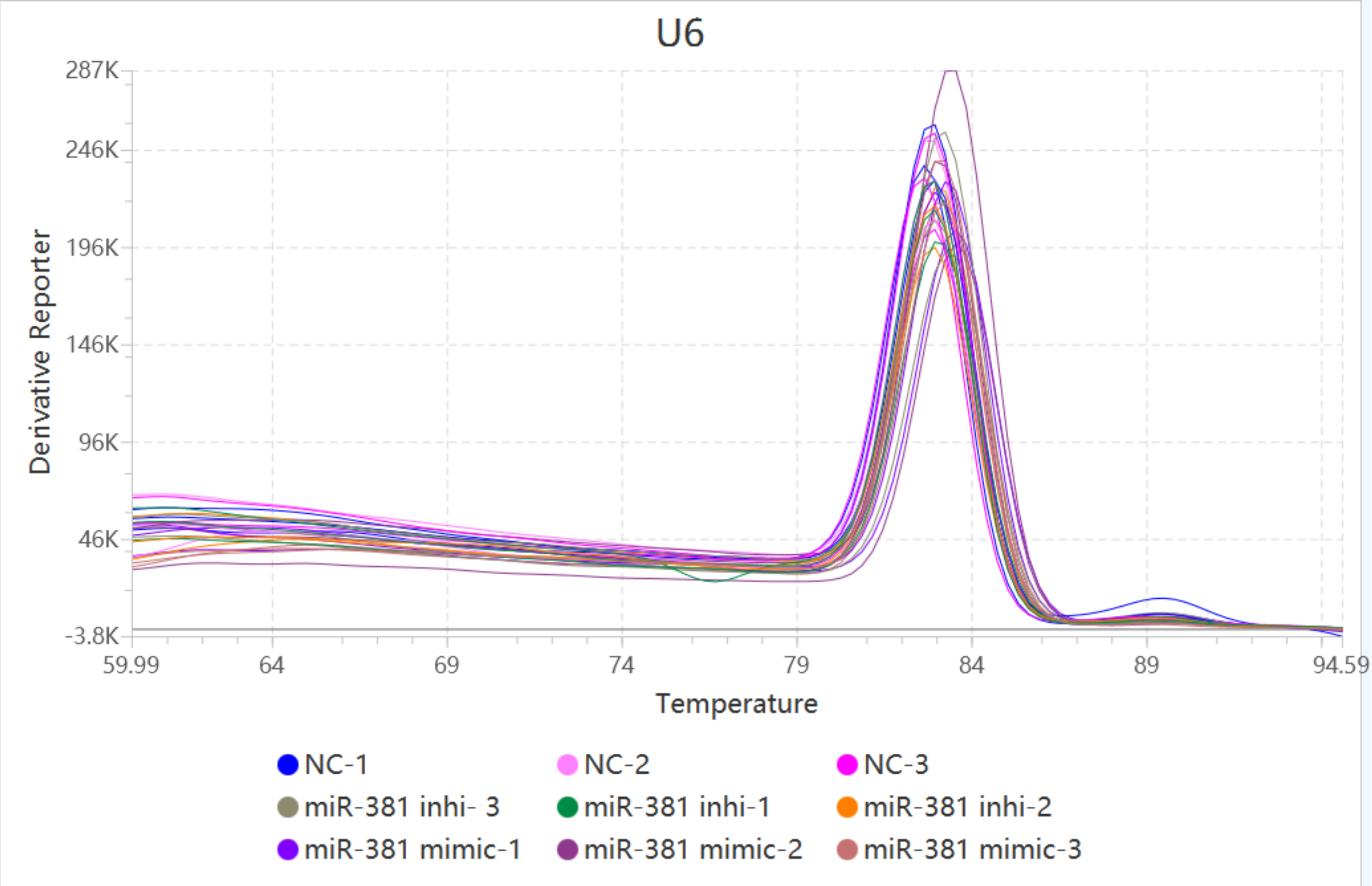

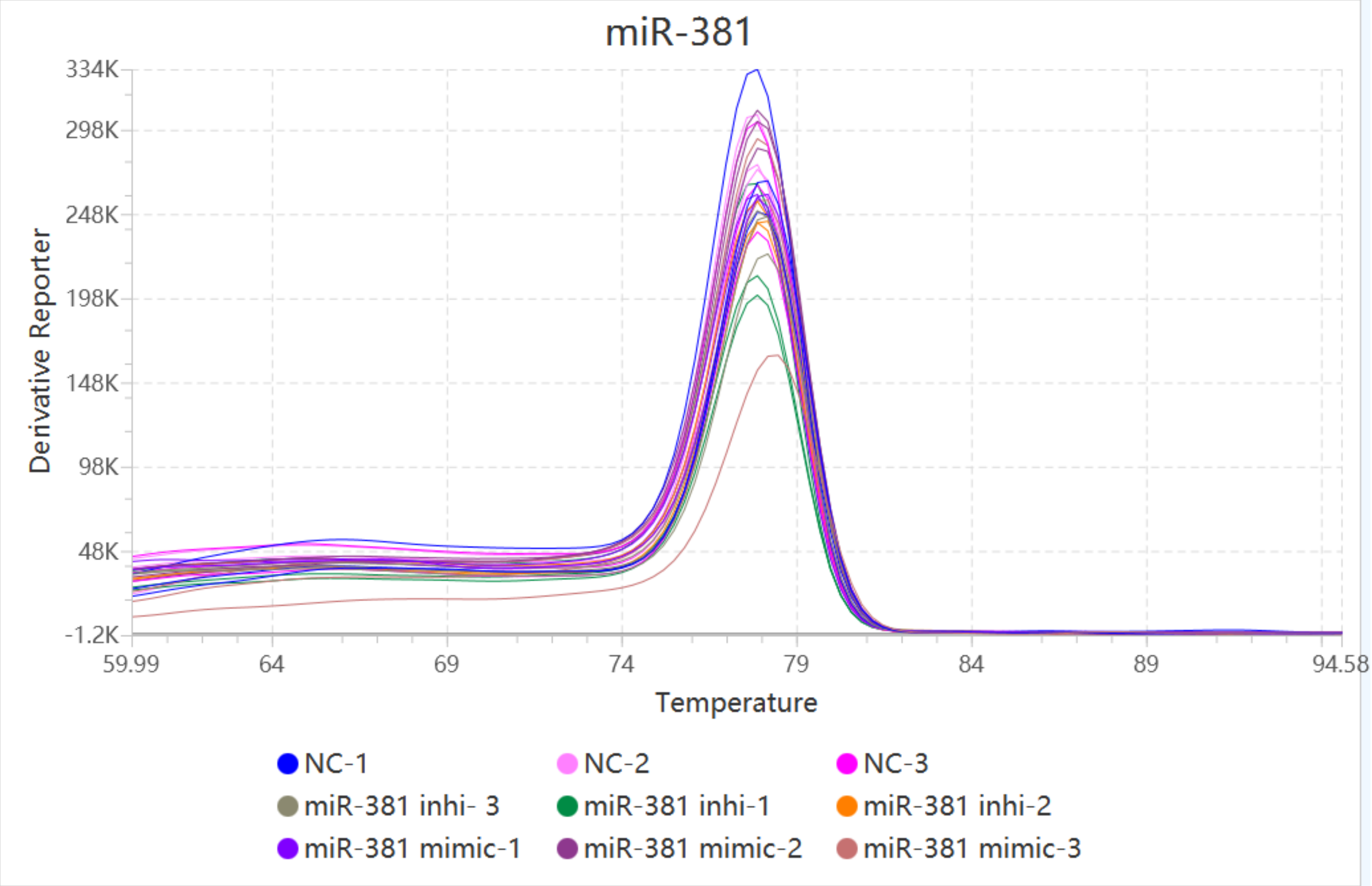

Run Method

Block Type	96-Well 0.2-mL Block
Sample Volume	20.0
Cover Temperature	105.0
Run mode	FAST

Stage	Collection Flag	Ramp Rate	Temperature	Hold Time	Starting Cycle	Auto Delta Temperature	Auto Delta Hold Time
Hold Stage	false	2.74°C/sec	95.0°C	20	-	-	-
PCR Stage (40 cycles)	false	2.74°C/sec	95.0°C	1	-	-	-
	true	2.12°C/sec	60.0°C	20	-	-	-
Melt Stage	false	2.74°C/sec	95.0°C	1	-	-	-
	false	2.12°C/sec	60.0°C	20	-	-	-
	true	0.15°C/sec	95.0°C	1	-	-	-

Primary Analysis Settings

General

PCR Stage/Step	Stage 2, Step 2
Quantification Cycle Method	Baseline Threshold

Target	Auto Threshold	Threshold	Auto Baseline	Baseline Start	Baseline End
DEFAULT	Yes	AUTO	Yes	AUTO	AUTO

Melt

Melt Stage/Step	Stage 3, Step 3

Target	Multi Peak	Threshold Type	Peak Level (%)	Peak Height
DEFAULT	Yes	Percentage	10	-
U6	Yes	Percentage	10	-
miR-381	Yes	Percentage	10	-

QC Alerts

Curve Quality Alert Enabled	No
Results Quality Alert Enabled	Yes

Advanced

Set the Delta-Rn below which curves will be considered Non-Amplified	No
Primary Analysis Variant	N/A

Relative Quantification Settings

General

RQ Min/Max Calculations	Confidence Level (95.0)
Max Allowed EqCq Mean	40
Include Adjusted EqCq Mean	No
Analysis Type	Singleplex

Endo Controls

Normalization Type	Specific endogenous control

Target	Endogenous Control	Auto	Efficiency(%)
U6	Yes	Yes	AUTO

References

Reference Sample	NC-1

Relative Quantification Results (Sample)

Sample	Target	EqCq Mean	Adjusted EqCq Mean	ΔEqCq Mean	ΔEqCq SD	ΔEqCq SE	ΔΔEqCq	RQ	RQ Min	RQ Max
NC-1	U6	18.538	18.538	-	-	-	-	-	-	-
NC-1	miR-381	21.971	21.971	3.433	0.339	0.196	-	1	0.686	1.457
miR-381 mimic-3	U6	17.893	17.893	-	-	-	-	-	-	-
miR-381 mimic-3	miR-381	19.929	19.929	2.036	0.104	0.06	-1.398	2.635	2.348	2.956
NC-2	U6	18.299	18.299	-	-	-	-	-	-	-
NC-2	miR-381	21.878	21.878	3.58	0.129	0.074	0.146	0.904	0.783	1.042
NC-3	U6	18.229	18.229	-	-	-	-	-	-	-
NC-3	miR-381	21.842	21.842	3.613	0.148	0.085	0.18	0.883	0.749	1.04
miR-381 inhi-1	U6	18.19	18.19	-	-	-	-	-	-	-
miR-381 inhi-1	miR-381	23.032	23.032	4.843	0.085	0.049	1.409	0.376	0.343	0.414
miR-381 inhi-2	U6	18.082	18.082	-	-	-	-	-	-	-
miR-381 inhi-2	miR-381	22.846	22.846	4.764	0.147	0.085	1.331	0.397	0.338	0.468
miR-381 inhi- 3	U6	17.964	17.964	-	-	-	-	-	-	-
miR-381 inhi- 3	miR-381	22.554	22.554	4.59	0.289	0.167	1.157	0.449	0.325	0.618
miR-381 mimic-1	U6	17.869	17.869	-	-	-	-	-	-	-
miR-381 mimic-1	miR-381	19.585	19.585	1.716	0.215	0.124	-1.717	3.288	2.59	4.173
miR-381 mimic-2	U6	17.985	17.985	-	-	-	-	-	-	-
miR-381 mimic-2	miR-381	19.65	19.65	1.665	0.194	0.112	-1.768	3.406	2.747	4.224

Relative Quantification Plot

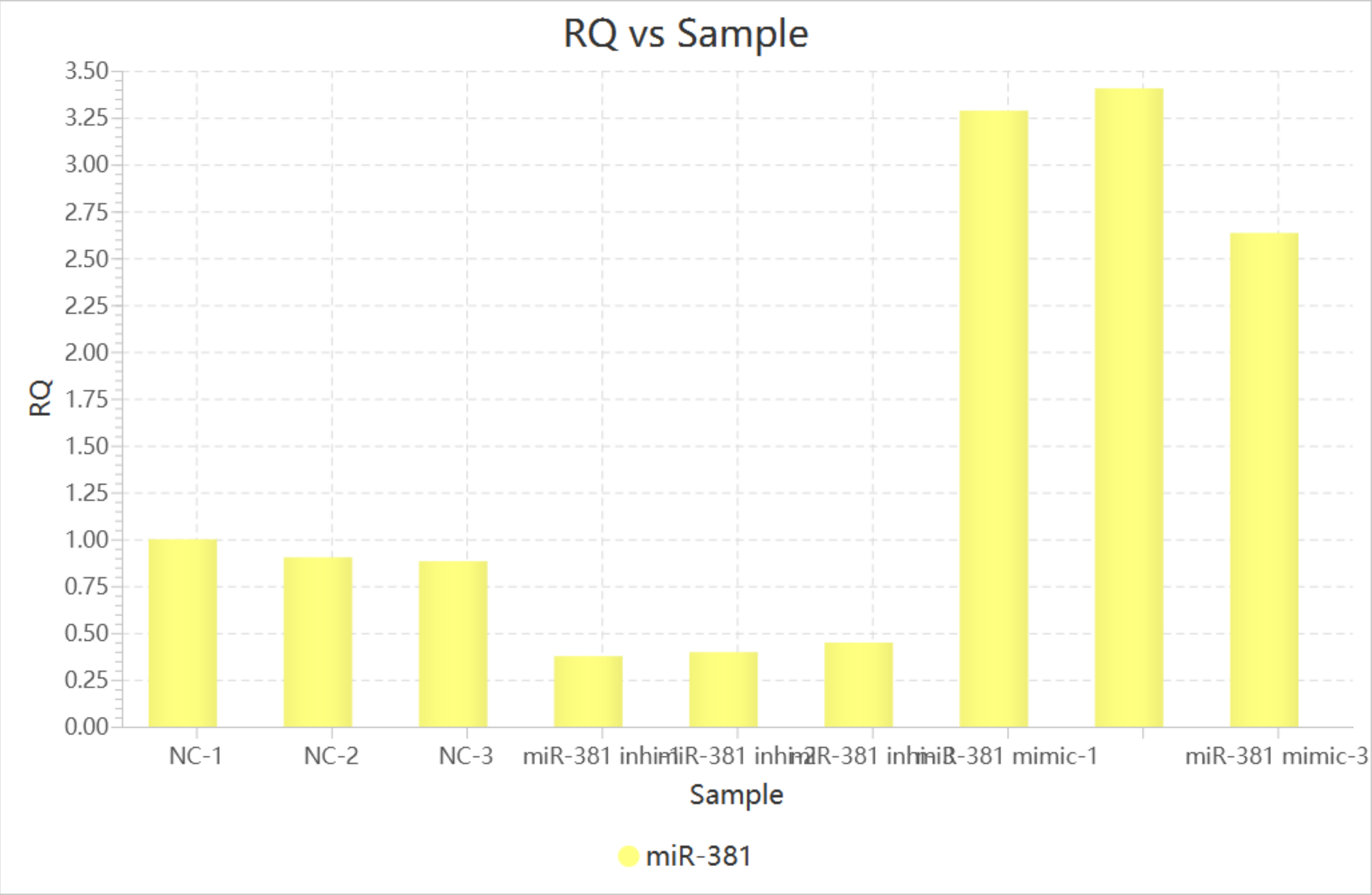

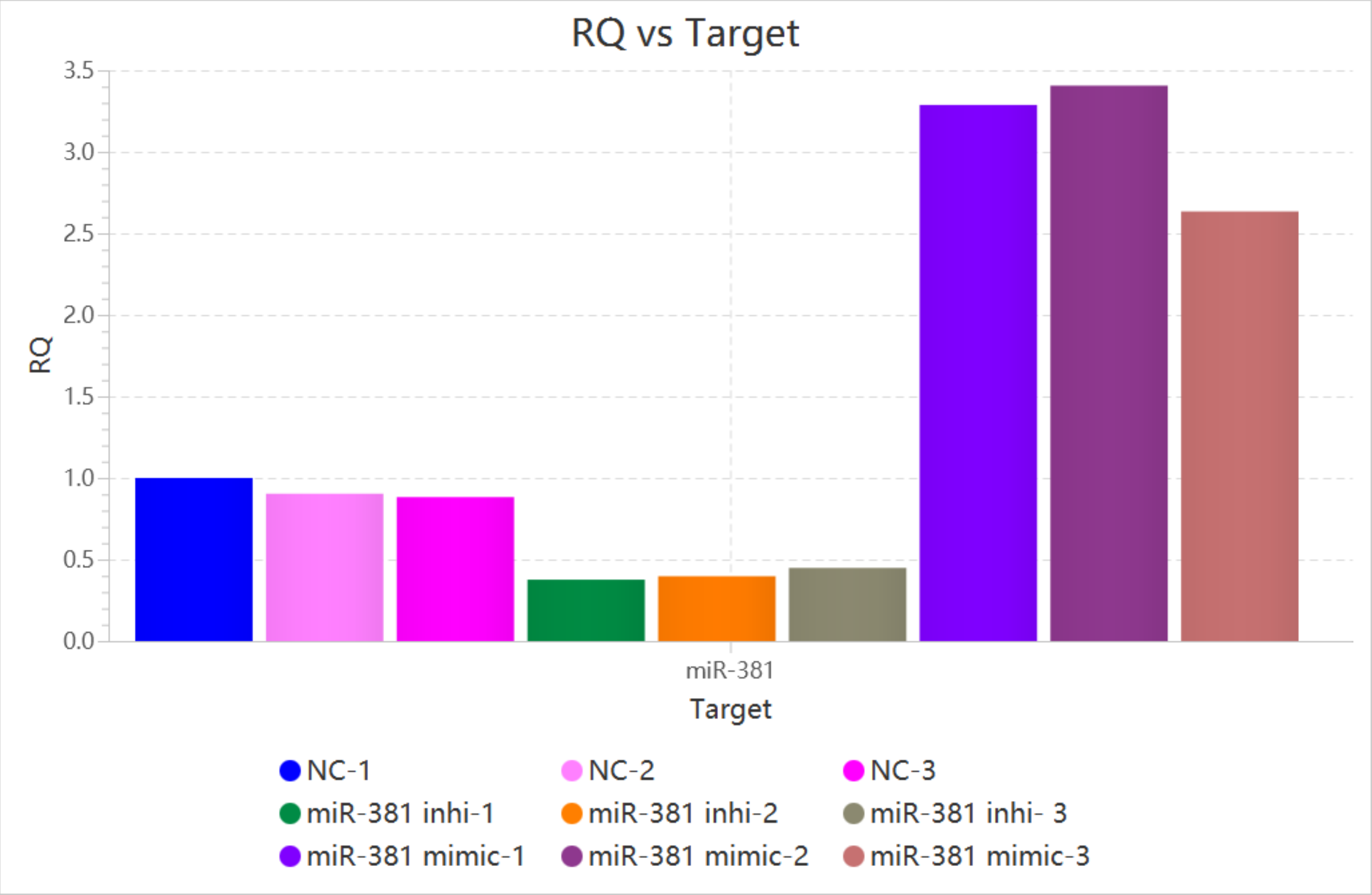

- End of Report -
