# Supplementary figures and images for: Combination therapy with budesonide and acetylcysteine alleviates LPS-induced acute lung injury via the miR-381/NLRP3 molecular axis (part 2 of 2)
Source: PLoS One. 2023 Aug 9;18(8):e0289818. doi: 10.1371/journal.pone.0289818 (PMC10411794; doi:10.1371/journal.pone.0289818)

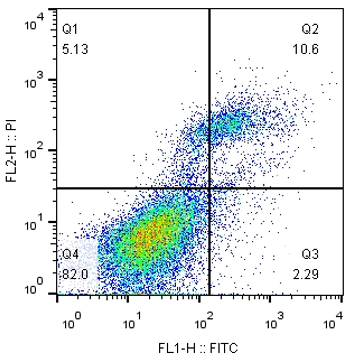

Supplement: S5 File — (ZIP) [file pone.0289818.s005.zip › S5 File. Fig5 Original data/image/5C/LPS+Bud+NAC+miR-381 inhi/1 (3).jpg]

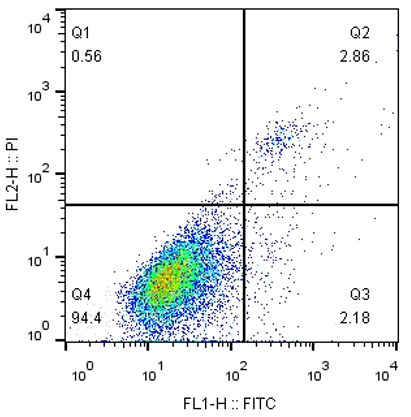

Supplement: S5 File — (ZIP) [file pone.0289818.s005.zip › S5 File. Fig5 Original data/image/5C/NC/1 (1).jpg]

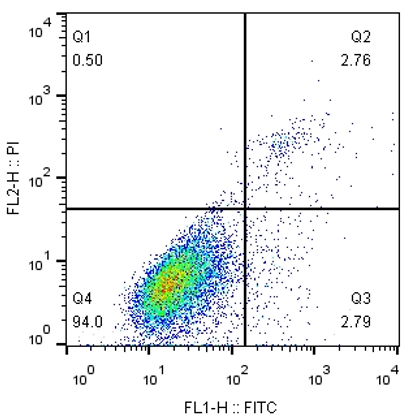

Supplement: S5 File — (ZIP) [file pone.0289818.s005.zip › S5 File. Fig5 Original data/image/5C/NC/1 (2).jpg]

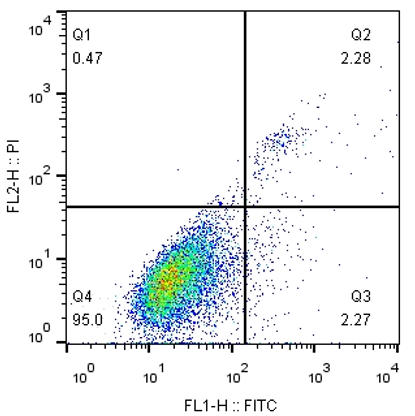

Supplement: S5 File — (ZIP) [file pone.0289818.s005.zip › S5 File. Fig5 Original data/image/5C/NC/1 (3).jpg]

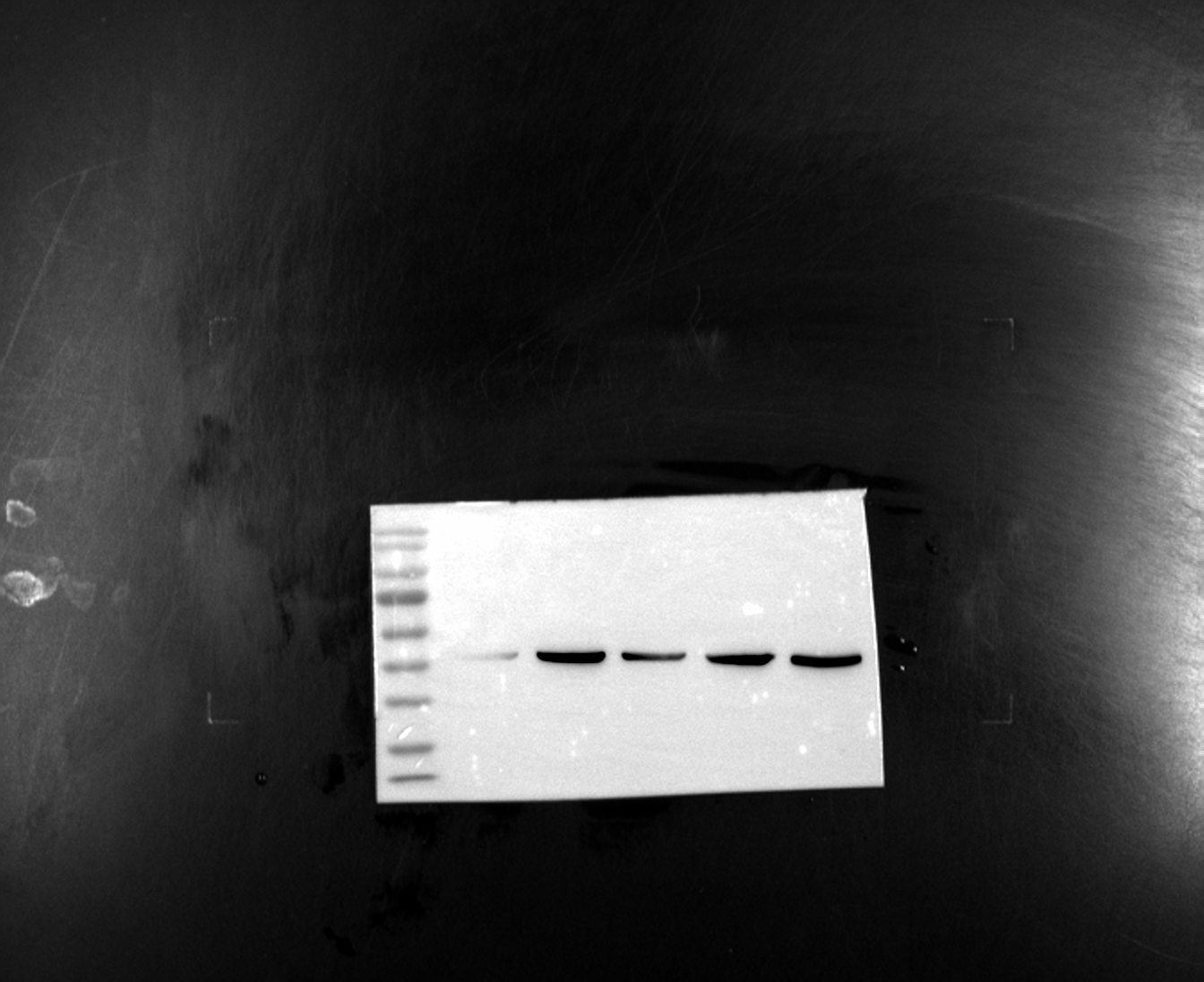

Supplement: S5 File — (ZIP) [file pone.0289818.s005.zip › S5 File. Fig5 Original data/image/5D/1.Caspase 1.tif]

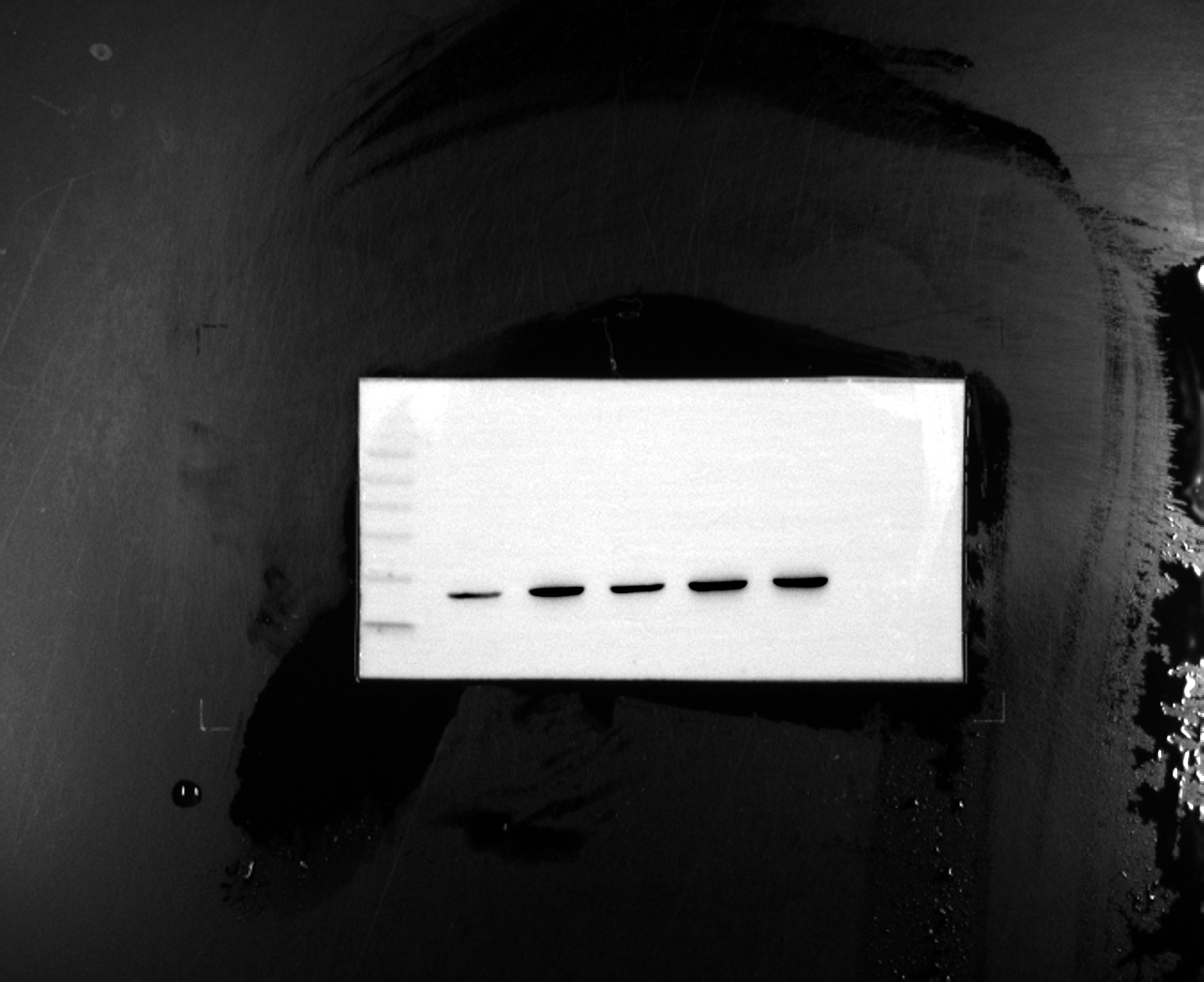

Supplement: S5 File — (ZIP) [file pone.0289818.s005.zip › S5 File. Fig5 Original data/image/5D/2.ASC.tif]

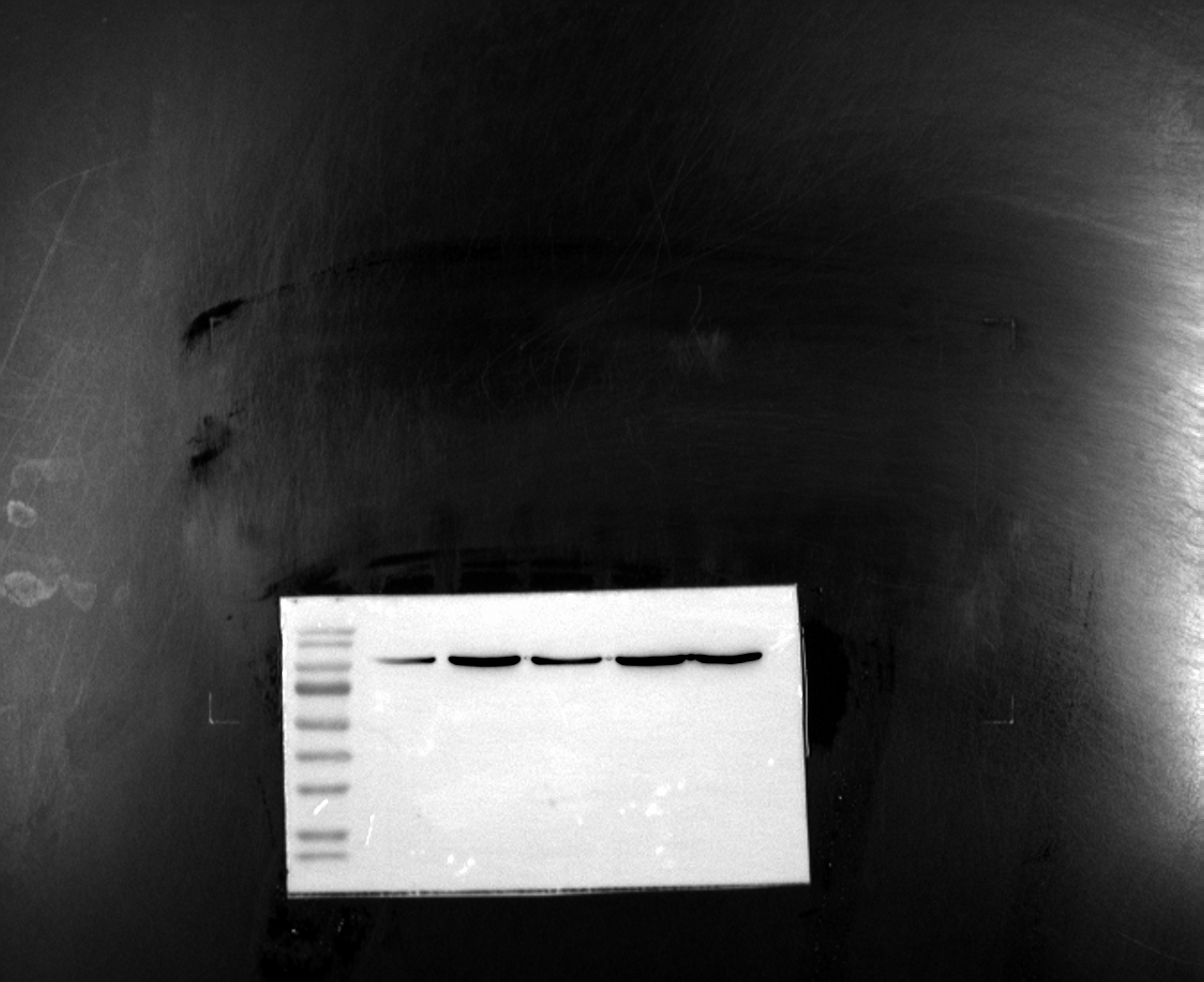

Supplement: S5 File — (ZIP) [file pone.0289818.s005.zip › S5 File. Fig5 Original data/image/5D/3.NLRP3.tif]

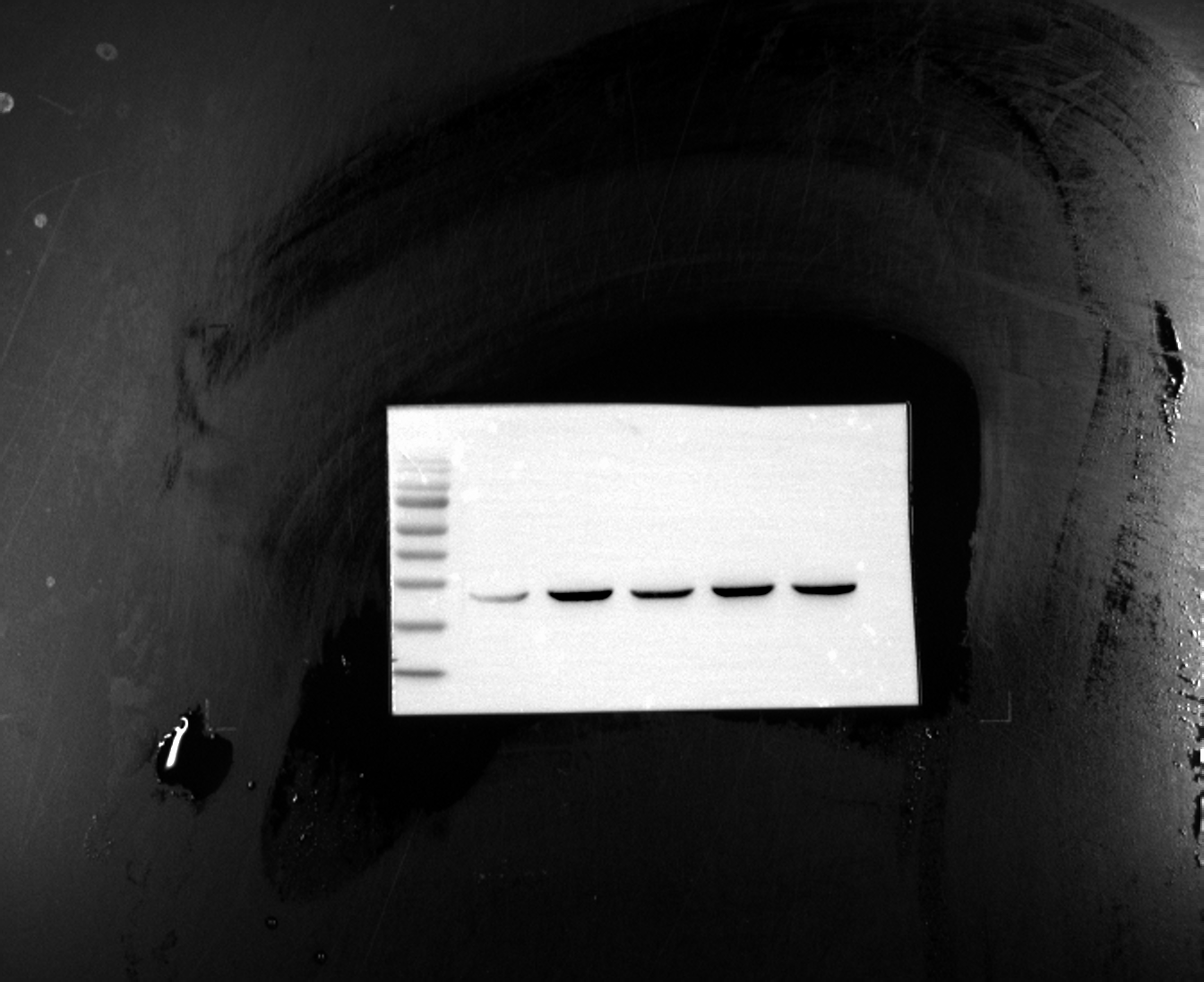

Supplement: S5 File — (ZIP) [file pone.0289818.s005.zip › S5 File. Fig5 Original data/image/5D/4.IL-1β.tif]

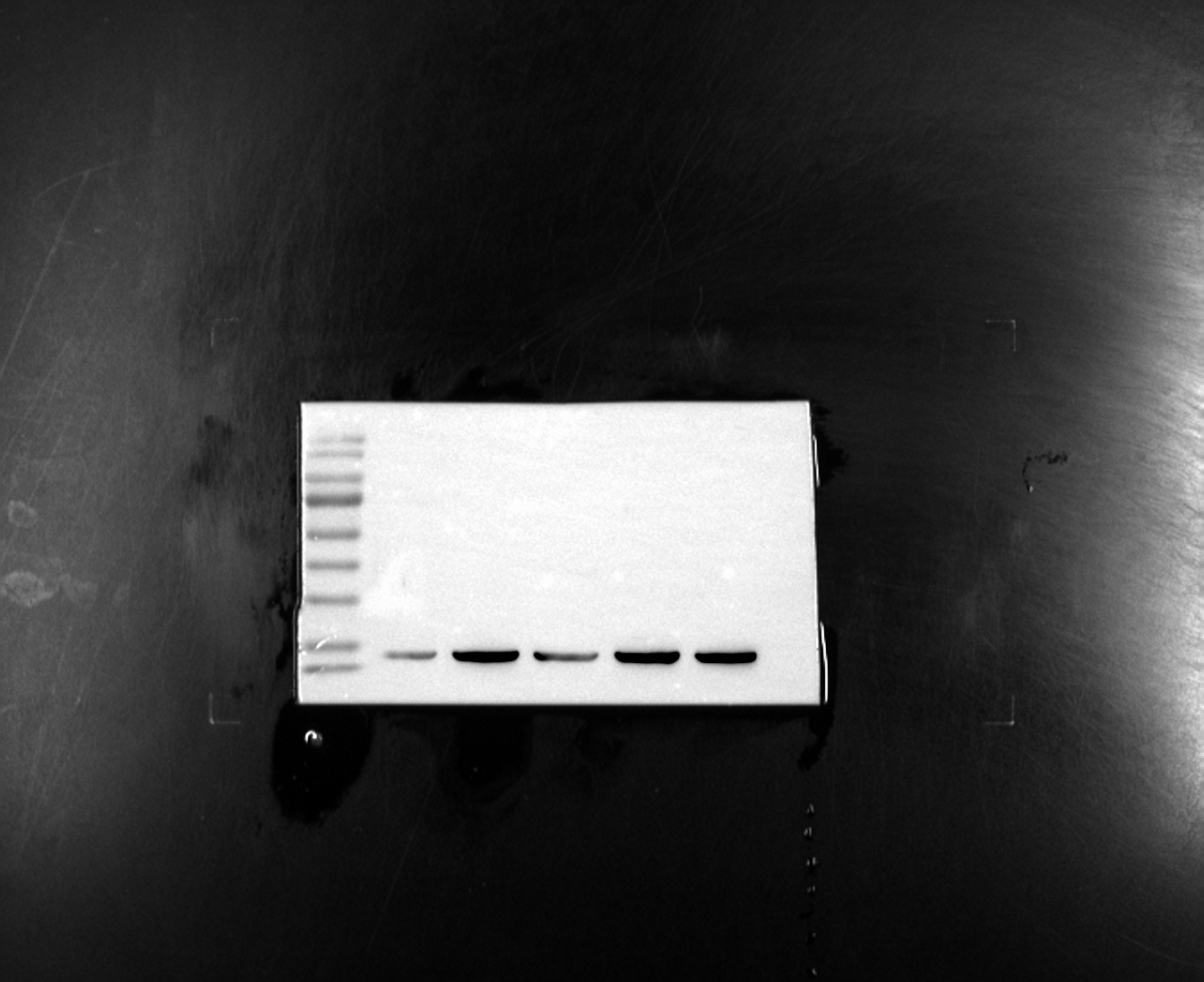

Supplement: S5 File — (ZIP) [file pone.0289818.s005.zip › S5 File. Fig5 Original data/image/5D/5.IL-18.tif]

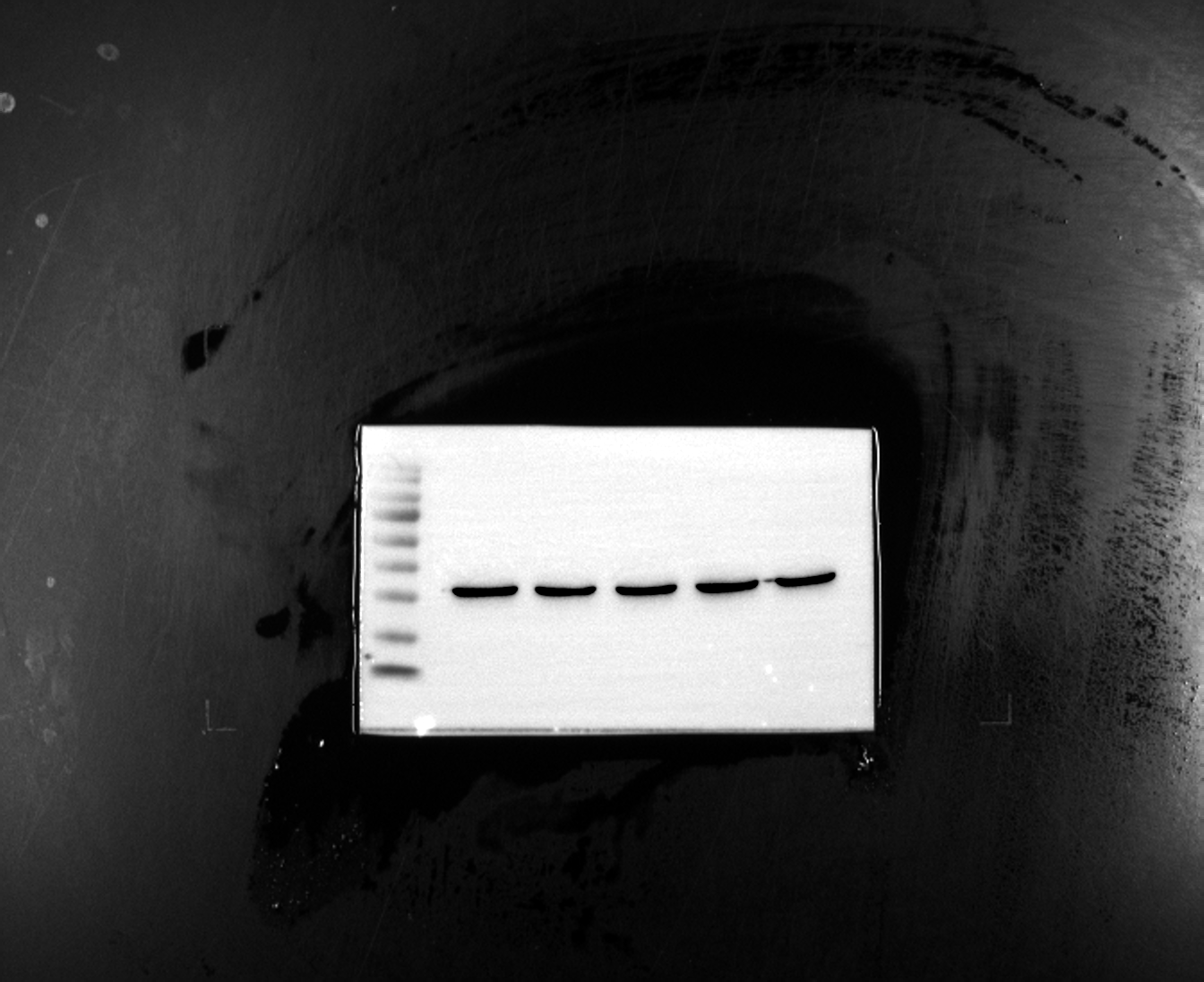

Supplement: S5 File — (ZIP) [file pone.0289818.s005.zip › S5 File. Fig5 Original data/image/5D/6.GAPDH.tif]
