# Supplementary material for: Combination therapy with budesonide and acetylcysteine alleviates LPS-induced acute lung injury via the miR-381/NLRP3 molecular axis
Source: PLoS One. 2023 Aug 9;18(8):e0289818. doi: 10.1371/journal.pone.0289818 (PMC10411794; doi:10.1371/journal.pone.0289818)
Supplement: S1 Raw images — (PDF) [file pone.0289818.s006.pdf]

1E

1. 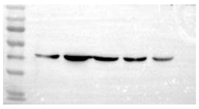

Caspase-1: 42kd. Marker (Vazyme, Mp102-02). Sample loading order: Marker, NC, ALI, Bud, NAC, Bud+NAC. Exposure method : Luminescent liquid (meilunbio, Mao186-Feb-281) , Tennant Chemiluminescent Instrument (Tanon, 4600), automatic exposure.

2. 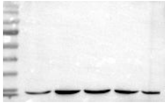

ASC: 22kd. Marker (Vazyme, Mp102-02). Sample loading order: Marker, NC, ALI, Bud, NAC, Bud+NAC. Exposure method : Luminescent liquid (meilunbio, Mao186-Feb-281) , Tennant Chemiluminescent Instrument (Tanon, 4600), automatic exposure.

3. 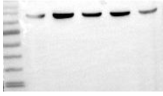

NLRP3: 118kd. Marker (Vazyme, Mp102-02). Sample loading order: Marker, NC, ALI, Bud, NAC, Bud+NAC. Exposure method : Luminescent liquid (meilunbio, Mao186-Feb-281) , Tennant Chemiluminescent Instrument (Tanon, 4600), automatic exposure.

4. 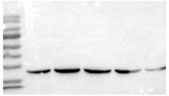

IL-1β: 31kd. Marker (Vazyme, Mp102-02). Sample loading order: Marker, NC, ALI, Bud, NAC, Bud+NAC. Exposure method : Luminescent liquid (meilunbio, Mao186-Feb-281) , Tennant Chemiluminescent Instrument (Tanon, 4600), automatic exposure.

5. 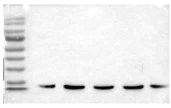

IL-18: 22kd. Marker (Vazyme, Mp102-02). Sample loading order: Marker, NC, ALI, Bud, NAC, Bud+NAC. Exposure method : Luminescent liquid (meilunbio, Mao186-Feb-281) , Tennant Chemiluminescent Instrument (Tanon, 4600), automatic exposure.

6. 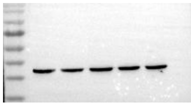

GAPDH: 36kd. Marker (Vazyme, Mp102-02). Sample loading order: Marker, NC, ALI, Bud, NAC, Bud+NAC. Exposure method : Luminescent liquid (meilunbio, Mao186-Feb-281) , Tennant Chemiluminescent Instrument (Tanon, 4600), automatic exposure.

2C

1. 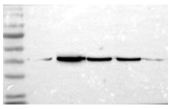

Caspase-1: 42kd. Marker (Vazyme, Mp102-02). Sample loading order: Marker, NC, LPS, LPS+Bud, LPS+NAC, LPS+Bud+NAC. Exposure method：Luminescent liquid（meilunbio, Mao186-Feb-281）, Tennant Chemiluminescent Instrument (Tanon, 4600)，automatic exposure.

2. 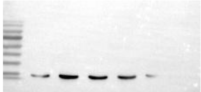

ASC: 22kd. Marker (Vazyme, Mp102-02). Sample loading order: Marker, NC, LPS, LPS+Bud, LPS+NAC, LPS+Bud+NAC. Exposure method：Luminescent liquid（meilunbio, Mao186-Feb-281）, Tennant Chemiluminescent Instrument (Tanon, 4600)，automatic exposure.

3. 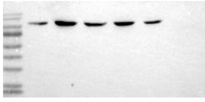

NLRP3: 118kd. Marker (Vazyme, Mp102-02). Sample loading order: Marker, NC, LPS, LPS+Bud, LPS+NAC, LPS+Bud+NAC. Exposure method：Luminescent liquid（meilunbio, Mao186-Feb-281）, Tennant Chemiluminescent Instrument (Tanon, 4600)，automatic exposure.

4. 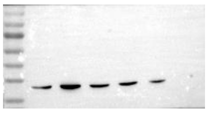

IL-1β: 31kd. Marker (Vazyme, Mp102-02). Sample loading order: Marker, NC, LPS, LPS+Bud, LPS+NAC, LPS+Bud+NAC. Exposure method：Luminescent liquid（meilunbio, Mao186-Feb-281）, Tennant Chemiluminescent Instrument (Tanon, 4600)，automatic exposure.

5. 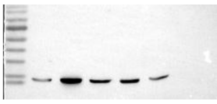

IL-18: 22kd. Marker (Vazyme, Mp102-02). Sample loading order: Marker, NC, LPS, LPS+Bud, LPS+NAC, LPS+Bud+NAC. Exposure method：Luminescent liquid（meilunbio, Mao186-Feb-281）, Tennant Chemiluminescent Instrument (Tanon, 4600)，automatic exposure.

6. 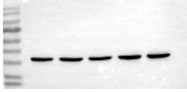

GAPDH: 36kd. Marker (Vazyme, Mp102-02). Sample loading order: Marker, NC, LPS, LPS+Bud, LPS+NAC, LPS+Bud+NAC. Exposure method：Luminescent liquid（meilunbio, Mao186-Feb-281）, Tennant Chemiluminescent Instrument (Tanon, 4600)，automatic exposure.

3F

1. 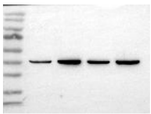

Caspase-1: 42kd. Marker (Vazyme, Mp102-02). Sample loading order: Marker, NC, LPS, LPS+Bud+NAC, LPS+Bud+NAC+miR-381 inhi. Exposure method: Luminescent liquid (meilunbio, Mao186-Feb-281), Tennant Chemiluminescent Instrument (Tanon, 4600), automatic exposure.

2. 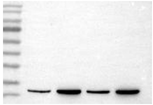

ASC: 22kd. Marker (Vazyme, Mp102-02). Sample loading order: Marker, NC, LPS, LPS+Bud+NAC, LPS+Bud+NAC+miR-381 inhi. Exposure method: Luminescent liquid (meilunbio, Mao186-Feb-281), Tennant Chemiluminescent Instrument (Tanon, 4600), automatic exposure.

3. 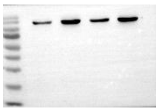

NLRP3: 118kd. Marker (Vazyme, Mp102-02). Sample loading order: Marker, NC, LPS, LPS+Bud+NAC, LPS+Bud+NAC+miR-381 inhi. Exposure method: Luminescent liquid (meilunbio, Mao186-Feb-281), Tennant Chemiluminescent Instrument (Tanon, 4600), automatic exposure.

4. 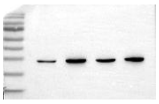

IL-1β: 31kd. Marker (Vazyme, Mp102-02). Sample loading order: Marker, NC, LPS, LPS+Bud+NAC, LPS+Bud+NAC+miR-381 inhi. Exposure method: Luminescent liquid (meilunbio, Mao186-Feb-281), Tennant Chemiluminescent Instrument (Tanon, 4600), automatic exposure.

5. 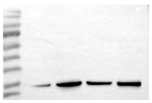

IL-18: 22kd. Marker (Vazyme, Mp102-02). Sample loading order: Marker, NC, LPS, LPS+Bud+NAC, LPS+Bud+NAC+miR-381 inhi. Exposure method: Luminescent liquid (meilunbio, Mao186-Feb-281), Tennant Chemiluminescent Instrument (Tanon, 4600), automatic exposure.

6. 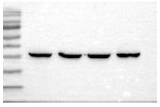

GAPDH: 36kd. Marker (Vazyme, Mp102-02). Sample loading order: Marker, NC, LPS, LPS+Bud+NAC, LPS+Bud+NAC+miR-381 inhi. Exposure method: Luminescent liquid (meilunbio, Mao186-Feb-281), Tennant Chemiluminescent Instrument (Tanon, 4600), automatic exposure.

4C

1. 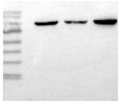

NLRP3: 118kd. Marker (Vazyme, Mp102-02). Sample loading order: Marker, NC, miR-381 mimic, miR-381 inhi. Exposure method：Luminescent liquid （meilunbio, Mao186-Feb-281），Tennant Chemiluminescent Instrument (Tanon, 4600)，automatic exposure.

2. 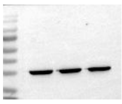

GAPDH: 36kd. Marker (Vazyme, Mp102-02). Sample loading order: Marker, NC, miR-381 mimic, miR-381 inhi. Exposure method：Luminescent liquid （meilunbio, Mao186-Feb-281），Tennant Chemiluminescent Instrument (Tanon, 4600)，automatic exposure.

5D

1. 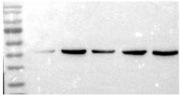

Caspase-1: 42kd. Marker (Vazyme, Mp102-02). Sample loading order: Marker, NC, LPS, LPS+Bud+NAC, LPS+Bud+NAC+miR-381 inhi, LPS+Bud+NAC+OE-NLRP3. Exposure method： Luminescent liquid （meilunbio, Mao186-Feb-281）, Tennant Chemiluminescent Instrument (Tanon, 4600)， automatic exposure.

2. 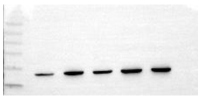

ASC: 22kd. Marker (Vazyme, Mp102-02). Sample loading order: Marker, NC, LPS, LPS+Bud+NAC, LPS+Bud+NAC+miR-381 inhi, LPS+Bud+NAC+OE-NLRP3. Exposure method： Luminescent liquid （meilunbio, Mao186-Feb-281）, Tennant Chemiluminescent Instrument (Tanon, 4600)， automatic exposure.

3. 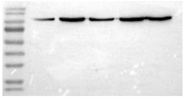

NLRP3: 118kd. Marker (Vazyme, Mp102-02). Sample loading order: Marker, NC, LPS, LPS+Bud+NAC, LPS+Bud+NAC+miR-381 inhi, LPS+Bud+NAC+OE-NLRP3. Exposure method： Luminescent liquid （meilunbio, Mao186-Feb-281）, Tennant Chemiluminescent Instrument (Tanon, 4600)， automatic exposure.

4. 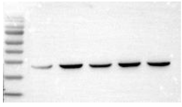

IL-1β: 31kd. Marker (Vazyme, Mp102-02). Sample loading order: Marker, NC, LPS, LPS+Bud+NAC, LPS+Bud+NAC+miR-381 inhi, LPS+Bud+NAC+OE-NLRP3. Exposure method： Luminescent liquid （meilunbio, Mao186-Feb-281）, Tennant Chemiluminescent Instrument (Tanon, 4600)， automatic exposure.

5. 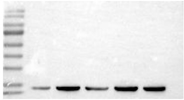

IL-18: 22kd. Marker (Vazyme, Mp102-02). Sample loading order: Marker, NC, LPS, LPS+Bud+NAC, LPS+Bud+NAC+miR-381 inhi, LPS+Bud+NAC+OE-NLRP3. Exposure method： Luminescent liquid （meilunbio, Mao186-Feb-281）, Tennant Chemiluminescent Instrument (Tanon, 4600)， automatic exposure.

6. 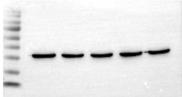

GAPDH: 36kd. Marker (Vazyme, Mp102-02). Sample loading order: Marker, NC, LPS, LPS+Bud+NAC, LPS+Bud+NAC+miR-381 inhi, LPS+Bud+NAC+OE-NLRP3. Exposure method： Luminescent liquid （meilunbio, Mao186-Feb-281）, Tennant Chemiluminescent Instrument (Tanon, 4600)， automatic exposure.
